# Radical α-Trifluoromethoxylation of Ketones under Batch and Flow Conditions by Means of Organic Photoredox Catalysis

**DOI:** 10.1021/acs.orglett.1c02494

**Published:** 2021-09-01

**Authors:** Thibaut Duhail, Tommaso Bortolato, Javier Mateos, Elsa Anselmi, Benson Jelier, Antonio Togni, Emmanuel Magnier, Guillaume Dagousset, Luca Dell’Amico

**Affiliations:** † Université Paris-Saclay, UVSQ, CNRS, UMR 8180, Institut Lavoisier de Versailles, 78035 Versailles Cedex, France; ‡ Department of Chemical Sciences, University of Padova, Via Marzolo 1, 35131 Padova, Italy; § Université de Tours, Faculté des Sciences et Techniques, 37200 Tours, France; ∥ Department of Chemistry and Applied Biosciences, Swiss Federal Institute of Technology, ETH Zurich, Vladimir-Prelog-Weg 2, 8093 Zurich, Switzerland

## Abstract

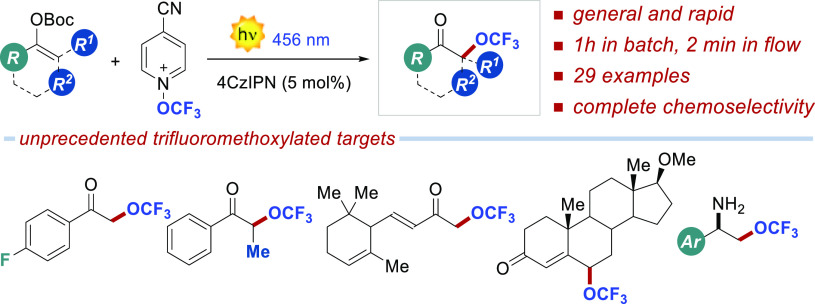

The first light-driven method for the α-trifluoromethoxylation of ketones is reported. Enol carbonates react with *N*-trifluoromethoxy-4-cyano-pyridinium, using the photoredox catalyst 4-CzIPN under 456 nm irradiation, affording the α-trifluoromethoxy ketones in ≤50% isolated yield and complete chemoselectivity. As shown by 29 examples, the reaction is general and proceeds very rapidly under batch (1 h) and flow conditions (2 min). Diverse product manipulations demonstrate the synthetic potential of the disclosed method in accessing elusive trifluoromethoxylated bioactive ingredients.

Among the rapidly emerging perfluorinated groups whose introduction into organic structures is of great interest, the OCF_3_ moiety occupies a very special place.^1^ Electronic and steric properties are among the main reasons for the popularity of this group. It brings indeed a high lipophilicity (Hansch parameter π = +1.04)^2^ to the molecules and possesses a high electronegativity (Pauling’s electronegativity scale χ = 3.7) that has earned it the nickname of superhalogen.^3^ These remarkable physicochemical properties associated with good metabolic stability and unique conformational properties make this group particularly attractive for the life sciences.^4^ Despite this, the number of marketed pharmaceutical and agrochemical products containing OCF_3_ remains low. To date, only four of the 340 identified drugs containing at least one fluorine atom bear a OCF_3_ group (Figure 1).^5^ Among the 424 fluoro-agrochemicals, 10 with OCF_3_ are listed.^6^ It should also be pointed out that for these 14 commercial molecules the OCF_3_ is always attached to an aromatic ring. This contrasting situation is mainly due to the small number of existing methods and/or the lack of reagents capable of delivering this functional target under selective conditions at the intermediate or late stage of a synthetic route. Pioneering works have focused on the construction of the O–CF_3_ bond from the already installed OH group, via (i) multistep processes, requiring harsh conditions and toxic reagents (e.g., HF and SF_4_),^7^ or (ii) direct electrophilic trifluoromethylation of alcohols, either with hypervalent iodine reagents that require large excesses of alcohol (5–75 equiv) to achieve reasonable yields or with an unstable oxonium salt.^8^

**Figure 1:**
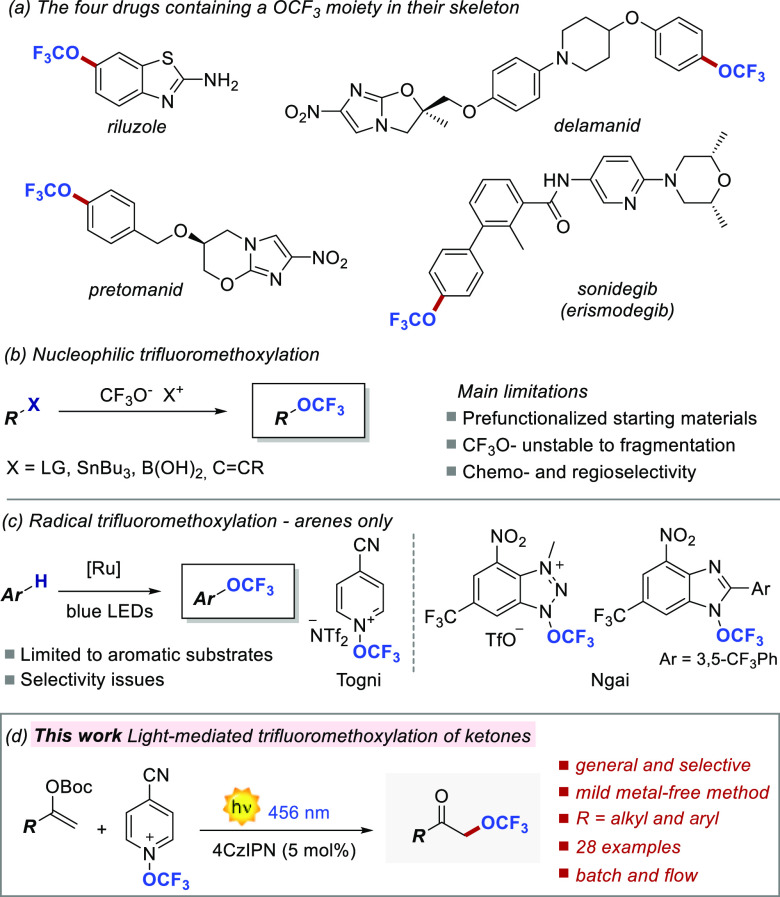
Drugs containing the OCF_3_ group and currently available trifluoromethoxylation processes.

Despite recent improvements,^9^ such approaches are still limited in practicality and scope. An elegant alternative for accessing trifluoromethoxylated compounds is the direct introduction of the OCF_3_ functionality (Figure 1a). To this end, nucleophilic routes have been proposed, with recent leading advances involving the description of new sources of the trifluoromethoxide anion or new methods for its in situ formation (Figure 1b).^10^ However, the use of this approach is hampered by the need for a prefunctionalized starting material reagent, the innate instability of the OCF_3_ anion, and the low chemo- and regioselectivity. Previously unknown, the radical approach emerged in 2018 and has seen rapid development,^11^ in particular with the invention of three new reagents (Figure 1c). One of us designed a pyridine *N*-oxide reagent,^12^ and the group of Ngai reported the use of azole-based compounds.^13^ Under photoredox conditions, these three reagents proved to be efficient for the catalytic C–H trifluoromethoxylation of arenes and heteroarenes.^14^ To date, their scope has not been extended beyond (hetero)aromatic substrates. This represents an unprecedented challenge, the success of which would provide access to new or hitherto poorly described molecules due to their cumbersome synthesis.

We herein report a mild metal-free visible-light-driven strategy for tackling this unsolved synthetic issue. We identified enol carbonates as substrates for their peculiar stereoelectronic properties, their ease of preparation, and the molecular diversity they offer in light of the trifluoromethoxylation of structurally diverse ketones (Figure 1d).^15^

Our optimization began by studying the reaction between enol acetate **1a** (10 equiv) and *N*-trifluoromethoxypyridinium **2a**, commercially available as NTf_2_^–^ salt (Table 1, entry 1). We initially evaluated the possibility of exploiting an electron–donor–acceptor (EDA) complex between the two reagents.^16^ Indeed, by mixing **1a** and **2a**, we observed a clear charge-transfer (CT) band in the absorption spectra. Irradiation of the CT band at 400 nm delivered trifluoromethoxylated target **3** in 17% yield. Quite unexpectedly, product **3** was accompanied by undesired side product **4**, where the OCF_3_ group was introduced onto the aromatic ring.^12^ We reasoned that the low chemoselectivity of the process could be overcome by channeling the process toward a purely photoredox manifold, possibly resulting in a chain-propagation process (*vide infra*). We thus screened various photocatalysts (PCs) characterized by diverse redox and photochemical properties.^17^ Naphthochromenone [NTC1 (Table 1, entry 2)] resulted in only slight improvements (20% yield and 12:1 ratio).^18^ We thus selected a red-shifted light source (456 nm) and evaluated the performance of Mes-Acr^+^, Ru(bpy)_3_^2+^, and 4-CzIPN. The low yield (7%) and chemoselectivity (9:1) obtained with the highly oxidizing Mes-Acr^+^ are attributed to the oxidation of **1a**.^19^ On the contrary, Ru(bpy)_3_^2+^ and 4-CzIPN delivered product **3** in promising yield and selectivity, ≤37% and >20:1, respectively (entries 4 and 5, respectively). Remarkably, when using 4-CzIPN, **4** was not detected. We observed additional improvements by increasing the temperature in a more diluted medium (entries 6 and 7). Finally, replacing the acetyl (Ac) with a *tert*-butyloxycarbonyl (Boc) group led to a 52% yield in only 1 h of reaction time (entry 8). Under these conditions, we were able to halve the substrate loading with minimal yield erosion (entry 9). Further decreasing the amount of **1b** resulted in 30% yield (entry 10). It is worth noting that we were able to recover, after purification, >80% of unreactive starting material **1**. Longer reaction times did not result in any improvements in yield, favoring the previously described degradation pathway of **2a**, herein confirmed by experimental evidence.^12^

**Table 1:**
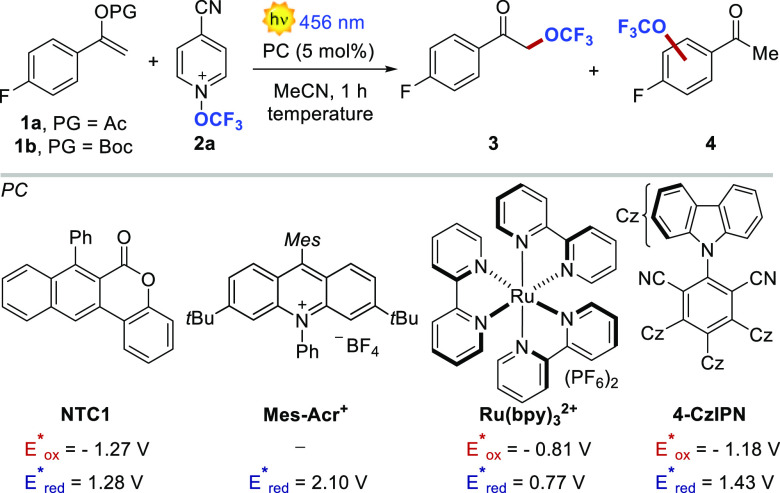
Selected Optimization Results for the Light-Driven α-Trifluoromethoxylation of Ketones^a^

entry	**1** (equiv)	PC	light source (nm)	**3**:**4** ratio	yield of **3** (%)
1	**1a**, 10	–	400	8:1	17
2	**1a**, 10	NTC1	420	13:1	20
3	**1a**, 10	Mes-Acr^+^	456	9:1	7
4	**1a**, 10	Ru(bpy)_3_	456	12:1	33
5	**1a**, 10	4-CzlPN	456	>20:1	37
6^b^	**1a**, 10	4-CzlPN	456	>20:1	41
7^b^,^c^	**1a**, 10	4-CzlPN	420	>20:1	44
8^b^,^c^	**1b**, 10	4-CzlPN	456	>20:1	52
9^b^,^c^	**1b**, 5	4-CzlPN	456	>20:1	50
10^b^,^c^	**1b**, 1.5	4-CzlPN	456	>20:1	30
11^b^,^c^	**1b**, 5	4-CzlPN	456	>20:1	0

a Reaction conditions, unless otherwise stated: 1.5 mL of MeCN, [**2a**]_0_ = 0.033 M, at rt for irradiation for 1 h (see the Supporting Information). ^19^F NMR yield using CF_3_-Ph as an internal standard.

b The reaction was performed at [**2a**]_0_ = 0.01 M.

c Performed at 50 °C.

As expected, the reaction did not proceed in the dark, confirming the light-driven nature of the process (entry 11). Before exploring the generality of the optimized conditions, we decided to decipher the operative mechanism to understand the impact of alternative reaction manifolds on the reaction outcome. As mentioned, the EDA-based pathway resulted in inefficiency and poor chemoselectivity. The poor chemoselectivity was ascribed to the rapid *in situ* deprotection of **1a**, promoted by its single-electron oxidation and the following trifluoromethoxylation of the resulting acetophenone.^12^ This observation was supported by the fact that more diluted conditions disfavor the EDA complex formation supporting a chain-propagation mechanism (Table 1 entry 5 vs entry 6). The reaction catalyzed by Mes-Acr^+^ further corroborated this hypothesis, indicating that the initial oxidation of **1a** is detrimental to the outcome of reaction. Hence, the available concentration of **1a** in the reaction mixture is a key parameter to channel the reactivity toward the intended α-trifluoromethoxylation. Interestingly, the identification of enol carbonate **1b** was the key to increase the overall reactivity of the system. We speculated that the use of the Boc group facilitates the formation of the final product by exploiting the driving force for CO_2_ and isobutylene formation. The higher reactivity observed under the optimized reaction conditions, together with the Stern–Volmer analysis (Figure 2b), led us to depict the mechanistic scenario shown in Figure 2c. Upon excitation, the PC reaches an electronically excited state that reduces **2** by SET, with the generation of the OCF_3_ radical, and the formation of the PC^•+^ radical cation. The OCF_3_ radical is readily intercepted by **1**, with the formation of the C–O bond within **5**. At this juncture, **5** can reduce a second molecule of **2**, in a radical chain process that delivers carbocation **6**. This rapidly evolves to final product **3** with the formation of CO_2_ and isobutylene. Finally, the chain process is terminated by the oxidation of **5** by PC^•+^. This mechanistic hypothesis was supported by quantum yield measurements in the presence of different trifluoromethoxylating agents **2a** and **2b**. When using the easily reducible **2a** (*E*_red_ = 0.15 V versus SCE), we measured a quantum yield of 1.47, indicating that a chain propagation is operative.^20^ On the contrary, when using the more electron-rich **2b** (*E*_red_ = −0.70 V), the quantum yield dropped drastically to <0.01, indicating that intermediate **5** cannot reduce this pyridinium reagent and the mechanism switches to a classic photoredox cycle. Additionally, the reaction appeared to be much slower, affording in 1 h product **3** in 12% yield instead of 50%.

**Figure 2:**
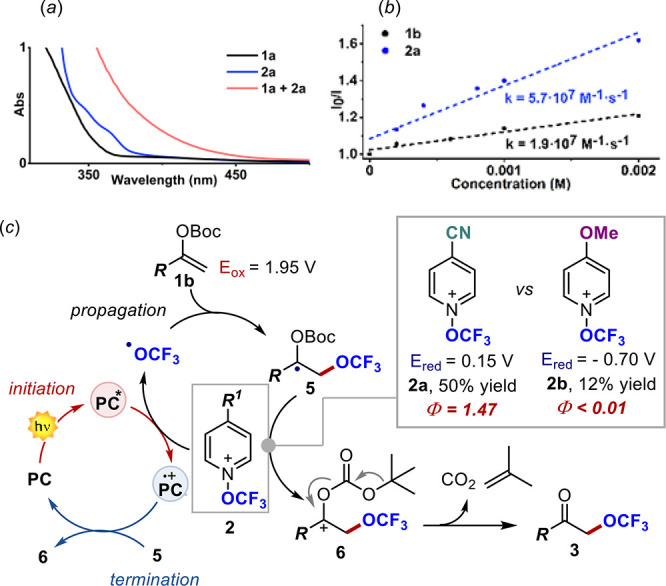
(a) Ultraviolet absorption spectra of **1a**, **2a**, and their mixture. (b) Stern–Volmer quenching experiment of 4-CzIPN with **1b** or **2a**. (c) Proposed reaction manifold.

Having deciphered the operative reaction manifold, we tested the generality of the developed trifluoromethoxylation method. We were pleased to see that substitutions at all of the positions of the aromatic ring were tolerated. Alkyl substituents (**7–9**) gave comparable results with yields of ≤46%. Interestingly, the OCF_3_ group was also readily installed on enol carbonates bearing electron-withdrawing functionalities (CN, Ac, CF_3_, and Br), affording the corresponding products **10–16** in ≤46% yield in 1 h. Remarkably, the reaction was easily transferred into a flow photoreactor without any significant yield erosion (48% for **3**, 50% for **10**, and 41% for **16**), allowing a very short reaction time of only 2 min.

It is worth noting that this mild photoredox-catalyzed protocol is not limited to terminal enol carbonates and that trifluoromethoxylated ketones (Scheme 1, **17** and **18**) bearing a methyl or benzyl group at the α position can also be prepared. We then turned our attention to cyclic ketones. Thus, 1-indanone-derived enol carbonates (**19** and **20**) and benzosuberone (**21**) were also trifluoromethoxylated under our conditions.

**Scheme 1:**
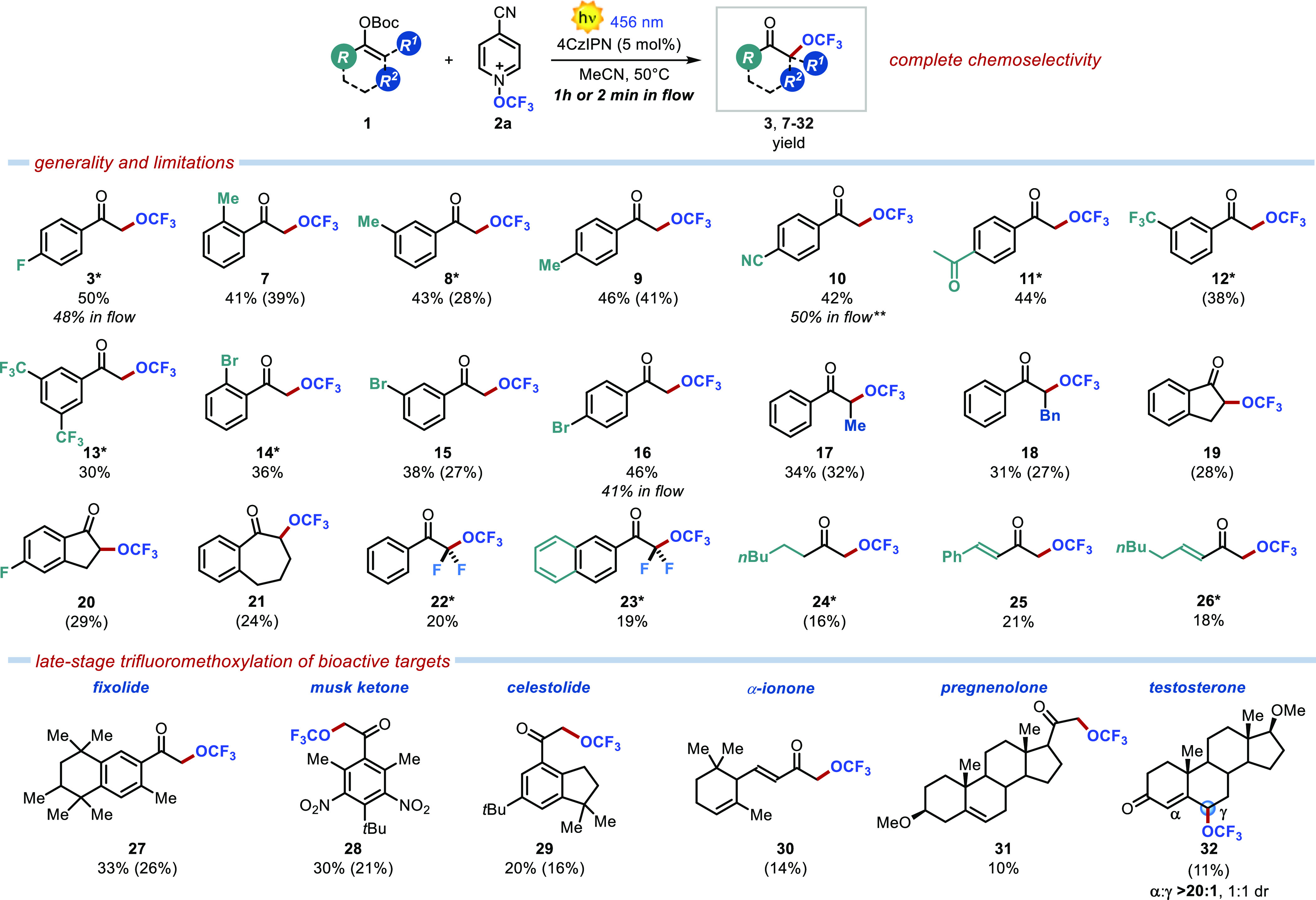
Scope of the Developed Visible-Light-Driven α-Trifluoromethoxylation of Ketones Volatile substrate. Batch reactions were performed in 10 mL of MeCN on a 0.1 mmol scale. Yields determined using the triflimide peak or PhCF_3_ as the internal standard. Isolated yields are reported in parentheses. Isolated yield of the reaction performed with 6 equiv of starting material. A >20:1 chemoselectivity was observed in all cases.

Synthetically appealing difluorinated enol carbonates were also investigated. Remarkably, unprecedented perfluoroalkylated ketones **22** and **23** were easily obtained. We then evaluated the use of challenging enol carbonates derived from aliphatic ketones and enones. We were pleased to see that the intended trifluoromethoxylated ketones (**24–26**) were still successfully produced, in spite of a less important stabilization of the corresponding radical intermediate. It is noteworthy that **25** and **26** were formed as single regioisomers despite the presence of two conjugated double bonds. We next tested the versatility of the developed method for the mild late stage trifluoromethoxylation of biorelevant targets. To our delight, α-OCF_3_ ketones **27–29** derived from fixolide, musk ketone, and celestolide were readily obtained. Remarkably, even the structurally complex bioactive natural products α-ionone and pregnenolone participated in the developed trifluoromethoxylation process, although with inferior results (**30** and **31**). Despite the presence of several double bonds, full chemoselectivity was observed in all of these reactions, while preserving the fragile nature of these complex natural scaffolds. Encouraged by these results, we attempted the installation of the OCF_3_ fragment into the testosterone scaffold. In this case, two conjugated double bonds are present in the starting material, possibly leading to the formation of two diverse regioisomers (α- vs γ-OCF_3_). We were pleased to see that the trifluoromethoxylation occurred selectively at the vinylogous γ-position, furnishing **32** exclusively, in 11% isolated yield.

To further demonstrate the synthetic potential of the developed visible-light-driven method, we performed a large-scale flow synthesis of trifluoromethoxylated ketones **10** and **16** (Scheme 2a).^21^ By applying a flow rate of 5 mL min^–1^ and a residence time as short as 2 min, we were able to scale up the process by 20-fold. A routine reduction with NaBH_4_ furnished the synthetically appealing monotrifluoromethylated vicinal diol **33**. This molecule was subsequently subjected to a Buchwald–Hartwig amination with morpholine, resulting in the formation of derivative **34**. Simple treatment with ammonium acetate under reductive conditions of **16** delivered trifluoromethylated amino alcohol **35**, which is an essential ingredient for the synthesis of allosteric modulators of muscarinic receptors.^22^ Additionally, ketone **10** was reacted with dimethylformamide/dimethylacetal exploiting its pronucleophilic nature giving trifluoromethoxylated enaminone **36**. These experiments demonstrate the synthetic versatility of the products as building blocks. Finally, we tested the robustness of the process in a one-pot two-step sequence starting from acetophenone **37** (Scheme 2b). The crude enol carbonate was directly subjected to the optimized reaction conditions. It is noteworthy that the reaction proceeded with the formation of biorelevant target **38** in 23% yield within an overall reaction time of only 2 h, while previous approaches required 92 h and prefunctionalized substrates.^23^

**Scheme 2:**
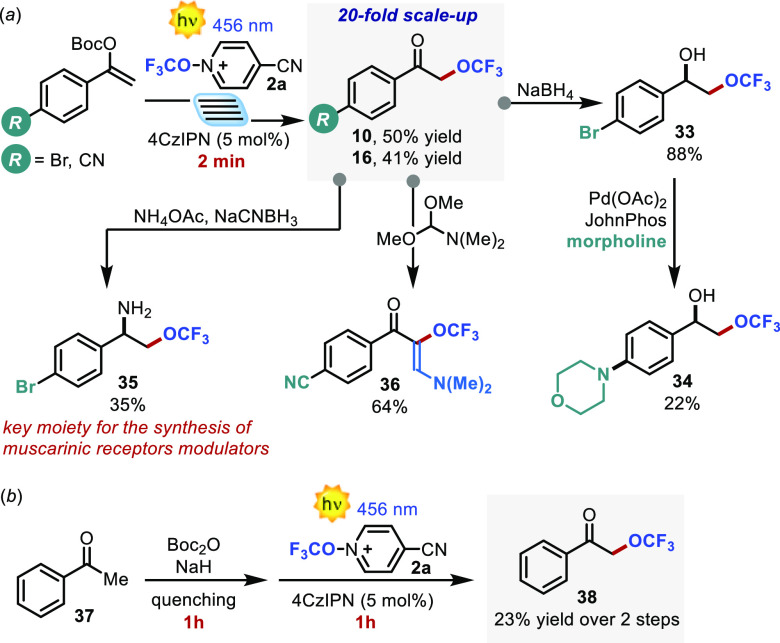
(a) In-Flow Implementation and Product Manipulations and (b) One-Pot Two-Step Sequence to Access Biorelevant Synthetic Targets

In conclusion, a mild and selective visible-light method for the α-trifluoromethoxylation of ketones has been developed. The process uses a commercially available trifluoromethoxylating reagent, an organic photocatalyst, and a wide range of structurally diverse enol carbonates. Mechanistic investigations revealed that a radical chain mechanism is essential for accessing the desired trifluoromethoxylated products in useful synthetic yields. The easy in-flow upscaling and the straightforward manipulations of the products make of this methodology an unprecedented tool for the incorporation of the OCF_3_ fragment into synthetically and biologically relevant targets.

## Supporting Information Available

Experimental procedures and characterization data, cyclic voltammetry, and ultraviolet spectra (PDF)
