# Supplementary material for: Radical α-Trifluoromethoxylation of Ketones under Batch and Flow Conditions by Means of Organic Photoredox Catalysis
Source: Org Lett. 2021 Sep 1;23(18):7088–93. doi: 10.1021/acs.orglett.1c02494 (PMC8453633; doi:10.1021/acs.orglett.1c02494)

Supporting Information

Radical α-Trifluoromethoxylation of Ketones under Batch and Flow Conditions by Means of Organic Photoredox Catalysis

Thibaut Duhail,‡† Tommaso Bortolato,‡§ Javier Mateos,§ Elsa Anselmi, †‖ Benson Jelier,⁋ Antonio Togni,⁋ Emmanuel Magnier, †* Guillaume Dagousset, †* and Luca Dell'Amico§*

†Université Paris-Saclay, UVSQ, CNRS, UMR 8180, Institut Lavoisier de Versailles, 78035 Versailles Cedex (France).

§Department of Chemical Sciences, University of Padova, Via Marzolo 1, 35131, Padova (Italy).

‖Université de Tours, Faculté des Sciences et Techniques, 37200 Tours (France).

⁋ Department of Chemistry and Applied Biosciences, Swiss Federal Institute of Technology, ETH Zurich, Vladimir-Prelog-Weg 2, 8093 Zurich (Switzerland).

‡These authors contributed equally.

Table of Contents

A. GENERAL INFORMATION 1

B. LIGHT SOURCES EMISSION SPECTRA 3

C. EXPERIMENTAL SETUP 4

456 NM REACTION SETUP – ROOM TEMPERATURE 4
456 NM REACTION SETUP – 50°C 4

D. REACTION OPTIMIZATION 5

E. MASS BALANCE INVESTIGATION 7

COF_2 FORMATION AND TRAPPING (SCHEME S1A) 7
COF_2 FORMATION AND TRAPPING IN THE REACTION CONDITIONS (SCHEME S1B) 7

F. SYNTHETIC PROCEDURES AND CHARACTERIZATION 8

GENERAL PROCEDURES FOR THE SYNTHESIS OF STARTING MATERIALS 8
GENERAL PROCEDURES FOR THE TRIFLUOROMETHOXYLATION OF ENOL CARBONATES 17
POORLY REACTIVE SUBSTRATES 26
SCALE-UP EXPERIMENTS 26
POST-MODIFICATIONS 27
ONE-POT PROCEDURE FOR THE TRIFLUOROMETHOXYLATION OF ACETOPHENONE 29

G. MECHANISTIC STUDIES 31

ALPHA-TRIFLUOROMETHOXYLATION OF 1B WITH PYRIDINIUM 2B AND 4CZIPN AS THE PHOTOCATALYST 31
LUMINESCENCE QUENCHING EXPERIMENTS 31
QUANTUM YIELD MEASUREMENTS 32

H. CYCLIC VOLTAMMETRIES 38

I. UV-VIS SPECTRA 39

J. REFERENCES 41

K. NMR SPECTRA 42

A. General Information

All reactions were carried out in anhydrous solvents purchased from commercial suppliers over molecular sieves in a sealed bottle which were used without further purification. Chemicals were purchased from commercial sources (Sigma–Aldrich, Alfa Aesar, Fluorochem, TCI or ABCR) and used without further purification. Organic solvents were purchased from Sigma–Aldrich and Carlo Erba companies. Reactions were monitored by thin-layer chromatography on silica gel 60F254, and/or by ^{19}F NMR spectroscopy.

NMR spectra were collected on a Bruker AC-300 spectrometer fitted with a Bruker PABBO BB/19F-1H/D probe head, Bruker 200 equipped with a QNP probehead, Bruker 400 Avance III HD spectrometer equipped with a BBI-z grad probehead, Bruker 500 Avance III equipped with a BBI-ATM-z grad probehead and Bruker Neo 600 equipped with a Prodigy probehead operating at the denoted spectrometer frequency given in MHz for the specified nucleus. Reported coupling constants and chemicals shifts were based on a first order analysis. The internal reference for ^{1}H NMR was the residual peak of $CDCl_3$ (7.26 ppm), central peak of $CDCl_3$ (77.16 ppm) for ^{13}C NMR spectra, $CFCl_3$ (0.0 ppm) as internal reference for ^{19}F NMR spectra. All coupling constants (J) are reported in Hz with the following abbreviations: s = singlet, d = doublet, dd = double doublet, t = triplet, dt = double triplet, q = quadruplet, m = multiplet, br = broad. High resolution mass spectrometry (HRMS) was recorded on a Mass Spectrometer XEVO-QTOF in the Institute Lavoisier of Versailles – University of Versailles Saint Quentin or on a Xevo G2-XS QToF in the Department of Pharmaceutical Sciences – University of Padua.

Thin-layer chromatography (TLC) analysis was performed on pre-coated Merck TLC plates (silica gel 60G F254, 0.25 mm). Visualization of the developed purification was performed by checking UV absorbance (254 nm) as well as with aqueous ceric ammonium molybdate and potassium permanganate solutions. Chromatographic purification of the products was accomplished using flash chromatography on silica gel (SiO_2, 0.04-0.0063 mm) purchased from Sigma-Aldrich, with the indicated solvent system according to the standard techniques, or with pre-coated Merck preparative TLC plates (silica gel 60G F254, 20x20 cm). Organic solutions were concentrated under reduced pressure on a Büchi rotary evaporator.

Steady-state absorption spectroscopy studies were performed at room temperature on a Varian Cary 50 UV-vis; 10 mm path length Hellma Analytics 100 QS quartz cuvettes were used.

The electrochemical characterizations were carried out in acetonitrile (MeCN)/0.1 M tetrabutylammonium hexafluorophosphate ($TBAPF_6$) at room temperature, on an BASi EC Epsilon potentiostat-galvanostat in a glass cell. All the cyclic voltammograms were recorded with a scan rate of 0.1 V/s. A typical three-electrode cell was employed, which was composed of a glassy carbon (GC) working electrode (3 mm diameter), a platinum wire as counter electrode and a saturated aqueous calomel electrode (SCE) as reference electrode. The glass electrochemical cell was kept closed with a stopper annexed to the potentiostat. Oxygen was removed by purging the solvent with high-purity Nitrogen (N_2), introduced from a line into the cell by means of a glass pipe. The potential of ferrocenium/ferrocene (Fc+/Fc) couple was used as internal reference system to calibrate the potentiostat. All the results are subsequently converted in V vs SCE, in agreement with the value reported in literature [$E_{1/2}$(Fc+/Fc) = +0.395 V vs SCE].[1] The IR compensation implemented within the potentiostat was used, and every effort was made throughout the experiments to minimize the resistance of the solution. The full electrochemical reversibility of the voltammetric wave of ferrocene was taken as an indicator of the absence of uncompensated resistance effects. The GC electrode was polished before any measurement with diamond paste, carefully rinsed with de-ionized water, ethanol, acetone and ultrasonically rinsed with a methanol/ethanol/acetone 1:1:1 (v/v) mixture for 5 minutes. After each series of CV experiments, the electrochemical cell was carefully rinsed with

ethanol, acetone and de-ionized water; afterwards, the cell and the magnetic stirrer were sonicated for 5-10 min with acetone.

456 nm LEDs were purchased on Amazon (https://www.amazon.it/Striscia-Decorazioni-Connettori-Connessione-Alimentatore/dp/B00HSF64E6), as well as the waterproof silicone tubing used for submerging the LEDs under water (https://www.amazon.it/Silicone-Guaine-Flessibile-Bobina-Striscia/dp/B00TOM6NWO/ref=sr_1_4?__mk_it_IT=%C3%85M%C3%85%C5%BD%C3%95%C3%91&dchild=1&keywords=tubo+silicone+led&qid=1622812658&s=lighting&sr=1-4).

Flow reactions were performed on a Vapourtec apparatus equipped with a UV-150 10 mL photochemical reactor.

B. Light sources emission spectra

The following spectrum was recorded by an AvaSpec ULS3648 high-resolution fiber-optic spectrometer which was placed at a fixed distance of 0.5 cm from the light source.

(more info at: https://www.avantes.com/products/spectrometers/starline/item/209-avaspec-uls3648-high-resolution-spectrometer).

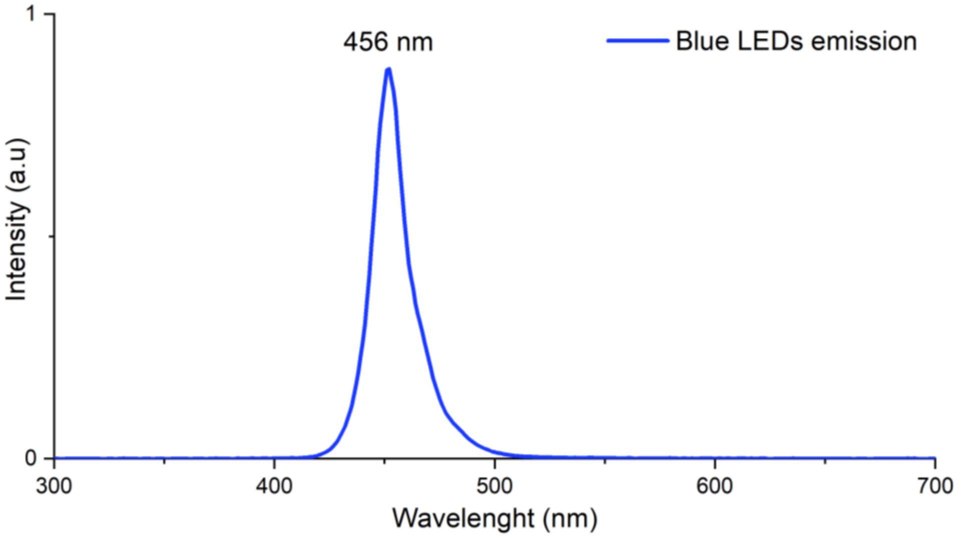

Figure S1 Emission spectrum of the 456 nm LEDs used in this work

C. Experimental setup

456 nm reaction setup – room temperature

Figure S2 shows the general setup of a batch reaction under 456 nm irradiation. The reaction mixture was placed in the middle of the photoreactor (at about 1.5 cm distance from the light source. To maintain a stable reaction temperature one fan was placed close to the vials (25±2 °C) and the temperature was controlled by a thermometer.

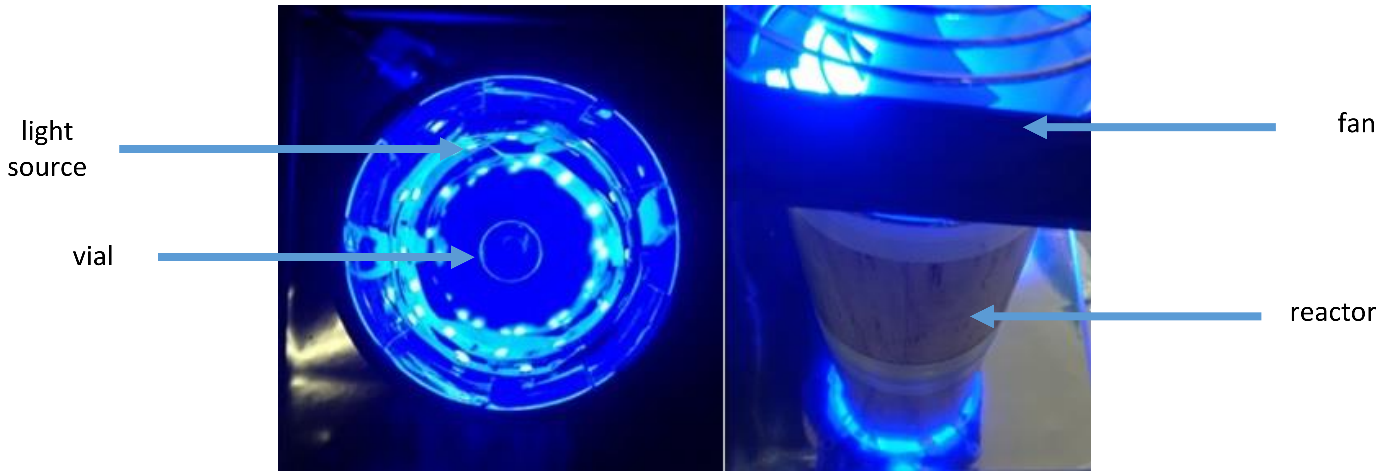

Figure S2 Left: top view of the photoreactor used in this work. Right: Front view of the photoreactor used in this work.

456 nm reaction setup – 50°C

Figure S3 shows the general setup of a batch reaction under 456 nm irradiation at 50°C. The reaction mixture was wrapped by 456 nm LEDs inserted in the silicone tubing to avoid contact with water and placed in a water bath at a stable temperature of 50°C.

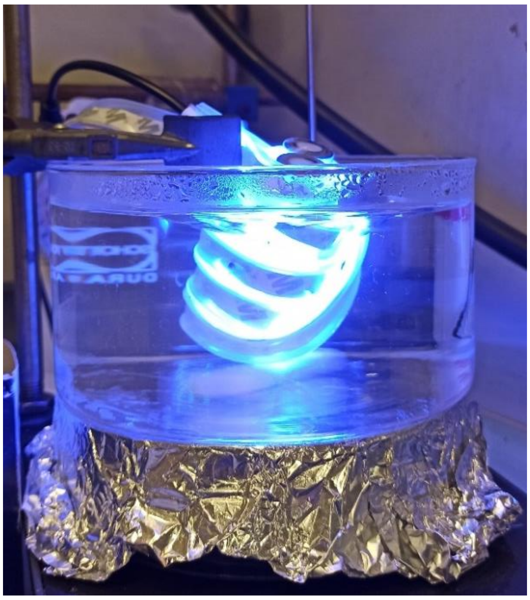

Figure S3 Front view of the reaction setup under 456 nm irradiation at 50°C

D. Reaction optimization

For the trifluoromethoxylation reaction between enol derivatives **1** and the pyridinium reagent **2a** investigated in the present work, different reaction conditions were tested in function of the used enol derivative, reagents ratio, concentration, solvent, photocatalyst, additives, temperature and reaction time (Table S1). Photocatalysts NTC1, 4CzIPN and 3DPAFIPN were synthesised according to reported procedures.[2,3]

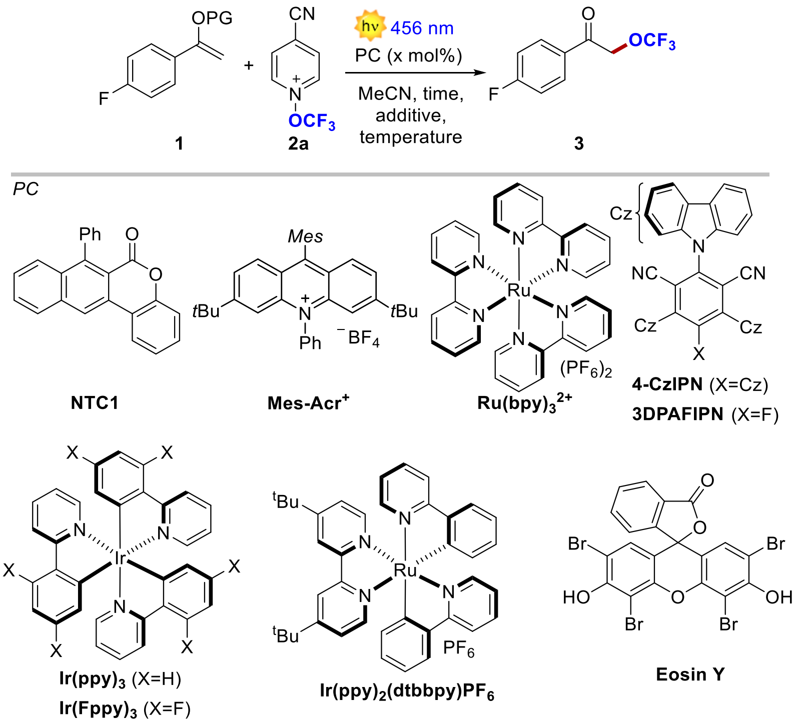

*Table S1 Exploratory studies of the reaction between enol derivative **1** and pyridinium **2a***

Entry	PG	Ratio (1/2a)	Concentration	PC (mol%)	Additive	Yield of 3 (%)a)
**1**	TIPS	1:3	0.1 M	$Ru(bpy)_3(PF_6)_2$ (1)	-	0
**2**	Ac	1:3	0.1 M	$Ru(bpy)_3(PF_6)_2$ (1)	-	8
**3**	Ac	1:1.5	0.1 M	$Ru(bpy)_3(PF_6)_2$ (1)	-	5
**4**	Ac	3:1	0.1 M	$Ru(bpy)_3(PF_6)_2$ (1)	-	6
**7**	Ac	1:3	0.1 M	$Ru(bpy)_3(PF_6)_2$ (1)	K_3PO_4 (2 eq)	0
**8**	Ac	1:3	0.1 M	$Ru(bpy)_3(PF_6)_2$ (1)	K_2CO_3 (2 eq)	0
**9**	Ac	1:3	0.1 M	$Ru(bpy)_3(PF_6)_2$ (1)	K_2HPO_4 (2 eq)	17
**10**	Ac	10:1	0.1 M	$Ru(bpy)_3(PF_6)_2$ (5)	K_2HPO_4 (2 eq)	23
**11**	Ac	10:1	0.1 M	$Ru(bpy)_3(PF_6)_2$ (5)	-	30
**12**	Ac	10:1	0.033 M	$Ru(bpy)_3(PF_6)_2$ (5)	-	33
**13b)**	Ac	10:1	0.033 M	$Ru(bpy)_3(PF_6)_2$ (5)	-	28

Entry	PG	Ratio (1/2a)	Concentration	PC (mol%)	Additive	Yield of 3 (%)[a)]
**14**[c)]	Ac	10:1	0.033 M	$Ru(bpy)_3(PF_6)_2$ (5)	-	30
**15**[d)]	Ac	10:1	0.033 M	$Ru(bpy)_3(PF_6)_2$ (5)	-	26
**16**[e)]	Ac	10:1	0.033 M	$Ru(bpy)_3(PF_6)_2$ (5)	-	25
**17**[f)]	Ac	10:1	0.033 M	-	-	17
**18**	Ac	10:1	0.033 M	$Ir(ppy)_3$ (5)	-	0
**19**	Ac	10:1	0.033 M	$Ir(Fppy)_3$ (5)	-	15
**20**	Ac	10:1	0.033 M	$Ir(ppy)_2(dtbbpy)PF_6$ (5)	-	3
**21**	Ac	10:1	0.033 M	Eosin Y (5)	-	14
**22**	Ac	10:1	0.033 M	Mes-Acr-BF_4 (5)	-	7
**23**	Ac	10:1	0.033 M	3-DPAFIPN (5)	-	27
**24**[g)]	Ac	10:1	0.033 M	NTC1 (5)	-	20
**25**	Ac	10:1	0.033 M	4CzIPN (5)	-	37
**26**	Ac	10:1	0.033 M	4CzIPN (10)	-	30
**27**	Ac	1:3	0.033 M	4CzIPN (5)	-	2
**28**	Ac	10:1	0.01 M	4CzIPN (5)	-	41
**29**	Ac	10:1	0.01 M	4CzIPN (2)	-	33
**30**[h)]	Ac	10:1	0.01 M	4CzIPN (5)	-	44
**31** [h)]	Boc	10:1	0.01 M	4CzIPN (5)	-	52
**32** [h)]	Boc	5:1	0.01 M	4CzIPN (5)	-	50
**33** [h)]	Boc	1.5:1	0.01 M	4CzIPN (5)	-	30
**34** [h)]	Boc	5:1	0.01 M	4CzIPN (5)	BF_3OEt_2 (1 eq)	8
**35** [h)]	Boc	5:1	0.01 M	4CzIPN (5)	Bu_4NOAc (1 eq)	0
**36**[h,i)]	Boc	5:1	0.01 M	4CzIPN (5)	-	0

a) ^{19}F-NMR yield using $PhCF_3$ or the peak of triflimide as the internal standard b) The reaction was performed with a total reaction time of 6h c) The reaction was performed in acetone as solvent d) The reaction was performed in ethyl acetate as solvent e) **2a** was added with a driver syringe at a flow of 0.2 mL per hour in 0.6 mL of acetonitrile f) The reaction was performed under 400 nm light irradiation g) The reaction was performed under 420 nm light irradiation h) The reaction was performed at 50°C i) The reaction was performed in the dark

E. Mass balance investigation

The mass balance of the optimized reaction was investigated.

COF_2 formation and trapping (Scheme S1a)

The in-situ trapping of COF_2 was attempted based on the recent report from the group of Prof. König.[4] A gas-tight NMR tube equipped with a septum containing 600 µL of a 0.01 M solution of **2a** with 1.1 equiv. of *N*-benzylethanolamine **39**, 3 equiv. of DIPEA and 5 mol% 4CzIPN in deuterated acetonitrile was irradiated with the photoreactor (Section C, Figure S2) for 1h. ^{19}F NMR analysis of the crude mixture was readily performed and carbamoyl fluoride **40** was detected in 98% yield. Full consumption of **2a** was observed.

COF_2 formation and trapping in the reaction conditions (Scheme S1b)

A gas-tight NMR tube equipped with a septum containing 600 µL of the reaction mixture in deuterated acetonitrile in the optimized conditions (Table S1, Entry 32) with 1.1 equiv. of *N*-benzylethanolamine **39** was irradiated with the photoreactor (Section C, Figure S2) for 1h. Immediate ^{19}F NMR analysis of the crude mixture was performed and carbamoyl fluoride **40** was detected in 24% yield. Full consumption of **2a** was observed. Product formation was however suppressed and **3** was not detected in the crude reaction mixture.

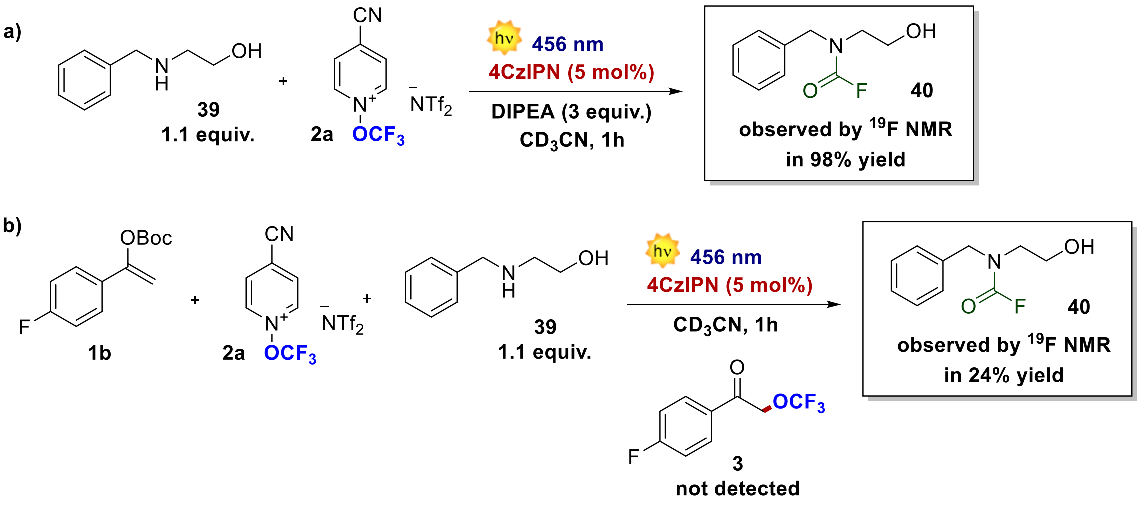

*Scheme S1 a) COF_2 formation from **2a** and trapping with N-benzylethanolamine **39.** b) COF_2 formation from **2a** in the optimized reaction conditions and trapping with N-benzylethanolamine **39.***

These experiments corroborate the known degradation pathway of trifluoromethoxylating reagent **2a** to the corresponding pyridine radical cation and fluorophosgene (see reference 12 in the main text), also under the developed reaction conditions.

F. Synthetic procedures and characterization

General procedures for the synthesis of starting materials

General procedure A, for enol carbonates synthesis

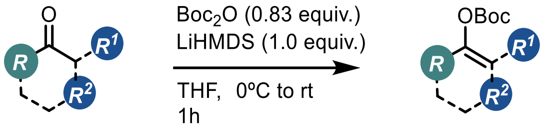

A round-bottomed flask was charged with the required ketone (3.2 mmol, 1.2 equiv.) and suspended in THF (5 mL, 0.6M) at 0°C under argon atmosphere. LiHMDS (1.06 M in THF, 3.0 mL, 1.2 equiv.) was added at once to this suspension, which was then stirred for 5 mins at 0°C. Boc_2O (600 µL, 2.6 mmol, 1 equiv.) was then added and the reaction medium was stirred for 1h at room temperature. The resulting mixture was then quenched with water (20 mL) and extracted with CH_2Cl_2 (3x20mL). The organic phases were then dried over $MgSO_4$ and concentrated under vacuum. Corresponding enol carbonates were isolated by column chromatography on silica gel (Pentane/EtOAc 95/5).

General procedure B, for enol carbonates synthesis

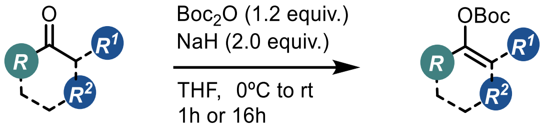

This procedure is the modification of a literature one.[5] A round-bottomed flask was charged with the required ketone (5 mmol, 1 equiv.) and dissolved in dry THF (5 mL, 1M) at 0°C under nitrogen atmosphere. NaH (60% dispersion in mineral oil, 400 mg, 10 mmol, 2 equiv.) was added portionwise to the reaction mixture, which was then stirred for 15 mins at 0°C. Boc_2O (1.38 mL, 6 mmol, 1.2 equiv.) was then added dropwise and the reaction mixture was stirred for 16h at room temperature. The resulting mixture was then quenched with water (20 mL) and DMAP (61 mg, 0.5 mmol, 0.1 equiv.) was added and the reaction mixture stirred for 2 hours. The crude was then diluted with CH_2Cl_2 (50 mL) and washed with brine (3x20 mL). The organic phase was then dried over $MgSO_4$ and concentrated under vacuum. Corresponding enol carbonates were isolated by column chromatography on silica gel with the indicated eluent system.

General procedure C, for enol carbonates synthesis

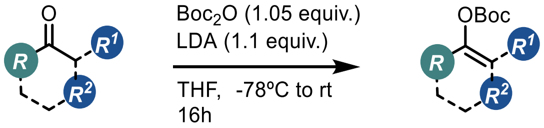

A flame-dried, nitrogen flushed round-bottomed flask was charged with the required ketone (5 mmol, 1 equiv.) and dissolved in dry THF (5 mL, 1M) under nitrogen atmosphere. A freshly prepared solution of LDA (0.5 M in THF, 11 mL, 5.5 mmol, 1.1 equiv.) was dropwise at -78°C to the reaction mixture, which was then stirred for 90 minutes at -78°C. Boc_2O (1.21 mL, 5.25 mmol, 1.05 equiv.) was then added dropwise and the reaction medium was stirred for 30 minutes at -78°C and at room temperature for 16 hours. The resulting mixture was then quenched with water (20 mL), DMAP (61 mg, 0.5 mmol, 0.1 equiv.) was added and the resulting mixture stirred for 2 hours. The crude was then diluted with CH_2Cl_2 (50mL) and washed with brine (3x20 mL). The organic phase was then dried over $MgSO_4$ and

concentrated under vacuum. Corresponding enol carbonates were isolated by column chromatography on silica gel with the indicated eluent system.

1-(4-fluorophenyl)ethenyl acetate 1a

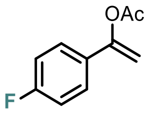

1a

1-(4-fluorophenyl)ethenyl acetate **1a** was synthesized according to a procedure reported in the literature.[6] The spectral data matched with those reported in the literature.

tert-butyl (1-(4-fluorophenyl)vinyl) carbonate 1b

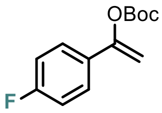

1b

According to general procedure B, 1-(4-fluorophenyl)vinyl tert-butyl carbonate **1b** was obtained from 1-(4-fluorophenyl)ethan-1-one as a yellow oil in 41% yield (494 mg, 2.07 mmol) after purification by column chromatography on silica gel (hexane/EtOAc 95/5). **^{1}H NMR (500 MHz, $CDCl_3$)** δ 7.50 – 7.44 (m, 2H), 7.06 – 7.00 (m, 2H), 5.32 (d, *J* = 2.4 Hz, 1H), 5.07 (d, *J* = 2.4 Hz, 1H), 1.49 (s, 9H) ppm. **^{13}C NMR (126 MHz, $CDCl_3$)** δ 163.2 (d, *J* = 248.7 Hz, **C**-F), 152.5, 151.4, 130.8 (d, *J* = 3.4 Hz), 126.9 (d, *J* = 8.2 Hz), 115.6 (d, *J* = 21.9 Hz), 101.4 (d, *J* = 1.7 Hz, C=**C**H_2), 83.4 (O-**C**-$(CH_3)_3$), 27.6 (C-(**C**$H_3)_3$) ppm. **^{19}F NMR (188 MHz, ^{1}H decoupled, $CDCl_3$)** δ -112.84 ppm. **HRMS (ESI+)** Calcd. for $C_8H_6F^+$ [M - OBoc]$^+$: 121.0448. Found: 121.0449.

tert-butyl (1-phenylvinyl) carbonate 1c

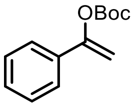

1c

According to general procedure B, tert-butyl (1-phenylvinyl) carbonate **1c** was obtained from acetophenone as a yellow oil in 21% yield (233 mg, 1.06 mmol) after purification by column chromatography on silica gel (hexane/DCM 75/25). The spectral data matched with those reported in the literature.[5]

tert-butyl (1-(o-tolyl)vinyl) carbonate 1d

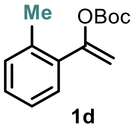

1d

According to general procedure A, tert-butyl (1-(o-tolyl)vinyl) carbonate **1d** was obtained from 2'-methylacetophenone as a yellow oil in 96% yield (600 mg, 2.5 mmol**). ^{1}H NMR (300 MHz, $CDCl_3$)** δ 7.40 (d, *J* = 7.0 Hz, 1H), 7.29 – 7.14 (m, 3H), 5.25 (s, 1H), 4.97 (s, 1H), 2.44 (s, 3H), 1.44 (s, 9H) ppm. **^{13}C NMR (75 MHz, $CDCl_3$)** δ 154.1, 151.1, 136.0, 135.2, 130.6, 129.0, 128.8, 125.7, 104.9 (s, C=**C**H_2), 83.1 (s, 1C, O-**C**-$(CH_3)_3$), 27.7 (s, 3C, C-(**C**$H_3)_3$), 20.5 ppm. **HRMS (ASAP +)** Calcd for $C_{14}H_{18}O_3Na^+$ [M + Na]$^+$: 257.1148. Found: 257.1156.

tert-butyl (1-(m-tolyl)vinyl) carbonate 1e

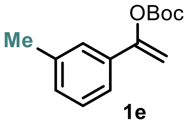

1e

According to general procedure A, tert-butyl (1-(m-tolyl)vinyl) carbonate **1e** was obtained from 3'-methylacetophenone as a yellow oil in 90% yield (550 mg, 2.3 mmol). **^{1}H NMR (300 MHz, $CDCl_3$)** δ 7.35 (d, *J* = 7.6 Hz, 2H), 7.28 (t, *J* = 7.6 Hz, 1H), 7.18 (d, *J* = 7.5 Hz, 1H), 5.43 (d, *J* = 2.2 Hz, 1H), 5.12 (d, *J* = 2.3 Hz, 1H), 2.40 (s, 3H), 1.55 (s, 9H) ppm. **^{13}C NMR (75 MHz, $CDCl_3$)** δ 153.6, 151.5, 138.2, 134.4, 129.8, 128.5, 125.6, 122.0, 101.4 (s, C=**C**H_2), 83.2 (s, 1C, O-**C**-$(CH_3)_3$), 27.7 (s, 3C, C-(**C**$H_3)_3$), 21.5 ppm. **HRMS (ASAP +)** Calcd for $C_{14}H_{18}O_3Na^+$ $[M + Na]^+$: 257.1148. Found: 257.1154.

tert-butyl (1-(p-tolyl)vinyl) carbonate 1f

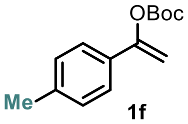

1f

According to general procedure A, tert-butyl (1-(p-tolyl)vinyl) carbonate **1f** was obtained from 4'-methylacetophenone as a yellow oil in 90% yield (550 mg, 2.3 mmol). **^{1}H NMR (300 MHz, $CDCl_3$)** δ 7.40 (d, *J* = 8.5 Hz, 2H), 7.17 (d, *J* = 8.1 Hz, 2H), 5.36 (d, *J* = 2.4 Hz, 1H), 5.04 (d, *J* = 2.5 Hz, 1H), 2.35 (s, 3H), 1.50 (s, 9H) ppm. **^{13}C NMR (75 MHz, $CDCl_3$)** δ 153.5, 151.6, 139.1, 131.7, 129.4, 124.9, 100.9 (s, C=**C**H_2), 83.3 (s, 1C, O-**C**-$(CH_3)_3$), 27.8 (s, 3C, C-(**C**$H_3)_3$), 21.4 ppm. **HRMS (ASAP +)** Calcd for $C_{14}H_{18}O_3Na^+$ [M + Na]$^+$: 257.1148. Found: 257.1153.

tert-butyl (1-(4-cyanophenyl)vinyl) carbonate 1g

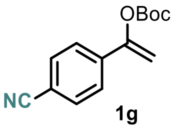

1g

A round-bottomed flask was charged with 4-acetylbenzonitrile (23 mmol, 1.2 equiv.) and suspended in THF (50 mL, 0.5M) at 0°C under argon atmosphere. LiHMDS (1.06 M in THF, 21.7 mL, 1.2 equiv.) was added at once to this suspension, which was then stirred for 5 mins at 0°C. Boc_2O (4.4 ml, 19 mmol, 1 equiv.) was then added and the reaction medium was stirred for 1h at room temperature. The resulting mixture was then quenched with water (50 mL) and extracted with CH_2Cl_2 (3x100mL). The organic phases were then dried over $MgSO_4$ and concentrated under vacuum. Tert-butyl (1-(4-cyanophenyl)vinyl) carbonate **1g** was isolated by column chromatography on silica gel (Pentane/EtOAc 95/5) as a white solid in 87% yield (4.1 g, 16.7 mmol). **^{1}H NMR (300 MHz, $CDCl_3$)** δ 7.66 (d, *J* = 8.1 Hz, 2H), 7.59 (d, *J* = 8.3 Hz, 2H), 5.53 (s, 1H), 5.28 (s, 1H), 1.50 (s, 9H) ppm. **^{13}C NMR (75 MHz, $CDCl_3$)** δ 151.8, 151.2, 139.0, 132.6, 125.6, 118.6, 112.6, 105.0 (C=**C**H_2), 84.2 (O-**C**-$(CH_3)_3$), 27.8 (C-(**C**$H_3)_3$) ppm. **MP** 59-61°C. **HRMS (ASAP +)** Calcd for $C_{14}H_{16}NO_3^+$ [M + H]$^+$: 246.1125. Found: 246.1119.

1-(4-acetylphenyl)vinyl tert-butyl carbonate 1h

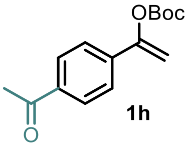

1h

According to general procedure C, 1-(4-acetylphenyl)vinyl tert-butyl carbonate **1h** was obtained from 1,4-diacetylbenzene as a colourless oil in 26% yield (344 mg, 1.3 mmol) after purification by column chromatography on silica gel (hexane/EtOAc 8/2). **^{1}H NMR (400 MHz, $CDCl_3$)** δ 7.98 – 7.90 (m, 2H), 7.61

– 7.53 (m, 2H), 5.52 (d, *J* = 2.5 Hz, 1H), 5.22 (d, *J* = 2.5 Hz, 1H), 2.59 (s, 3H), 1.49 (s, 9H) ppm **^{13}C NMR (101 MHz, $CDCl_3$)** δ 197.4, 152.5, 151.3, 138.9, 137.2, 128.8, 125.1, 104.1 (C=**C**H_2), 83.8 (O-**C**-$(CH_3)_3$), 27.7 (C-(**C**H_3)$_3$), 26.7 ppm. **HRMS (ESI+)** Calcd. for $C_{15}H_{19}O_4H^+$ [M + H]$^+$: 263.1278. Found: 263.1286.

tert-butyl (1-(3-(trifluoromethyl)phenyl)vinyl) carbonate 1i

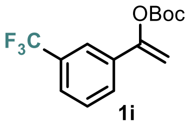

1i

According to general procedure A, tert-butyl (1-(3-(trifluoromethyl)phenyl)vinyl) carbonate **1i** was obtained from 1-(3-(trifluoromethyl)phenyl)ethanone as a yellow oil in 96% yield (725 mg, 2.5 mmol). **^{1}H NMR (300 MHz, $CDCl_3$)** δ 7.74 (s, 1H), 7.67 (d, *J* = 8.0 Hz, 1H), 7.57 (d, *J* = 7.9 Hz, 1H), 7.45 (t, *J* = 7.8 Hz, 1H), 5.47 (s, 1H), 5.20 (s, 1H), 1.49 (s, 5H) ppm. **^{13}C NMR (75 MHz, $CDCl_3$)** δ 152.1, 151.3, 135.5, 131.2 (q, *J* = 32.5 Hz), 129.2, 128.2, 125.6 (q, *J* = 3.8 Hz), 124.0 (q, *J* = 272.3 Hz, CF_3), 121.7 (q, *J* = 3.9 Hz), 103.1 (s, C=**C**H_2), 83.8 (s, 1C, O-**C**-$(CH_3)_3$), 27.5 (s, 3C, C-(**C**H_3)$_3$) ppm. **^{19}F NMR (188 MHz, $CDCl_3$)** δ -63.4 ppm. **HRMS (ASAP +)** Calcd for $C_{14}H_{16}F_3O_3^+$ [M + H]$^+$: 289.1046. Found: 289.1060.

1-(3,5-bis(trifluoromethyl)phenyl)vinyl tert-butyl carbonate 1j

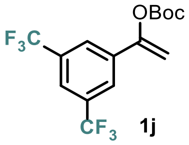

1j

According to general procedure B, 1-(3,5-bis(trifluoromethyl)phenyl)vinyl tert-butyl carbonate **1j** was obtained from 1-(3,5-bis(trifluoromethyl)phenyl)ethan-1-one as a brown solid in 14% yield (241 mg, 0.68 mmol) after purification by column chromatography on silica gel (hexane/DCM 9/1). **^{1}H NMR (400 MHz, $CDCl_3$)** δ 7.92 (s, 2H), 7.84 (s, 1H), 5.58 (d, *J* = 2.9 Hz, 1H), 5.34 (d, *J* = 2.9 Hz, 1H), 1.51 (s, 9H) ppm. **^{13}C NMR (101 MHz, $CDCl_3$)** δ 151.1, 150.8, 136.9, 132.3 (q, *J* = 33.5 Hz, **C**-CF_3), 125.1 (d, *J* = 2.7 Hz), 123.2 (q, *J* = 272.9 Hz, C-**C**F_3), 122.6 (p, *J* = 7.1, 3.7, 3.4 Hz), 104.9 (C=**C**H_2), 84.5 (O-**C**-$(CH_3)_3$), 27.7 (C-(**C**H_3)$_3$) ppm. **^{19}F NMR (188 MHz, ^{1}H decoupled, $CDCl_3$)** δ -63.6 ppm. **HRMS (ASAP +)** Calcd for $C_{10}H_7F_6O^+$ [M - Boc + H]$^+$: 257.0396. Found: 257.0390.

1-(2-bromophenyl)vinyl tert-butyl carbonate 1k

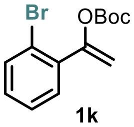

1k

According to general procedure B, 1-(2-bromophenyl)vinyl tert-butyl carbonate **1k** was obtained from 1-(2-bromophenyl)ethan-1-one as a yellow oil in 34% yield (508 mg, 1.7 mmol) after purification by column chromatography on silica gel (hexane/EtOAc 95/5). **^{1}H NMR (500 MHz, $CDCl_3$)** δ 7.57 (dd, *J* = 8.0, 1.2 Hz, 1H), 7.44 (dd, *J* = 7.7, 1.7 Hz, 1H), 7.29 (td, *J* = 7.5, 1.2 Hz, 1H), 7.17 (ddd, *J* = 8.0, 7.4, 1.7 Hz, 1H), 5.32 (d, *J* = 2.0 Hz, 1H), 5.16 (d, *J* = 2.0 Hz, 1H), 1.44 (s, 9H) ppm. **^{13}C NMR (126 MHz, $CDCl_3$)** δ 152.4, 150.8, 136.4, 133.4, 130.8, 130.1, 127.3, 121.5, 106.3 (C=**C**H_2), 83.3 (O-**C**-$(CH_3)_3$), 27.7 (C-(**C**H_3)$_3$) ppm. **HRMS (ESI+)** Calcd. for $C_8H_6Br^+$ [M - OBoc]$^+$: 180.9647. Found: 180.9623.

1-(3-bromophenyl)vinyl tert-butyl carbonate 1l

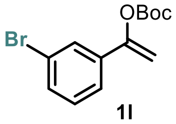

1l

According to general procedure B, 1-(2-bromophenyl)vinyl tert-butyl carbonate **1l** was obtained from 1-(3-bromophenyl)ethan-1-one as a yellow oil in 67% yield (772 mg, 3.35 mmol) after purification by column chromatography on silica gel (hexane/EtOAc 95/5). **^{1}H NMR (500 MHz, CDCl$_3$)** δ 7.64 (t, *J* = 1.9 Hz, 1H), 7.45 (ddd, *J* = 7.9, 2.0, 1.0 Hz, 1H), 7.42 (ddd, *J* = 7.9, 1.8, 1.0 Hz, 1H), 7.21 (t, *J* = 7.9 Hz, 1H), 5.41 (d, *J* = 2.5 Hz, 1H), 5.14 (d, *J* = 2.5 Hz, 1H), 1.50 (s, 9H) ppm. **^{13}C NMR (126 MHz, CDCl$_3$)** δ 152.0, 151.3, 136.6, 132.0, 130.1, 128.1, 123.5, 122.8, 102.9 (C=**C**H$_2$), 83.7 (O-**C**-(CH$_3$)$_3$), 27.7 (C-(**C**H$_3$)$_3$) ppm. **HRMS (ASAP +)** Calcd for $C_8H_8BrO^+$ [M - Boc + H]$^+$: 198.9753. Found: 198.9759.

1-(4-bromophenyl)vinyl tert-butyl carbonate 1m

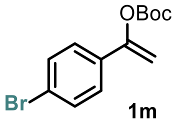

1m

According to general procedure B, 1-(4-bromophenyl)vinyl tert-butyl carbonate **1m** was obtained from 1-(4-bromophenyl)ethan-1-one as a yellow solid in 39% yield (586 mg, 1.95 mmol) after purification by column chromatography on silica gel (hexane/EtOAc 95/5). **^{1}H NMR (500 MHz, CDCl$_3$)** δ 7.48 (d, *J* = 8.7 Hz, 2H), 7.36 (d, *J* = 8.7 Hz, 2H), 5.40 (d, *J* = 2.4 Hz, 1H), 5.12 (d, *J* = 2.4 Hz, 1H), 1.50 (s, 9H) ppm. **^{13}C NMR (126 MHz, CDCl$_3$)** δ 152.5, 151.4, 133.6, 131.8, 126.6, 123.2, 102.4 (C=**C**H$_2$), 83.7 (O-**C**-(CH$_3$)$_3$), 27.8 (C-(**C**H$_3$)$_3$) ppm. **HRMS (ESI+)** Calcd. for $C_{13}H_{15}BrO_3Na^+$ [M + Na]$^+$: 321.0102. Found: 321.0110.

tert-butyl (1-phenylprop-1-en-1-yl) carbonate 1n

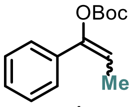

1n

According to general procedure B, tert-butyl (1-phenylprop-1-en-1-yl) carbonate **1n** was obtained from propiophenone as a colourless oil in 24% yield (276 mg, 1.2 mmol) after purification by column chromatography on silica gel (hexane/DCM 7/3). **^{1}H NMR (500 MHz, CDCl$_3$)** δ 7.37 – 7.32 (m, 1H), 7.27 – 7.21 (m, 2H), 7.20 – 7.15 (m, 1H), 5.75 (q, *J* = 7.0 Hz, 1H), 1.69 (d, *J* = 7.0 Hz, 3H), 1.42 (s, 9H) ppm. **^{13}C NMR (126 MHz, CDCl$_3$)** δ 151.2, 147.4, 135.4, 128.6, 128.1, 124.4, 112.7 (C=**C**HR), 83.1 (O-**C**-(CH$_3$)$_3$), 27.8 (C-(**C**H$_3$)$_3$), 11.4 ppm. **HRMS (ESI+)** Calcd. for $C_9H_9^+$ [M - OBoc]$^+$: 117.0699. Found: 117.0701.

tert-butyl (1,3-diphenylprop-1-en-1-yl) carbonate 1o

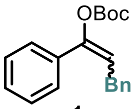

1o

According to general procedure C, tert-butyl (1,3-diphenylprop-1-en-1-yl) carbonate **1o** was obtained from dihydrochalcone as a white solid in 79% yield (1.23 g, 3.9 mmol) after purification by column chromatography on silica gel (hexane/DCM 7/3). **^{1}H NMR (500 MHz, CDCl$_3$)** δ 7.41 – 7.35 (m, 2H), 7.28 – 7.15 (m, 7H), 7.16 – 7.09 (m, 1H), 5.82 (t, *J* = 7.5 Hz, 1H), 3.48 (d, *J* = 7.5 Hz, 2H), 1.41 (s, 9H) ppm. **^{13}C NMR (126 MHz, CDCl$_3$)** δ 151.3, 147.0, 139.8, 135.2, 128.7, 128.6, 128.6, 128.4, 126.4, 124.6, 116.8 (C=**C**HR), 83.4 (O-**C**-(CH$_3$)$_3$), 32.3, 27.7 (C-(**C**H$_3$)$_3$) ppm. **HRMS (ESI+)** Calcd. for $C_{15}H_{13}^+$ [M - OBoc]$^+$: 193.1012. Found: 193.1002.

tert-butyl 1H-inden-3-yl carbonate 1p

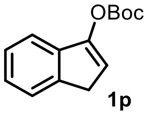

1p

According to general procedure A, tert-butyl 1H-inden-3-yl carbonate **1p** was obtained from 1-Indanone as a yellow oil in 99% yield (600 mg, 2.6 mmol). **^{1}H NMR (300 MHz, CDCl$_3$)** δ 7.45 (dd, *J* = 15.5, 7.3 Hz, 2H), 7.39 – 7.27 (m, 2H), 6.34 (s, 1H), 3.44 (s, 2H), 1.62 (s, 9H) ppm. **^{13}C NMR (75 MHz, CDCl$_3$)** δ 150.9, 149.6, 142.0, 139.0, 126.4, 125.8, 124.2, 118.3, 114.6, 83.7 (s, 1C, O-**C**-(CH$_3$)$_3$), 34.9, 27.8 (s, 3C, C-(**C**H$_3$)$_3$) ppm.

tert-butyl (6-fluoro-1H-inden-3-yl) carbonate 1q

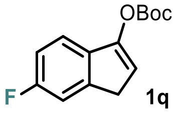

1q

According to general procedure A, tert-butyl (6-fluoro-1H-inden-3-yl) carbonate **1q** was obtained from 5-fluoro-2,3-dihydro-1H-inden-1-one as a yellow oil in 92% yield (600 mg, 2.4 mmol). **^{1}H NMR (300 MHz, CDCl$_3$)** δ 7.34 – 7.25 (m, 3H), 7.15 (d, *J* = 8.8 Hz, 1H), 7.03 (t, *J* = 8.9 Hz, 1H), 6.28 (s, 1H), 3.39 (s, 2H), 1.59 (s, 9H) ppm. **^{13}C NMR (75 MHz, CDCl$_3$)** δ 163.8, 160.5, 150.8, 148.9, 144.0 (d, *J* = 8.8 Hz, 1C), 135.0 (d, *J* = 2.2 Hz, 1C), 119.1 (d, *J* = 8.9 Hz, 1C), 114.0 (d, *J* = 3.8 Hz, 1C), 112.70 (dd, *J* = 121.1, 23.3 Hz), 83.8 (s, 1C, O-**C**-(CH$_3$)$_3$), 34.9 (d, *J* = 2.4 Hz, 1C), 27.8 (s, 3C, C-(**C**H$_3$)$_3$) ppm. **^{19}F NMR (188 MHz, CDCl$_3$)** δ -117.84 (td, *J* = 9.0, 5.0 Hz, 1F) ppm.

tert-butyl (6,7-dihydro-5H-benzo[7]annulen-9-yl) carbonate 1r

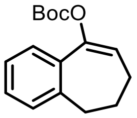

1r

According to general procedure A, tert-butyl (6,7-dihydro-5H-benzo[7]annulen-9-yl) carbonate **1r** was obtained from 1-Benzosuberone as a colorless oil in 92% yield (620 mg, 2.4 mmol). **^{1}H NMR (300 MHz, CDCl$_3$)** δ 7.47 – 7.34 (m, 1H), 7.27 – 7.16 (m, 3H), 5.93 (t, *J* = 6.1 Hz, 1H), 2.83 (t, *J* = 6.0 Hz, 2H), 2.18 (q, *J* = 6.5 Hz, 2H), 2.13 – 7.02 (m, 2H), 1.47 (s, 9H) ppm. **^{13}C NMR (75 MHz, CDCl$_3$)** δ 152.3, 146.2, 141.8, 134.5, 129.2, 128.2, 126.2, 125.3, 119.3, 82.9 (s, 1C, O-**C**-(CH$_3$)$_3$), 33.8, 31.1, 27.8 (s, 3C, C-(**C**H$_3$)$_3$), 25.3 ppm.

tert-butyl 2,2-difluoro-1-phenylvinyl carbonate 1s

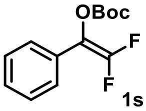

1s

tert-butyl 2,2-difluoro-1-phenylvinyl carbonate **1s** was synthesized according to a procedure reported in the literature.[7] The spectral data matched with those reported in the literature.

tert-butyl 2,2-difluoro-1-(naphthalen-2-yl)vinyl carbonate 1t

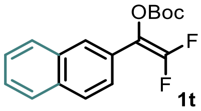

1t

2,2,2-trifluoro-1-(naphthalen-2-yl)ethan-1-one was synthesized according to a reported literature procedure.[8] The spectral data matched with those reported in the literature.

2,2,2-trifluoro-1-(naphthalen-2-yl)ethan-1-one (433 mg, 1.93 mmol, 1 equiv.) was dissolved in MeOH (5 mL, 0.4M) at 0°C. $NaBH_4$ (80 mg, 2.12 mmol, 1.1 equiv.) was then added portionwise and the reaction stirred for 1h at room temperature. The reaction was then cooled to 0°C, quenched with sat. NH_4Cl until gas evolution stopped and evaporated to dryness. The crude residue was diluted with DCM (20 mL) and washed with brine (3x 10 mL). The organic phase was then dried over $MgSO_4$ and concentrated under reduced pressure. The crude 2,2,2-trifluoro-1-(naphthalen-2-yl)ethan-1-ol product was used in the subsequent step without further purification. The spectral data matched with those reported in the literature.[9]

Crude 2,2,2-trifluoro-1-(naphthalen-2-yl)ethan-1-ol (340 mg) was dissolved in MeCN (7 mL) and DMAP (18.3 mg, 0.15 mmol) was added. Boc_2O (517 µL, 2.25 mmol) was then added dropwise and the reaction mixture stirred for 20h, after which time the mixture was concentrated under reduced pressure. The residue was filtered on silica using DCM as eluent and the eluted solution evaporated under reduced pressure. The crude tert-butyl (2,2,2-trifluoro-1-(naphthalen-2-yl)ethyl) carbonate product was used in the subsequent step without further purification.

To a solution of crude tert-butyl (2,2,2-trifluoro-1-(naphthalen-2-yl)ethyl) carbonate (485 mg) in dry THF (7 mL) at −78 °C was added dropwise a freshly prepared solution of LDA (0.7 M, 3.21 mL). After stirring for 1h at −78 °C, the reaction mixture was stirred for an additional 1 h at room temperature. The reaction mixture was then diluted with water (10 mL) and extracted with EtOAc (3 × 10 mL), dried over anhydrous $MgSO_4$, and the solvent evaporated to give a residue. Finally, the residue was purified by silica gel chromatography (hexane/EtOAc 97/3) to give tert-butyl 2,2-difluoro-1-(naphthalen-2-yl)vinyl carbonate **1t** in 45% yield over 3 steps (266 mg, 0.87 mmol). **^{1}H NMR (500 MHz, $CDCl_3$)** δ 7.92 – 7.88 (m, 1H), 7.89 – 7.79 (m, 3H), 7.56 (dt, *J* = 8.7, 1.8 Hz, 1H), 7.54 – 7.47 (m, 2H), 1.53 (s, 9H) ppm. **^{13}C NMR (126 MHz, $CDCl_3$)** δ 155.2 (dd, *J* = 292.4, 290.5 Hz, (C=**C**F_2), 151.0 (dd, *J* = 3.5, 2.3 Hz), 133.2 (d, *J* = 10.5 Hz), 128.6, 128.4, 127.8, 126.9, 126.9, 126.8, 126.8, 125.1 (t, *J* = 5.3 Hz), 122.9 (dd, *J* = 7.0, 2.3 Hz), 113.4 (dd, *J* = 38.8, 19.5 Hz, **C**=CF_2), 84.7 (O-**C**-$(CH_3)_3$), 27.7 (C-$(CH_3)_3$) ppm. **^{19}F NMR (188 MHz, $CDCl_3$)** δ -92.4 (d, *J* = 46.6 Hz), -103.9 (d, *J* = 46.6 Hz) ppm. **HRMS (ASAP +)** Calcd for $C_{12}H_9F_2O^+$ [M - Boc + H]$^+$: 207.0616. Found: 207.0621.

tert-butyl oct-1-en-2-yl carbonate 1u

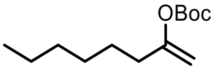

1u

According to general procedure C, tert-butyl oct-1-en-2-yl carbonate **1u** was obtained from octan-2-one as a colourless oil in 33% yield (380 mg, 1.66 mmol) after purification by column chromatography on silica gel (hexane/EtOAc 98/2). **^{1}H NMR (400 MHz, $CDCl_3$)** δ 4.75 – 4.67 (m, 1H), 4.62 – 4.56 (m, 1H), 2.21 – 2.10 (m, 2H), 1.43 (s, 9H), 1.41 – 1.38 (m, 4H), 1.24 – 1.21 (m, 4H), 0.83 – 0.80 (m, 3H) ppm. **^{13}C NMR (101 MHz, $CDCl_3$)** δ 156.6, 151.46, 100.4 (C=**C**H_2), 82.4 (O-**C**-$(CH_3)_3$), 33.1, 31.6, 28.6, 27.6 (C-(**C**H_3)$_3$), 26.4, 22.5, 14.0 ppm. **HRMS (ESI+)** Calcd. for $C_{13}H_{24}O_3Na^+$ [M + Na]$^+$: 251.1618. Found: 251.1596.

(E)-tert-butyl (4-phenylbuta-1,3-dien-2-yl) carbonate 1v

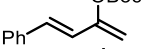

1v

According to general procedure A, (E)-tert-butyl (4-phenylbuta-1,3-dien-2-yl) carbonate **1v** was obtained from 4- (E)-4-phenylbut-3-en-2-one as a colorless oil in 63% yield (400 mg, 1.6 mmol). This product had to be stored at -20°C, decomposition was noticed after few days in the fridge. **^{1}H NMR (300 MHz, $CDCl_3$)** δ 7.44 (d, *J* = 7.5 Hz, 2H), 7.40 – 7.27 (m, 3H), 6.70 (dd, *J* = 16.1, 11.3 Hz, 2H), 5.10 (d, *J* = 8.7 Hz, 2H), 1.58 (s, 9H) ppm. **^{13}C NMR (75 MHz, $CDCl_3$)** δ 152.3, 136.2, 130.1, 128.8, 128.4, 127.0, 122.6, 105.7 (s, C=**C**H_2), 83.4 (s, 1C, O-**C**-$(CH_3)_3$), 27.8 (s, 3C, C-(**C**H_3)$_3$) ppm.

(E)-tert-butyl nona-1,3-dien-2-yl carbonate 1w

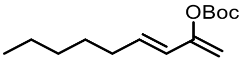

1w

According to general procedure A, (E)-tert-butyl nona-1,3-dien-2-yl carbonate **1w** was obtained from (E)-non-3-en-2-one as a colorless oil in 99% yield (620 mg, 2.6 mmol). **^{1}H NMR (300 MHz, $CDCl_3$)** δ 5.99 – 5.78 (m, 2H), 4.83 (s, 2H), 2.10 (q, *J* = 7.0 Hz, 2H), 1.52 (s, 9H), 1.40 (t, *J* = 7.0 Hz, 2H), 1.29 (s, 4H), 0.92 – 0.82 (t, *J* = 6.7 Hz, 3H) ppm. **^{13}C NMR (75 MHz, $CDCl_3$)** δ 152.2, 151.4, 132.9, 124.0, 102.8 (s, C=**C**H_2), 82.8 (s, 1C, O-**C**-$(CH_3)_3$), 32.2, 31.3, 28.6, 27.6 (s, 3C, C-(**C**$H_3)_3$), 22.5, 14.0 ppm. **HRMS (ASAP +)** Calcd for $C_{14}H_{24}NaO_3^+$ [M + Na]$^+$: 263,1613. Found: 263,1618.

tert-butyl 1-(3,5,5,6,8,8-hexamethyl-5,6,7,8-tetrahydronaphthalen-2-yl)vinyl carbonate 1x

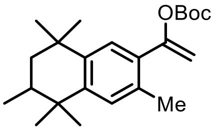

1x

According to general procedure C, tert-butyl 1-(3,5,5,6,8,8-hexamethyl-5,6,7,8-tetrahydronaphthalen-2-yl)vinyl) carbonate **1x** was obtained from 6-Acetyl-1,1,2,4,4,7-hexamethyltetralin as a white solid in 42% yield (752 mg, 2.1 mmol) after purification by column chromatography on silica gel (hexane/DCM 7/3). **^{1}H NMR (500 MHz, $CDCl_3$)** δ 7.29 (s, 1H), 7.15 (s, 1H), 5.21 (d, *J* = 1.6 Hz, 1H), 4.96 (d, *J* = 1.6 Hz, 1H), 2.39 (s, 3H), 1.85 (dqd, *J* = 13.5, 6.8, 2.6 Hz, 1H), 1.61 (t, *J* = 13.2 Hz, 1H), 1.43 (s, 9H) 1.36 (dd, *J* = 13.5, 2.6 Hz, 1H), 1.31 (s, 3H), 1.27 (s, 3H), 1.23 (s, 3H), 1.06 (s, 3H), 0.98 (d, *J* = 6.8 Hz, 3H) ppm. **^{13}C NMR (126 MHz, $CDCl_3$)** δ 153.9, 151.2, 146.8, 142.3, 132.8, 132.4, 129.3, 126.5, 104.3 (C=**C**H_2), 83.0 (O-**C**-$(CH_3)_3$), 43.8, 37.7, 34.7, 34.1, 32.5, 32.1, 28.7, 27.8 (C-(**C**$H_3)_3$), 25.0, 20.5, 17.0 ppm. **HRMS (ESI+)** Calcd. for $C_{18}H_{25}^+$ [M - OBoc]$^+$: 241.1951. Found: 241.1967.

tert-butyl (1-(4-(tert-butyl)-2,6-dimethyl-3,5-dinitrophenyl)vinyl) carbonate 1y

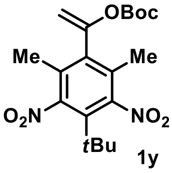

1y

According to general procedure A, tert-butyl (1-(4-(tert-butyl)-2,6-dimethyl-3,5-dinitrophenyl)vinyl) carbonate **1y** was obtained from musk ketone as a colorless oil in 99% yield (620 mg, 2.6 mmol). **^{1}H NMR (300 MHz, $CDCl_3$)** δ 5.56 (s, 1H), 4.94 (s, 1H), 2.28 (s, 6H), 1.48 (s, 9H), 1.44 (s, 9H) ppm. **^{13}C NMR (75 MHz, $CDCl_3$)** δ 150.4, 148.6, 136.8, 131.9, 131.8, 108.2 (s, C=**C**H_2), 84.2 (s, 1C, O-**C**-$(CH_3)_3$), 37.5, 30.5, 27.7 (s, 3C, C-(**C**$H_3)_3$), 16.1 ppm. **HRMS (ASAP +)** Calcd for $C_{19}H_{27}N_2O_7^+$ [M + H]$^+$: 395.1813. Found: 395.1813.

tert-butyl (1-(6-(tert-butyl)-1,1-dimethyl-2,3-dihydro-1H-inden-4-yl)vinyl) carbonate 1z

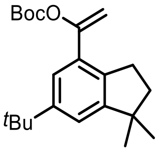

1z

According to general procedure A, tert-butyl (1-(6-(tert-butyl)-1,1-dimethyl-2,3-dihydro-1H-inden-4-yl)vinyl) carbonate **1z** was obtained from celestolide as a white solid in 70% yield (630 mg, 1.8 mmol). **^{1}H NMR (300 MHz, $CDCl_3$)** δ 7.28 (s, 1H), 7.14 (s, 1H), 5.16 (d, *J* = 9.7 Hz, 2H), 2.96 (t, *J* = 7.1 Hz, 2H), 1.93 (t, *J* = 7.2 Hz, 2H), 1.45 (s, 9H), 1.32 (s, 9H), 1.26 (s, 6H) ppm. **^{13}C NMR (75 MHz, $CDCl_3$)** δ 153.9,

153.5, 151.4, 150.0, 137.3, 130.7, 121.5, 119.6, 103.8 (s, C=**C**H_2), 83.1 (s, 1C, O-**C**-(CH_3)$_3$), 43.9, 41.6, 34.8, 31.7, 30.1, 28.8, 27.8 (s, 3C, C-(**C**H_3)$_3$) ppm. **HRMS (ASAP +)** Calcd for $C_{34}H_{50}O_2^+$ [2(M - Boc + H) + H]$^+$: 489.3727. Found: 489.3726.

(E)-tert-butyl (4-(2,6,6-trimethylcyclohex-2-en-1-yl)buta-1,3-dien-2-yl) carbonate 1aa

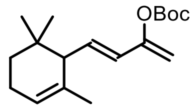

1aa

According to general procedure A, (E)-tert-butyl (4-(2,6,6-trimethylcyclohex-2-en-1-yl)buta-1,3-dien-2-yl) carbonate **1aa** was obtained from α-ionone as an orange oil in 72% yield (550 mg, 1.9 mmol). **^{1}H NMR (300 MHz, $CDCl_3$)** δ 5.91 (d, *J* = 15.6 Hz, 1H), 5.64 (dd, *J* = 15.4, 9.4 Hz, 1H), 5.34 (d, *J* = 36.8 Hz, 1H), 4.84 (s, 2H), 2.16 (d, *J* = 9.5 Hz, 1H), 1.98 (s, 2H), 1.55 (s, 3H), 1.49 (s, 9H), 1.42 – 1.32 (m, 1H), 1.23 – 1.09 (m, 1H), 0.88 (s, 3H), 0.80 (s, 3H) ppm. **^{13}C NMR (75 MHz, $CDCl_3$)** δ 152.2, 151.4, 133.8, 133.6, 125.2, 121.5, 103.3 (s, C=**C**H_2), 82.9 (s, 1C, O-**C**-(CH_3)$_3$), 54.1, 32.6, 31.7, 27.7 (s, 3C, C-(**C**H_3)$_3$), 27.5, 27.1, 23.1, 23.0 ppm. **HRMS (ASAP +)** Calcd for $C_{13}H_{21}O^+$ [M - Boc + H]$^+$: 193.1587. Found: 193.1586.

tert-butyl (1-(-3-methoxy-10,13-dimethyl-tetradecahydro-1H-cyclopenta[a]phenanthren-17-yl)vinyl) carbonate 1ab

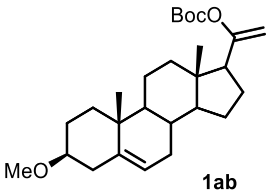

1ab

Pregnenolone methyl ether was synthesised according to a literature procedure.[10] Spectral data matched with those reported in the literature.

According to general procedure C, tert-butyl (1-((3S,10R,13S,17S)-3-methoxy-10,13-dimethyl-tetradecahydro-1H-cyclopenta[a]phenanthren-17-yl)vinyl) carbonate **1ab** was obtained from pregnenolone methyl ether as a white solid in 57% yield (1.23 g, 2.85 mmol) after purification by column chromatography on silica gel (hexane/EtOAc 9/1). **^{1}H NMR (500 MHz, $CDCl_3$)** δ 5.34 (dt, *J* = 5.4, 2.1 Hz, 1H), 4.85 (dd, *J* = 1.9, 1.0 Hz, 1H), 4.74 (t, *J* = 1.7 Hz, 1H), 3.34 (s, 3H), 3.04 (tt, *J* = 11.4, 4.6 Hz, 1H), 2.43 – 2.31 (m, 2H), 2.19 – 2.10 (m, 1H), 2.02 – 1.94 (m, 2H), 1.93 – 1.81 (m, 2H), 1.74 – 1.64 (m, 2H), 1.58 – 1.50 (m, 2H), 1.49 (s, 9H), 1.45 – 1.35 (m, 2H), 1.25 (ddd, *J* = 18.7, 9.0, 4.6 Hz, 2H), 1.21 – 1.14 (m, 2H), 1.14 – 1.02 (m, 2H), 0.99 (s, 3H), 0.65 (s, 3H). **^{13}C NMR (126 MHz, $CDCl_3$)** δ 157.6, 151.8, 141.1, 121.4, 101.8 (C=**C**H_2), 82.6 (O-**C**-(CH_3)$_3$), 80.4, 56.1, 55.7, 53.3, 50.3, 43.1, 38.8, 38.0, 37.3, 37.1, 32.3, 31.9, 28.1, 27.9 (C-(**C**H_3)$_3$), 24.9, 24.3, 21.1, 19.5, 12.7 ppm. **HRMS (ESI+)** Calcd. for $C_{27}H_{42}O_4Na^+$ [M + Na]$^+$: 453.2975. Found: 453.2980.

tert-butyl (-17-methoxy-10,13-dimethyl-dodecahydro-1H-cyclopenta[a]phenanthren-3-yl) carbonate 1ac

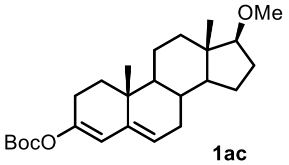

1ac

17β-methoxy-4-androsten-3-one was synthesised according to a literature procedure.[11] Spectral data matched with those reported in the literature.

According to general procedure A, tert-butyl ((10R,13S,17S)-17-methoxy-10,13-dimethyl-dodecahydro-1H-cyclopenta[a]phenanthren-3-yl) carbonate **1ac** was obtained from 17β-methoxy-4-androsten-3-one as a white solid in 76% yield (800 mg, 2.0 mmol). **^{1}H NMR (300 MHz, $CDCl_3$)** δ 5.38 (s, 1H), 5.25 (s, 1H), 3.33 (s, 3H), 3.20 (t, *J* = 8.3, 1H), 2.23 (t, *J* = 7.7 Hz, 4H), 2.05 – 1.85 (m, 2H), 1.74 – 1.55 (m, 2H), 1.49 (d, 9H), 1.46 – 1.35 (m, 4H), 1.33 – 1.07 (m, 3H), 1.00 – 0.90 (m, 5H), 0.77 (s, 3H) ppm. **^{13}C NMR (75 MHz, $CDCl_3$)** δ 152.0, 149.5, 145.1, 115.6, 107.2, 90.8, 82.9 (s, 1C, O-**C**-$(CH_3)_3$), 58.0, 54.7, 50.9, 43.0, 38.1, 38.0, 37.2, 36.2, 31.2, 30.8, 27.9, 27.8 (s, 3C, C-(**C**$H_3)_3$), 23.6, 21.4, 17.5, 11.6 ppm. **HRMS (ASAP +)** Calcd for $C_{25}H_{38}O_4Na^+$ $[M + Na]^+$: 425.2662. Found: 425.2677.

General procedures for the trifluoromethoxylation of enol carbonates

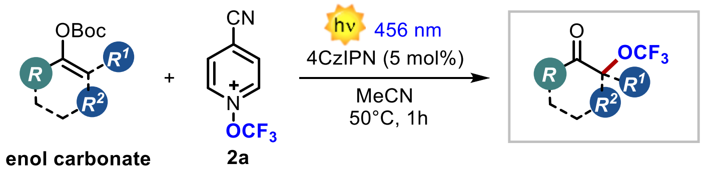

General procedure D, for trifluoromethoxylation of enol carbonates

A sealable tube surrounded by blue LEDs with a watertight protection, purged with argon and equipped with a cap was charged with 4-cyano-1-(trifluoromethoxy)pyridin-1-ium bis((trifluoromethyl)sulfonyl)amide **2a** (46.9 mg, 0.1 mmol, 1 equiv.), the desired enol carbonate (0.5 mmol, 5 equiv.) and 4CzIPN (3.9 mg, 0.005 mmol, 0.05 equiv.). Dry acetonitrile (10 mL, 0.01 M) was finally added and the reaction medium purged with argon again. The resulting mixture in the sealable tube was placed in a water bath and allowed to stir vigorously for 1h at 50°C under irradiation of the blue LEDs. The crude was then concentrated under vacuum. Alpha-trifluoromethoxylated ketones were isolated by preparative thin layer chromatography (PTLC).

General procedure E, for trifluoromethoxylation of enol carbonates

Two 8-mL vials surrounded by blue LEDs with a watertight protection, equipped with a screwcap and a septum were purged with argon and each one charged with 4-cyano-1-(trifluoromethoxy)pyridin-1-ium bis((trifluoromethyl)sulfonyl)amide **2a** (23.5 mg, 0.05 mmol, 1 equiv.), the desired enol carbonate (0.25 mmol, 5 equiv.) and 4CzIPN (1.9 mg, 0.0025 mmol, 0.05 equiv.). Dry acetonitrile (5 mL, 0.01 M) was finally added and the reaction medium purged with argon again. The resulting mixtures in the vials were placed in a water bath and allowed to stir vigorously for 1h at 50°C under irradiation of the blue LEDs. The crude reaction mixture was then transferred to a round-bottom flask, 1 mL of 6M HCl was added and the reaction mixture stirred until complete hydrolysis of the unreacted starting material (controlled by TLC analysis). The crude reaction mixture was then quenched with sat. $NaHCO_3$ until gas evolution stopped, diluted with DCM (30 mL) and washed with brine (3x10 mL). The organic phase was dried over $MgSO_4$ and concentrated under vacuum. Corresponding alpha-trifluoromethoxylated ketones were isolated by column chromatography on silica gel.

1-(4-fluorophenyl)-2-(trifluoromethoxy)ethanone 3

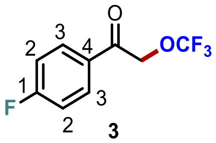

3

According to general procedure D, 1-(4-fluorophenyl)-2-(trifluoromethoxy)ethanone **3** was obtained from the corresponding enol carbonate **1b**; the yield of the desired product was determined by

comparing the integration of the ^{19}F NMR resonance of the product with that of bis((trifluoromethyl)sulfonyl)amide. ^{19}F NMR yield of 50**%.** The product was isolated by PTLC (Pentane/Et_2O (85/15, 2 elutions)) in 32% yield (7.0 mg, 0.031 mmol). **^{1}H NMR (300 MHz, $CDCl_3$)** δ 8.02 – 7.89 (m, 1H), 7.19 (t, *J* = 8.4 Hz, 1H), 5.13 (s, 1H) ppm. **^{13}C NMR (75 MHz, $CDCl_3$)** δ 188.9 (**C**=O), 166.5 (d, *J* = 257.2 Hz, **C**1), 130.9 (d, *J* = 9.5 Hz, **C**2), 130.4 (d, *J* = 3.1 Hz, **C**4), 121.8 (q, *J* = 256.6 Hz, RO**C**F_3), 116.48 (d, *J* = 22.2 Hz, **C**3), 68.3 (q, *J* = 2.9 Hz, R**C**H_2-OCF_3) ppm. **^{19}F NMR (188 MHz, $CDCl_3$)** δ -61.56 (s, 3F), -102.88 (m, 1F) ppm. **HRMS (ASAP +)** Calcd for $C_9H_7F_4O_2^+$ $[M + H]^+$: 223.0377. Found: 223.0386.

1-(*o*-tolyl)-2-(trifluoromethoxy)ethanone 7

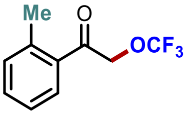

7

According to general procedure D, 1-(*o*-tolyl)-2-(trifluoromethoxy)ethanone **7** was obtained from the corresponding enol carbonate **1d**; the yield of the desired product was determined by comparing the integration of the ^{19}F NMR resonance of the product with that of bis((trifluoromethyl)sulfonyl)amide. ^{19}F NMR yield of 41%. The product was isolated by PTLC (Pentane/Et_2O (95/5, 2 elutions)) in 39% yield (8.5 mg, 0.036 mmol). **^{1}H NMR (300 MHz, $CDCl_3$)** δ 7.54 (d, *J* = 7.7 Hz, 1H), 7.46 (t, *J* = 7.6 Hz, 1H), 7.31 (d, *J* = 8.0 Hz, 2H), 5.04 (s, 2H), 2.54 (s, 3H) ppm. **^{13}C NMR (75 MHz, $CDCl_3$)** δ 193.6, 139.7, 133.8, 132.8, 132.7, 128.3, 126.0, 121.8 (q, *J* = 256 Hz, RO**C**F_3), 69.3 (q, *J* = 2.9 Hz, R**C**H_2-OCF_3), 21.4 ppm. **^{19}F NMR (188 MHz, $CDCl_3$)** δ -61.40 ppm. **HRMS (ASAP +)** Calcd for $C_{10}H_{10}F_3O_2^+$ $[M + H]^+$: 219.0627. Found: 219.0634.

1-(*m*-tolyl)-2-(trifluoromethoxy)ethanone 8

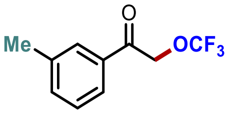

8

According to general procedure D, 1-(*m*-tolyl)-2-(trifluoromethoxy)ethanone **8** was obtained from the corresponding enol carbonate **1e**; the yield of the desired product was determined by comparing the integration of the ^{19}F NMR resonance of the product with that of bis((trifluoromethyl)sulfonyl)amide. ^{19}F NMR yield of 43%. The product was isolated by PTLC (Pentane/Et_2O (95/5, 2 elutions)) in 28% yield (6.1 mg, 0.028 mmol). **^{1}H NMR (300 MHz, $CDCl_3$)** δ 7.74 – 7.65 (m, 2H), 7.49 – 7.34 (m, 2H), 5.17 (s, 2H), 2.43 (s, 3H) ppm. **^{13}C NMR (75 MHz, $CDCl_3$)** δ 190.4, 139.2, 135.2, 133.9, 129.0, 128.5, 125.2, 121.8 (q, *J* = 256.5 Hz, RO**C**F_3), 68.4 (q, *J* = 2.9 Hz, R**C**H_2-OCF_3), 21.5 ppm. **^{19}F NMR (188 MHz, $CDCl_3$)** δ -61.41 ppm. **HRMS (ASAP +)** Calcd for $C_{10}H_{10}F_3O_2^+$ $[M + H]^+$: 219.0627. Found: 219.0625.

1-(*p*-tolyl)-2-(trifluoromethoxy)ethanone 9

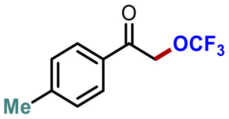

9

According to general procedure D, 1-(*p*-tolyl)-2-(trifluoromethoxy)ethanone **9** was obtained from the corresponding enol carbonate **1f**; the yield of the desired product was determined by comparing the integration of the ^{19}F NMR resonance of the product with that of bis((trifluoromethyl)sulfonyl)amide. ^{19}F NMR yield of 46%. The product was isolated by PTLC (Pentane/Et_2O (95/5, 2 elutions)) in 41% yield (9.0 mg, 0.041 mmol). **^{1}H NMR (300 MHz, $CDCl_3$)** δ 7.80 (d, *J* = 7.8 Hz, 2H), 7.30 (d, *J* = 7.8 Hz, 2H), 5.15 (s, 2H), 2.43 (s, 3H) ppm. **^{13}C NMR (75 MHz, $CDCl_3$)** δ 189.9, 145.6, 131.4, 129.8, 128.2, 121.8 (q, *J* = 257

Hz, RO**C**F_3). 68.4 (q, *J* = 2.8 Hz, R**C**H_2-OCF_3), 21.9 ppm. **^{19}F NMR (188 MHz, $CDCl_3$)** δ -61.46 ppm. **HRMS (ASAP +)** Calcd for $C_{10}H_{10}F_3O_2^+$ [M + H]$^+$: 219.0627. Found: 219.0627.

4-(2-(trifluoromethoxy)acetyl)benzonitrile 10

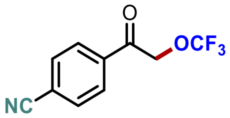

10

According to general procedure E, 4-(2-(trifluoromethoxy)acetyl)benzonitrile **10** was obtained from the corresponding enol carbonate **1g**; the yield of the desired product was determined by comparing the integration of the ^{19}F NMR resonance of the product with that of bis((trifluoromethyl)sulfonyl)amide. ^{19}F NMR yield of 42%. The product was isolated by flash column chromatography on silica gel hexane/EtOAc 8/2) in 27% yield (6.2 mg, 0.027 mmol). **^{1}H NMR (300 MHz, $CDCl_3$)** δ 8.01 (d, *J* = 7.8 Hz, 2H), 7.82 (d, *J* = 8.0 Hz, 2H), 5.15 (s, 2H) ppm. **^{13}C NMR (75 MHz, $CDCl_3$)** δ 189.6, 136.8, 133.0, 128.7, 121.7 (q, *J* = 257.4 Hz, RO**C**F_3), 117.8, 117.6, 68.5 (q, *J* = 3.0 Hz, R**C**H_2-OCF_3) ppm. **^{19}F NMR (188 MHz, $CDCl_3$)** δ -61.63 ppm. **HRMS (ASAP +)** Calcd for $C_{10}H_7F_3NO_2^+$ [M + H]$^+$: 230.0423. Found: 230.0422.

1-(4-acetylphenyl)-2-(trifluoromethoxy)ethan-1-one 11

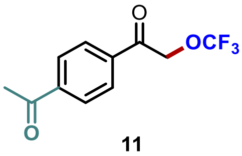

11

According to general procedure E, 1-(4-acetylphenyl)-2-(trifluoromethoxy)ethan-1-one **11** was obtained from the corresponding enol carbonate **1h**. The yield of the desired product was determined by comparing the integration of the ^{19}F NMR resonance of the product with that of bis((trifluoromethyl)sulfonyl)amide. ^{19}F NMR yield of 44%. **^{19}F NMR (188 MHz, $CDCl_3$)** δ -61.32 ppm.

2-(trifluoromethoxy)-1-(3-(trifluoromethyl)phenyl)ethanone 12

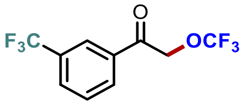

12

According to general procedure D, 2-(trifluoromethoxy)-1-(3-(trifluoromethyl)phenyl)ethanone **12** was obtained from the corresponding enol carbonate **1i**; the yield of the desired product was determined by comparing the integration of the ^{19}F NMR resonance of the product with that of bis((trifluoromethyl)sulfonyl)amide. ^{19}F NMR yield of 38%. PTLC conditions: 2 successive purifications with Pentane/Et_2O (9/1) and then 100% DCM. The product was isolated in 15% yield (4.0 mg, 0.015 mmol). **^{1}H NMR (300 MHz, $CDCl_3$)** δ 8.16 (s, 1H), 8.10 (d, *J* = 7.8 Hz, 1H), 7.90 (d, *J* = 7.7 Hz, 1H), 7.68 (t, *J* = 7.8 Hz, 1H), 5.18 (s, 2H) ppm. **^{13}C NMR (75 MHz, $CDCl_3$)** δ 189.4, 134.4, 131.9 (d, *J* = 33 Hz), 131.3, 130.8 (q, *J* = 3.5 Hz), 129.9, 125.1 (q, *J* = 3.9 Hz), 123.6 (d, *J* = 272 Hz) 121.7 (q, *J* = 256 Hz, RO**C**F_3), 68.4 (q, *J* = 3.0 Hz, R**C**H_2-OCF_3) ppm. **^{19}F NMR (188 MHz, $CDCl_3$)** δ -61.59 (O**C**F_3), -63.44 (**C**F_3) ppm. **HRMS (ASAP +)** Calcd for $C_{10}H_7F_6O_2^+$ [M + H]$^+$: 273.0345. Found: 273.0341.

1-(3,5-bis(trifluoromethyl)phenyl)-2-(trifluoromethoxy)ethan-1-one 13

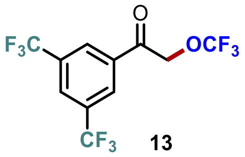

13

According to general procedure E, 1-(3,5-bis(trifluoromethyl)phenyl)-2-(trifluoromethoxy)ethan-1-one **13** was obtained from the corresponding enol carbonate **1j**. The yield of the desired product was determined by comparing the integration of the ^{19}F NMR resonance of the product with that of bis((trifluoromethyl)sulfonyl)amide. ^{19}F NMR yield of 30%. **^{19}F NMR (188 MHz, $CDCl_3$)** δ -61.23 (s, 3F), -63.36 (s, 6F) ppm.

1-(2-bromophenyl)-2-(trifluoromethoxy)ethan-1-one 14

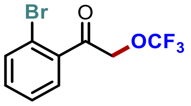

14

According to general procedure E, 1-(2-bromophenyl)-2-(trifluoromethoxy)ethan-1-one **14** was obtained from the corresponding enol carbonate **1k**. The yield of the desired product was determined by comparing the integration of the ^{19}F NMR resonance of the product with that of bis((trifluoromethyl)sulfonyl)amide. ^{19}F NMR yield of 36%. The product was isolated by flash column chromatography on silica gel (hexane/EtOAc 8/2) in 18% yield (5.1 mg, 0.018 mmol). Particular care was taken during solvent evaporation steps due to the observed volatility of the product. **^{1}H NMR (500 MHz, $CDCl_3$)** δ 7.66 (d, *J* = 7.8 Hz, 1H), 7.51 – 7.33 (m, 3H), 5.05 (s, 2H) ppm. **^{13}C NMR (126 MHz, $CDCl_3$)** δ 194.8, 137.8, 134.0, 133.1, 129.6, 127.8, 121.6 (q, *J* = 256.8 Hz, RO**C**F_3), 119.4, 69.8 (q, *J* = 3.0 Hz, R**C**H_2-OCF_3) ppm. **^{19}F NMR (188 MHz, $CDCl_3$)** δ -61.53 ppm. **HRMS (ASAP +)** Calcd for $C_8H_5BrF_3O_2^+$ [M + H]$^+$: 282.9576. Found: 282.9580.

1-(3-bromophenyl)-2-(trifluoromethoxy)ethan-1-one 15

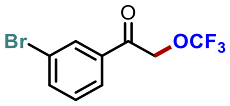

15

According to general procedure E, 1-(3-bromophenyl)-2-(trifluoromethoxy)ethan-1-one **15** was obtained from the corresponding enol carbonate **1l**. The yield of the desired product was determined by comparing the integration of the ^{19}F NMR resonance of the product with that of bis((trifluoromethyl)sulfonyl)amide. ^{19}F NMR yield of 38%. The product was isolated by flash column chromatography on silica gel (hexane/DCM 7/3) in 27% yield (7.6 mg, 0.027 mmol). **^{1}H NMR (500 MHz, $CDCl_3$)** δ 8.04 (t, *J* = 1.8 Hz, 1H), 7.83 (dt, *J* = 7.8, 1.3 Hz, 1H), 7.77 (ddd, *J* = 8.0, 2.0, 1.0 Hz, 1H), 7.40 (t, *J* = 7.9 Hz, 1H), 5.13 (s, 2H) ppm. **^{13}C NMR (126 MHz, $CDCl_3$)** δ 189.2, 137.4, 135.6, 131.2, 130.7, 126.6, 123.6, 121.8 (q, *J* = 256.8 Hz, RO**C**F_3), 68.3 (q, *J* = 3.0 Hz, R**C**H_2-OCF_3) ppm. **^{19}F NMR (188 MHz, $CDCl_3$)** δ -61.59 ppm. **HRMS (ASAP +)** Calcd for $C_8H_5BrF_3O_2^+$ [M + H]$^+$: 282.9576. Found: 282.9576.

1-(4-bromophenyl)-2-(trifluoromethoxy)ethan-1-one 16

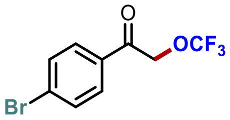

16

According to general procedure E, 1-(4-bromophenyl)-2-(trifluoromethoxy)ethan-1-one **16** was obtained from the corresponding enol carbonate **1m**. The yield of the desired product was determined by comparing the integration of the ^{19}F NMR resonance of the product with that of bis((trifluoromethyl)sulfonyl)amide. ^{19}F NMR yield of 46%. The product was isolated by flash column chromatography on silica gel (hexane/EtOAc 95/5) in 34% yield (9.6 mg, 0.034 mmol). **^{1}H NMR (500 MHz, $CDCl_3$)** δ 7.78 (d, *J* = 8.6 Hz, 2H), 7.66 (d, *J* = 8.6 Hz, 2H), 5.12 (s, 2H) ppm. **^{13}C NMR (126 MHz, $CDCl_3$)** δ 189.6, 132.6, 132.6, 129.8, 129.6, δ 121.8 (q, *J* = 256.8 Hz, RO**C**F_3), 68.3 (q, *J* = 3.0 Hz, R**C**H_2-OCF_3) ppm. **^{19}F NMR (188 MHz, $CDCl_3$)** δ -61.56 ppm. **HRMS (ASAP +)** Calcd for $C_8H_5BrF_3O_2^+$ [M + H]$^+$: 282.9576. Found: 282.9572.

1-phenyl-2-(trifluoromethoxy)propan-1-one 17

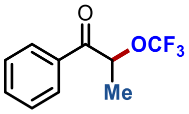

17

According to general procedure E, 1-phenyl-2-(trifluoromethoxy)propan-1-one **17** was obtained from the corresponding enol carbonate **1n**. The yield of the desired product was determined by comparing the integration of the ^{19}F NMR resonance of the product with that of bis((trifluoromethyl)sulfonyl)amide. ^{19}F NMR yield of 34%. The product was isolated by flash column chromatography on silica gel (hexane/DCM 8/2) in 32% yield (7.0 mg, 0.032 mmol). **^{1}H NMR (500 MHz, $CDCl_3$)** δ 7.97 (d, *J* = 8.0 Hz, 2H), 7.63 (t, *J* = 7.4 Hz, 1H), 7.51 (t, *J* = 7.7 Hz, 2H), 5.48 (q, *J* = 6.9 Hz, 1H), 1.66 (d, *J* = 6.9 Hz, 3H) ppm. **^{13}C NMR (126 MHz, $CDCl_3$)** δ 195.1, 134.2, 133.7, 129.1, 128.9, 121.6 (q, *J* = 256.2 Hz, RO**C**F_3), δ 75.5 (q, *J* = 2.5 Hz, R**C**H_2-OCF_3), 18.8 ppm. **^{19}F NMR (188 MHz, $CDCl_3$)** δ -59.29 ppm. **HRMS (ASAP +)** Calcd for $C_{10}H_{10}F_3O_2^+$ [M + H]$^+$: 219.0627. Found: 219.0629.

1,3-diphenyl-2-(trifluoromethoxy)propan-1-one 18

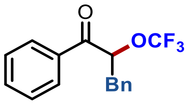

18

According to general procedure E, 1,3-diphenyl-2-(trifluoromethoxy)propan-1-one **18** was obtained from the corresponding enol carbonate **1o**. The yield of the desired product was determined by comparing the integration of the ^{19}F NMR resonance of the product with that of bis((trifluoromethyl)sulfonyl)amide. ^{19}F NMR yield of 31%. The product was isolated by flash column chromatography on silica gel (hexane/DCM 8/2) in 27% yield (7.9 mg, 0.027 mmol). **^{1}H NMR (500 MHz, $CDCl_3$)** δ 7.98 – 7.83 (m, 2H), 7.69 – 7.55 (m, 1H), 7.55 – 7.42 (m, 2H), 7.32 – 7.28 (m, 2H), 7.27 – 7.24 (m, 1H), 7.24 – 7.20 (m, 2H), 5.50 (dd, *J* = 7.8, 5.3 Hz, 1H), 3.31 – 3.14 (m, 2H) ppm. **^{13}C NMR (126 MHz, $CDCl_3$)** δ 194.7, 135.0, 134.2, 134.2, 129.5, 129.1, 128.9, 127.6, 121.4 (q, *J* = 256.8 Hz, RO**C**F_3), 79.5 (q, *J* = 2.0 Hz, R**C**H_2-OCF_3), 39.0 ppm. **^{19}F NMR (188 MHz, $CDCl_3$)** δ -59.46 ppm. **HRMS (ASAP +)** Calcd for $C_{10}H_{10}F_3O_2^+$ [M + H]$^+$: 295.0940. Found: 295.0941.

2-(trifluoromethoxy)-2,3-dihydro-1H-inden-1-one 19

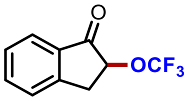

19

According to general procedure D, 2-(trifluoromethoxy)-2,3-dihydro-1H-inden-1-one 19 was obtained from the corresponding enol carbonate **1p**; the yield of the desired product was determined by comparing the integration of the ^{19}F NMR resonance of the product with that of bis((trifluoromethyl)sulfonyl)amide. ^{19}F NMR yield of 28%. PTLC conditions: Pentane/Et_2O (85/15, 2 elutions). **^{1}H NMR (300 MHz, $CDCl_3$)** δ 7.82 (d, *J* = 7.7 Hz, 1H), 7.68 (t, *J* = 7.1 Hz, 1H), 7.50 – 7.40 (m, 2H), 4.98 – 4.89 (m, 1H), 3.68 (dd, *J* = 17.1, 8.0 Hz, 1H), 3.26 (dd, *J* = 16.7, 4.2 Hz, 1H) ppm. **^{13}C NMR (75 MHz, $CDCl_3$)** δ 197.6, 149.7, 136.6, 133.8, 128.7, 126.8, 125.0, 122.1 (q, *J* = 257 Hz, RO**C**F_3), 33.9 ppm. **^{19}F NMR (188 MHz, $CDCl_3$)** δ -59.73 ppm. **HRMS (ASAP +)** Calcd for $C_{20}H_{15}F_6O_4^+$ [2M + H]$^+$: 433.0869. Found: 433.0864.

5-fluoro-2-(trifluoromethoxy)-2,3-dihydro-1H-inden-1-one 20

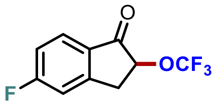

20

According to general procedure D, 5-fluoro-2-(trifluoromethoxy)-2,3-dihydro-1H-inden-1-one **20** was obtained from the corresponding enol carbonate **1q**; the yield of the desired product was determined by comparing the integration of the ^{19}F NMR resonance of the product with that of bis((trifluoromethyl)sulfonyl)amide. ^{19}F NMR yield of 30%. The product was isolated by PTLC (Pentane/Et_2O (85/15, 2 elutions)) in 22% yield (5.2 mg, 0.022 mmol). **^{1}H NMR (300 MHz, $CDCl_3$)** δ 7.88 – 7.80 (m, 1H), 7.20 – 7.09 (m, 2H), 4.98 – 4.89 (m, 1H), 3.67 (dd, *J* = 17.3, 7.9 Hz, 1H), 3.25 (dd, *J* = 17.1, 4.8 Hz, 1H) ppm. **^{13}C NMR (75 MHz, $CDCl_3$)** δ 195.6, 168.19 (d, *J* = 259 Hz, F-**C_{Ar}**), 152.66 (d, *J* = 10.5 Hz), 130.3, 127.61 (d, *J* = 10.6 Hz), 122.03 (q, *J* = 257 Hz, RO**C**F_3), 117.20 (d, *J* = 23.8 Hz), 113.62 (d, *J* = 22.8 Hz), 76.38 (q, *J* = 2.4 Hz, R**C**H_2-OCF_3), 33.8 ppm. **^{19}F NMR (188 MHz, $CDCl_3$)** δ -59.80 (s, CF_3), -99.41 (q, *J* = 7.7 Hz) ppm. **HRMS (ASAP +)** Calcd for $C_{10}H_7F_4O_2^+$ [M + H]$^+$: 235.0377. Found: 235.0380.

6-(trifluoromethoxy)-6,7,8,9-tetrahydro-5H-benzo[7]annulen-5-one 21

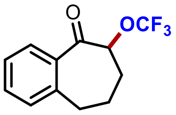

21

According to general procedure D, 6-(trifluoromethoxy)-6,7,8,9-tetrahydro-5H-benzo[7]annulen-5-one **21** was obtained from the corresponding enol carbonate **1r**; the yield of the desired product was determined by comparing the integration of the ^{19}F NMR resonance of the product with that of bis((trifluoromethyl)sulfonyl)amide. ^{19}F NMR yield of 24%. PTLC conditions: Pentane/Et_2O (85/15). **^{1}H NMR (300 MHz, $CDCl_3$)** δ 7.71 (d, *J* = 7.6 Hz, 1H), 7.44 (t, *J* = 7.4 Hz, 1H), 7.33 (t, *J* = 7.6 Hz, 1H), 7.23 (s, 1H), 4.99 (t, *J* = 7.0 Hz, 1H), 3.14 – 2.91 (m, 2H), 2.40 – 2.24 (m, 1H), 2.22 – 2.0 (m, 2H), 1.99 – 1.81 (m, 1H) ppm. **^{13}C NMR (75 MHz, $CDCl_3$)** δ 198.9, 141.7, 136.5, 132.4, 130.2, 129.4, 127.0, 121.50 (q, *J* = 257 Hz, RO**C**F_3), 81.0 (q, *J* = 2.6 Hz, R**C**H_2-OCF_3). 34.2, 30.8, 23.2 ppm. **^{19}F NMR (188 MHz, $CDCl_3$)** δ -59.52 ppm. **HRMS (ASAP +)** Calcd for $C_{12}H_{12}F_3O_2^+$ [M + H]$^+$: 245.0784. Found: 245.0781.

2,2-difluoro-1-phenyl-2-(trifluoromethoxy)ethan-1-one 22

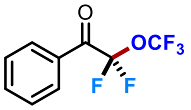

22

According to general procedure E, 2,2-difluoro-1-phenyl-2-(trifluoromethoxy)ethan-1-one **22** was obtained from the corresponding enol carbonate **1s**. The yield of the desired product was determined by comparing the integration of the ^{19}F NMR resonance of the product with that of bis((trifluoromethyl)sulfonyl)amide. ^{19}F NMR yield of 20%. **^{19}F NMR (188 MHz, $CDCl_3$)** δ -54.91 (t, J = 8.7 Hz, 3F), -76.80 (q, J = 9.1 Hz, 2F) ppm.

2,2-difluoro-1-(naphthalen-2-yl)-2-(trifluoromethoxy)ethan-1-one 23

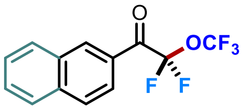

23

According to general procedure E, 2,2-difluoro-1-(naphthalen-2-yl)-2-(trifluoromethoxy)ethan-1-one **23** was obtained from the corresponding enol carbonate **1t**. The yield of the desired product was determined by comparing the integration of the ^{19}F NMR resonance of the product with that of bis((trifluoromethyl)sulfonyl)amide. ^{19}F NMR yield of 19%. **^{19}F NMR (188 MHz, $CDCl_3$)** δ -54.92 (t, J = 8.7 Hz, 3F), -76.23 (q, J = 8.5 Hz, 2F) ppm.

1-(trifluoromethoxy)octan-2-one 24

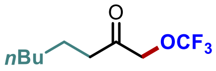

24

According to general procedure E, 1-(trifluoromethoxy)octan-2-one **24** was obtained from the corresponding enol carbonate **1u**. The yield of the desired product was determined by comparing the integration of the ^{19}F NMR resonance of the product with that of bis((trifluoromethyl)sulfonyl)amide. ^{19}F NMR yield of 16%. **^{19}F NMR (188 MHz, $CDCl_3$)** δ -61.30 ppm.

(E)-4-phenyl-1-(trifluoromethoxy)but-3-en-2-one 25

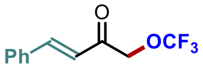

25

According to general procedure D, (E)-4-phenyl-1-(trifluoromethoxy)but-3-en-2-one **25** was obtained from the corresponding enol carbonate **1v**; the yield of the desired product was determined by comparing the integration of the ^{19}F NMR resonance of the product with that of bis((trifluoromethyl)sulfonyl)amide. ^{19}F NMR yield of 21%. PTLC conditions: Pentane/Et_2O (9/1, 2 elutions). **^{1}H NMR (300 MHz, $CDCl_3$)** δ 7.76 (d, *J* = 16.1 Hz, 1H), 7.65 – 7.56 (m, 2H), 7.48 – 7.36 (m, 3H), 6.97 (d, *J* = 16.1 Hz, 1H), 4.69 (s, 2H) ppm. **^{13}C NMR (75 MHz, $CDCl_3$)** δ 191.4, 145.7, 134.0, 131.5, 129.2, 128.9, 128.6, 121.7 (q, *J* = 256 Hz, ROCF_3), 69.9 (q, *J* = 2.5 Hz, RCH_2-OCF_3) ppm. **^{19}F NMR (188 MHz, $CDCl_3$)** δ -61.63 ppm. **HRMS (ASAP +)** Calcd for $C_{11}H_{10}F_3O_2^+$ [M + H]$^+$: 231.0627. Found: 231.0634.

(E)-1-(trifluoromethoxy)non-3-en-2-one 26

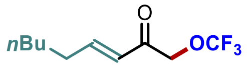

26

According to general procedure D, (E)-1-(trifluoromethoxy)non-3-en-2-one **26** was obtained from the corresponding enol carbonate **1w**; the yield of the desired product was determined by comparing the integration of the ^{19}F NMR resonance of the product with that of bis((trifluoromethyl)sulfonyl)amide. ^{19}F NMR yield of 18%. **^{19}F NMR (188 MHz, CDCl$_3$)** δ -61.67 ppm. **HRMS (ASAP +)** Calcd for $C_{10}H_{16}F_3O_2^+$ $[M + H]^+$: 225.1097. Found: 225.1093.

1-(3,5,5,6,8,8-hexamethyl-5,6,7,8-tetrahydronaphthalen-2-yl)-2-(trifluoromethoxy)ethan-1-one 27

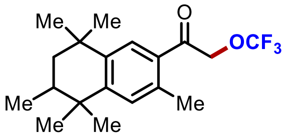

27

According to general procedure E, 1-(3,5,5,6,8,8-hexamethyl-5,6,7,8-tetrahydronaphthalen-2-yl)-2-(trifluoromethoxy)ethan-1-one **27** was obtained from the corresponding enol carbonate **1x**. The yield of the desired product was determined by comparing the integration of the ^{19}F NMR resonance of the product with that of bis((trifluoromethyl)sulfonyl)amide. ^{19}F NMR yield of 33%. The product was isolated by flash column chromatography on silica gel (hexane/DCM 7/3) in 26% yield (8.9 mg, 0.026 mmol). **^{1}H NMR (500 MHz, CDCl$_3$)** δ 7.48 (s, 1H), 7.26 (s, 1H), 5.02 (s, 2H), 2.50 (s, 3H), 1.88 (dqd, *J* = 13.5, 6.8, 2.6 Hz, 1H), 1.64 (t, *J* = 13.3 Hz, 1H), 1.41 (dd, *J* = 13.6, 2.6 Hz, 1H), 1.33 (s, 3H), 1.32 (s, 3H), 1.27 (s, 3H), 1.08 (s, 3H), 1.00 (d, *J* = 6.8 Hz, 3H) ppm. **^{13}C NMR (126 MHz, CDCl$_3$)** δ 193.0, 151.7, 142.7, 136.5, 131.3 (x2), 127.2, 121.8, (q, *J* = 256.2 Hz, RO**C**F$_3$), 69.3 (q, *J* = 2.9 Hz, R**C**H$_2$-OCF$_3$), 43.4, 38.2, 34.5, 34.2, 32.6, 32.0, 28.4, 24.8, 21.3, 16.9 ppm. **^{19}F NMR (188 MHz, CDCl$_3$)** δ -61.29 ppm. **HRMS (ESI+)** Calcd. for $C_{19}H_{26}F_3O_2^+$ $[M + H^+]^+$: 343.1879. Found: 343.1870.

1-(4-(tert-butyl)-2,6-dimethyl-3,5-dinitrophenyl)-2-(trifluoromethoxy)ethanone 28

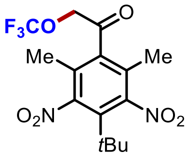

28

According to general procedure D, 1-(4-(tert-butyl)-2,6-dimethyl-3,5-dinitrophenyl)-2-(trifluoromethoxy)ethanone **28** was obtained from the corresponding enol carbonate **1y**; the yield of the desired product was determined by comparing the integration of the ^{19}F NMR resonance of the product with that of bis((trifluoromethyl)sulfonyl)amide. ^{19}F NMR yield of 29%. The product was isolated by PTLC (Pentane/Et$_2$O 9/1) in 21% yield (7.9 mg, 0.021 mmol) as a white solid. **^{1}H NMR (300 MHz, CDCl$_3$)** δ 4.74 (s, 2H), 2.12 (s, 6H), 1.45 (s, 9H) ppm. **^{13}C NMR (75 MHz, CDCl$_3$)** δ 197.6, 150.7, 138.5, 133.8, 128.5, 121.4 (q, *J* = 258 Hz, RO**C**F$_3$), 70.7 (q, *J* = 2.9 Hz, R**C**H$_2$-OCF$_3$), 37.8, 30.4, 15.2 ppm. **^{19}F NMR (188 MHz, CDCl$_3$)** δ -61.74 ppm. **HRMS (ASAP +)** Calcd for $C_{15}H_{18}F_3N_2O_6^+$ $[M + H]^+$: 379.1111. Found: 379.1126.

1-(6-(tert-butyl)-1,1-dimethyl-2,3-dihydro-1H-inden-4-yl)-2-(trifluoromethoxy)ethanone 29

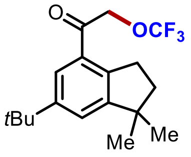

29

According to general procedure D, 1-(6-(tert-butyl)-1,1-dimethyl-2,3-dihydro-1H-inden-4-yl)-2-(trifluoromethoxy)ethanone **29** was obtained from the corresponding enol carbonate **1z**; the yield of the desired product was determined by comparing the integration of the ^{19}F NMR resonance of the product with that of bis((trifluoromethyl)sulfonyl)amide. ^{19}F NMR yield of 20%. The product was isolated by PTLC (Pentane/EtOAc (98/2, 2 elutions)) in 16% yield (5.2 mg, 0.016 mmol). **^{1}H NMR (300 MHz, $CDCl_3$)** δ 7.52 (s, 1H), 7.40 (s, 1H), 5.12 (s, 2H), 3.17 (t, *J* = 7.3 Hz, 2H), 1.96 (t, *J* = 7.3 Hz, 2H), 1.36 (s, 9H), 1.27 (s, 6H) ppm. **^{13}C NMR (75 MHz, $CDCl_3$)** δ 191.8, 155.2, 150.5, 142.2, 130.2, 124.7, 123.2, 117.5 (q, *J* = 336.7 Hz, RO**C**F_3), 69.2 (q, *J* = 2.6 Hz, R**C**H_2-OCF_3). 43.7, 41.5, 34.9, 31.6, 30.9, 28.9 ppm. **^{19}F NMR (188 MHz, $CDCl_3$)** δ -61.24 ppm. **HRMS (ASAP +)** Calcd for $C_{18}H_{24}F_3O_2^+$ [M + H]$^+$: 329.1723. Found: 329.1679.

(E)-1-(trifluoromethoxy)-4-(2,6,6-trimethylcyclohex-2-en-1-yl)but-3-en-2-one 30

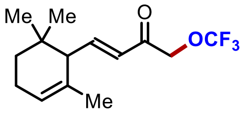

30

According to general procedure D, (E)-1-(trifluoromethoxy)-4-(2,6,6-trimethylcyclohex-2-en-1-yl)but-3-en-2-one **30** was obtained from the corresponding enol carbonate **1aa**; the yield of the desired product was determined by comparing the integration of the ^{19}F NMR resonance of the product with that of bis((trifluoromethyl)sulfonyl)amide. ^{19}F NMR yield of 14%. The product was isolated by PTLC (Pentane/EtOAc 98/2) in 11% yield (3.2 mg, 0.011 mmol). **^{1}H NMR (200 MHz, $CDCl_3$)** δ 6.87 (dd, *J* = 15.7, 9.8 Hz, 1H), 6.28 (d, *J* = 15.6 Hz, 1H), 5.53 (bs, 1H), 4.61 (s, 2H), 2.32 (d, *J* = 9.6 Hz, 1H), 1.66 (s, 1H), 2.14 – 1.98 (m, 3H), 1.52 (s, 3H), 0.93 (s, 3H), 0.85 (s, 3H) ppm. **^{13}C NMR (151 MHz, $CDCl_3$)** δ 191.04, 151.82, 131.35, 125.79, 123.39, 69.54 (q, *J* = 2.9 Hz, **C**H_2OCF_3), 54.77, 28.00, 27.81, 26.89, 23.14, 22.87 ppm. **^{19}F NMR (188 MHz, $CDCl_3$)** δ -61.62 ppm. **HRMS (ASAP +)** Calcd for $C_{14}H_{20}F_3O_2^+$ [M + H]$^+$: 277.1410. Found: 277.1407.

1-(3-methoxy-10,13-dimethyl-tetradecahydro-1H-cyclopenta[a]phenanthren-17-yl)-2-(trifluoromethoxy)ethan-1-one 31

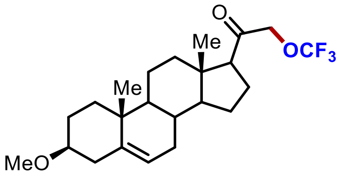

31

According to general procedure E, 1-((3*S*,8*S*,9*S*,10*R*,13*S*,14*S*)-3-methoxy-10,13-dimethyl-tetradecahydro-1*H*-cyclopenta[*a*]phenanthren-17-yl)-2-(trifluoromethoxy)ethan-1-one **31** was obtained from the corresponding enol carbonate **1ab**. The yield of the desired product was determined by comparing the integration of the ^{19}F NMR resonance of the product with that of bis((trifluoromethyl)sulfonyl)amide. ^{19}F NMR yield of 10%. **^{19}F NMR (188 MHz, CDCl3)** δ -61.10 ppm.

(8R,9S,10R,13S,14S,17S)-17-methoxy-10,13-dimethyl-6-(trifluoromethoxy)-dodecahydro-1H-cyclopenta[a]phenanthren-3(2H)-one 32

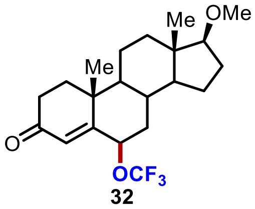

32

According to general procedure D, (8R,9S,10R,13S,14S,17S)-17-methoxy-10,13-dimethyl-6-(trifluoromethoxy)-dodecahydro-1H-cyclopenta[a]phenanthren-3(2H)-one **32** was obtained from the corresponding enol carbonate **1ac**; the yield of the desired product was determined by comparing the integration of the ^{19}F NMR resonance of the product with that of bis((trifluoromethyl)sulfonyl)amide. ^{19}F NMR yield of 11%. PTLC conditions: Pentane/Et_2O (9/1). The compound was isolated as an inseparable 1:1 mixture of diastereoisomers. In ^{1}H NMR, only characteristic peaks are listed.[11] **^{1}H NMR (600 MHz, $CDCl_3$)** δ 5.82 (d, *J* = 1.0 Hz, 1H, C=C**H**C=O), 5.77 (d, *J* = 1.4 Hz, 1H, C=C**H**C=O), 4.72 (dd, *J* = 14.0, 5.3 Hz, 1H, C**H**OCF_3), 4.57 (dd, *J* = 9.2, 4.5 Hz, 1H, C**H**OCF_3), 3.35 (s, 3H x2, OC**H**$_3$), 3.25 – 3.20 (m, 2H x2, C**H**OCH3), 2.54 – 2.45 (m, 1H, C**H**$_2$C=O), 2.34 (dt, *J* = 4.9, 2.3 Hz, 1H, C**H**$_2$C=O), 2.29 (dd, *J* = 4.2, 2.6 Hz, 1H, C**H**$_2$C=O), 2.27 (dt, *J* = 5.2, 2.7 Hz, 1H, C**H**$_2$C=O), 1.32 (s, 3H, C-19 C**H**$_3$), 1.27 (s, 3H, C-19 C**H**$_3$), 0.80 (s, 3H, C-18 C**H**$_3$), 0.80 (s, 3H, C-18 C**H**$_3$). **^{13}C NMR (151 MHz, $CDCl_3$)** δ 191.5, 191.1, 173.6, 171.3, 123.5 (d, *J* = 239.1 Hz, RO**C**F$_3$), 121.8, 120.8, 117.1, 90.8, 90.5, 75.2 – 75.1 (m, **C**HOCF_3), 74.5 – 74.4 (m, **C**HOCF_3), 58.0 (x2), 54.4, 54.2, 52.7, 51.0, 50.7 (x2), 43.2, 43.0, 42.9, 42.7, 40.9, 40.4, 39.6, 38.0, 37.8, 37.7, 37.6, 35.7, 35.5, 35.0, 33.7, 33.6, 33.1, 32.7, 32.5, 32.1, 31.6, 29.8, 27.8, 27.7, 27.6, 23.4 (x3), 21.9, 21.8, 21.2, 20.8, 18.3, 17.6, 11.8, 11.7 (x2). **^{19}F NMR (188 MHz, $CDCl_3$)** δ -59.05 (s, CF_3), -59.11 (s, CF_3) ppm. **HRMS (ASAP +)** Calcd for $C_{21}H_{30}F_3O_3^+$ [M + H]$^+$: 387.2142. Found: 387.2152.

Poorly reactive substrates

In Scheme S2 the trifluoromethoxylation products of poorly reactive enol carbnates are reported. The indicated yield refers to the ^{19}F NMR yield measured with the peak of triflimide as the internal standard.

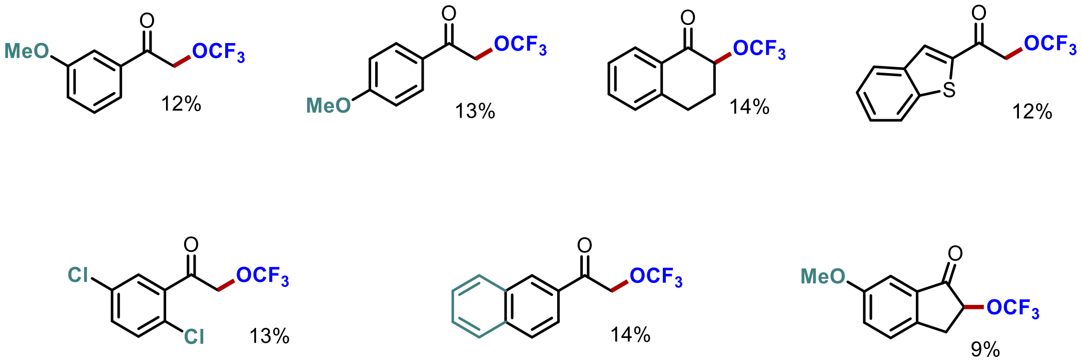

Scheme S2 Trifluoromethoxylation products of poorly reactive enol carbonates

Scale-up experiments

4-(2-(trifluoromethoxy)acetyl)benzonitrile 10

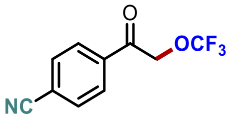

10

A round-bottomed flask purged with argon was charged with 4-cyano-1-(trifluoromethoxy)pyridin-1-ium bis((trifluoromethyl)sulfonyl)amide **2a** (939 mg, 2.1 mmol, 1 equiv.), tert-butyl (1-(4-cyanophenyl)vinyl) carbonate **1g** (2.58 g, 10.5 mmol, 5 equiv.) and 4CzIPN (83 mg, 0.11 mmol, 0.05 equiv.). Dry acetonitrile (210 mL, 0.01 M) was finally added and the reaction medium purged with argon again. 200 mL of the resulting mixture were taken at a flow rate of 5mL/min (2 minutes of residence time in a 10 mL coil UV-150 reactor) by the VapourTec apparatus, equipped with a blue lamp irradiating at 450 nm inside the reactor, at 50°C. The resulting mixture exiting the apparatus was concentrated under vacuum. ^{19}F NMR yield of 37%. 4-(2-(trifluoromethoxy)acetyl)benzonitrile **10** was isolated as a white solid by column chromatography on silica gel (Pentane/EtOAc 9/1) in 36% yield (165 mg, 0.72 mmol).

The scaled-up synthesis of **10** was then repeated with 4-cyano-1-(trifluoromethoxy)pyridin-1-ium bis((trifluoromethyl)sulfonyl)amide **2a** (1.06 g, 2.26 mmol, 1 equiv.) and tert-butyl (1-(4-cyanophenyl)vinyl) carbonate **1g** (3.0 g, 13.6 mmol, 6 equiv.). 4-(2-(trifluoromethoxy)acetyl)benzonitrile **10** was isolated as a white solid by column chromatography on silica gel (Pentane/EtOAc 9/1) in 50% yield (260 mg, 1.13 mmol).

1-(4-bromophenyl)-2-(trifluoromethoxy)ethan-1-one 16

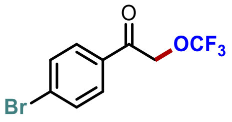

16

A 250 mL round-bottomed flask purged with argon was charged with 4-cyano-1-(trifluoromethoxy)pyridin-1-ium bis((trifluoromethyl)sulfonyl)amide **2a** (469 mg, 1 mmol, 1 equiv.), 1-(4-bromophenyl)vinyl tert-butyl carbonate **1m** (1.50 g, 5 mmol, 5 equiv.) and 4CzIPN (39 mg, 0.05 mmol, 0.05 equiv.). Dry acetonitrile (100 mL, 0.01 M) was finally added and the reaction medium purged with argon again. The resulting mixture was taken at a flow rate of 5 mL/min (2 minutes of residence time in a 10 mL coil UV-150 reactor) by the VapourTec apparatus, equipped with a medium pressure mercury lamp set at 75% of its maximum power output and a band-pass filter N°4 irradiating at 370 nm inside the reactor, at 50°C. The resulting mixture exiting the apparatus was treated with 10 mL of 6M HCl and the reaction mixture stirred until complete hydrolysis of the unreacted starting material (controlled by TLC analysis). The crude reaction mixture was then quenched with sat. $NaHCO_3$ until gas evolution stopped, diluted with DCM (200 mL) and washed with brine (3x50 mL). The organic phase was dried over $MgSO_4$ and concentrated under vacuum. ^{19}F NMR yield of 37%. 1-(4-bromophenyl)-2-(trifluoromethoxy)ethan-1-one **16** was isolated as a yellow solid by column chromatography on silica gel (hexane/EtOAc 95/5) in 33% yield (94 mg, 0.33 mmol).

Post-modifications

1-(4-bromophenyl)-2-(trifluoromethoxy)ethan-1-ol 33

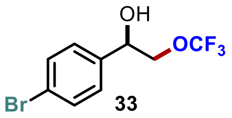

33

A 4 mL vial was charged with 1-(4-bromophenyl)-2-(trifluoromethoxy)ethan-1-one **16** (30 mg, 0.106 mmol, 1 equiv.) and MeOH was added (300 µL). The solution was cooled to 0°C and $NaBH_4$ (4.8 mg, 0.127 mmol, 1.2 equiv.) was added. The reaction mixture was stirred at 0°C for 1h, after which time it was quenched with 1M HCl (1 mL), extracted with EtOAc (3x10 mL) and washed with brine (10 mL). The organic phase was dried over $MgSO_4$ and concentrated under vacuum. 1-(4-bromophenyl)-2-(trifluoromethoxy)ethan-1-ol **33** was isolated without the need of further purification as a yellow oil,

which solidified upon standing, in 88% yield (26.6 mg, 0.093 mmol). **^{1}H NMR (500 MHz, $CDCl_3$)** δ 7.52 (d, *J* = 8.4 Hz, 2H), 7.28 (d, *J* = 8.4 Hz, 2H), 4.97 (dd, *J* = 8.5, 3.4 Hz, 1H), 4.05 (dd, *J* = 10.2, 3.5 Hz, 1H), 3.98 (dd, *J* = 10.2, 8.5 Hz, 1H), 2.44 (br s, 1H) ppm. **^{13}C NMR (126 MHz, $CDCl_3$)** δ 137.5, 132.1, 128.0, 122.8, 119.7 (q, *J* = 238.3 Hz, RO**C**F_3), 71.6 (q, *J* = 2.6 Hz, R**C**H_2-OCF_3), 71.4 ppm. **^{19}F NMR (188 MHz, $CDCl_3$)** δ -61.24 ppm. **HRMS (ESI-)** Calcd. for $C_{18}H_{14}O_4Br_2F_6K^-$ $[2M - 2H + K]^-$: 604.8806. Found: 604.8813.

1-(4-morpholinophenyl)-2-(trifluoromethoxy)ethan-1-ol 34

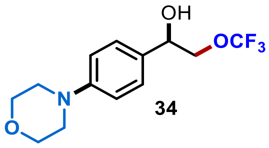

The procedure was adapted from the literature.[12] A 4 mL vial purged with nitrogen was charged with 1-(4-bromophenyl)-2-(trifluoromethoxy)ethan-1-ol **33** (14 mg, 0.05 mmol, 1 equiv.), $Pd(OAc)_2$ (0.22 mg, 0.001 mmol, 0.02 equiv.), 2-(di-*tert*-butylphosphino)biphenyl (JohnPhos, 0.44 mg, 0.0015 mmol, 0.03 equiv.) and anhydrous Cs_2CO_3 (24 mg, 0.075 mmol, 1.5 equiv.). The vial was closed with a cap equipped with a septum and 400 µL of degassed anhydrous 1,4-dioxane were added. Morpholine (5 µl, 0.06 mmol, 1.2 equiv.) was added and the reaction was stirred at 80°C for 16 hours, after which time the reaction mixture was concentrated under reduced pressure. ^{19}F NMR yield of 19%. 1-(4-morpholinophenyl)-2-(trifluoromethoxy)ethan-1-ol **34** was isolated as a yellow oil by column chromatography on silica gel (hexane/EtOAc 1/1) in 22% yield (3.2 mg, 0.011 mmol). **^{1}H NMR (500 MHz, $CDCl_3$)** δ 7.32 – 7.27 (m, 2H), 6.94 – 6.89 (m, 2H), 4.93 (t, *J* = 6.2 Hz, 2H), 4.07 – 3.98 (m, 2H), 3.91 – 3.78 (m, 4H), 3.20 – 3.13 (m, 4H), 2.34 (d, *J* = 2.9 Hz, 1H) ppm. **^{13}C NMR (126 MHz, $CDCl_3$)** δ 151.71, 129.65, 127.39, 121.78 (q, *J* = 255.0 Hz, RO**C**F_3), 115.76, 71.90 (q, *J* = 2.3 Hz, R**C**H_2-OCF_3), 71.63, 66.98, 49.23 ppm. **^{19}F NMR (188 MHz, $CDCl_3$)** δ -61.10 ppm. **HRMS (ASAP +)** Calcd for $C_{13}H_{17}F_3NO_3^+$ $[M + H]^+$: 292.1155. Found: 292.1161.

1-(4-bromophenyl)-2-(trifluoromethoxy)ethan-1-aminium acetate 35

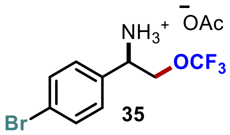

The procedure was adapted from the literature.[13] A flame-dried schlenk tube purged with nitrogen was charged with 1-(4-bromophenyl)-2-(trifluoromethoxy)ethan-1-one **16** (14 mg, 0.05 mmol), anhydrous NH_4OAc (77 mg, 1 mmol, 20 equiv.) and $NaBH_3CN$ (8.5 mg, 0.135 mmol, 2.7 equiv.). Finally, 200 µL of anhydrous MeOH were added and the schlenk tube sealed with a screw cap. The reaction was stirred at 70°C for 16 hours, after which time the reaction was concentrated under reduced pressure. ^{19}F NMR yield of 34%. 1-(4-bromophenyl)-2-(trifluoromethoxy)ethan-1-aminium acetate **35** was isolated as a white solid by column chromatography on silica gel (DCM/MeOH 95/5) in 31% yield (5.3 mg, 0.016 mmol). **^{1}H NMR (500 MHz, $CDCl_3$)** δ 7.50 (d, *J* = 8.4 Hz, 2H), 7.28 (d, *J* = 8.4 Hz, 2H), 4.66 (s, 3H), 4.31 (dd, *J* = 9.1, 4.2 Hz, 1H), 4.05 (dd, *J* = 9.7, 4.0 Hz, 1H), 3.94 (t, *J* = 9.1 Hz, 1H), 2.09 (s, 3H) ppm. **^{13}C NMR (126 MHz, $CDCl_3$)** δ 175.8, 132.3, 132.1, 128.8, 122.4, 121.7 (q, *J* = 255.3 Hz, RO**C**F_3), 71.9, 54.1, 20.7 ppm. **^{19}F NMR (188 MHz, $CDCl_3$)** δ -61.24 ppm. **HRMS (ESI+)** Calcd. for $C_9H_7OBrF_3^+$ $[M + H - NH_3]^+$: 266.9627. Found: 266.9619.

4-(3-(dimethylamino)-2-(trifluoromethoxy)acryloyl)benzonitrile 36

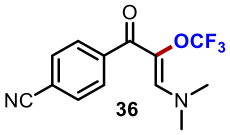

36

The procedure was adapted from the literature.[14] A 4 mL vial was charged with 4-(2-(trifluoromethoxy)acetyl)benzonitrile **10** (20 mg, 0.09 mmol, 1 equiv.) and N,N-dimethylformamide dimethyl acetal (25 µL, 0.18 mmol, 2 equiv.). The vial was closed with a septum and stirred at 90°C for 3h. The resulting mixture was then diluted in CH_2Cl_2 (10 mL) and washed with water (10 mL), dried over $MgSO_4$ and concentrated under vacuum. The product **36** was isolated by PTLC (Pentane/EtOAc 6/4) in 64% yield (16.5 mg, 0.06 mmol) as a yellow solid. **^{1}H NMR (300 MHz, $CDCl_3$)** δ 7.69 (s, 4H), 7.11 (bs, 1H), 3.16 (s, 6H) ppm. **^{13}C NMR (75 MHz, $CDCl_3$)** 186.5, 145.2 (bs, R**C**=C-N$(CH_3)_2$), 143.3, 132.1, 129.0, 122.1 (bs, RC=**C**-N$(CH_3)_2$), 121.2 (q, J = 257.1 Hz, RO**C**F_3), 118.5, 114.1 ppm. **^{19}F NMR (188 MHz, $CDCl_3$)** δ -59.29 (s, 3F) ppm. **HRMS (ASAP +)** Calcd for $C_{13}H_{11}F_3N_2NaO_2^+$ $[2M + Na]^+$: 591.1438. Found: 591.1440.

One-pot procedure for the trifluoromethoxylation of acetophenone

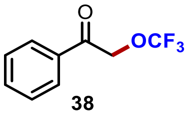

38

A round-bottomed flask was charged with acetophenone **37** (5 mmol, 1 equiv.) and dissolved in dry THF (5 mL, 1M) at 0°C under nitrogen atmosphere. NaH (60% dispersion in mineral oil, 400 mg, 10 mmol, 2 equiv.) was added portionwise to the reaction mixture, which was then stirred for 15 mins at 0°C. Boc_2O (1.38 mL, 6 mmol, 1.2 equiv.) was then added dropwise and the reaction mixture was stirred for 1h at room temperature. The resulting mixture was then quenched with water (20 mL), diluted with CH_2Cl_2 (50 mL) and washed with brine (3x20 mL). The organic phase was then dried over $MgSO_4$ and concentrated under vacuum. The resulting crude compound was diluted with 1 mL of $CDCl_3$ and trichloroethylene (450 µL, 5 mmol) was added as the internal standard to determine the yield by ^{1}H NMR. ^{1}H NMR yield: 43%. The crude was then evaporated and redissolved in 25 mL of DCM in a volumetric flask. 2.9 mL of the resulting solution (containing 0.25 mmol of enol carbonate according to the previously measured ^{1}H NMR yield) were placed in an 8 mL vial and the solvent evaporated. The vial was then purged with argon and charged with 4-cyano-1-(trifluoromethoxy)pyridin-1-ium bis((trifluoromethyl)sulfonyl)amide **2a** (23.5 mg, 0.05 mmol, 1 equiv.) and 4CzIPN (1.9 mg, 0.0025 mmol, 0.05 equiv.) and subjected to the trifluoromethoxylation reaction according to General procedure E. The yield of product **38** was determined by comparing the integration of the ^{19}F NMR resonance of the product with that of bis((trifluoromethyl)sulfonyl)amide. ^{19}F NMR yield of 23%. The compound is reported in the literature.[15]

G. Mechanistic studies

Alpha-trifluoromethoxylation of 1b with Pyridinium 2b and 4CzIPN as the photocatalyst

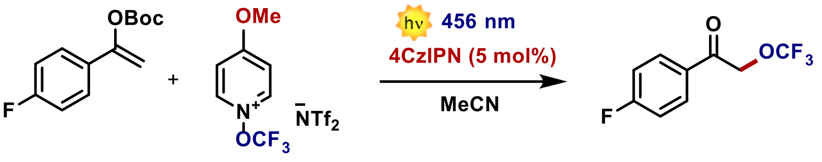

1b 2b 3

An 8-mL vial surrounded by blue LEDs with a watertight protection, equipped with a screwcap and a septum was purged with argon and charged with 4-methoxy-1-(trifluoromethoxy)pyridin-1-ium bis((trifluoromethyl)sulfonyl)amide **2b** (23.7 mg, 0.05 mmol, 1 equiv.), 1-(4-fluorophenyl)vinyl tert-butyl carbonate **1b** (0.25 mmol, 5 equiv.) and 4CzIPN (1.9 mg, 0.0025 mmol, 0.05 equiv.). Dry acetonitrile (5 mL, 0.01 M) was finally added and the reaction medium purged with argon again. The resulting mixture in the vial was placed in a water bath and allowed to stir vigorously for 1h at 50°C under irradiation of the blue LEDs. The yield of product **3** was determined by comparing the integration of the ^{19}F NMR resonance of the product with that of bis((trifluoromethyl)sulfonyl)amide. ^{19}F NMR yield of 12%.

Luminescence quenching experiments

Rates of quenching (k_q) were determined using Stern-Volmer kinetics:

$$I_0/I = k_q\tau_0[\text{quencher}] + 1$$

Where I_0 is the luminescence intensity without the quencher, I is the intensity with the quencher, and τ_0 is the excited state lifetime of the photocatalyst.

The following stock solutions were prepared in distilled acetonitrile and degassed by three freeze-pump-thaw cycles.

General procedure:

A stock solution of 4CzIPN was prepared by dissolving 4CzIPN (25 µmol) in 10 mL of acetonitrile. Of this solution, 0.1 mL were further diluted with acetonitrile to give a total volume of 10 mL. Concentration of [4CzIPN] = 2.5 x 10^{-5} M. A stock solution of 4-cyano-1-(trifluoromethoxy)pyridin-1-ium bis((trifluoromethyl)sulfonyl)amide **2a** was prepared by dissolving **2a** (30 µmol) in 10 mL of acetonitrile. Concentration of [**2a**] = 3 x 10-3 M. A stock solution of tert-butyl (1-(4-fluorophenyl)vinyl) carbonate **1b** was prepared by dissolving **1b** (30 µmol) in 10 mL of acetonitrile. Concentration of [**1b**] = 3 x 10-3 M. For each experiment, 6 samples were prepared in the dark. Quartz cuvettes (3.5 mL) were filled with photocatalyst stock solution (0.3 mL), reagent stock solution (0 mL, 0.2 mL, 0.4mL, 0.6 mL, 0.8 mL, 1.0 mL) and acetonitrile (2.7 mL, 2.5 mL, 2.3 mL, 2.1 mL, 1.9 mL, 1.7mL) to obtain a total volume of 3 mL. The final concentrations were [4CzIPN] = 2.5 10^{-6} M and [quencher] = 2 x 10^{-4} M, 4 x 10^{-4} M, 6 x 10^{-4} M, 8 x 10^{-4} M, 1 x 10^{-3} M. For each sample, emission spectra were acquired between 470 nm and 700 nm (excitation at 450 nm).

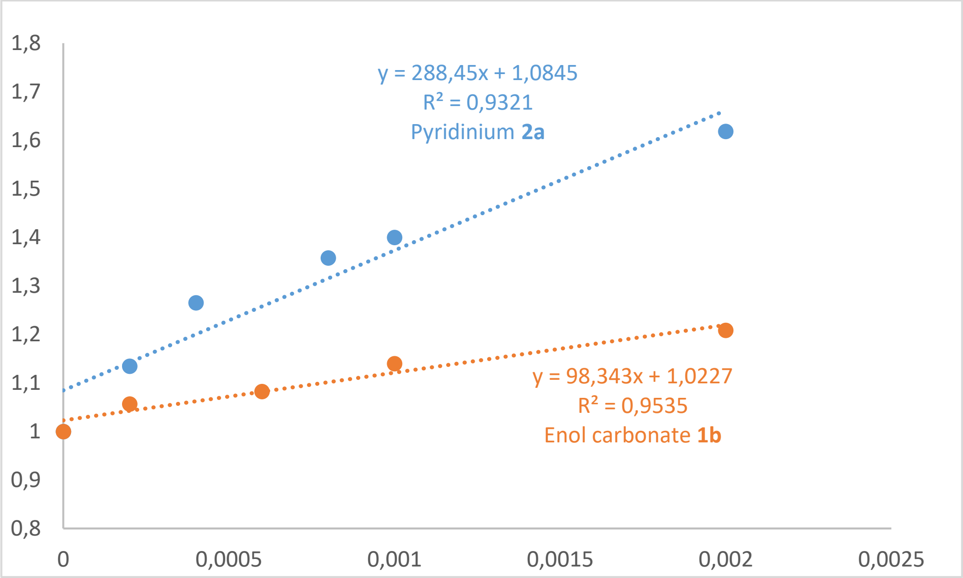

*Figure S4 Stern-Volmer plots for pyridinium **2a** and enol carbonate **1b** as quenchers*

k_2= 5.7*10^7 L.mol^{-1}.s^{-1} **k_1 = 1.9*10^7 L.mol^{-1}.s^{-1}** ($\tau_{0=}$ 5.1 μs for 4CzIPN)[16]

Quantum yield measurements

A ferrioxalate actinometry solution was prepared by following the Hammond variation of the Hatchard and Parker procedure outlined in Handbook of Photochemistry.[17] Ferrioxalate actinometer solution measures the decomposition of ferric ions to ferrous ions, which are complexed by 1,10-phenanthroline and monitored by UV/Vis absorbance at 510 nm. The moles of iron-phenanthroline complex formed are related to moles of photons absorbed. The solutions were prepared and stored in the dark:

1. Potassium ferrioxalate solution: 589.5 mg of potassium ferrioxalate (commercially available from Sigma Aldrich) and 278 μL of sulfuric acid (96%) were added to a 100 mL volumetric flask and filled to the mark with water (MilliQ grade).

2. Phenantroline solution: 0.2% by weight of 1,10-phenanthroline in water (200 mg in 100 mL volumetric flask).

3. Buffer solution: to a 100 mL volumetric flask 4.94 g of NaOAc and 1 mL of sulfuric acid (96%) were added and filled to the mark with water (MilliQ grade).

The actinometry measurements were done as follows:

1a. 456 nm LED: 1 mL of the actinometer solution was added to a quartz cuvette (l = 10 mm). The actinometry solutions (placed 1 cm away from a 456 nm LED) were irradiated for specified time intervals (0, 15, 30, 45, 60) seconds.

1b. 400 nm LED: 1 mL of the actinometer solution was added to a quartz cuvette (l = 10 mm). The actinometry solutions (placed 2 cm away from a 3W 400 nm LED) were irradiated for specified time intervals (0, 15, 20, 30, 40) seconds.

2. After irradiation all the actinometer solution was removed and placed in a 10 mL volumetric flask. 0.5mL of 1,10-phenanthroline solution and 2 mL of buffer solution was added to this flask and filled to the mark with water (MilliQ grade).

3. The UV-Vis spectra of actinometry samples were recorded for each time interval (Figure S5 and Figure S6). The absorbance of the actinometry solution was monitored at 510 nm.

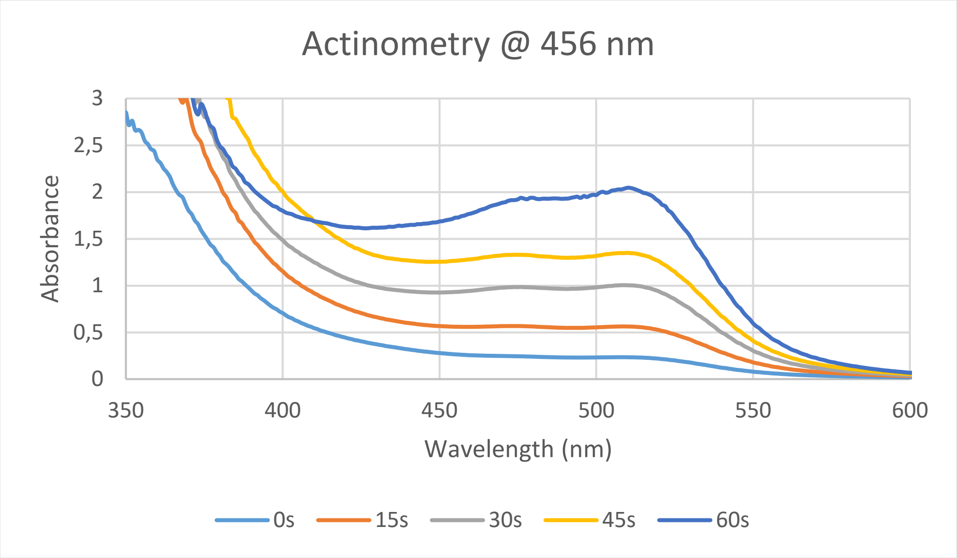

Figure S5 UV-Vis spectra of actinometry samples irradiated with 456 nm light for the indicated time intervals

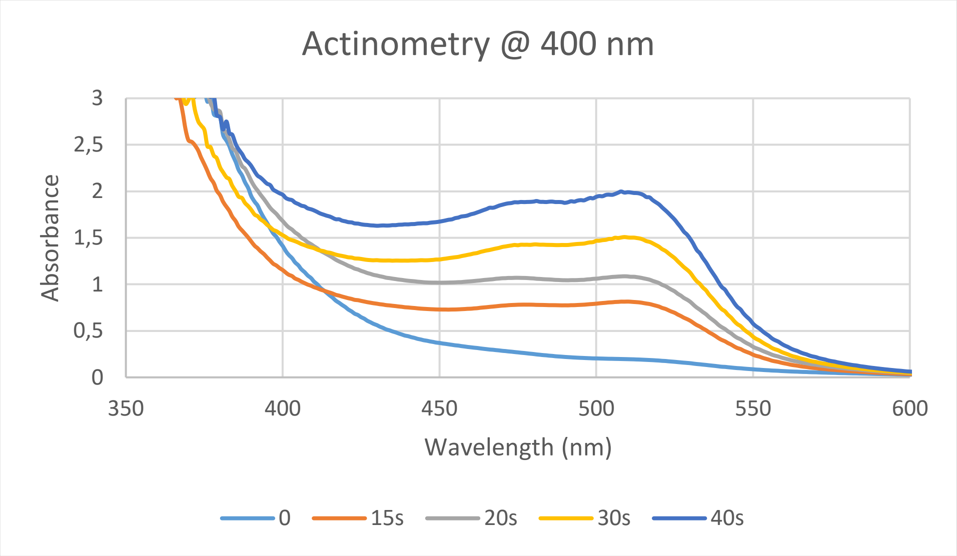

Figure S6 UV-Vis spectra of actinometry samples irradiated with 400 nm light for the indicated time intervals

4.The moles of Fe^{2+} formed for each sample are determined using Beers' Law:

$$Moles\ Fe^{2+} = \frac{V_1 V_3 \Delta A_{(510\ nm)}}{10^3 V_2 l \varepsilon_{(510\ nm)}}$$

where V_1 is the irradiated volume (1 mL), V_2 is the aliquot of the irradiated solution taken for the determination of the ferrous ions (1 mL), V_3 is the final volume after complexation with phenanthroline (10 mL), l is the optical path-length of the irradiation cell (1 cm), $\Delta A_{(510\ nm)}$ the optical difference in absorbance between the irradiated solution and that taken in the dark, $\varepsilon_{(510\ nm)}$ is the molar extinction coefficient of the complex $Fe(phen)_3^{2+}$ (11100 L mol^{-1} cm^{-1}).

5. The moles of Fe^{2+} formed (N) are plotted as a function of time (t) (Figure S7 and Figure S8). The slope is a product of the photon flux (F) and the quantum yield for Fe^{2+}($\phi(Fe^{2+}) = 1.13$), since $F = N/\phi(Fe^{2+})t$.

456 nm: The $F_{456\ nm}$ was determined to be $2.20 \cdot 10^{-8}$ einstein s^{-1}.

400 nm: The $F_{400\ nm}$ was determined to be $3.52 \cdot 10^{-8}$ einstein s^{-1}.

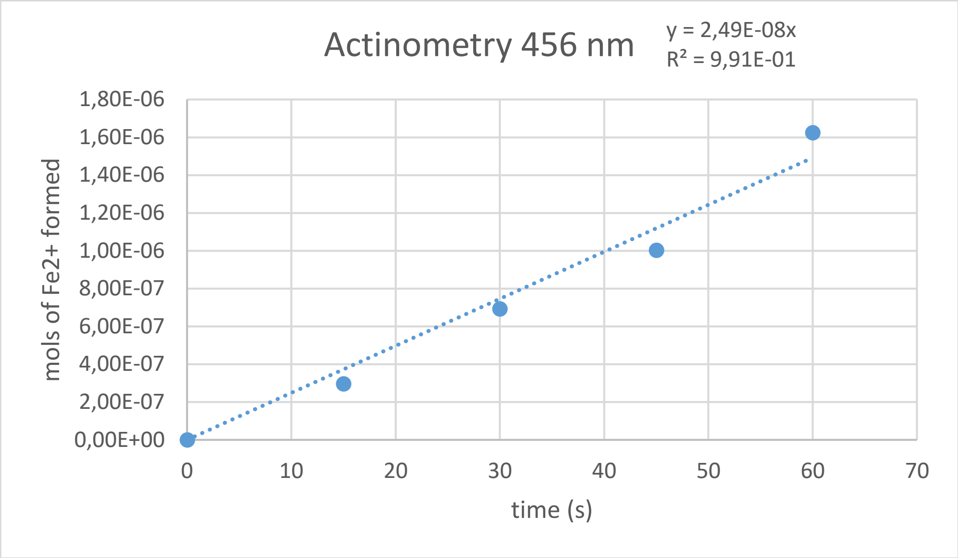

Figure S7 Moles of Fe^{2+} formed after irradiation with 456 nm light as a function of time

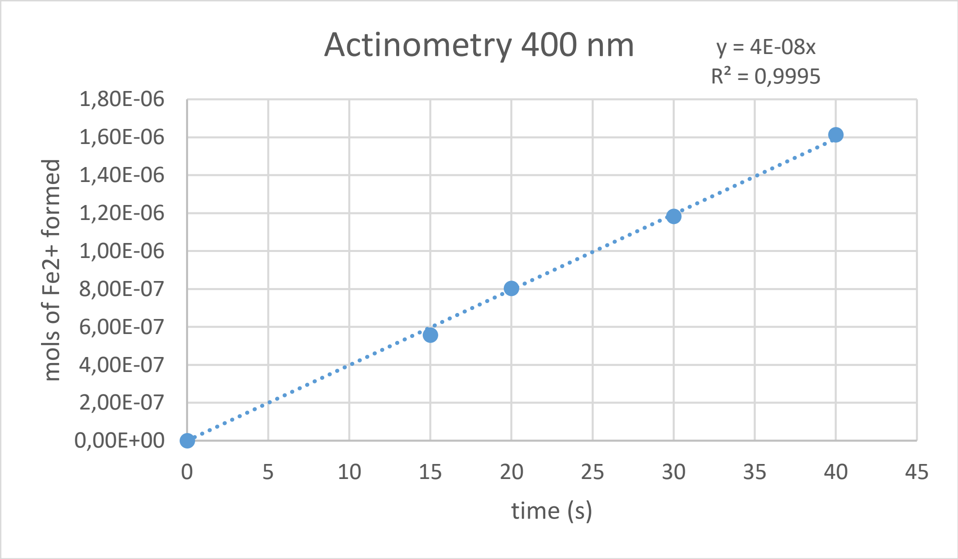

Figure S8 Moles of Fe^{2+} formed after irradiation with 400 nm light as a function of time

Since the ferrioxalate actinometer absorbance at 456 nm is 0.12 and at 400 nm is 0.47 and inferior to 2 in both cases (if is major than 2 at the wavelength used, it can be assumed that the entire incident light is absorbed), a correction factor (C) based on the fraction of light absorbed by the actinometer has to be considered to calculate the photon flux and the final quantum yield of the reaction (note that this is also needed if the absorbance of the reaction under study at the optimized concentration is inferior to 2 at the wavelength used). Thus, according to the definition of quantum yield:[18]

$$\Phi_{Fe2+} = \frac{(\frac{\partial_{moles\ Fe2+}}{\partial_{time}})}{F\ C_{Fe2+}} \rightarrow F = \frac{(\frac{\partial_{moles\ Fe2+}}{\partial_{time}})}{\Phi_{Fe2+}\ C_{Fe2+}} \qquad C = 1 - 10^{-A(\lambda)}$$

The photon flux (F) previously found must be divided by the appropriate correction factor C.

456 nm: C = 0.24, calculated with $A_{456\ nm}$ = 0.12. Corrected photon flux: $5.33 \cdot 10^{-9}$ einstein s^{-1}

400 nm: C = 0.66, calculated with $A_{400\ nm}$ = 0.47. Corrected photon flux: $2.33 \cdot 10^{-8}$ einstein s^{-1}

6. 1 mL of the model reaction solutions were irradiated using the same systems and the moles of product formed for the reaction of interest are described below. The moles of product formed were determined by ^{19}F NMR measurements using the peak of triflimide as the internal standard. The number of moles of product per unit of time is related to the number of photons absorbed.

Alpha-trifluoromethoxylation of 1b with Pyridinium 2a and 4CzIPN as the photocatalyst

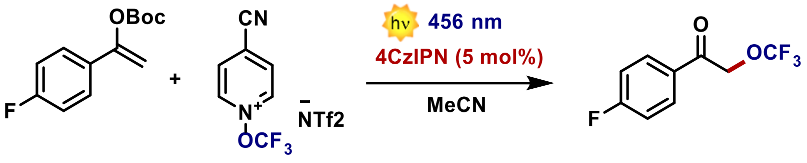

1b 2a 3

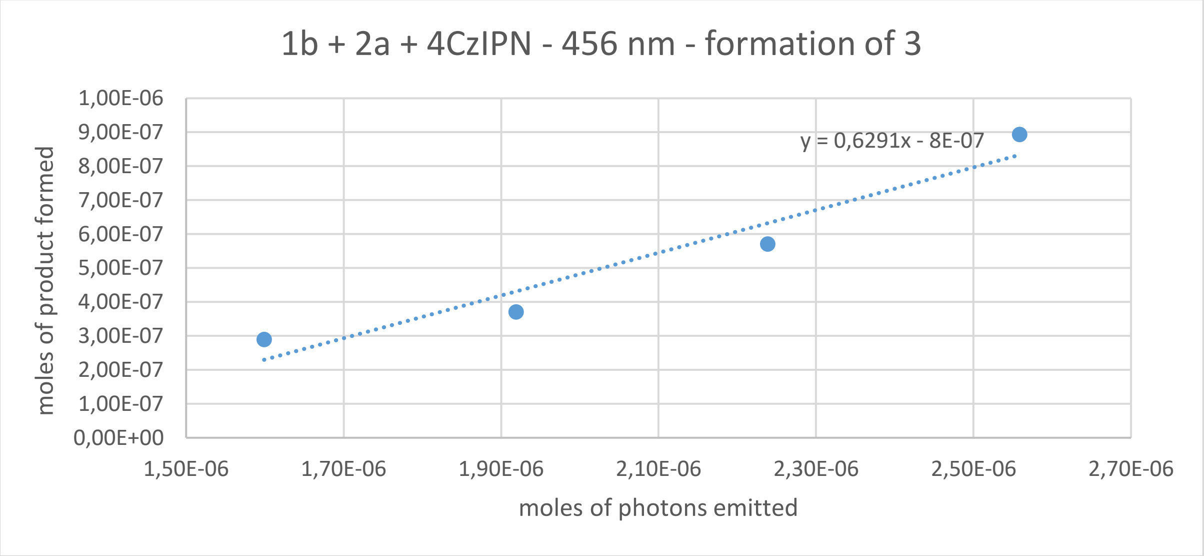

Figure S9 Moles of product formed after irradiation with 456 nm light as a function of the moles of emitted photons

The absorbance of the reaction at the irradiation wavelength (456 nm) is $A_{(456\ nm)} > 2$, hence no correction factor must be applied since it can be assumed that the entire incident light is absorbed. The quantum yield (ϕ) corresponding to the registered slope is 0.63 (Figure S9).

According to the IUPAC definition of the quantum yield,[18] we also calculated it considering the overall consumption of **2a**:

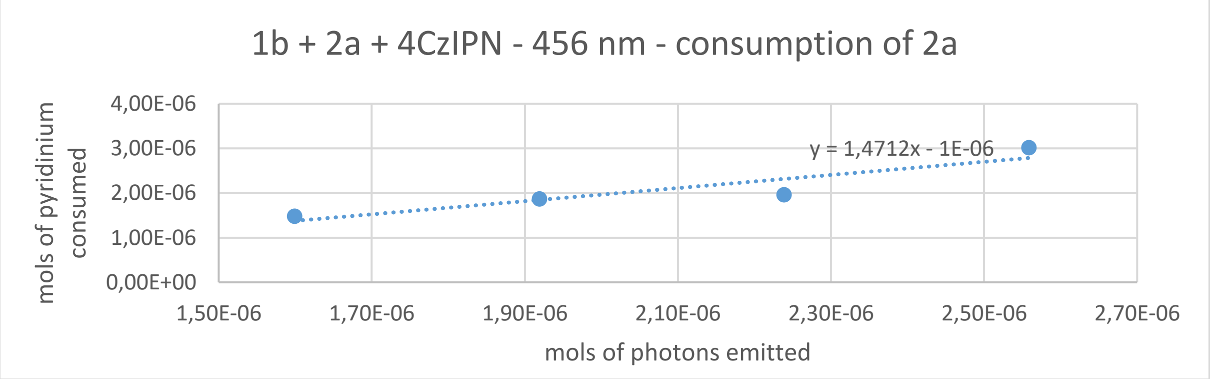

Figure S10 Moles of pyridinium consumed after irradiation with 456 nm light as a function of the moles of emitted photons

The quantum yield (ϕ) corresponding to the registered slope is 1.47 (Figure S10).

Alpha-trifluoromethoxylation of 1b with pyridinium 2a in the absence of a photocatalyst

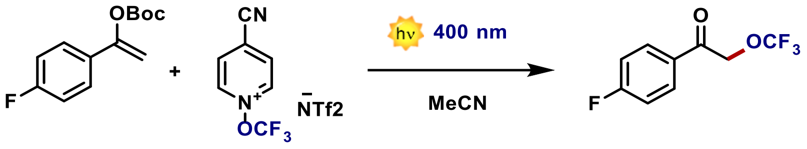

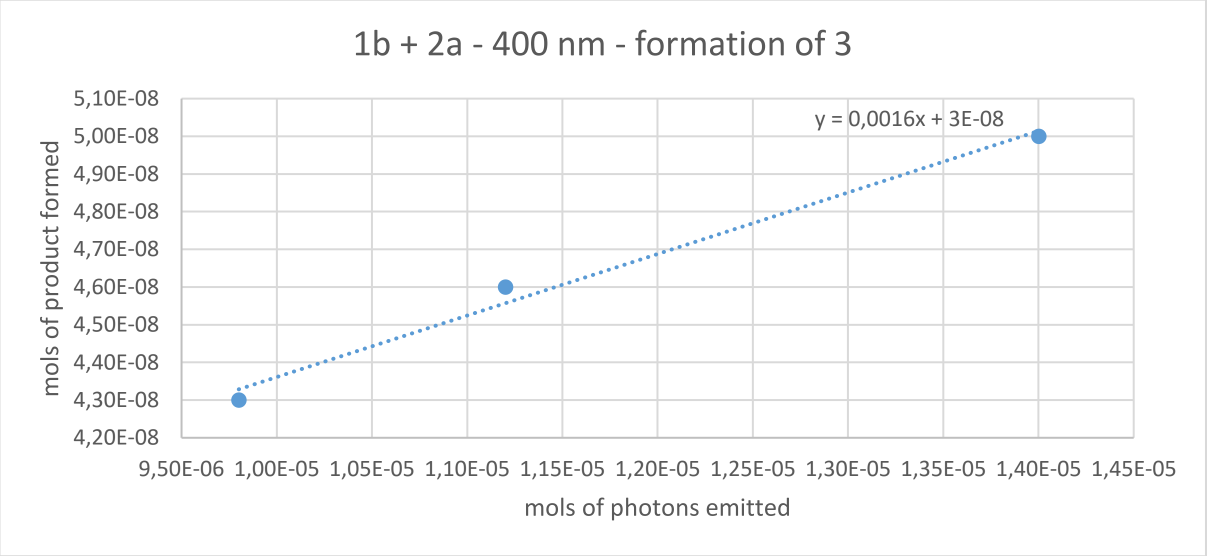

Figure S11 Moles of product formed after irradiation with 400 nm light as a function of the moles of emitted photons

The registered slope is 0.0016. The absorbance of the reaction at the irradiation wavelength (400 nm) is $A_{(400\ nm)}$ = 0.086 (Figure S11). The registered slope must be divided by the correction factor $C = 1 - 10^{-A(400\ nm)} = 0.18$ to obtain the quantum yield ϕ = 0.0089.

Alpha-trifluoromethoxylation of 1b with pyridinium 2b and 4CzIPN as the photocatalyst

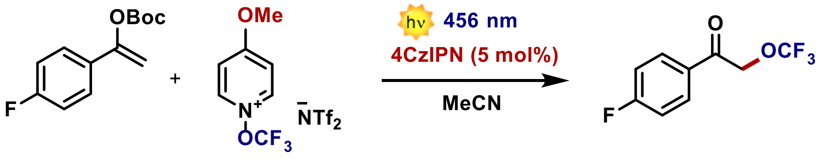

After 30 minutes of irradiation, no product **3** could be detected by ^{19}F NMR. Hence, the quantum yield can be estimated to be ϕ < 0.01.

Chain length calculation

The length L of the radical chain propagation can be calculated according to the following equation:[19]

$$L = \frac{\Phi}{Q} \qquad \text{with} \qquad Q = \frac{k_q[quencher]}{\tau_0^{-1} + k_q[quencher] + other\ processes}$$

Where τ_0 is the lifetime of the photocatalyst and k_q is the quenching rate constant obtained from Stern-Volmer analysis and "other processes" include quenching of the photocatalyst through non-productive pathways. The quenching fraction Q expresses the ratio of the rate at which the excited photocatalyst is productively quenched by **2a** to the sum of the rates of all the relaxation processes available to the excited state. Based on our mechanistic hypothesis (see main text Figure 2), assuming that i) the

quenching of 4CzIPN by **2a** is the only productive pathway for the reaction, ii) the quenching of 4CzIPN by **1b** is not productive (falling under “other processes”), and iii) considering τ_0=5.1 μs for 4CzIPN:[16]

$$L = \frac{\Phi}{Q} = \frac{0.63}{0.33} = 1.9 \qquad \text{with} \qquad Q = \frac{5.7{\cdot}10^7 M^{-1}s^{-1} \cdot 0.01\ \mathrm{M}}{(5.1{\cdot}10^{-6})^{-1} + 5.7{\cdot}10^7 M^{-1}s^{-1} \cdot 0.01\ \mathrm{M} + 1.9{\cdot}10^7 M^{-1}s^{-1} \cdot 0.05\ \mathrm{M}} = 0.33$$

The calculated length of the radical chain propagation L = 1.9, suggests that about 2 cycles of radical propagation are carried out between **2a** and intermediate **5** termination.

H. Cyclic Voltammetries

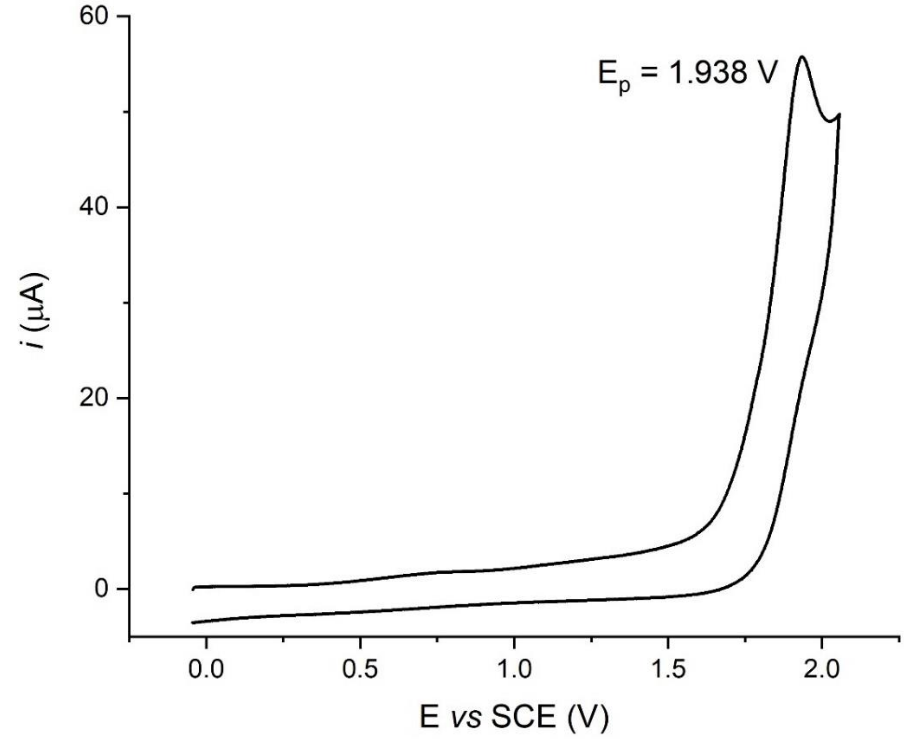

Figure S12 Cyclic Voltammogram of 1a in a supporting electrolyte 0.1 M $[Bu_4N][PF_6]$ in MeCN referenced to SCE at 0.1 Vs^{-1} sweep rate.

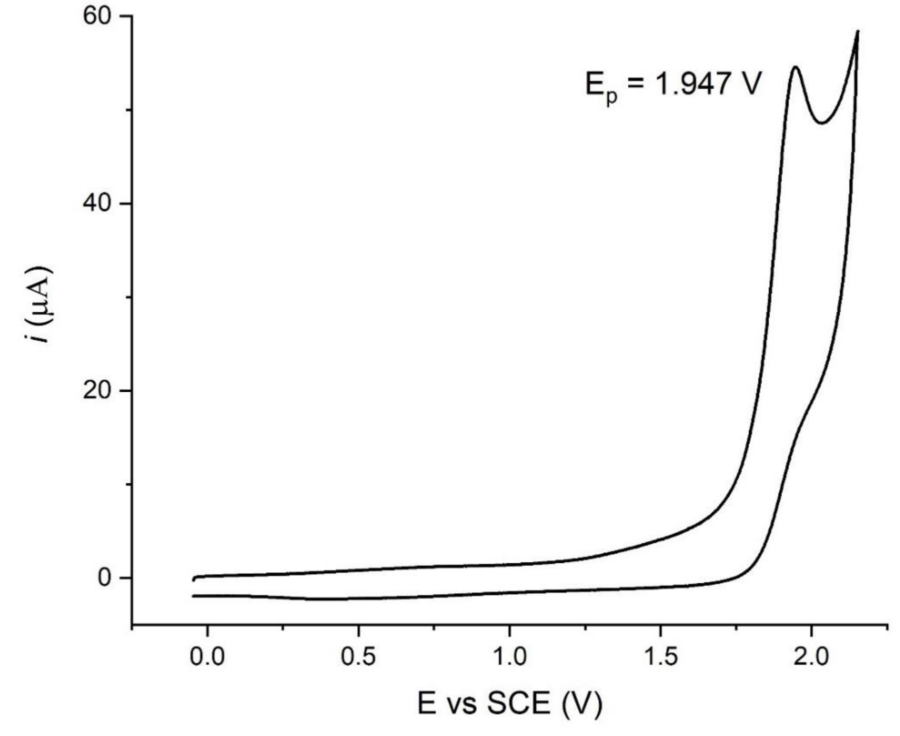

Figure S13 Cyclic Voltammogram of 1b in a supporting electrolyte 0.1 M $[Bu_4N][PF_6]$ in MeCN referenced to SCE at 0.1 Vs^{-1} sweep rate.

I. UV-Vis spectra

Figure S14 shows the UV-Vis spectra of 1a and 2a and the mixture of both components. The redshift of the absorbance in the orange trace with respect to the individual components suggests the formation of an EDA complex.

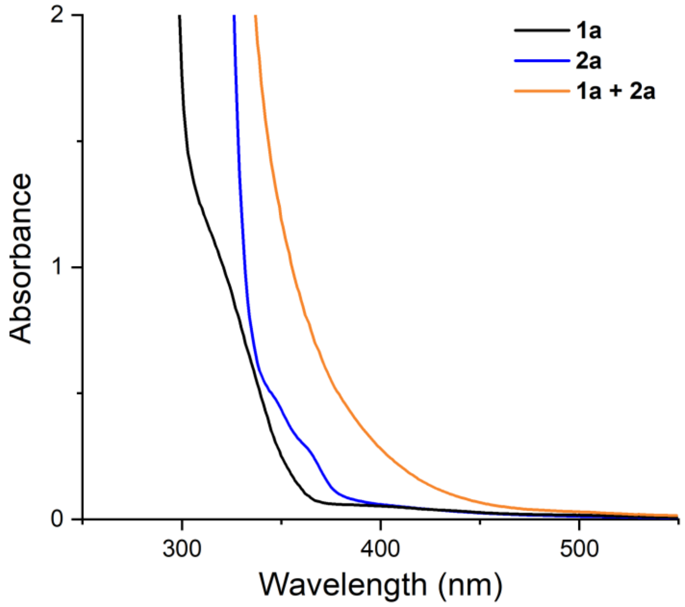

Figure S14 UV-Vis traces of enol acetate 1a, pyridinium 2a and the mixture of 1a and 2a. [1a]=0.1 M (black trace); [2a]=0.1 M (blue trace); [1a]=0.1 M and [2a]=0.1 M (orange trace).

Figure S15 shows the UV-Vis traces of 1a, 2a, mixture of 1a + 2a (5:1 equiv.) and the reaction mixture in the optimized reaction conditions ([2a]=0.01 M, 5 equiv. of enol derivative and 5 mol% of 4CzIPN).

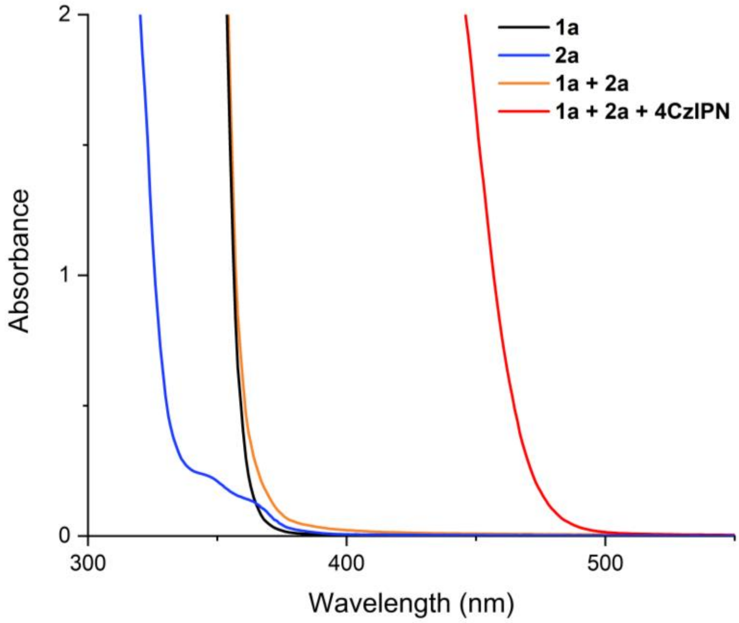

Figure S15 UV-Vis traces of enol acetate 1a, pyridinium 2a, mixture of 1a and 2a and the mixture of 1a and 2a with 5 mol% of 4CzIPN. [1a]=0.05 M (black trace); [2a]=0.01 M (blue trace); [1a]=0.05 M and [2a]=0.01 M (orange trace); [1a]=0.05 M, [2a]=0.01 M and [4CzIPN]=0.0005 M (red trace).

Figure S16 shows the UV-Vis traces of 1b, 2a, mixture of 1b + 2a (5:1 equiv.) and the reaction mixture in the optimized reaction conditions ([2a]=0.01 M, 5 equiv. of enol derivative and 5 mol% of 4CzIPN).

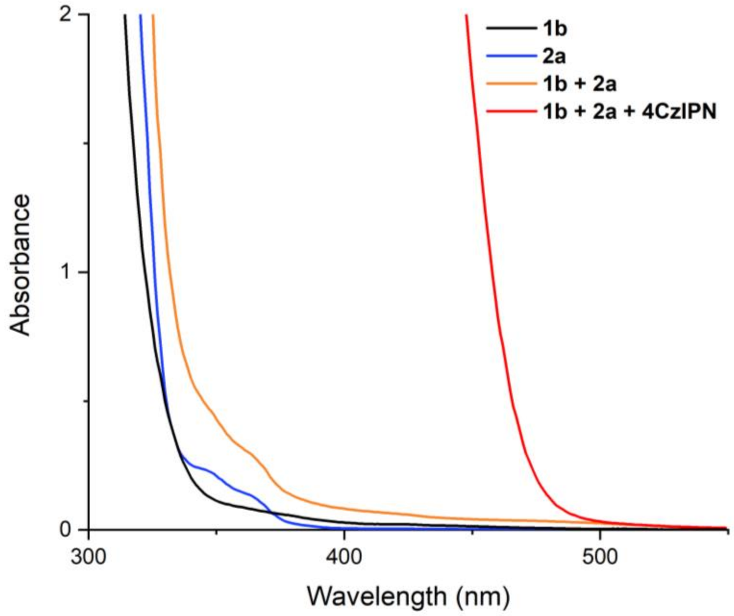

Figure S16 UV-Vis traces of enol carbonate 1b, pyridinium 2a, mixture of 1b and 2a and the mixture of 1b and 2a with 5 mol% of 4CzIPN. [1b]=0.05 M (black trace); [2a]=0.01 M (blue trace); [1b]=0.05 M and [2a]=0.01 M (orange trace); [1b]=0.05 M, [2a]=0.01 M and [4CzIPN]=0.0005 M (red trace).

J. References

[1] G. Ragazzon, C. Schäfer, P. Franchi, S. Silvi, B. Colasson, M. Lucarini, A. Credi, *Proc. Natl. Acad. Sci.* **2018**, *115*, 9385 LP – 9390.

[2] J. Mateos, F. Rigodanza, A. Vega-Peñaloza, A. Sartorel, M. Natali, T. Bortolato, G. Pelosi, X. Companyó, M. Bonchio, L. Dell'Amico, *Angew. Chemie Int. Ed.* **2020**, *59*, 1302–1312.

[3] E. Speckmeier, T. G. Fischer, K. Zeitler, *J. Am. Chem. Soc.* **2018**, *140*, 15353–15365.

[4] D. Petzold, P. Nitschke, F. Brandl, V. Scheidler, B. Dick, R. M. Gschwind, B. König, *Chem. – A Eur. J.* **2019**, *25*, 361–366.

[5] F. Houlihan, F. Bouchard, J. M. J. Fréchet, C. G. Willson, *Can. J. Chem.* **1985**, *63*, 153–162.

[6] W. Li, Y. Zhu, Y. Zhou, H. Yang, C. Zhu, *Tetrahedron* **2019**, *75*, 1647–1651.

[7] S. Sasaki, T. Suzuki, T. Uchiya, S. Toyota, A. Hirano, M. Tanemura, H. Teramoto, T. Yamauchi, K. Higashiyama, *J. Fluor. Chem.* **2016**, *192*, 78–85.

[8] A. Parrilla, I. Villuendas, A. Guerrero, *Bioorg. Med. Chem.* **1994**, *2*, 243–252.

[9] Q. Xu, H. Zhou, X. Geng, P. Chen, *Tetrahedron* **2009**, *65*, 2232–2238.

[10] Y. Hirayama, K. Okuzumi, H. Masubuti, H. Uekusa, J.-P. Girault, Y. Fujimoto, *J. Org. Chem.* **2014**, *79*, 5471–5477.

[11] R. Floresca, M. Kurihara, D. S. Watt, A. Demir, *J. Org. Chem.* **1993**, *58*, 2196–2200.

[12] L. Da Costa, E. Scheers, A. Coluccia, A. Rosetti, M. Roche, J. Neyts, T. Terme, R. Cirilli, C. Mirabelli, R. Silvestri, et al., *Eur. J. Med. Chem.* **2017**, *140*, 528–541.

[13] C. Yates, *Metalloenzyme Inhibitor Compounds*, **2018**, WO2018/165520Al.

[14] Y. A. Davydova, T. M. Sokolenko, Y. L. Yagupolskii, *J. Fluor. Chem.* **2014**, *157*, 58–62.

[15] R. Zriba, E. Magnier, J.-C. Blazejewski, *Synlett* **2009**, *2009*, 1131–1135.

[16] H. Uoyama, K. Goushi, K. Shizu, H. Nomura, C. Adachi, *Nature* **2012**, *492*, 234–238.

[17] *J. für Prakt. Chemie* **1976**, *318*, 702–703.

[18] S. E. Braslavsky, *Pure Appl. Chem.* **2007**, *79*, 293.

[19] M. A. Cismesia, T. P. Yoon, *Chem. Sci.* **2015**, *6*, DOI 10.1039/c5sc02185e.

K. NMR spectra

tert-butyl (1-(4-fluorophenyl)vinyl) carbonate 1b

^{1}H NMR (500 MHz, $CDCl_3$)

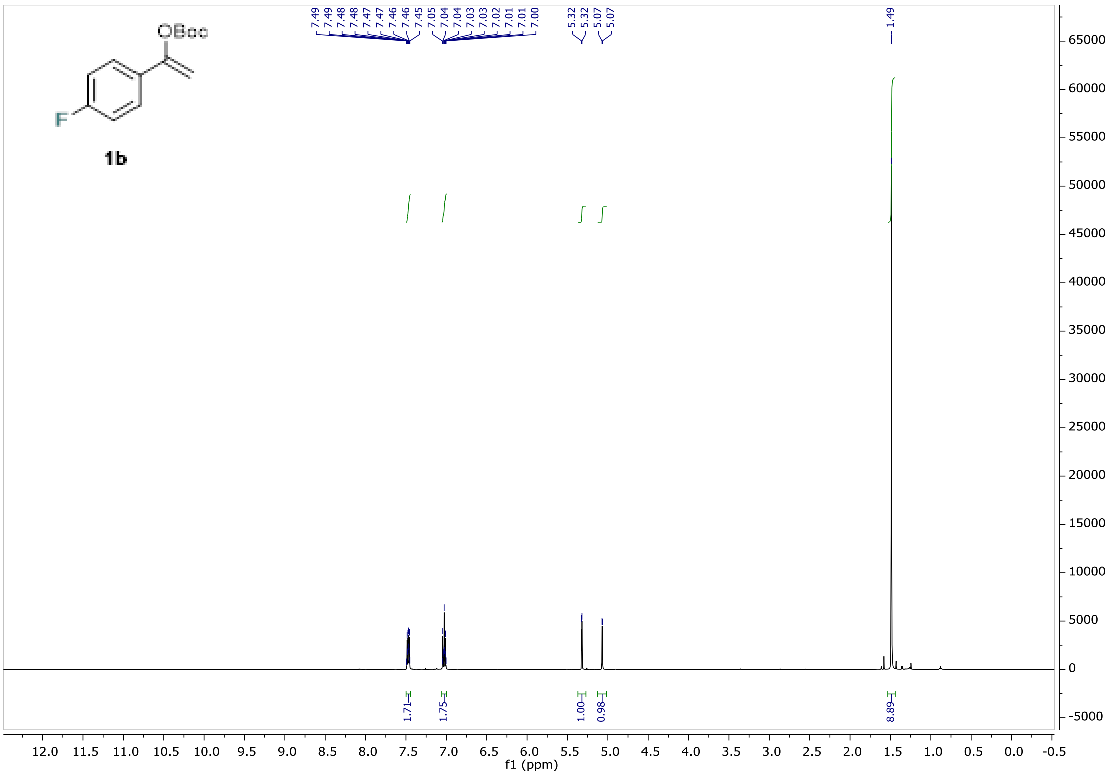

^{13}C NMR (126 MHz, $CDCl_3$)

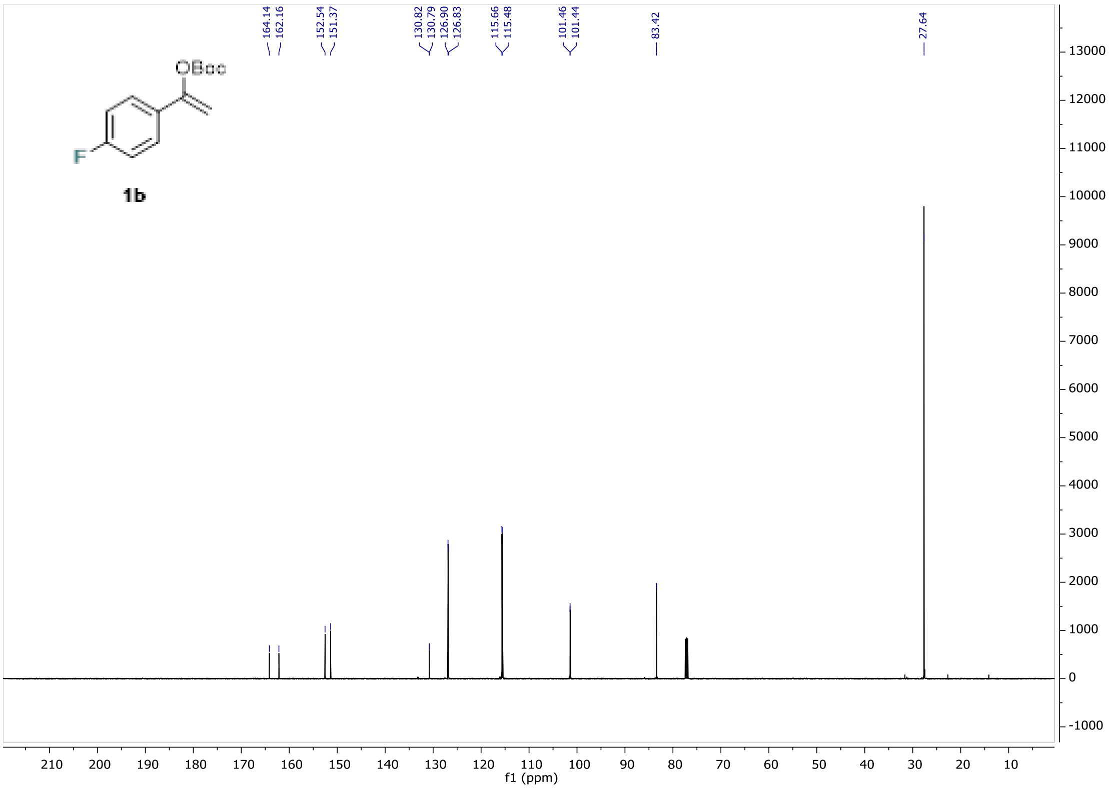

^{19}F NMR (188 MHz, ^{1}H decoupled, $CDCl_3$)

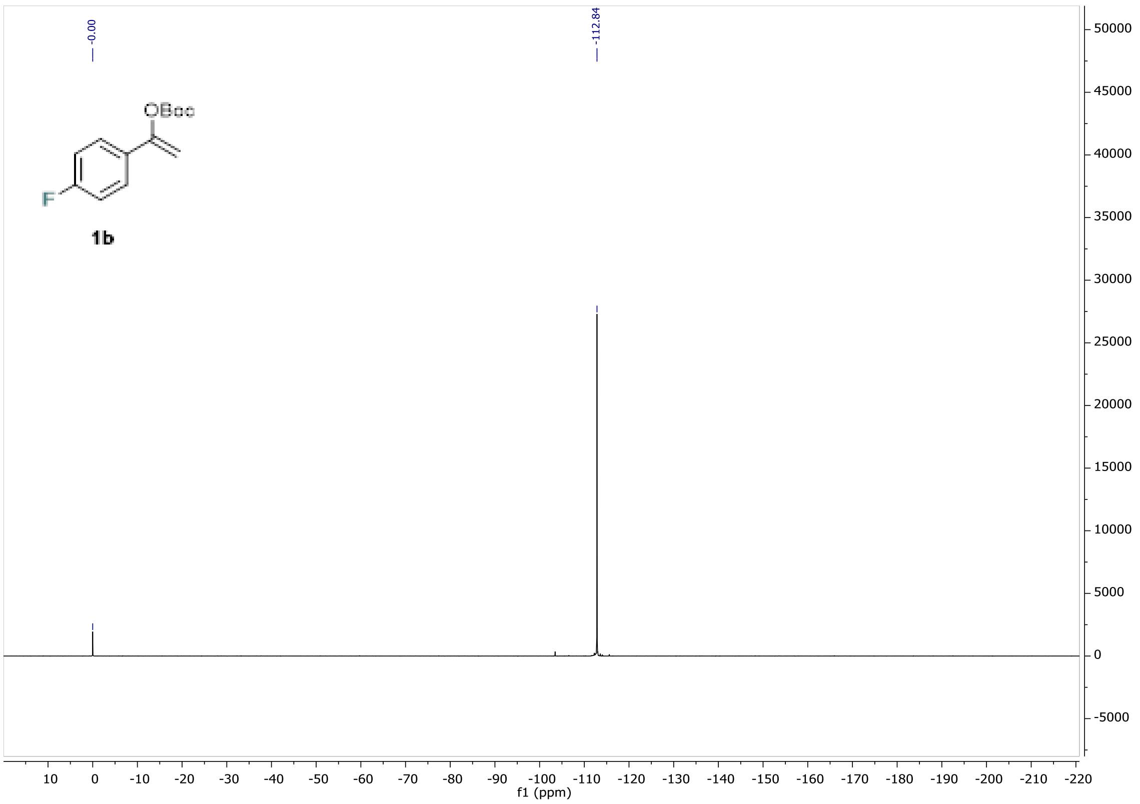

tert-butyl (1-(o-tolyl)vinyl) carbonate 1d

^{1}H NMR (300 MHz, $CDCl_3$)

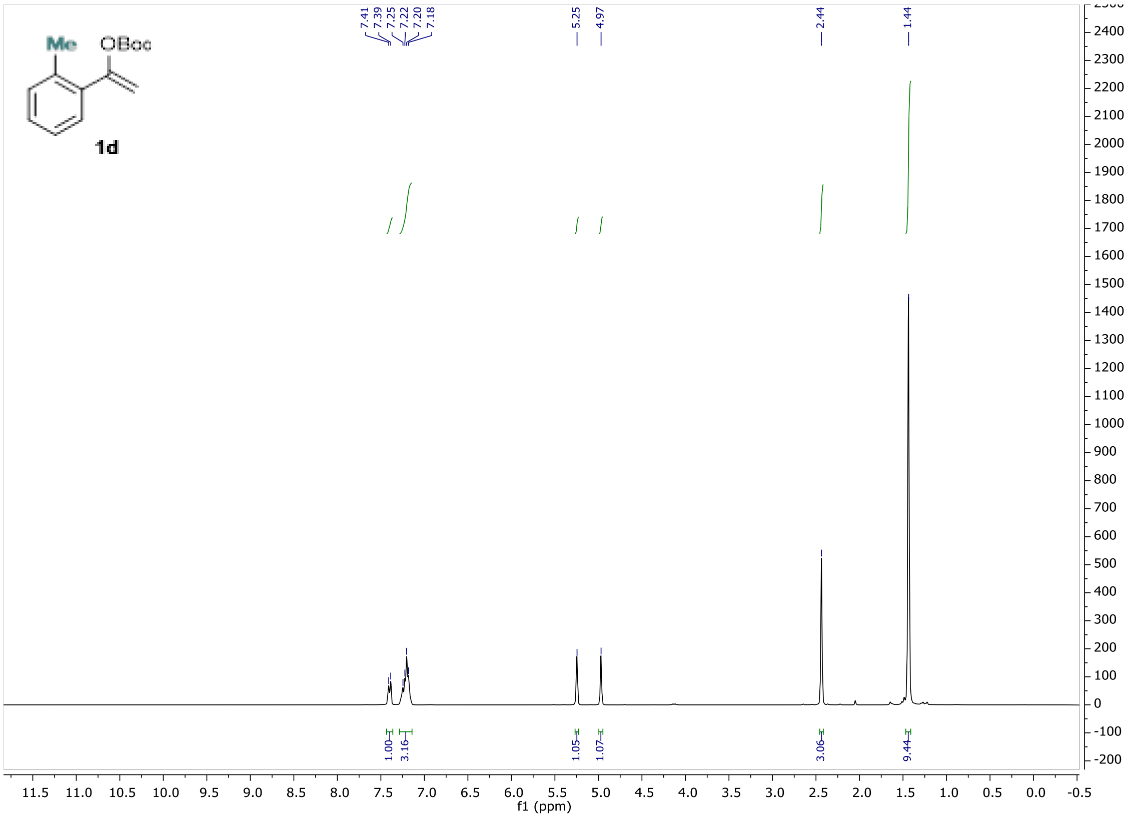

^{13}C NMR (75 MHz, $CDCl_3$)

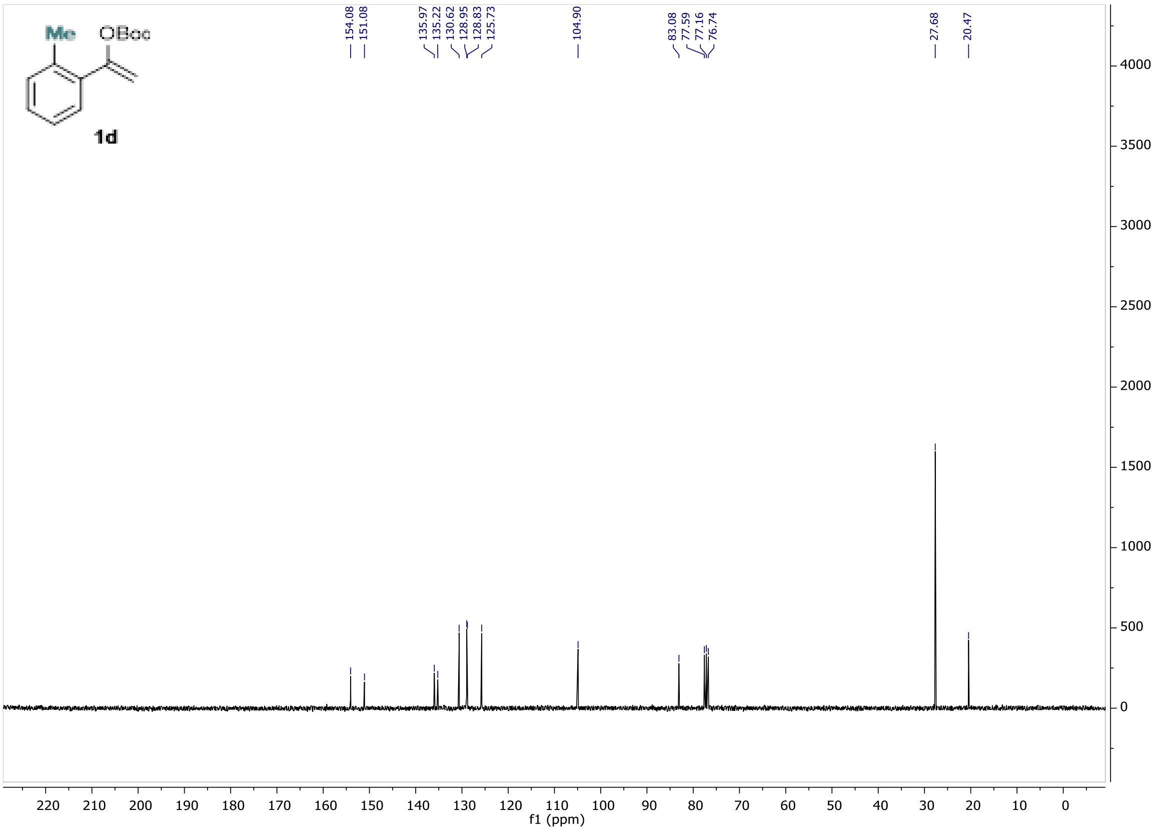

tert-butyl (1-(m-tolyl)vinyl) carbonate 1e

^{1}H NMR (300 MHz, $CDCl_3$)

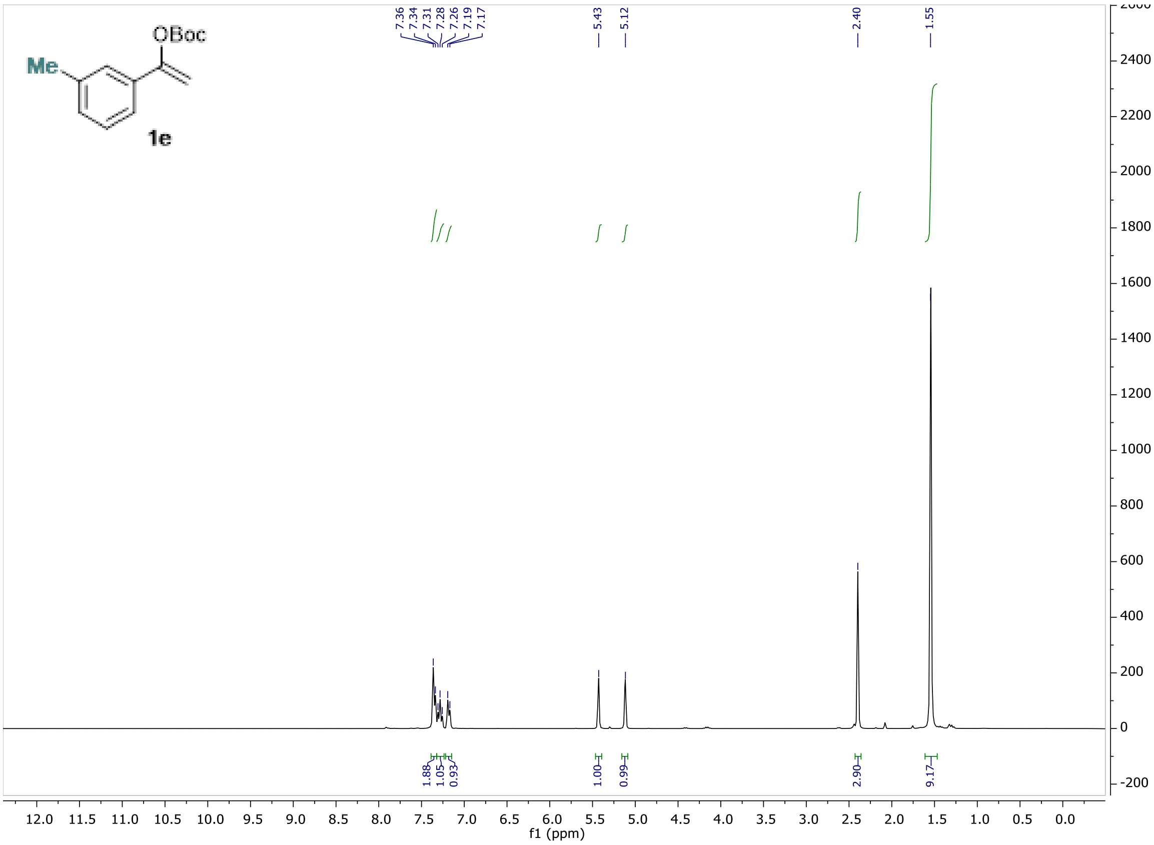

^{13}C NMR (75 MHz, $CDCl_3$)

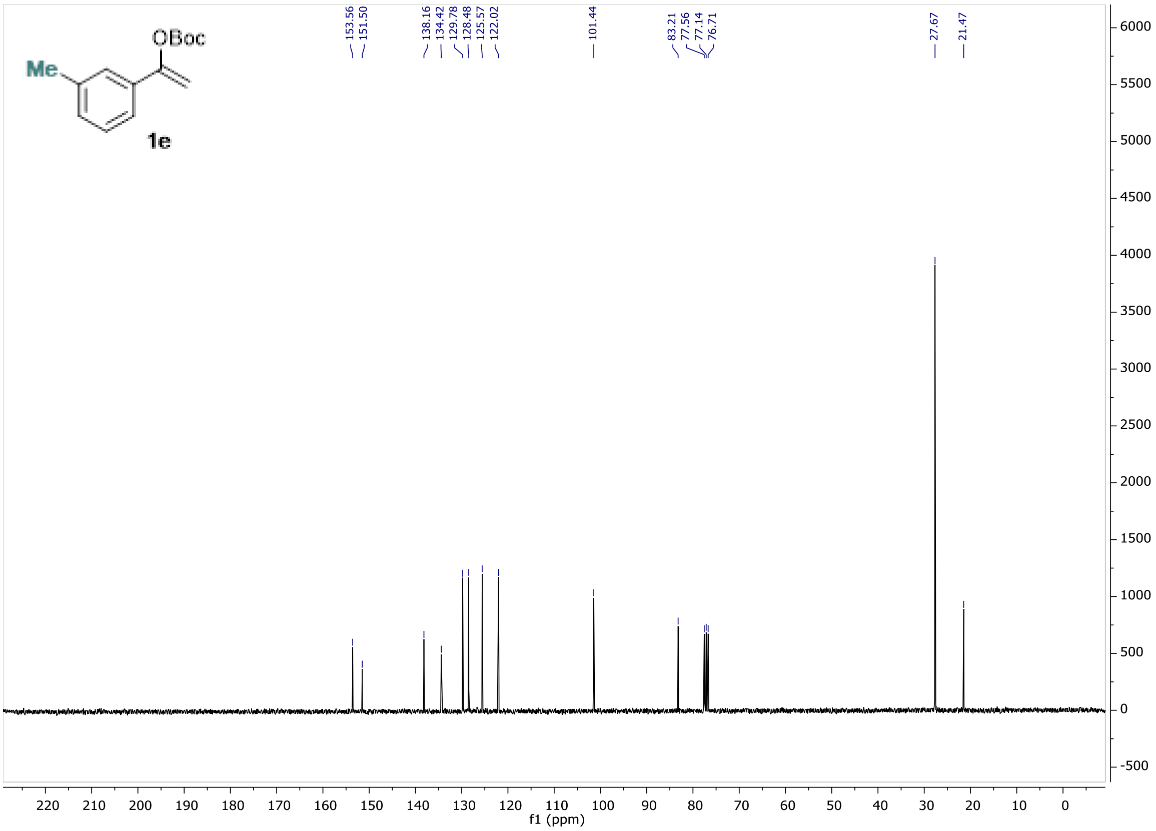

tert-butyl (1-(p-tolyl)vinyl) carbonate 1f

^{1}H NMR (300 MHz, $CDCl_3$)

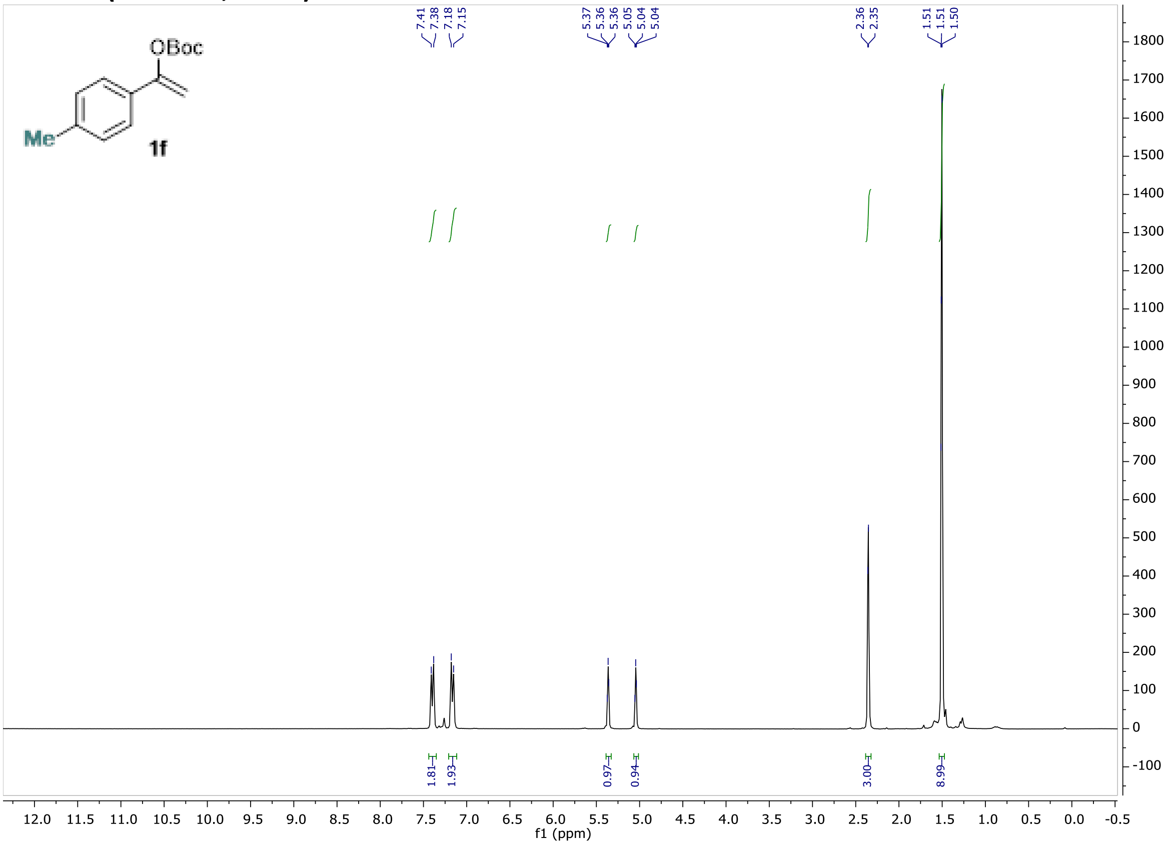

^{13}C NMR (75 MHz, $CDCl_3$)

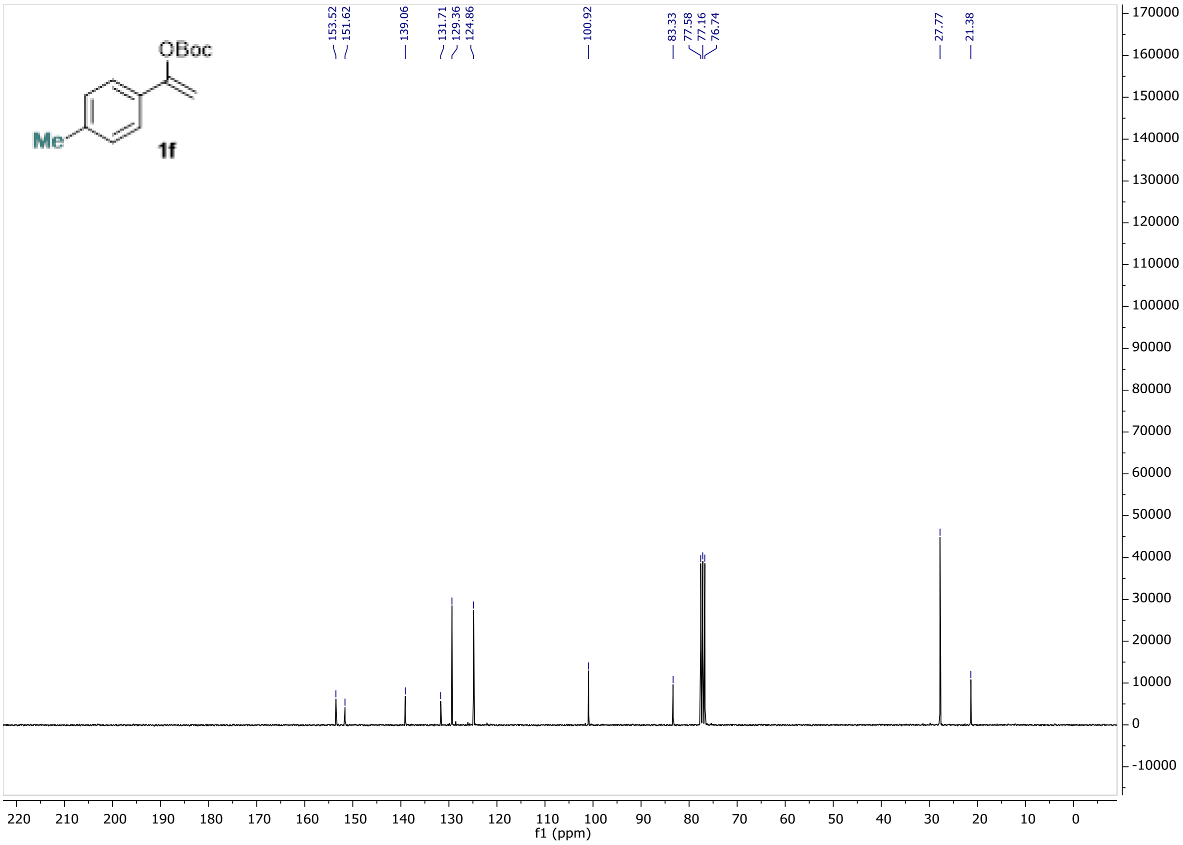

tert-butyl (1-(4-cyanophenyl)vinyl) carbonate 1g

^{1}H NMR (300 MHz, $CDCl_3$)

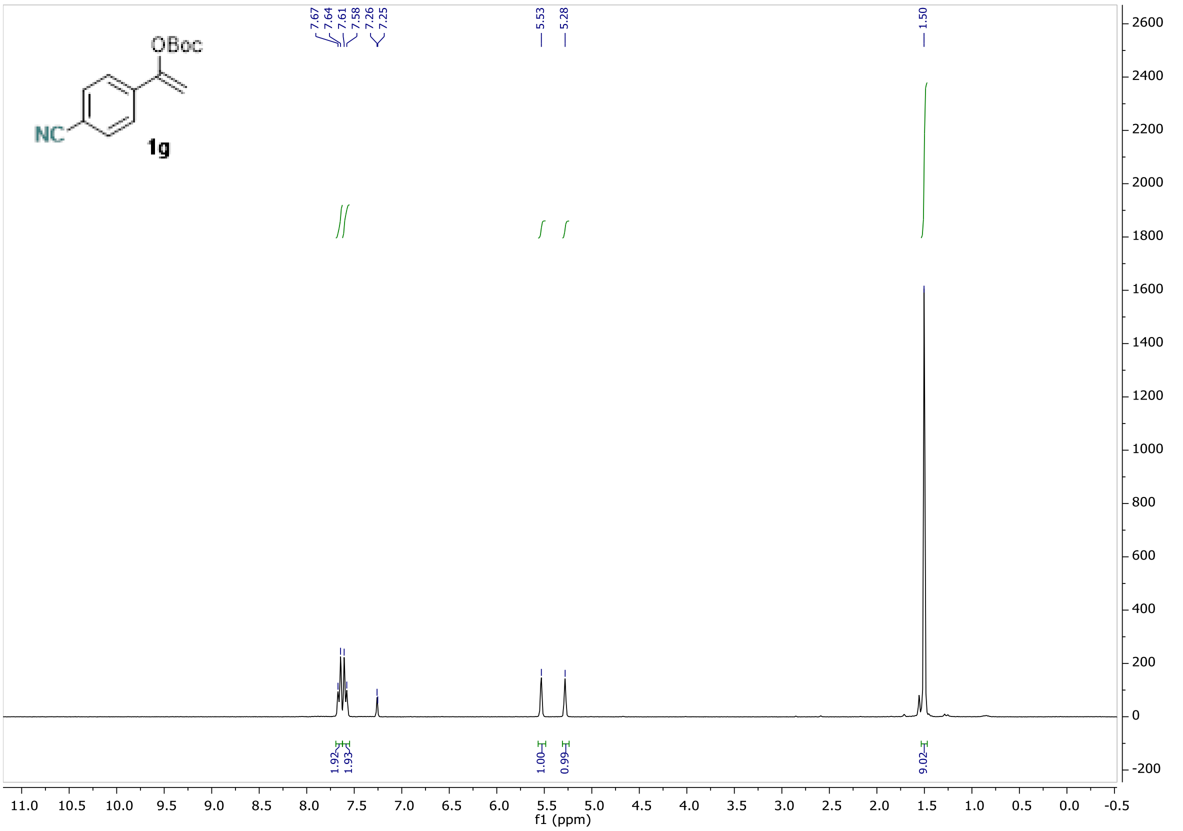

^{13}C NMR (75 MHz, $CDCl_3$)

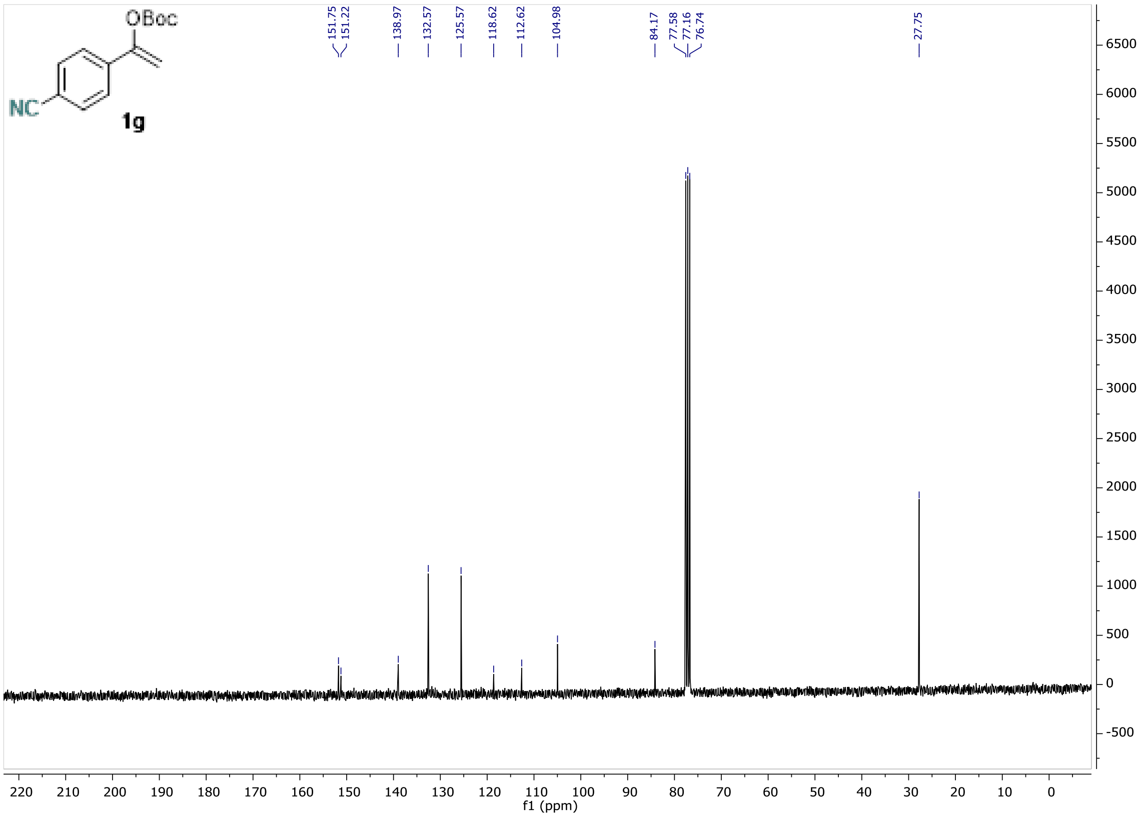

1-(4-acetylphenyl)vinyl tert-butyl carbonate 1h

^{1}H NMR (400 MHz, $CDCl_3$)

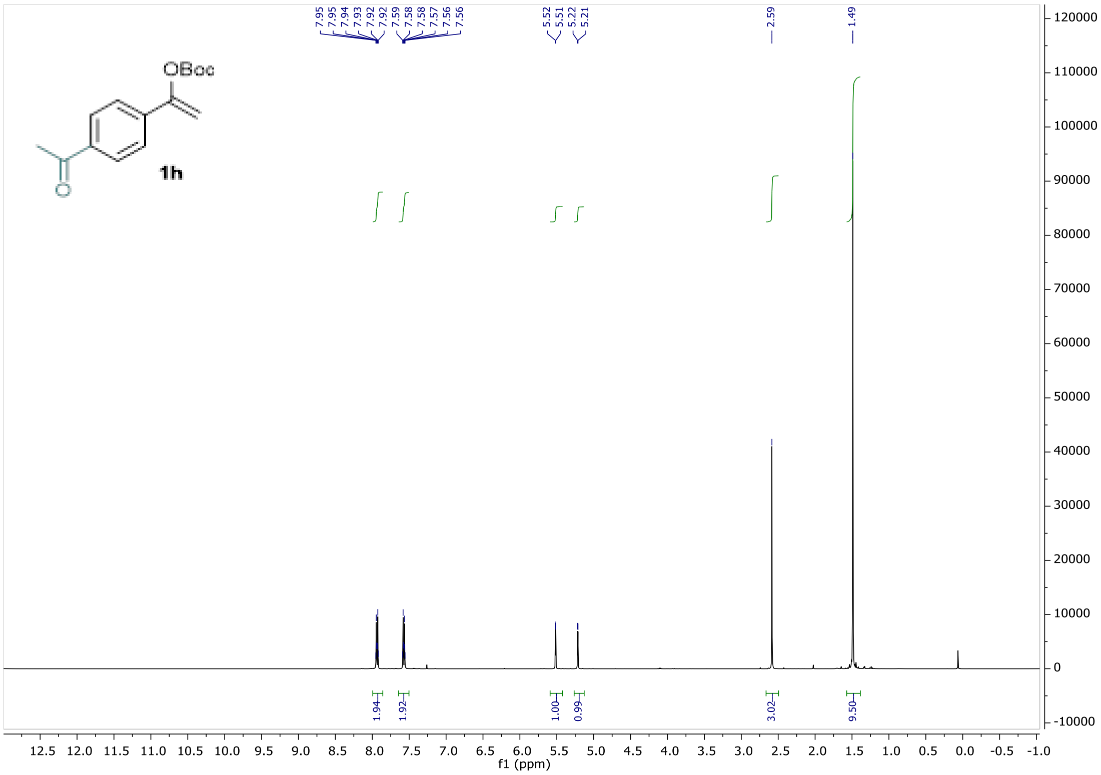

^{13}C NMR (101 MHz, $CDCl_3$)

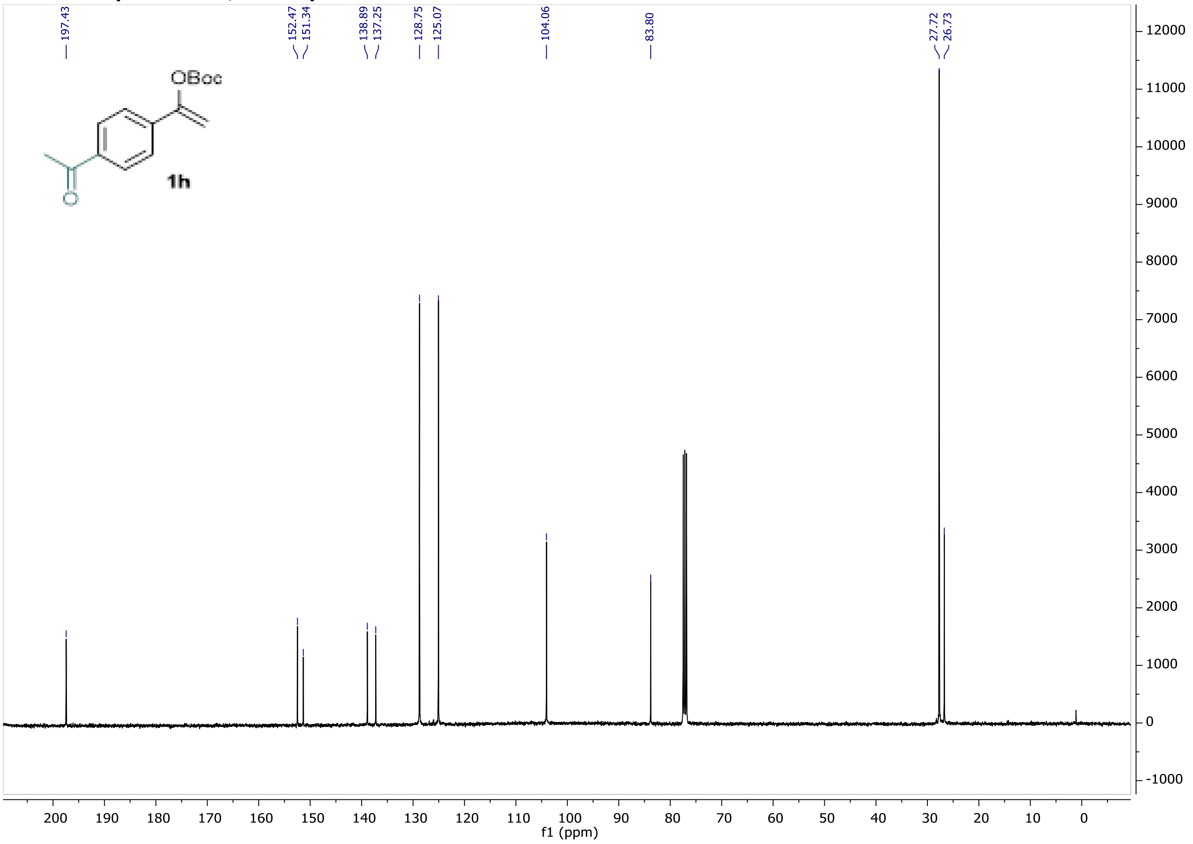

tert-butyl (1-(3-(trifluoromethyl)phenyl)vinyl) carbonate 1i

^{1}H NMR (300 MHz, $CDCl_3$)

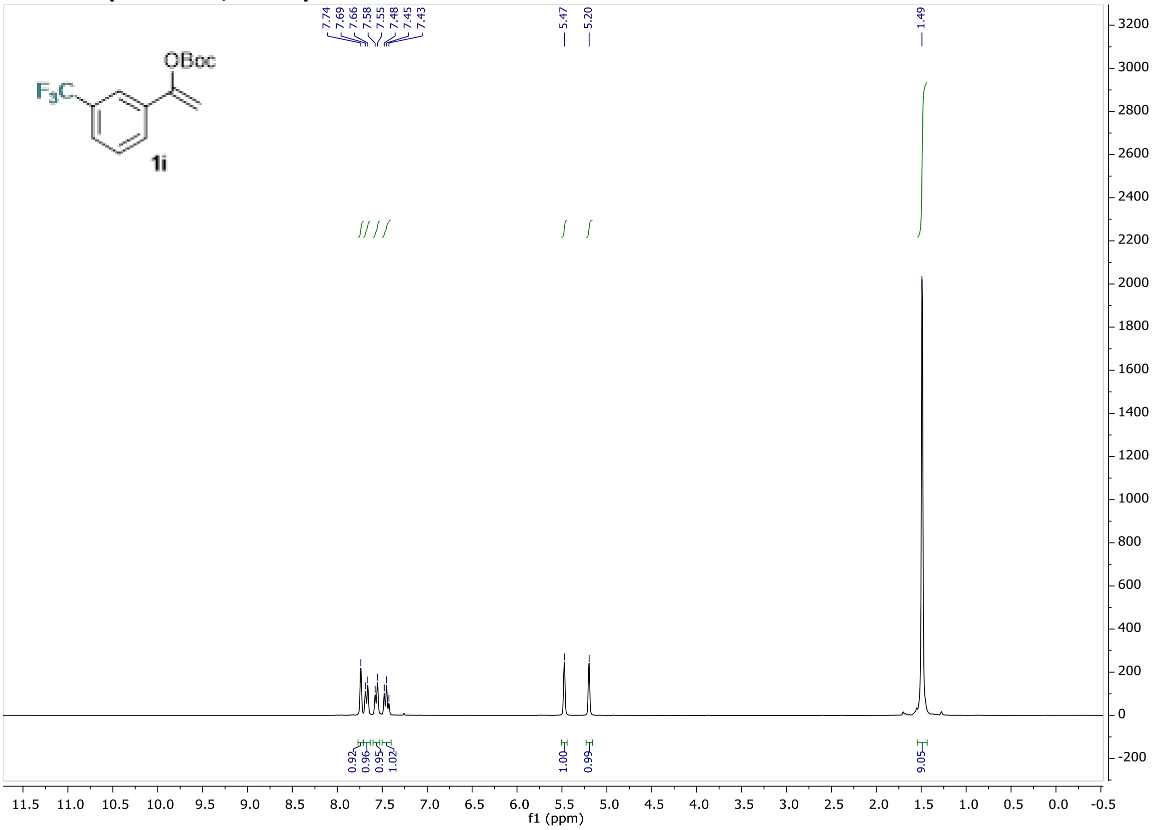

^{13}C NMR (75 MHz, $CDCl_3$)

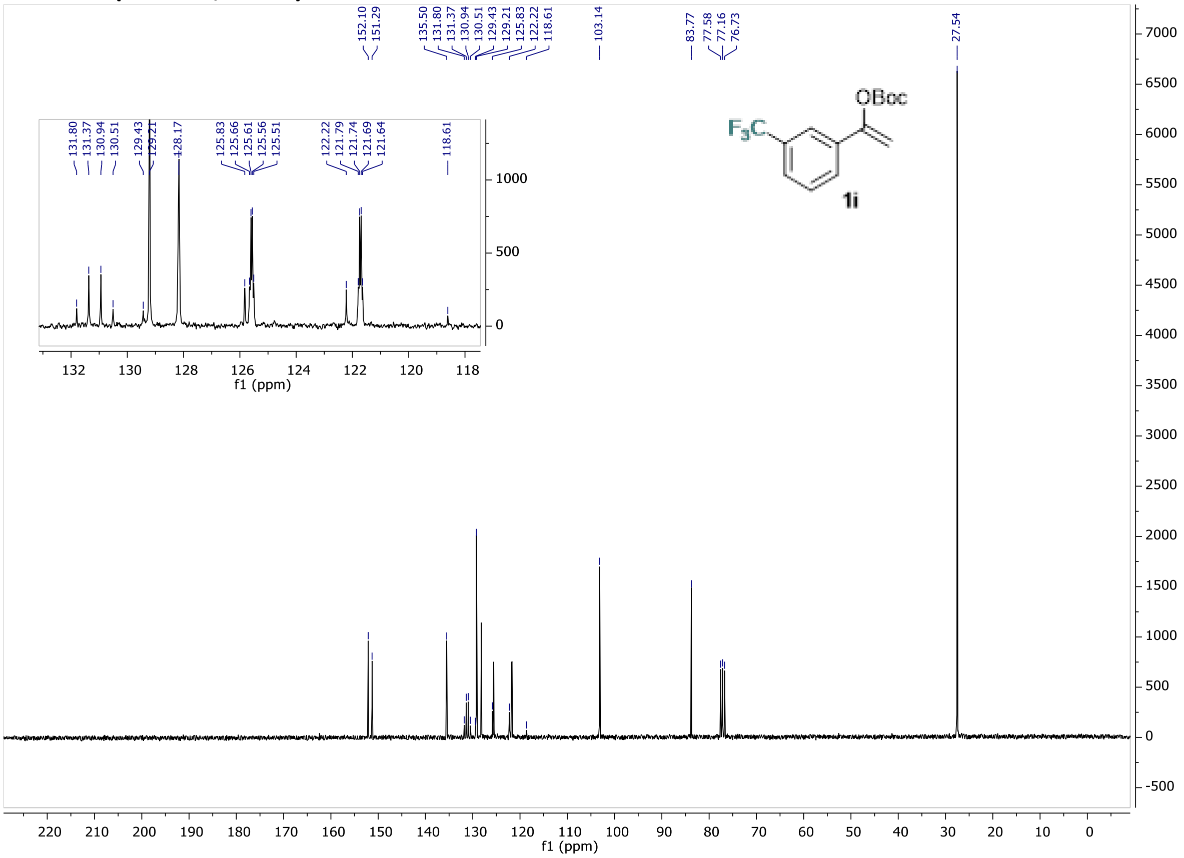

^{19}F NMR (188 MHz, $CDCl_3$)

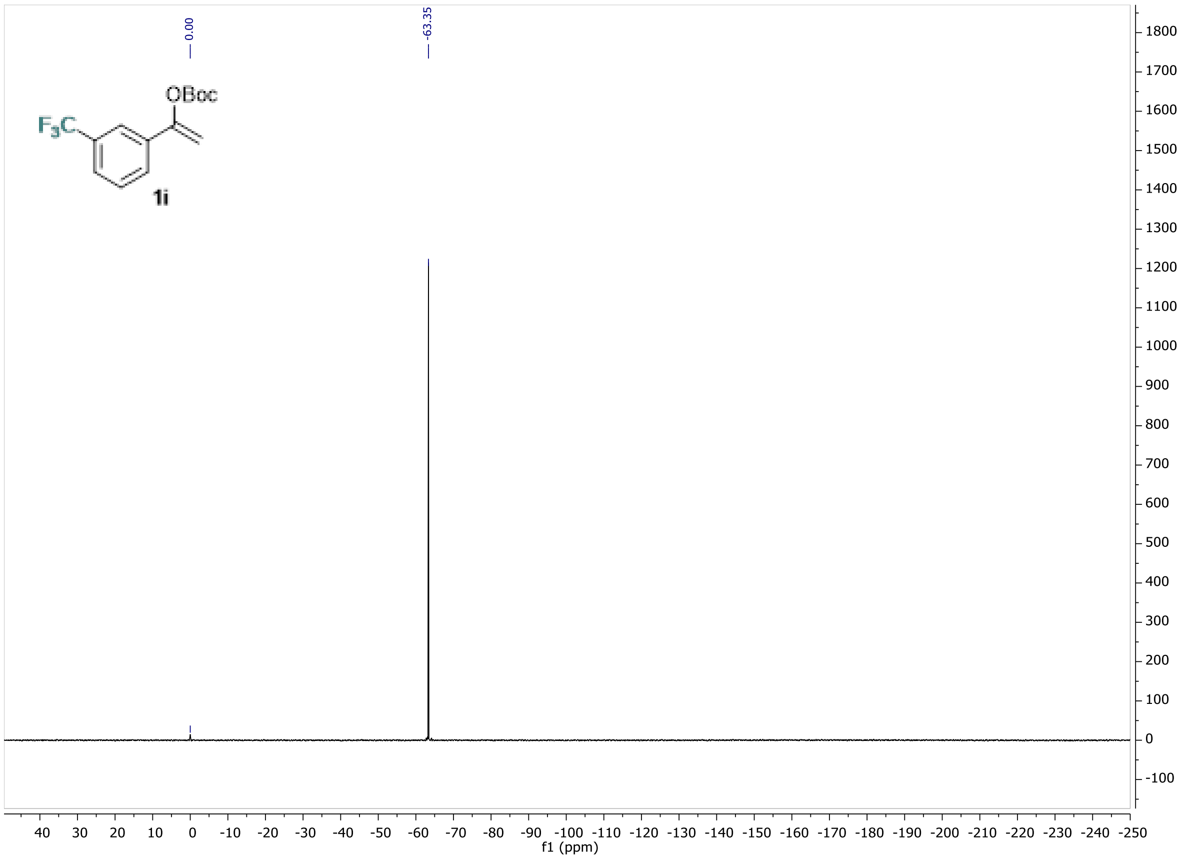

1-(3,5-bis(trifluoromethyl)phenyl)vinyl tert-butyl carbonate 1j

^{1}H NMR (400 MHz, $CDCl_3$)

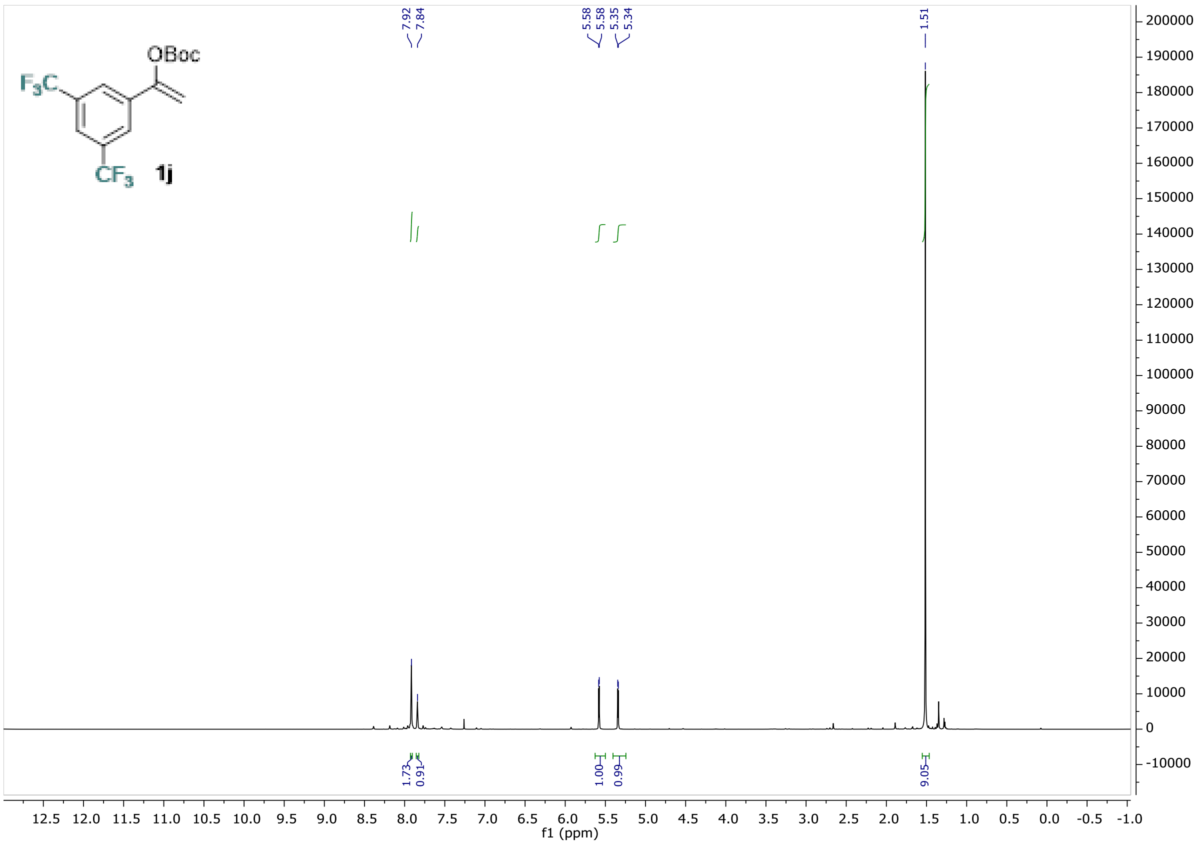

^{13}C NMR (101 MHz, $CDCl_3$)

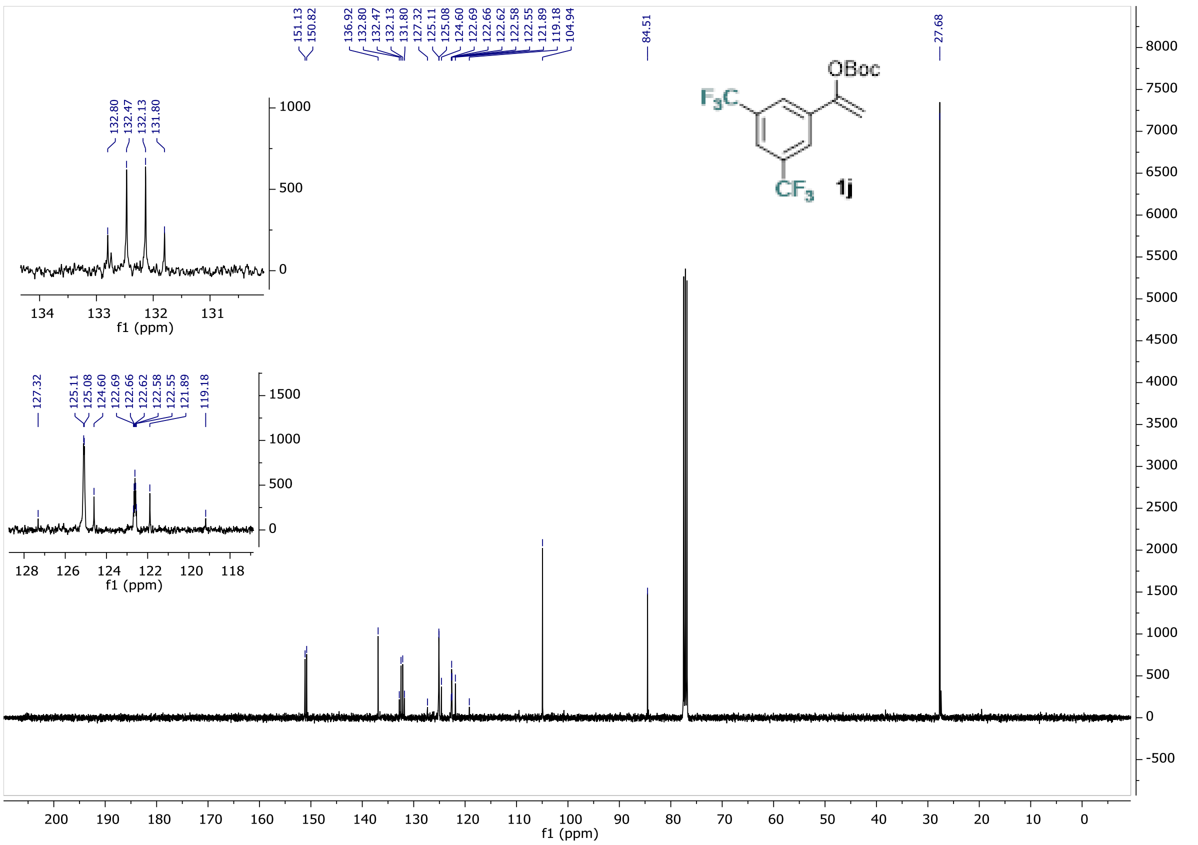

^{19}F NMR (188 MHz, ^{1}H decoupled, $CDCl_3$)

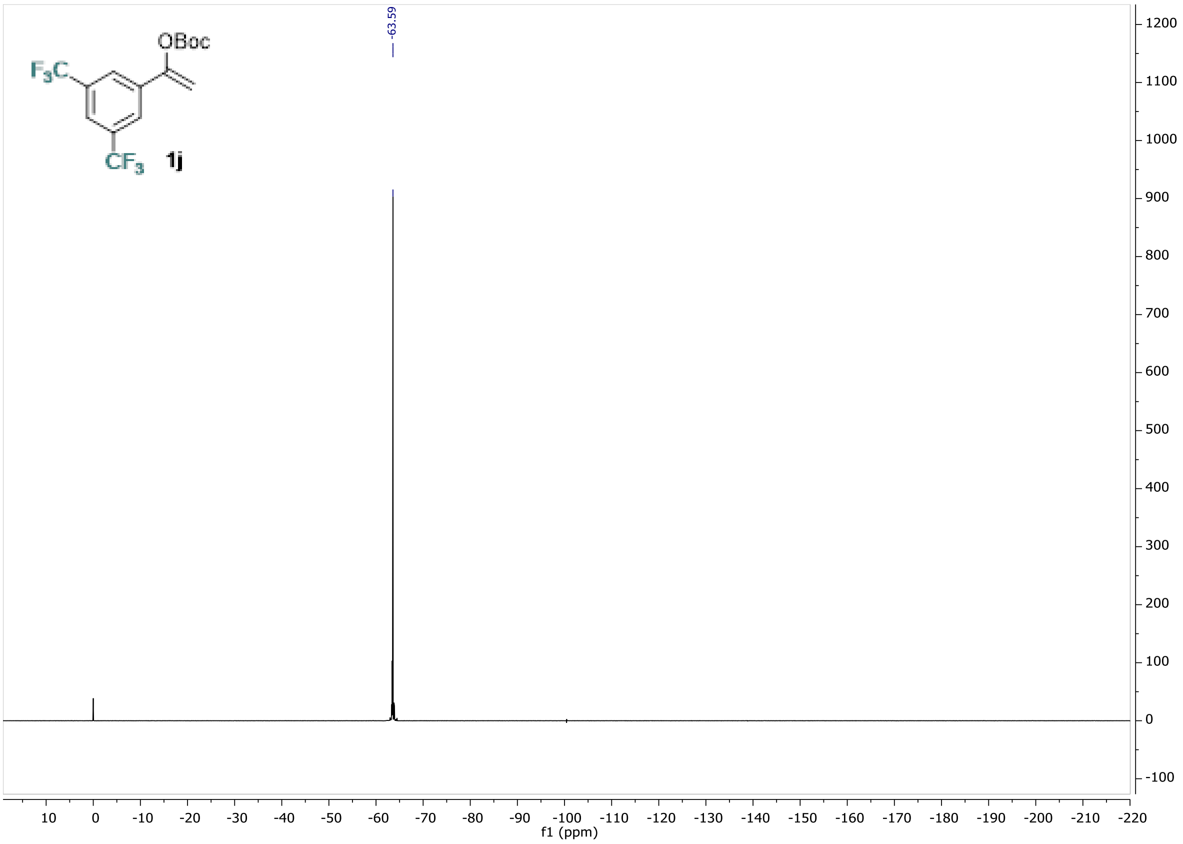

1-(2-bromophenyl)vinyl tert-butyl carbonate 1k

^{1}H NMR (500 MHz, $CDCl_3$)

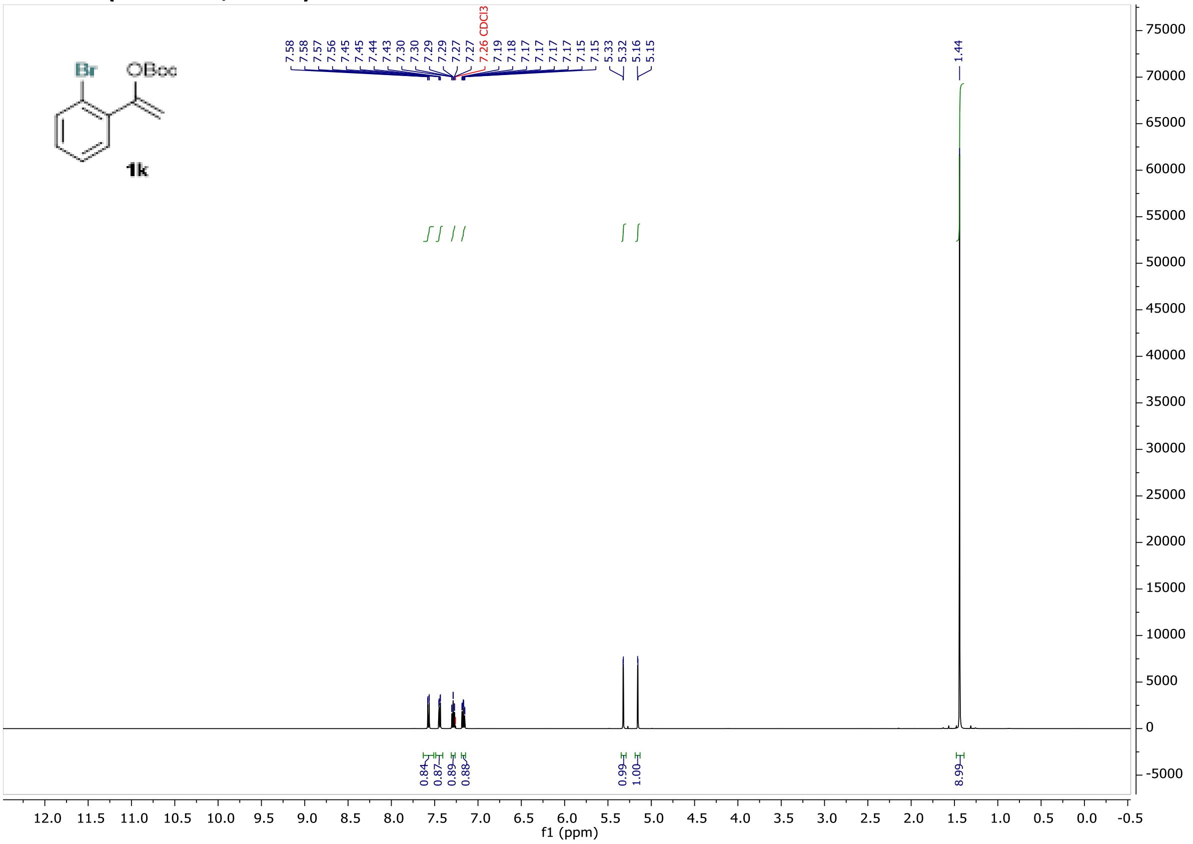

^{13}C NMR (126 MHz, $CDCl_3$)

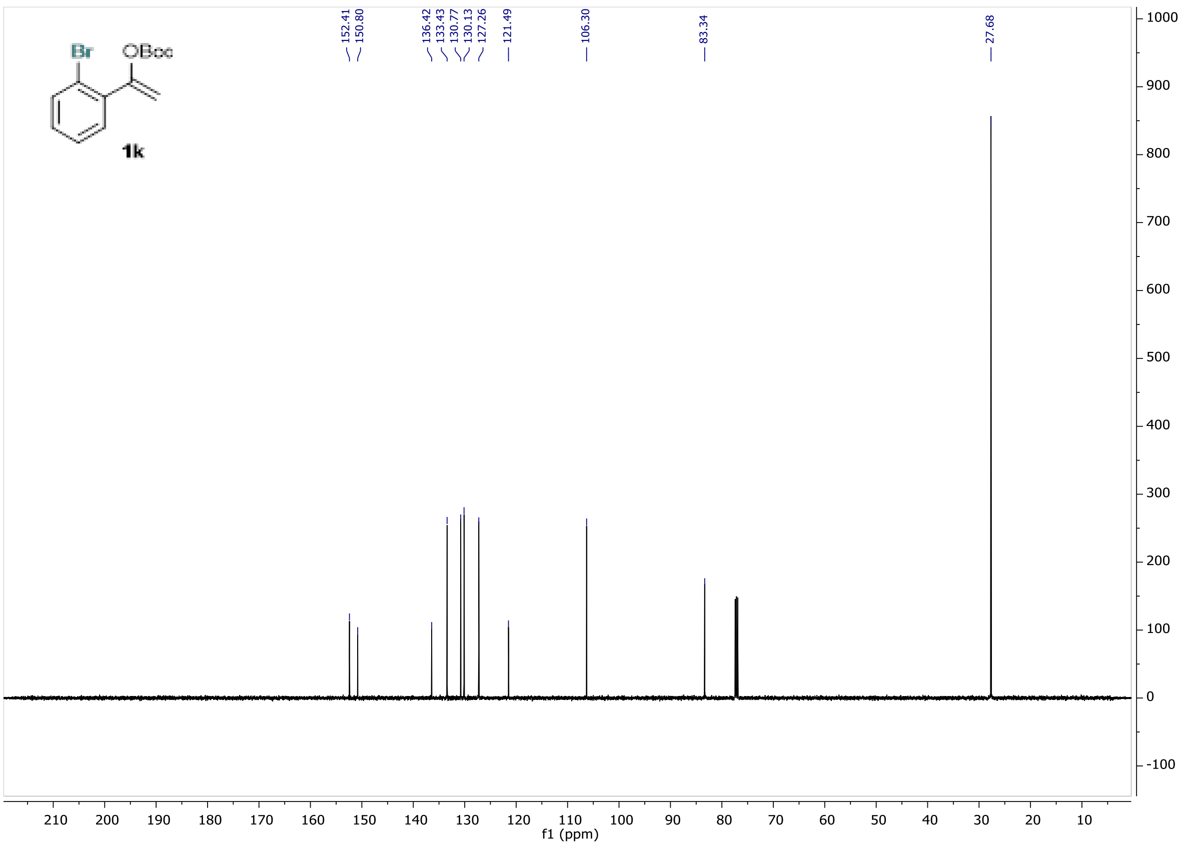

1-(3-bromophenyl)vinyl tert-butyl carbonate 1l

^{1}H NMR (500 MHz, $CDCl_3$)

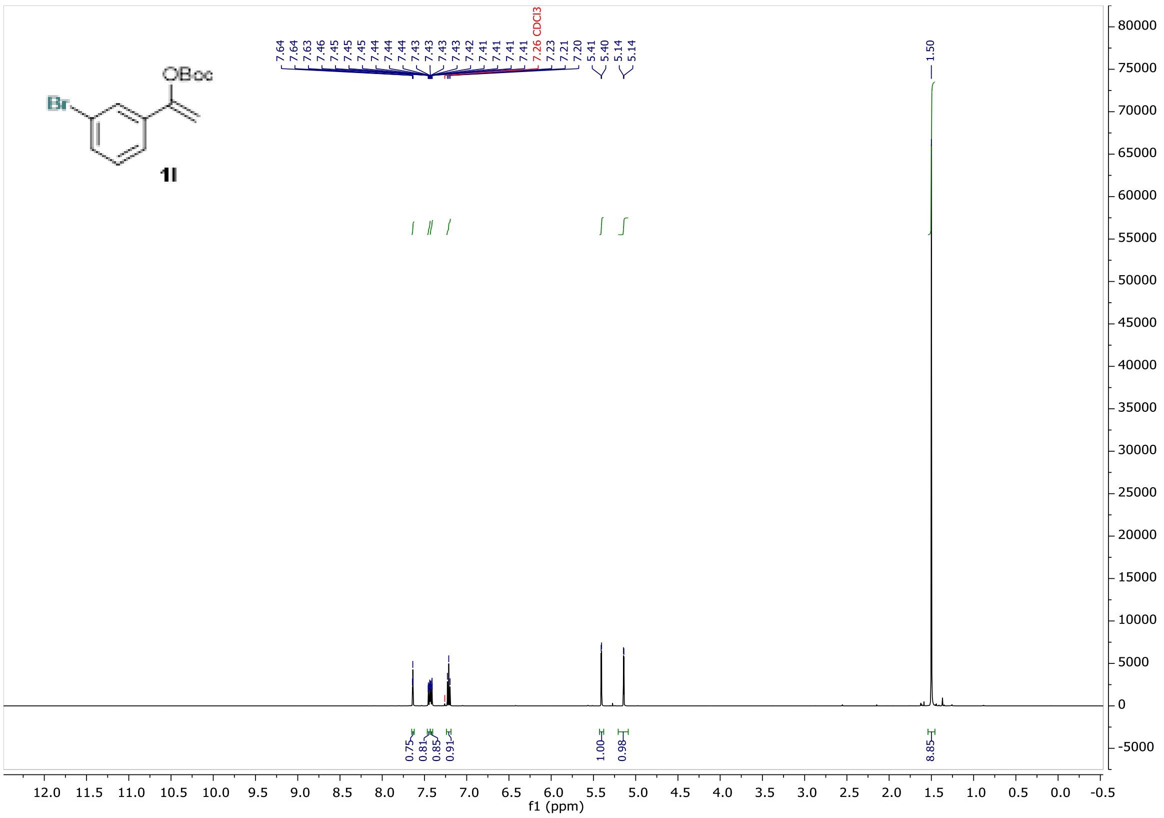

. ^{13}C NMR (126 MHz, $CDCl_3$)

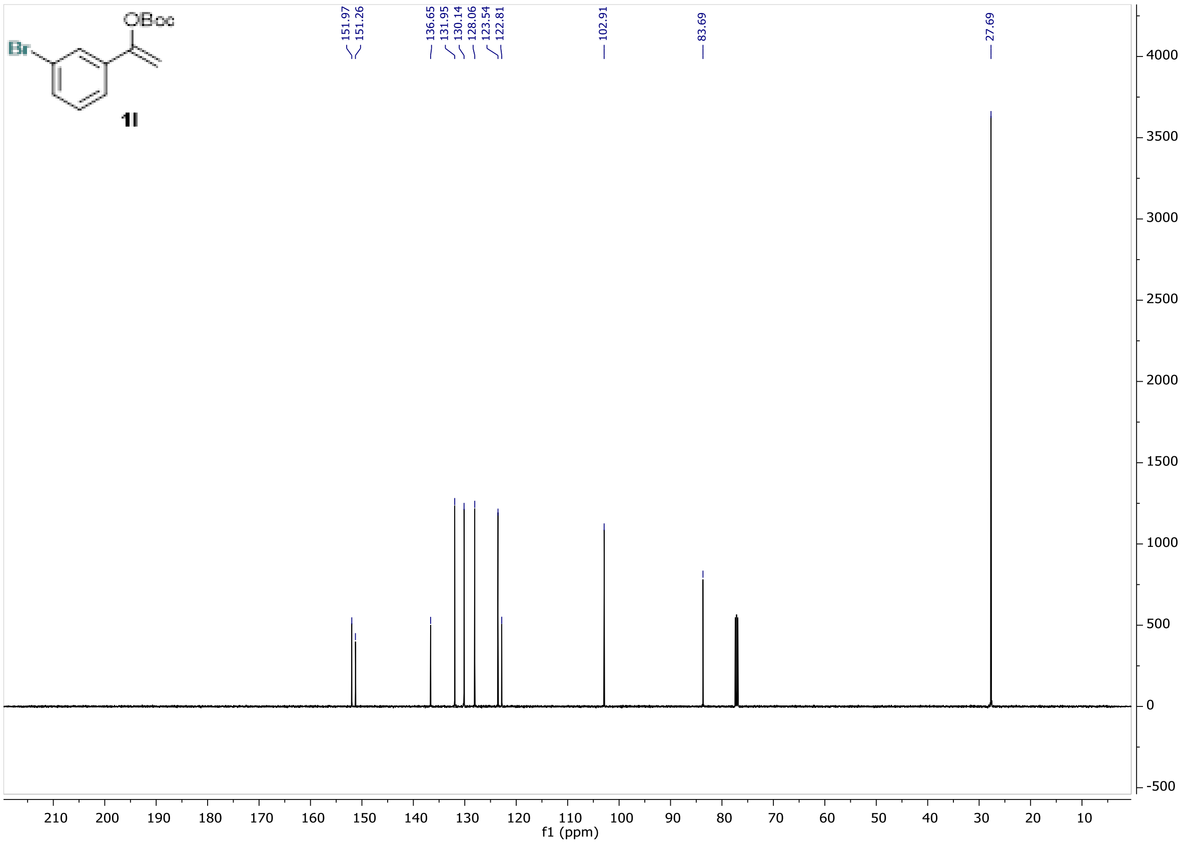

1-(4-bromophenyl)vinyl tert-butyl carbonate 1m

^{1}H NMR (500 MHz, $CDCl_3$)

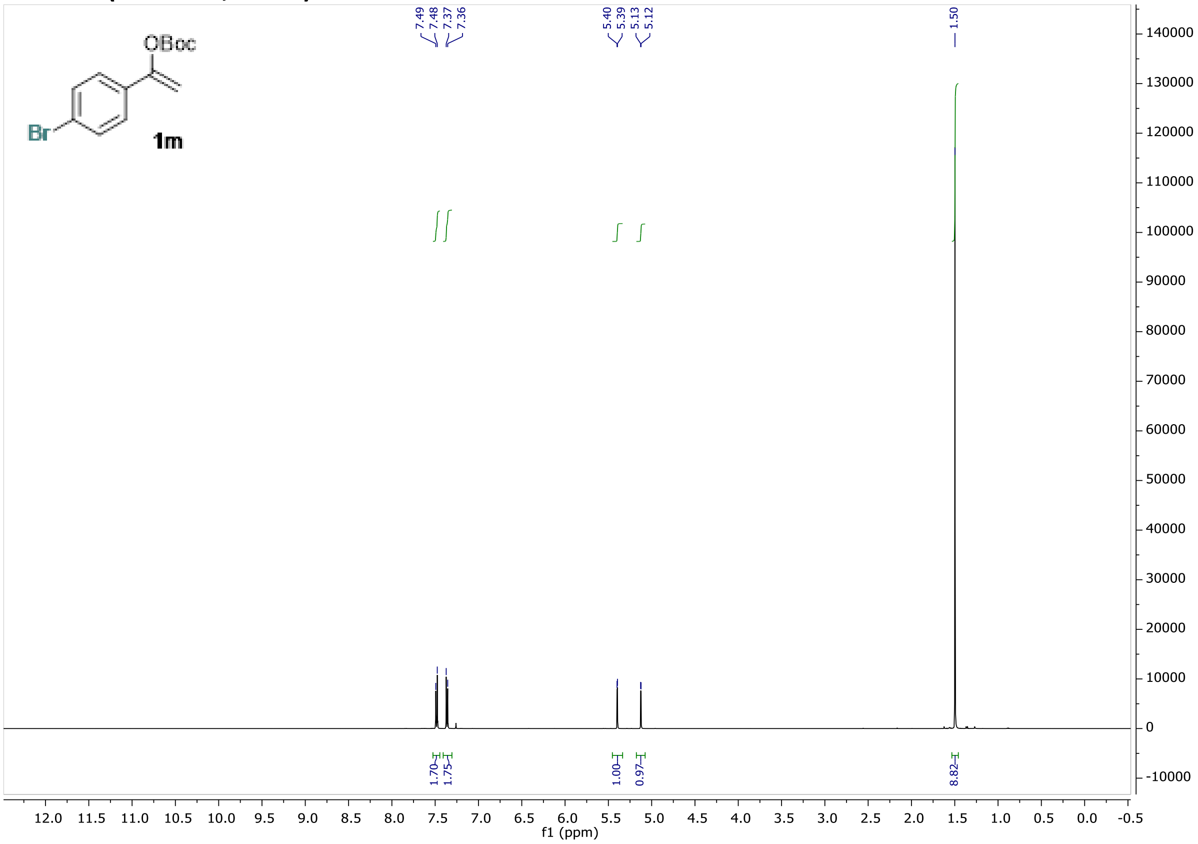

^{13}C NMR (126 MHz, $CDCl_3$)

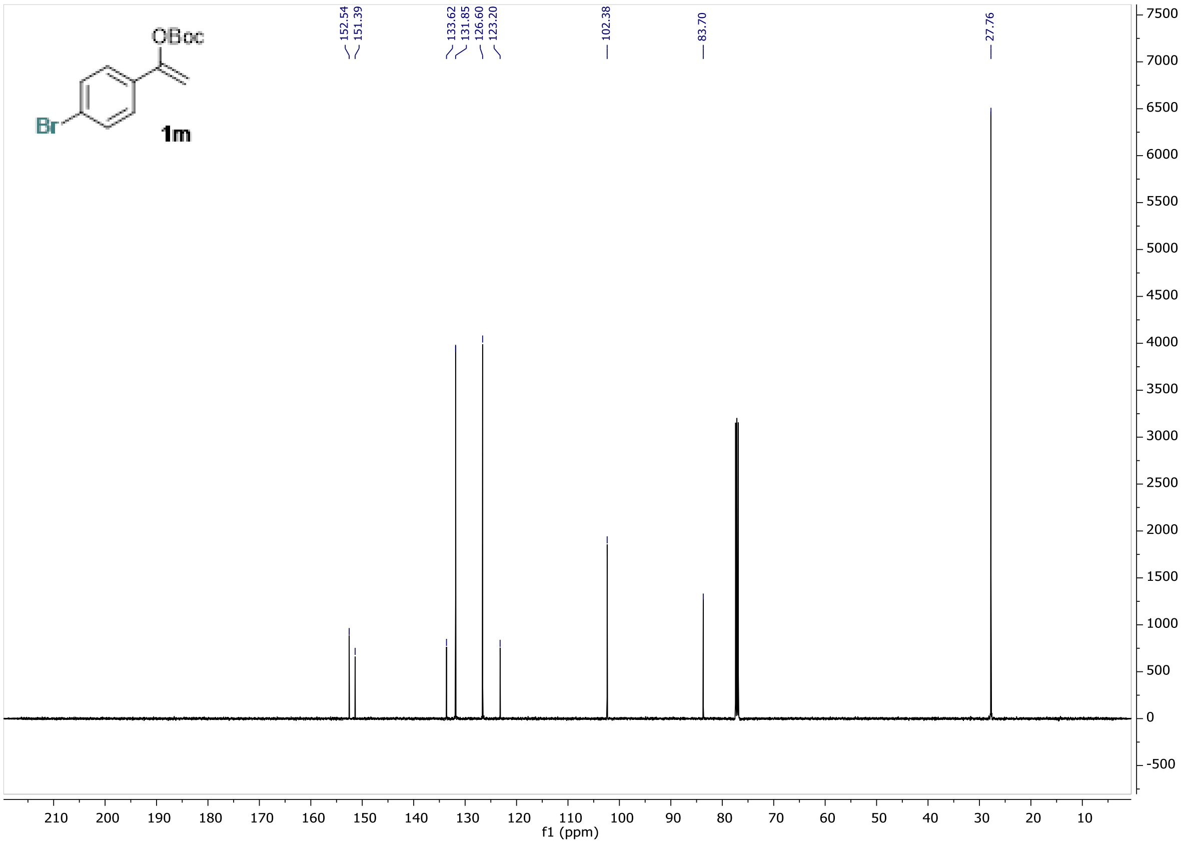

tert-butyl (1-phenylprop-1-en-1-yl) carbonate 1n

^{1}H NMR (500 MHz, $CDCl_3$)

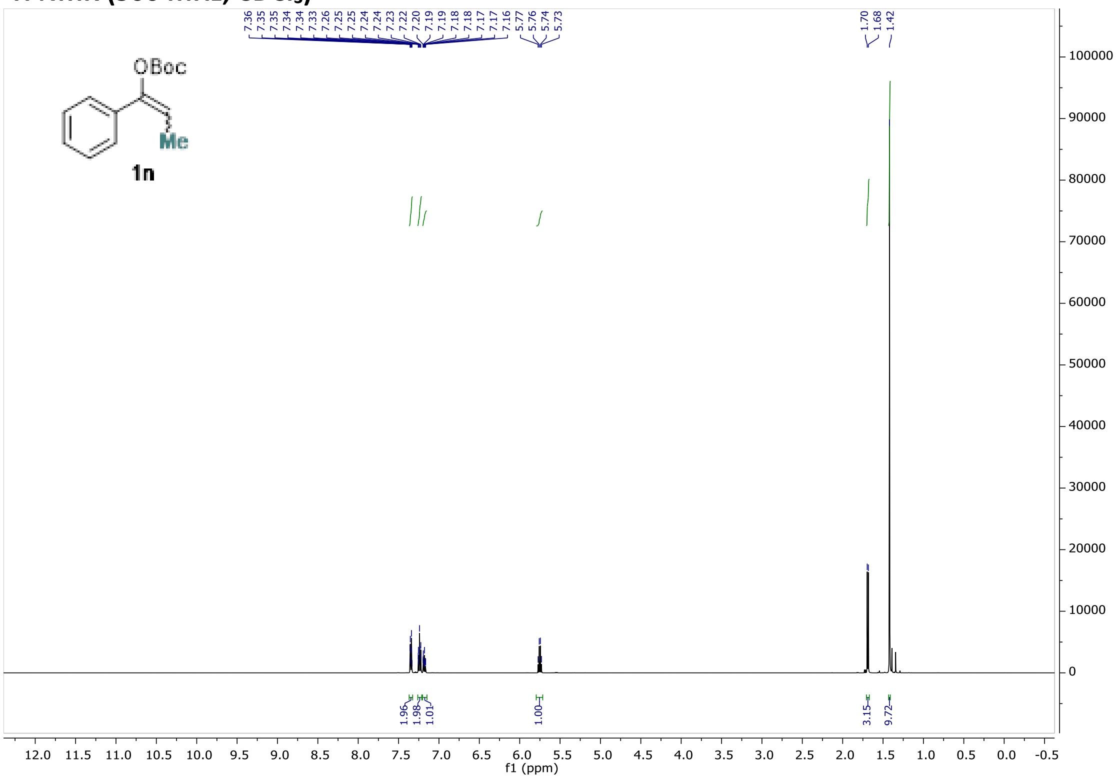

^{13}C NMR (126 MHz, $CDCl_3$)

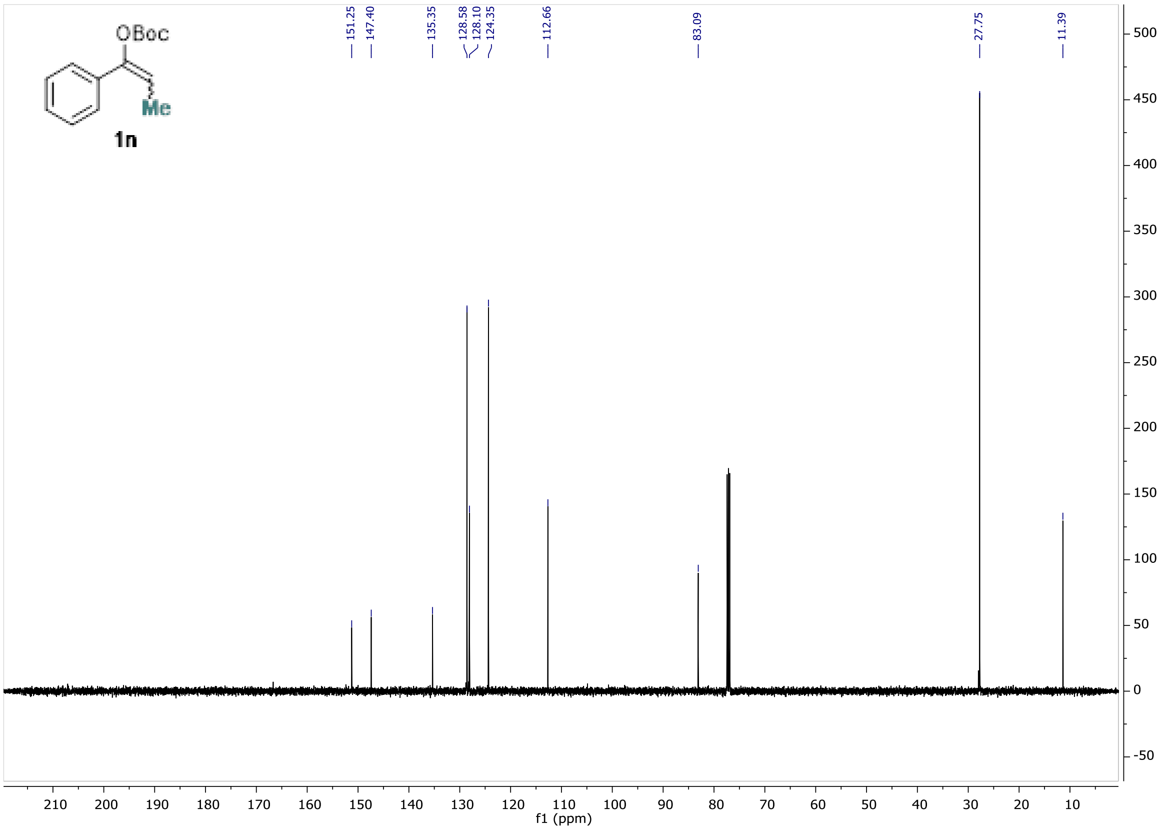

tert-butyl (1,3-diphenylprop-1-en-1-yl) carbonate 1o

^{1}H NMR (500 MHz, $CDCl_3$)

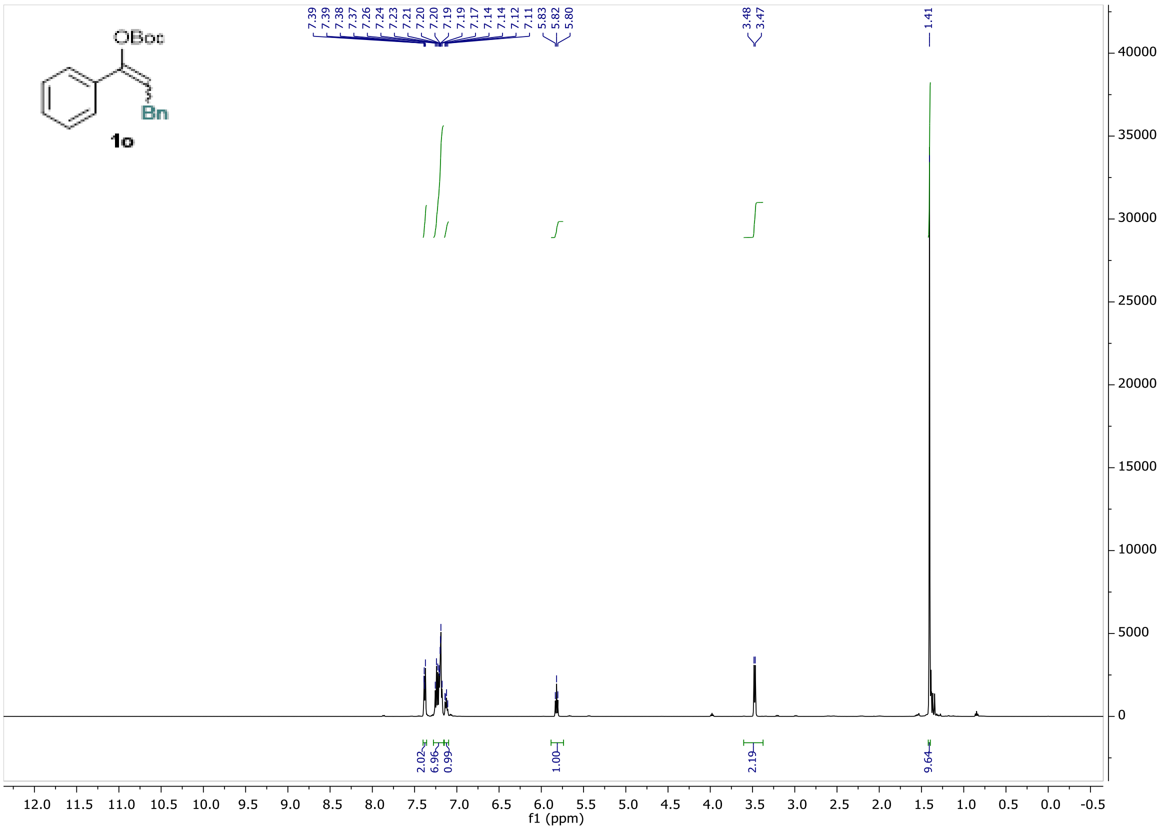

^{13}C NMR (126 MHz, CDCl$_3$)

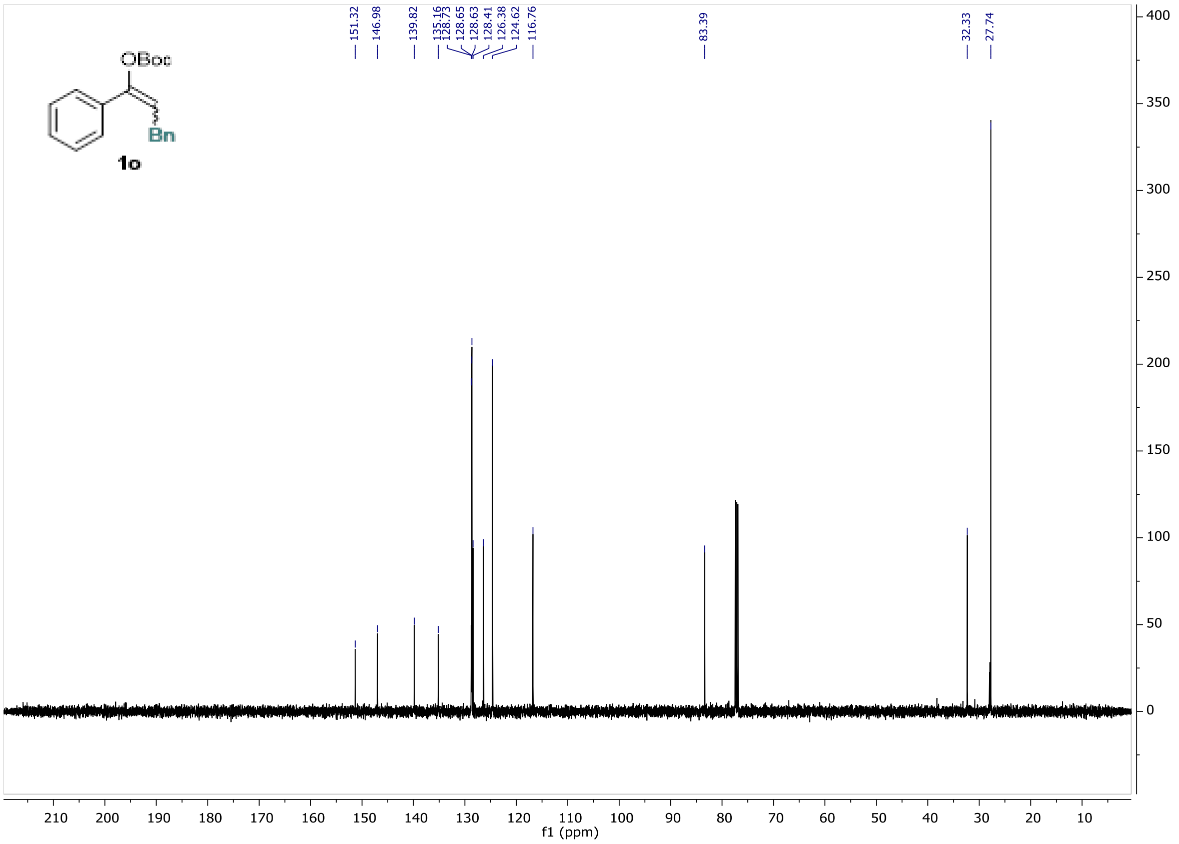

tert-butyl 1H-inden-3-yl carbonate 1p

^{1}H NMR (300 MHz, CDCl$_3$)

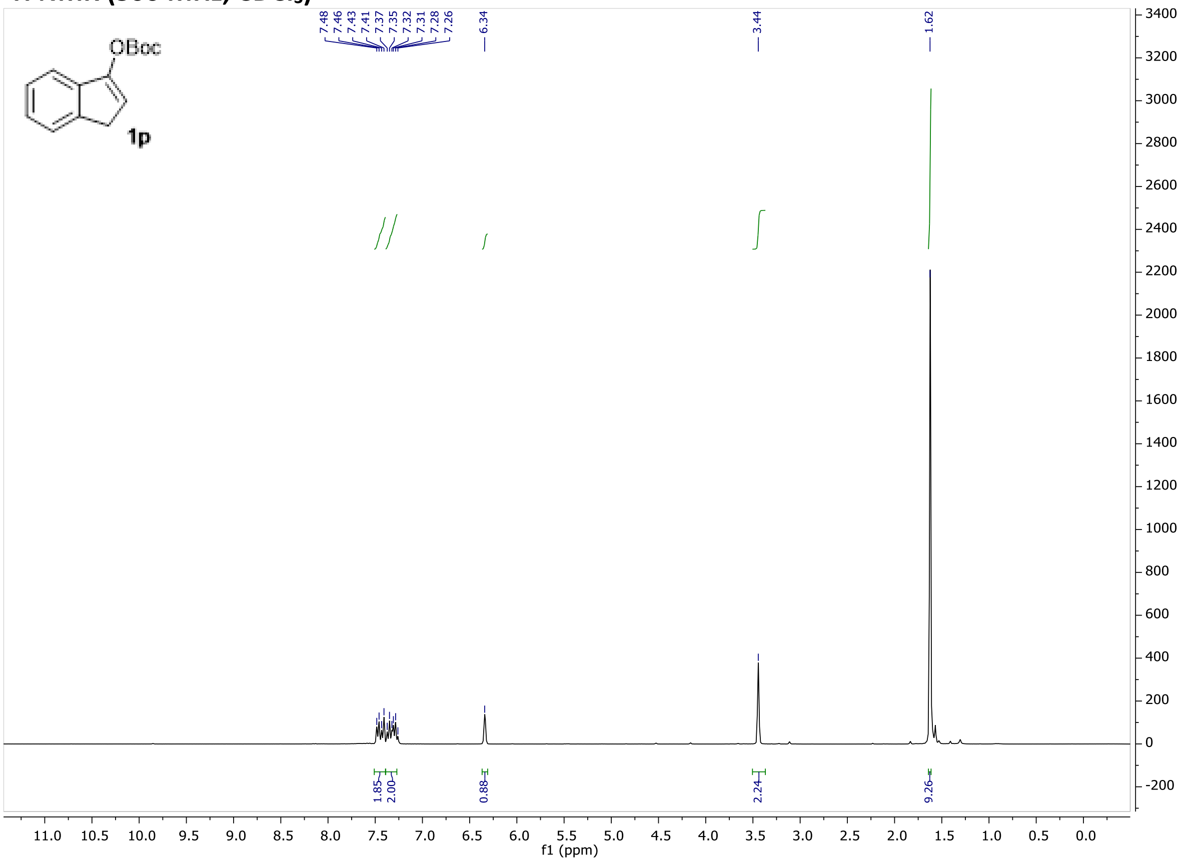

^{13}C NMR (75 MHz, $CDCl_3$)

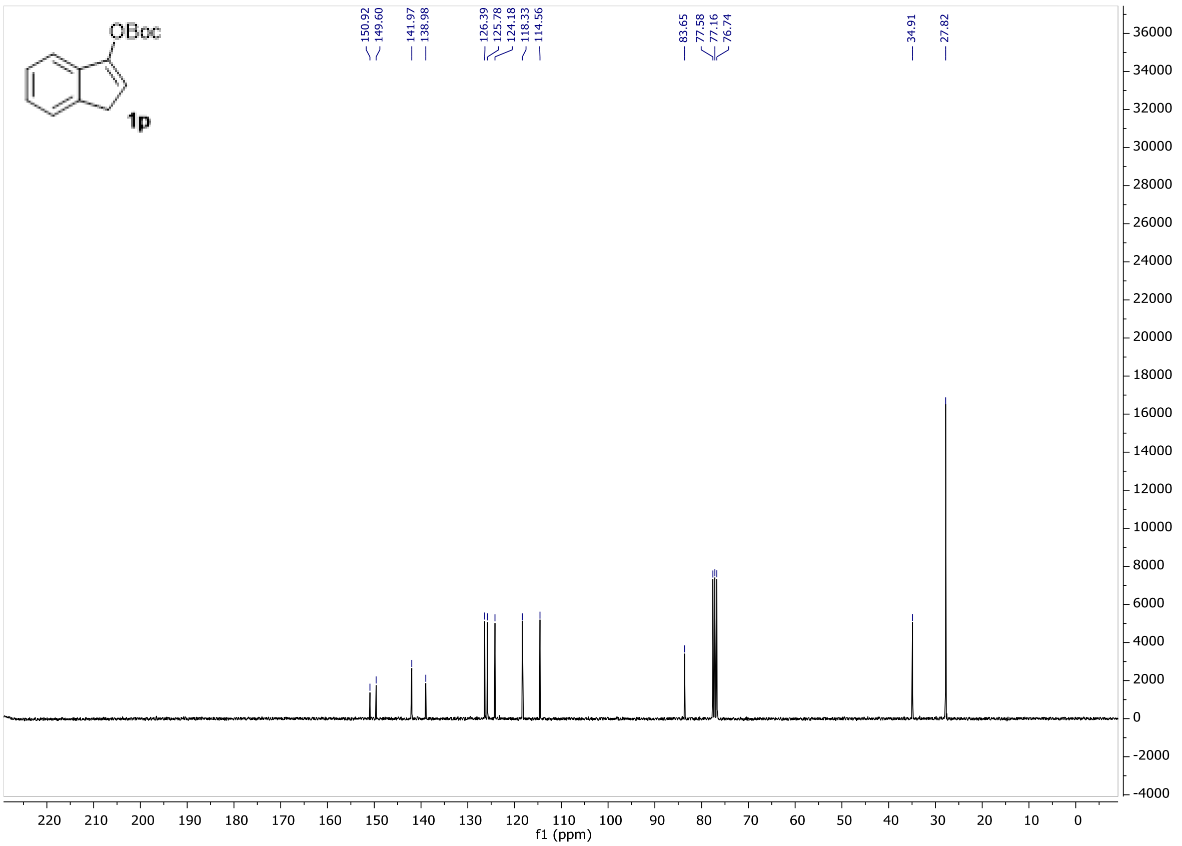

tert-butyl (6-fluoro-1H-inden-3-yl) carbonate 1q

^{1}H NMR (300 MHz, $CDCl_3$)

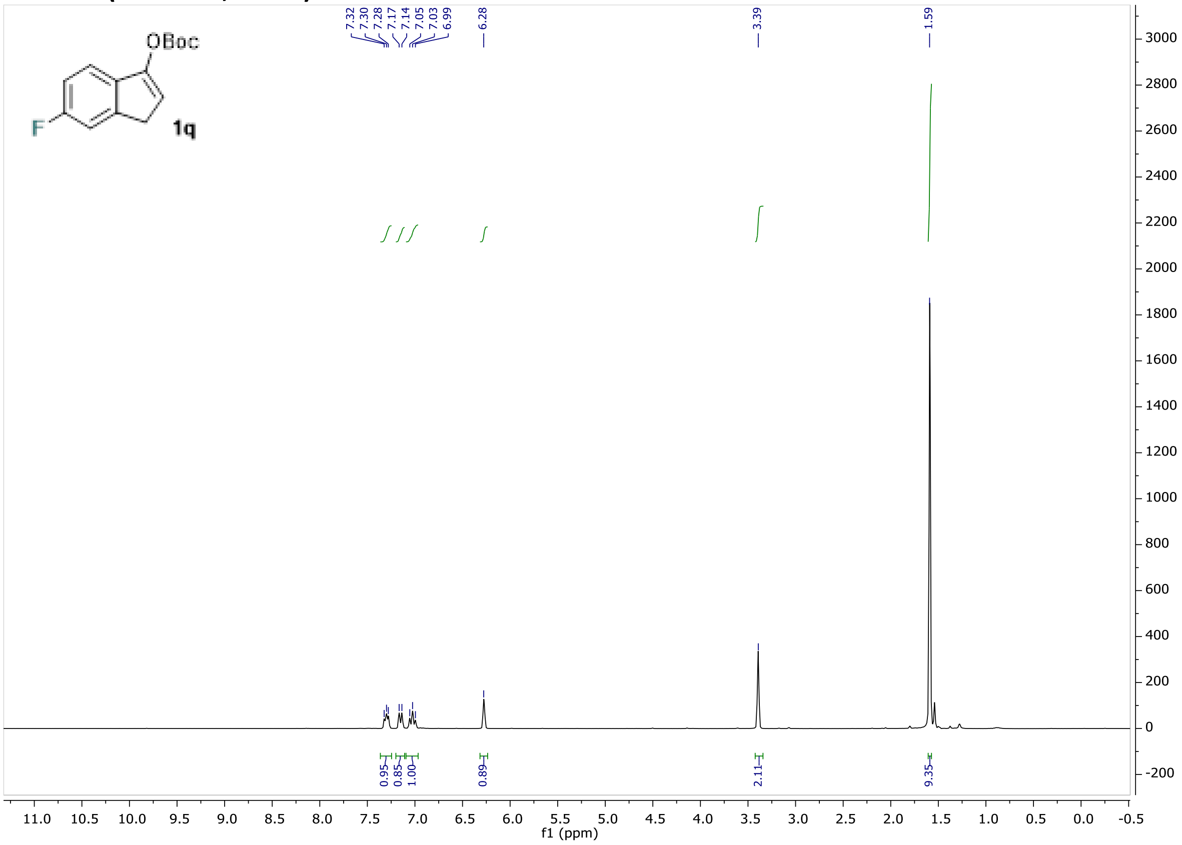

^{13}C NMR (75 MHz, CDCl$_3$)

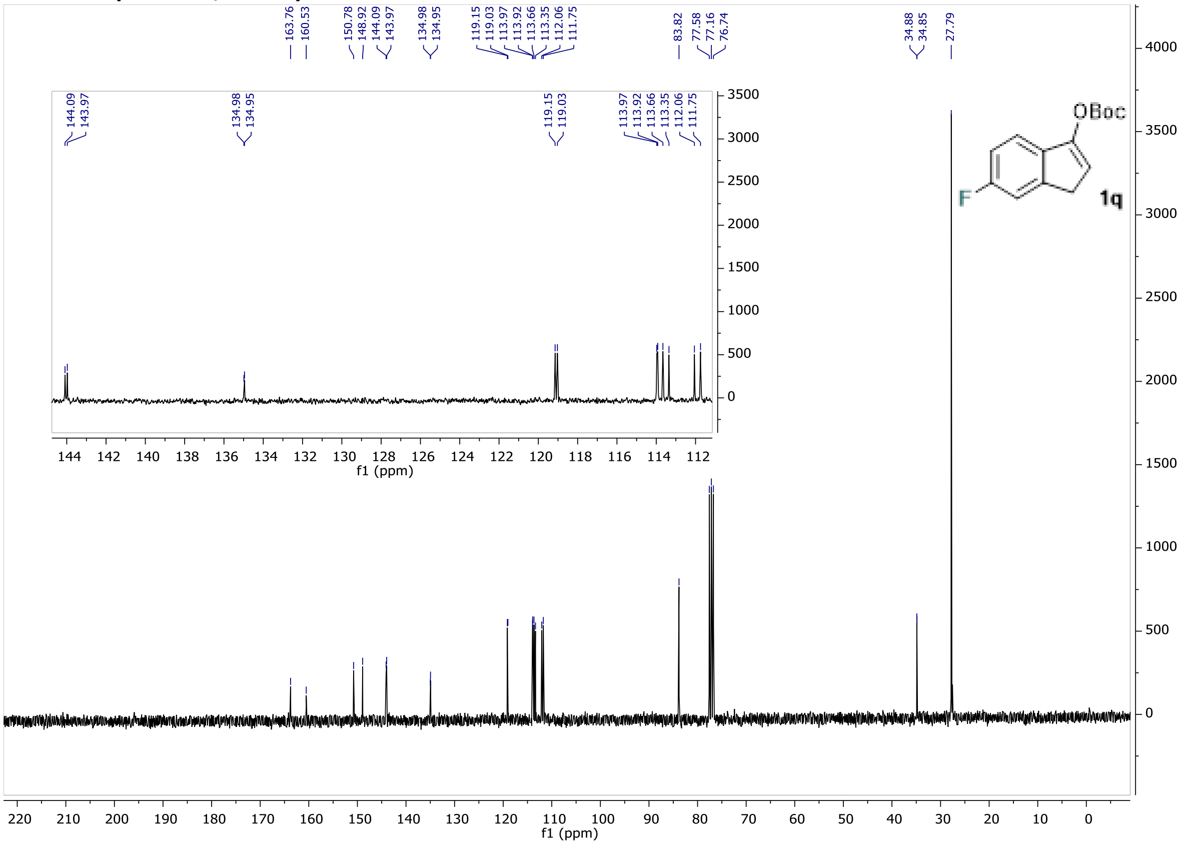

^{19}F NMR (188 MHz, CDCl$_3$

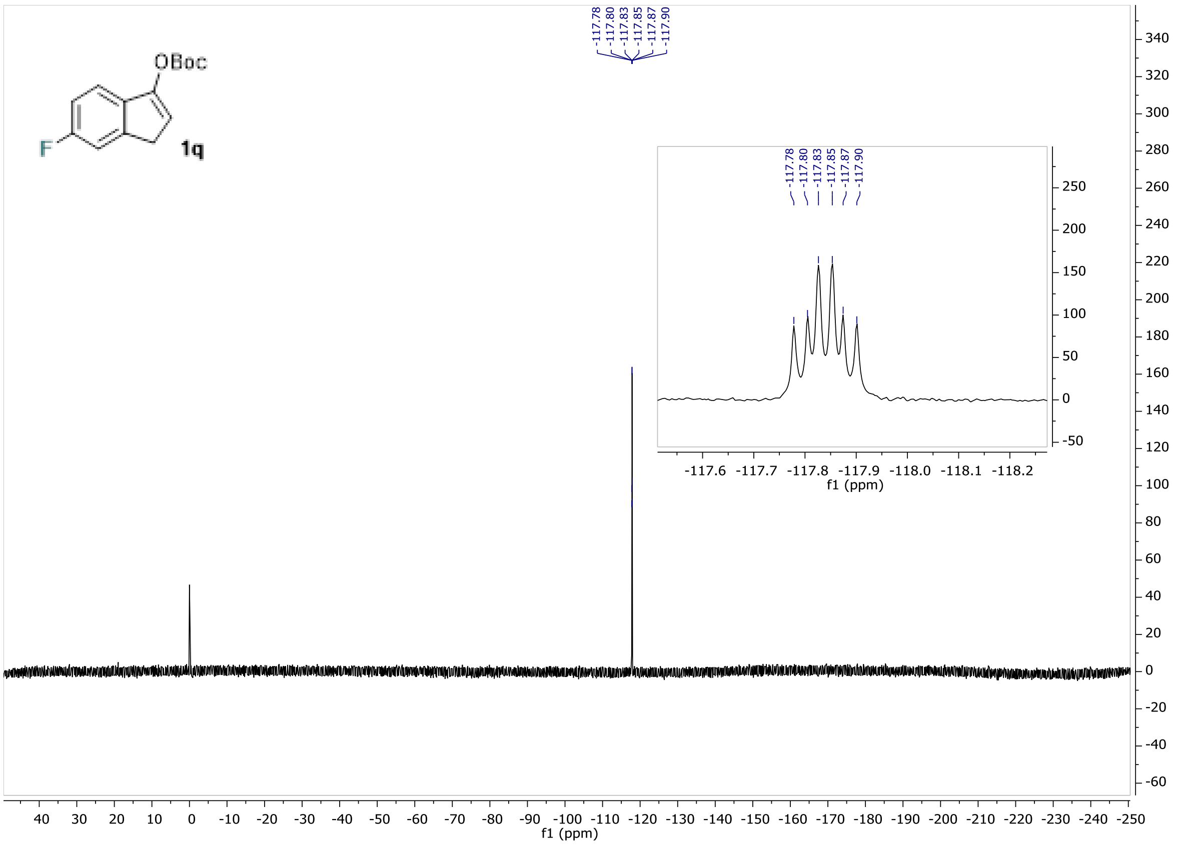

tert-butyl (6,7-dihydro-5H-benzo[7]annulen-9-yl) carbonate 1r

^{1}H NMR (300 MHz, $CDCl_3$)

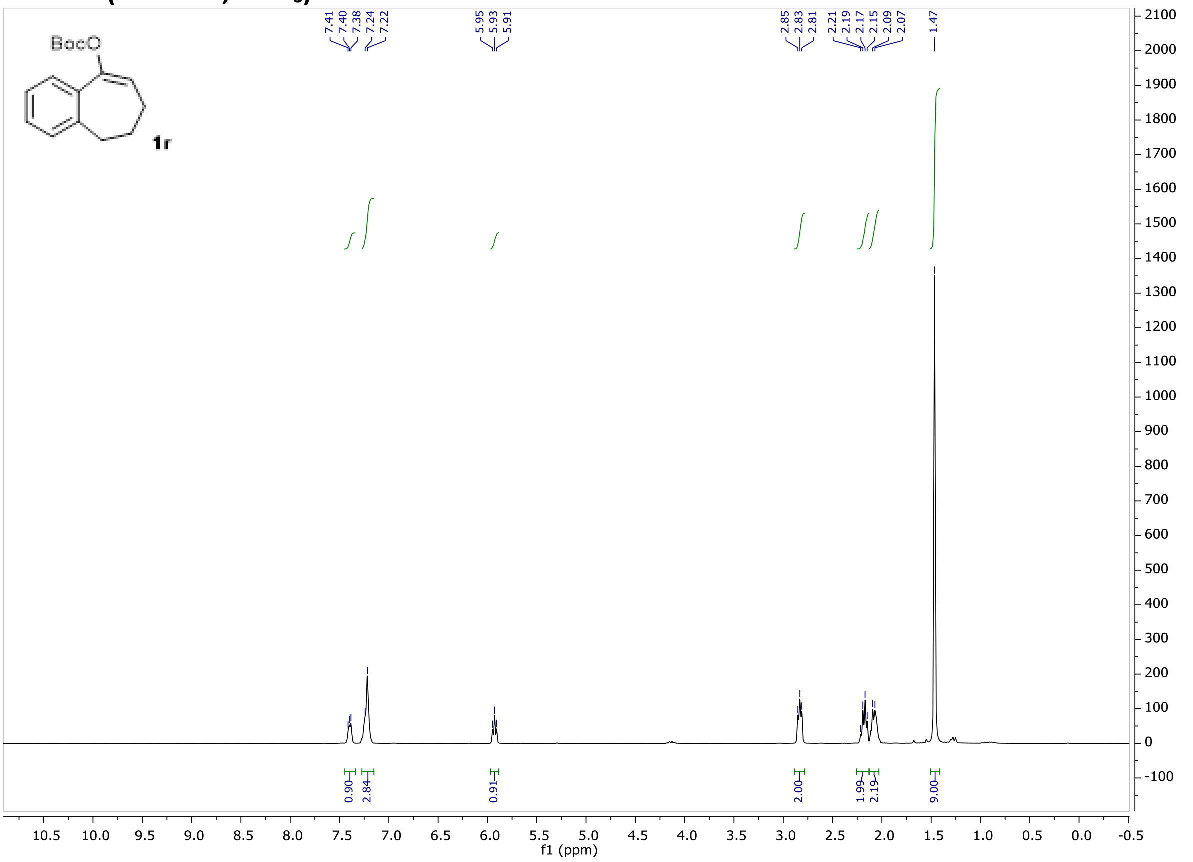

^{13}C NMR (75 MHz, $CDCl_3$)

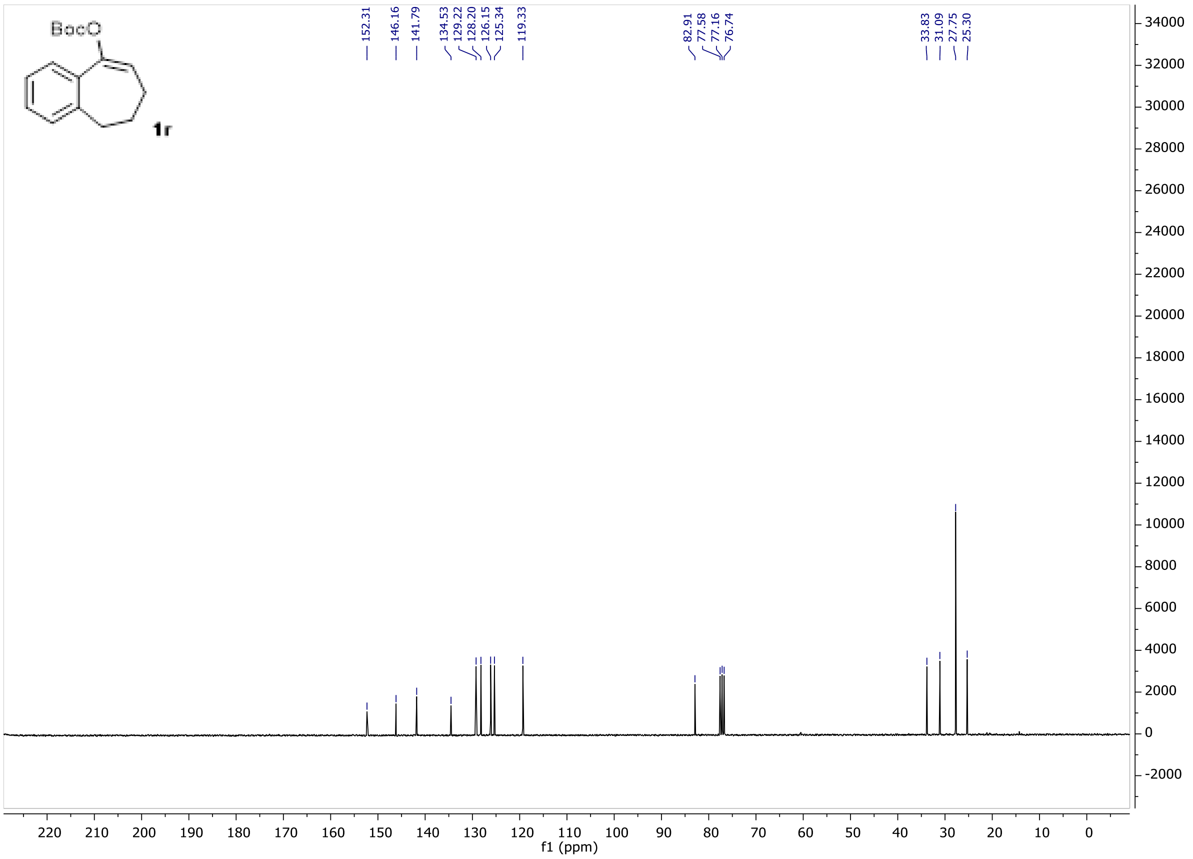

tert-butyl 2,2-difluoro-1-(naphthalen-2-yl)vinyl carbonate 1t

^{1}H NMR (500 MHz, $CDCl_3$)

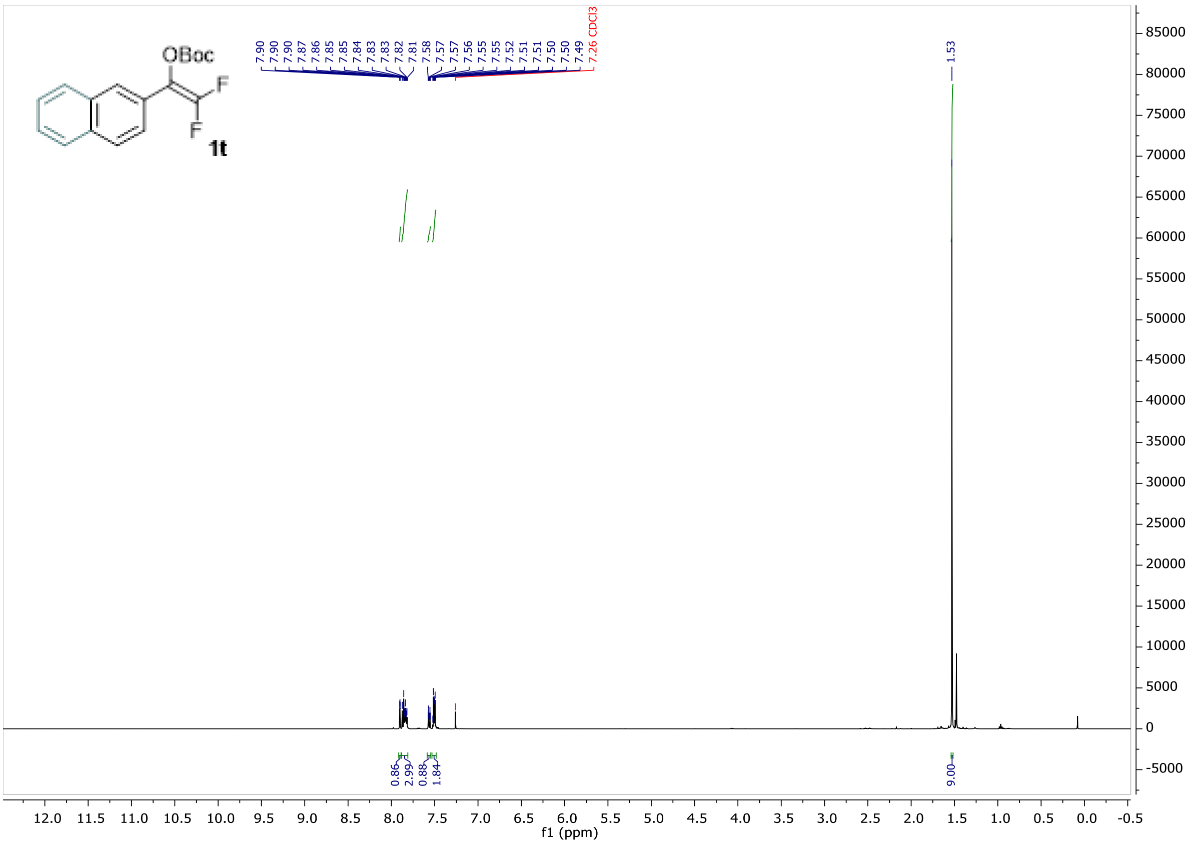

^{13}C NMR (126 MHz, $CDCl_3$)

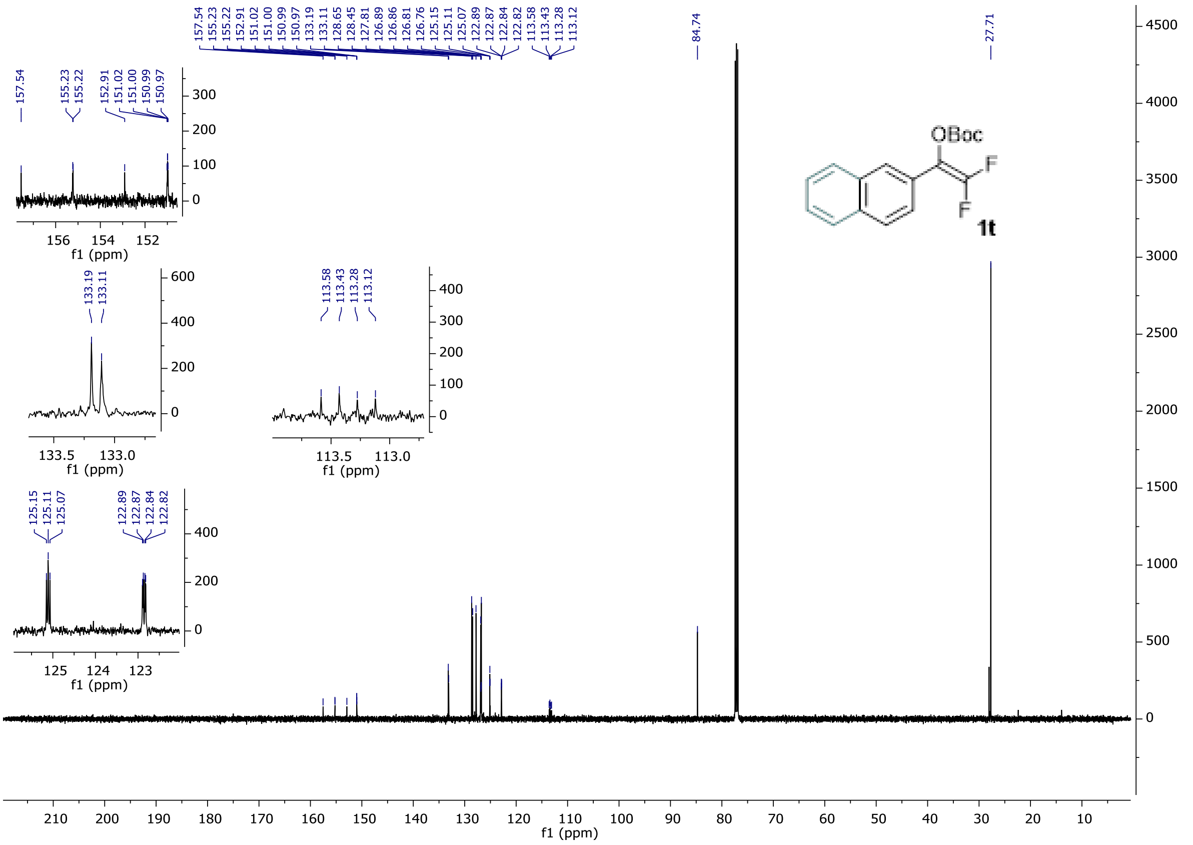

^{19}F NMR (188 MHz, CDCl$_3$)

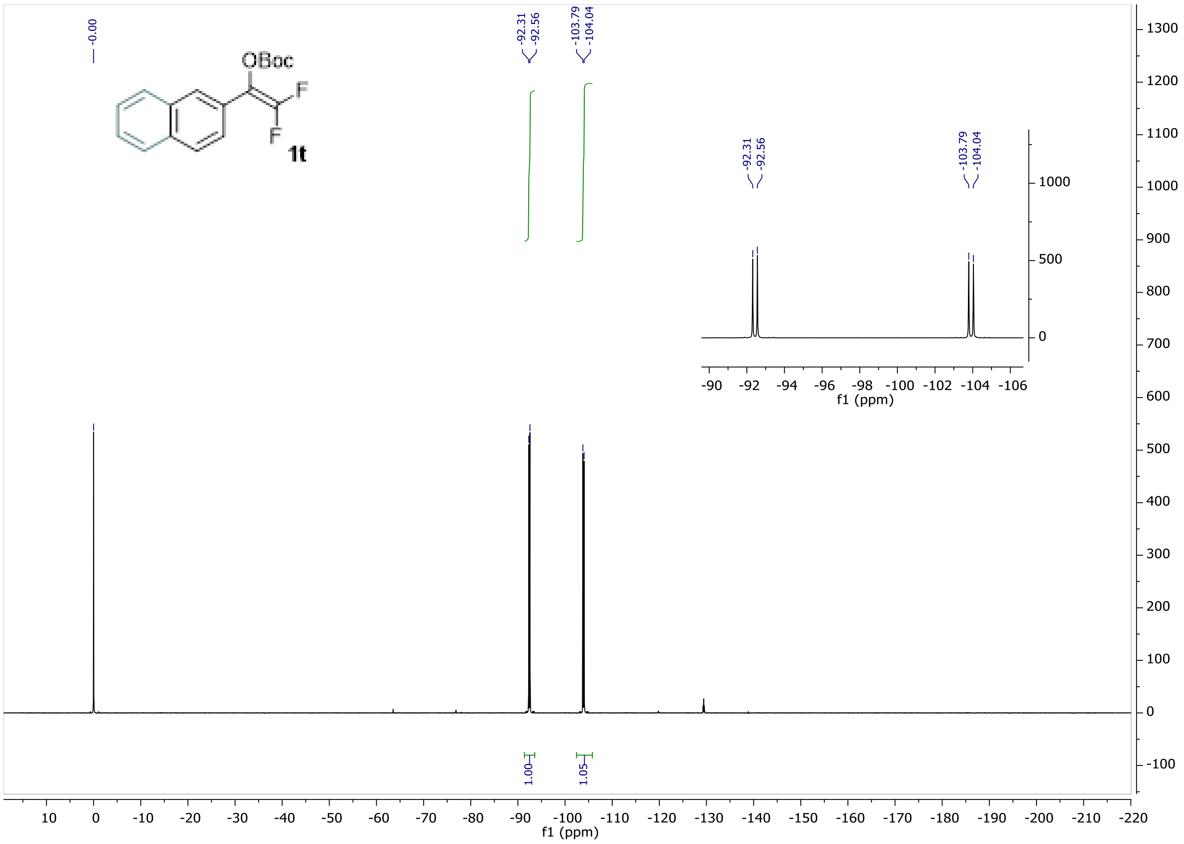

tert-butyl oct-1-en-2-yl carbonate 1u

^{1}H NMR (400 MHz, CDCl$_3$)

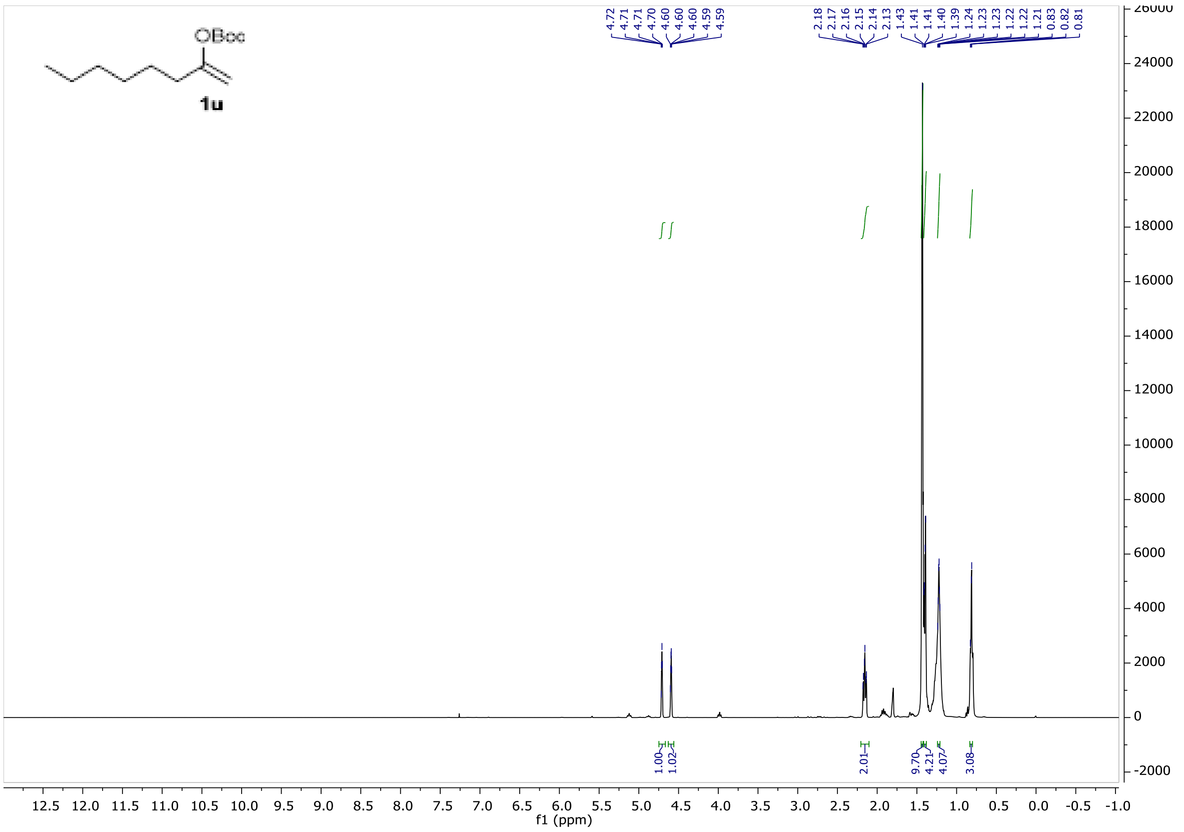

^{13}C NMR (101 MHz, $CDCl_3$)

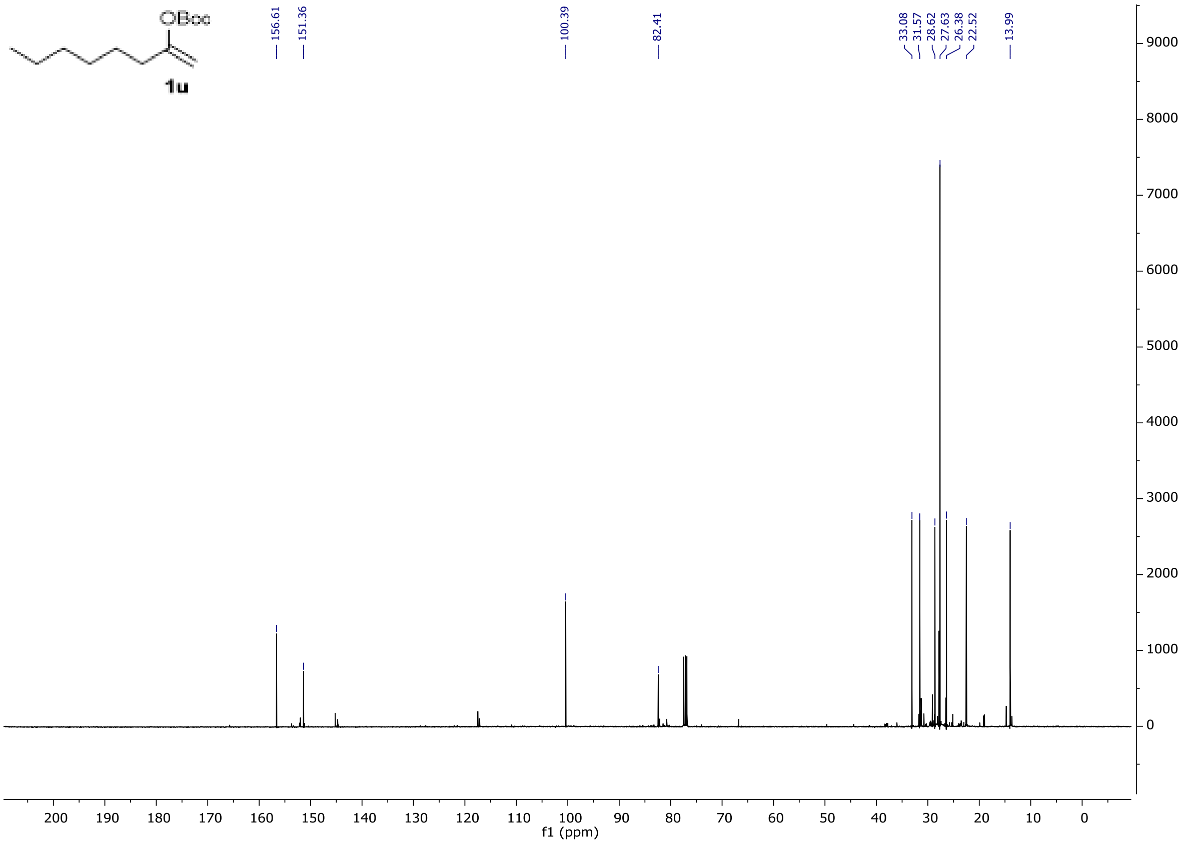

(E)-tert-butyl (4-phenylbuta-1,3-dien-2-yl) carbonate 1v

^{1}H NMR (300 MHz, $CDCl_3$)

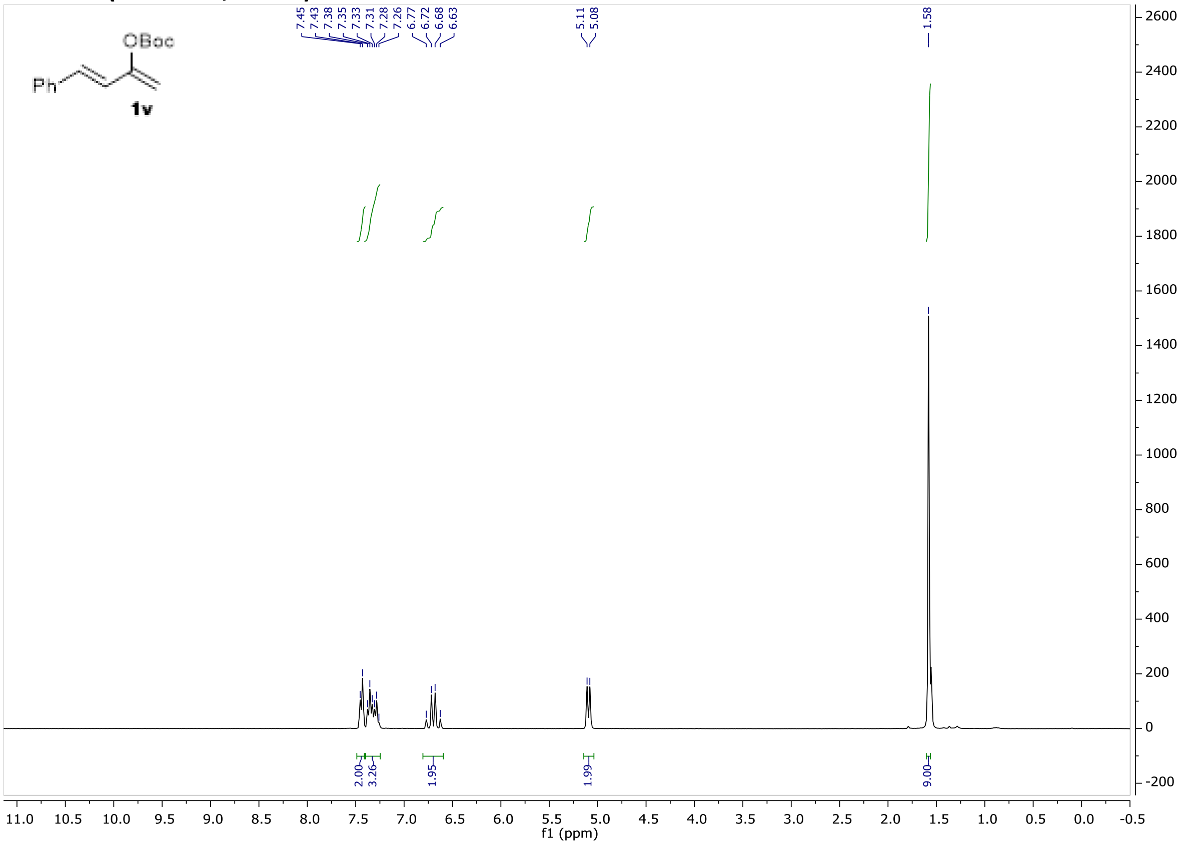

^{13}C NMR (75 MHz, $CDCl_3$)

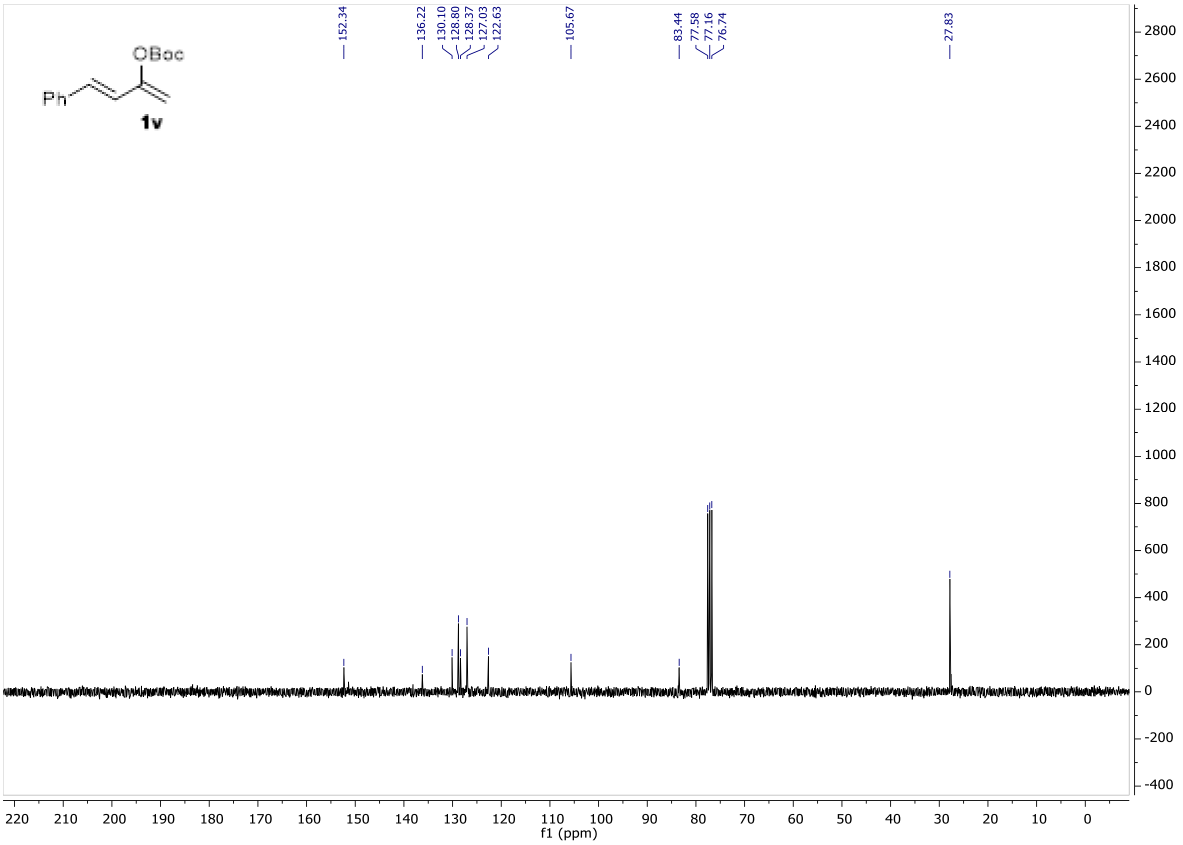

(E)-tert-butyl nona-1,3-dien-2-yl carbonate 1w

^{1}H NMR (300 MHz, $CDCl_3$)

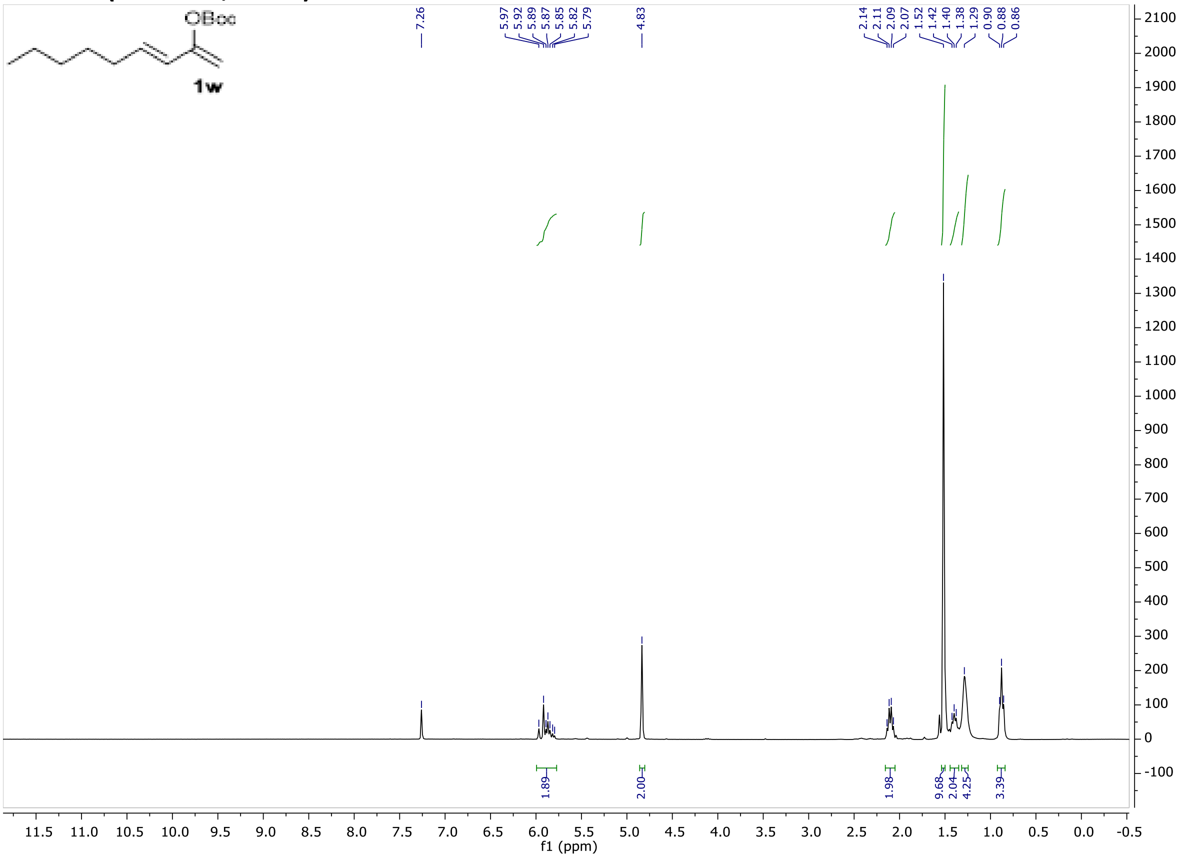

^{13}C NMR (75 MHz, $CDCl_3$)

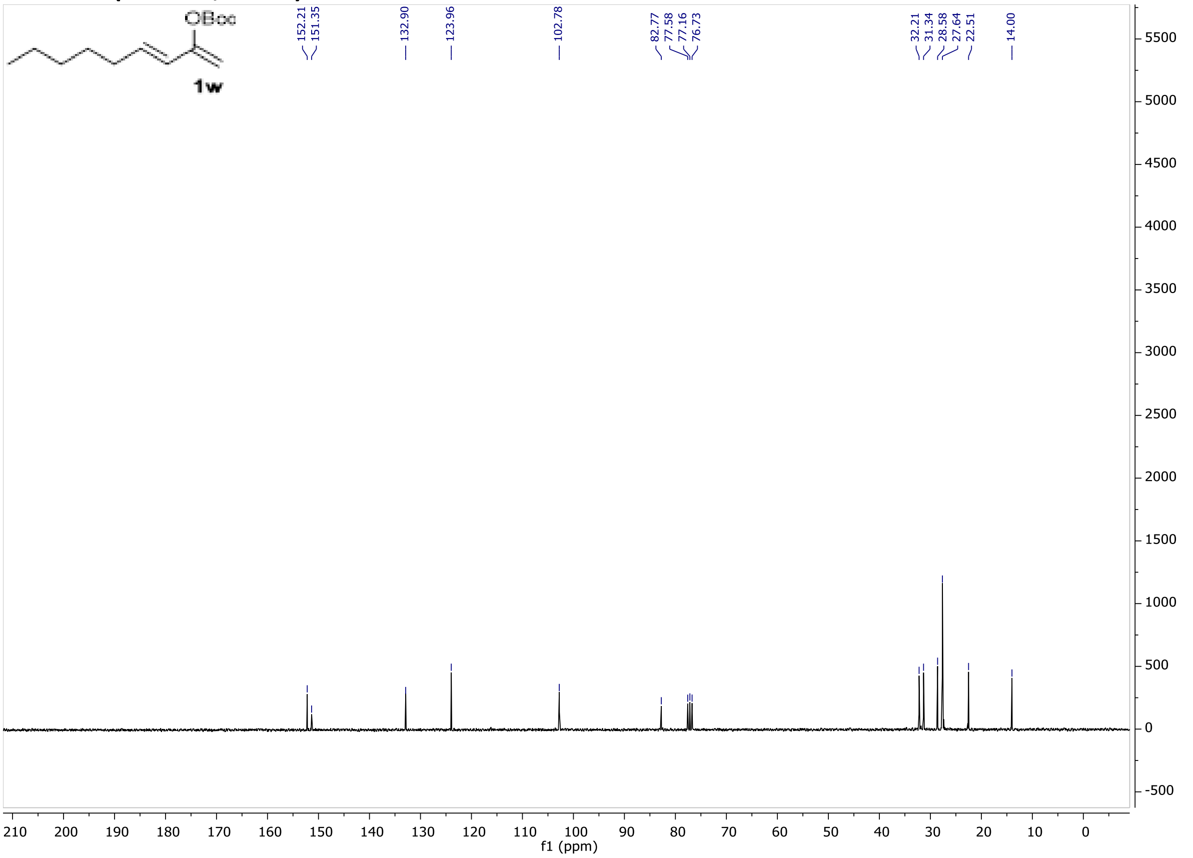

tert-butyl 1-(3,5,5,6,8,8-hexamethyl-5,6,7,8-tetrahydronaphthalen-2-yl)vinyl carbonate 1x

^{1}H NMR (500 MHz, $CDCl_3$)

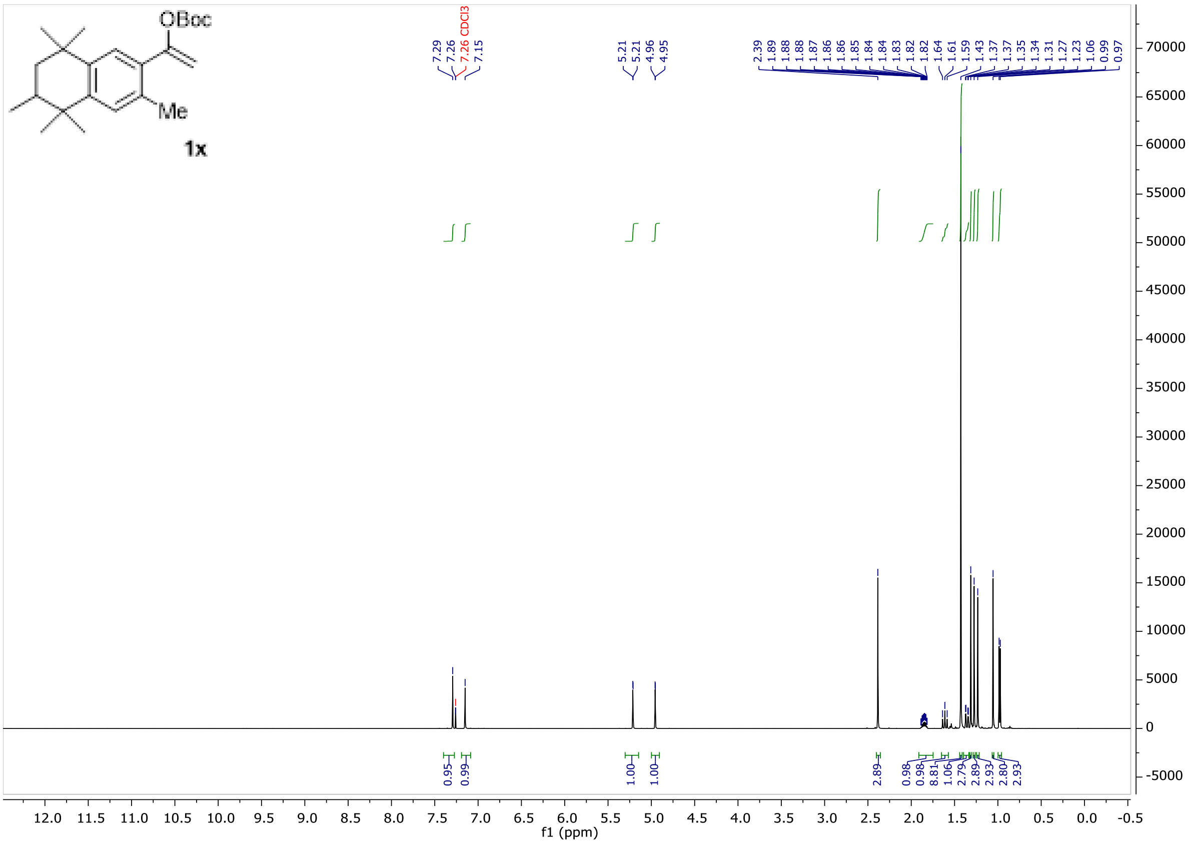

^{13}C NMR (126 MHz, $CDCl_3$)

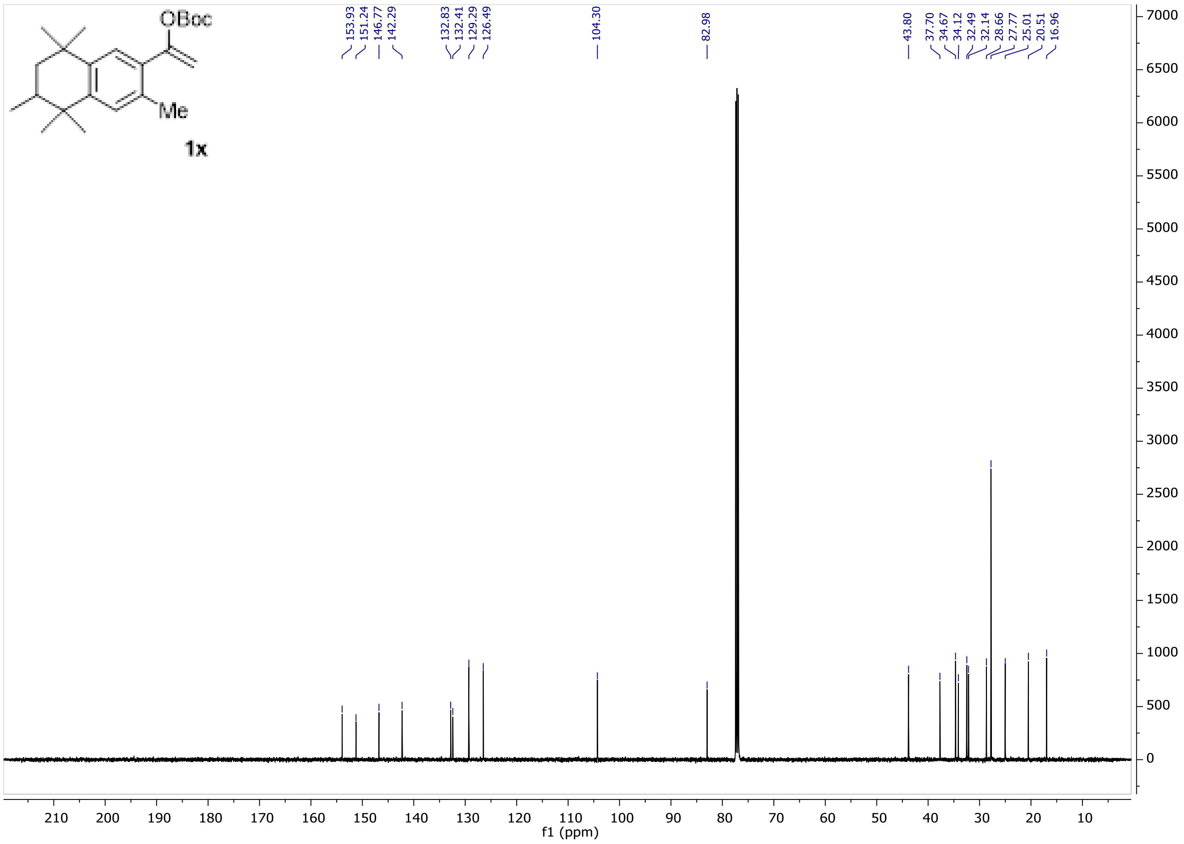

tert-butyl (1-(4-(tert-butyl)-2,6-dimethyl-3,5-dinitrophenyl)vinyl) carbonate 1y

^{1}H NMR (300 MHz, $CDCl_3$)

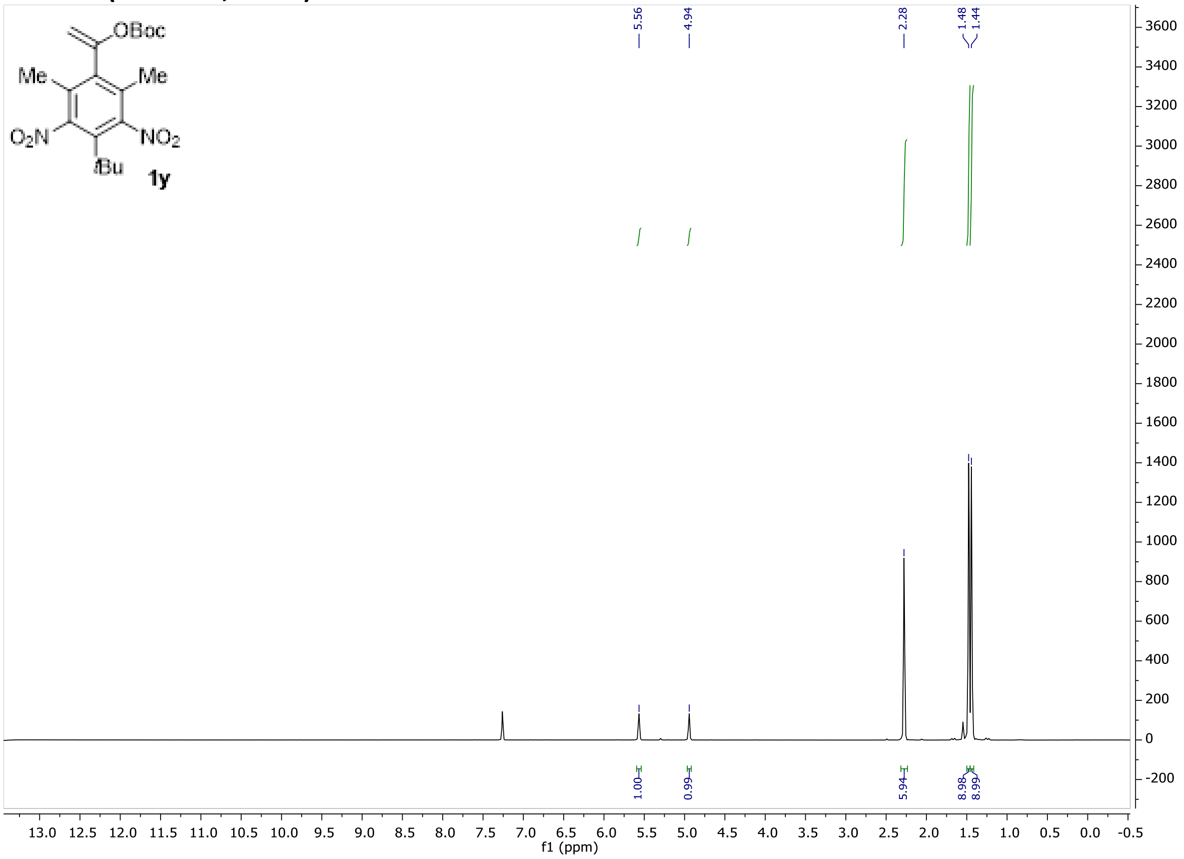

^{13}C NMR (75 MHz, $CDCl_3$)

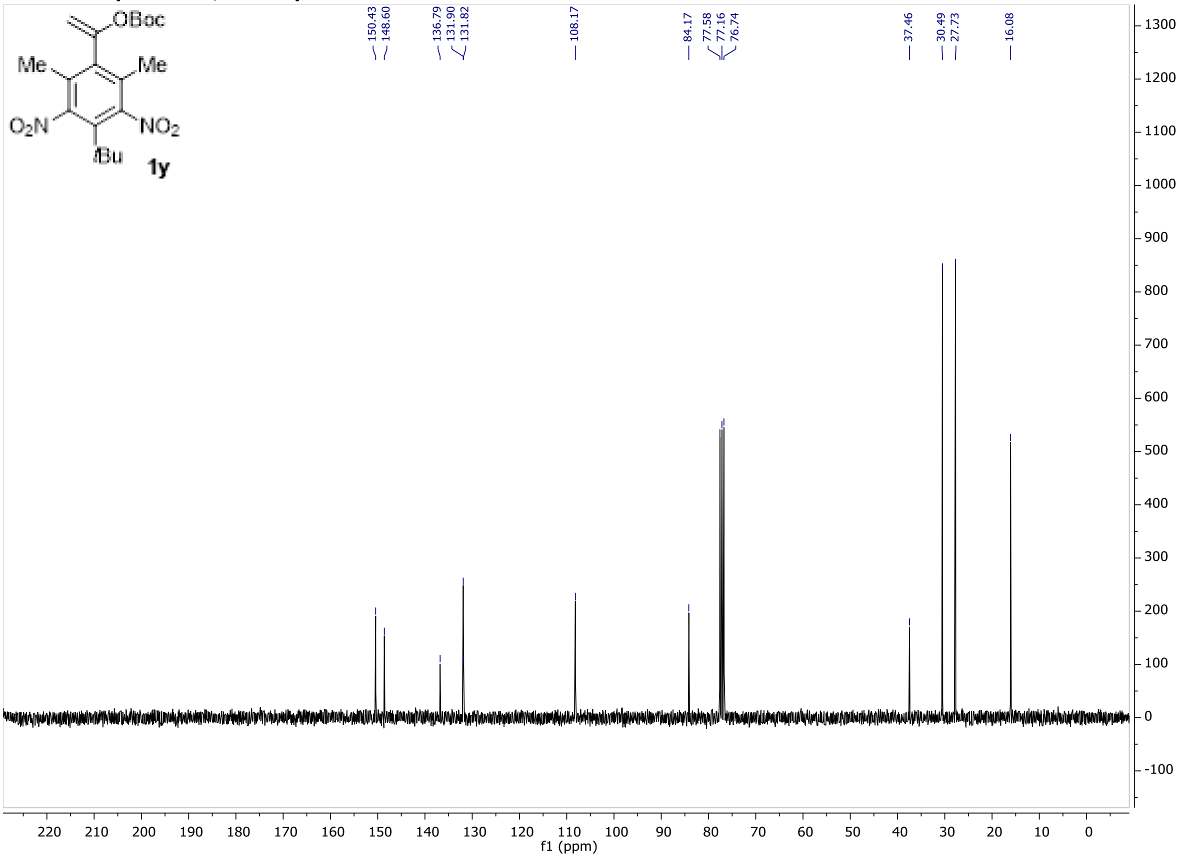

tert-butyl (1-(6-(tert-butyl)-1,1-dimethyl-2,3-dihydro-1H-inden-4-yl)vinyl) carbonate 1z

^{1}H NMR (300 MHz, $CDCl_3$)

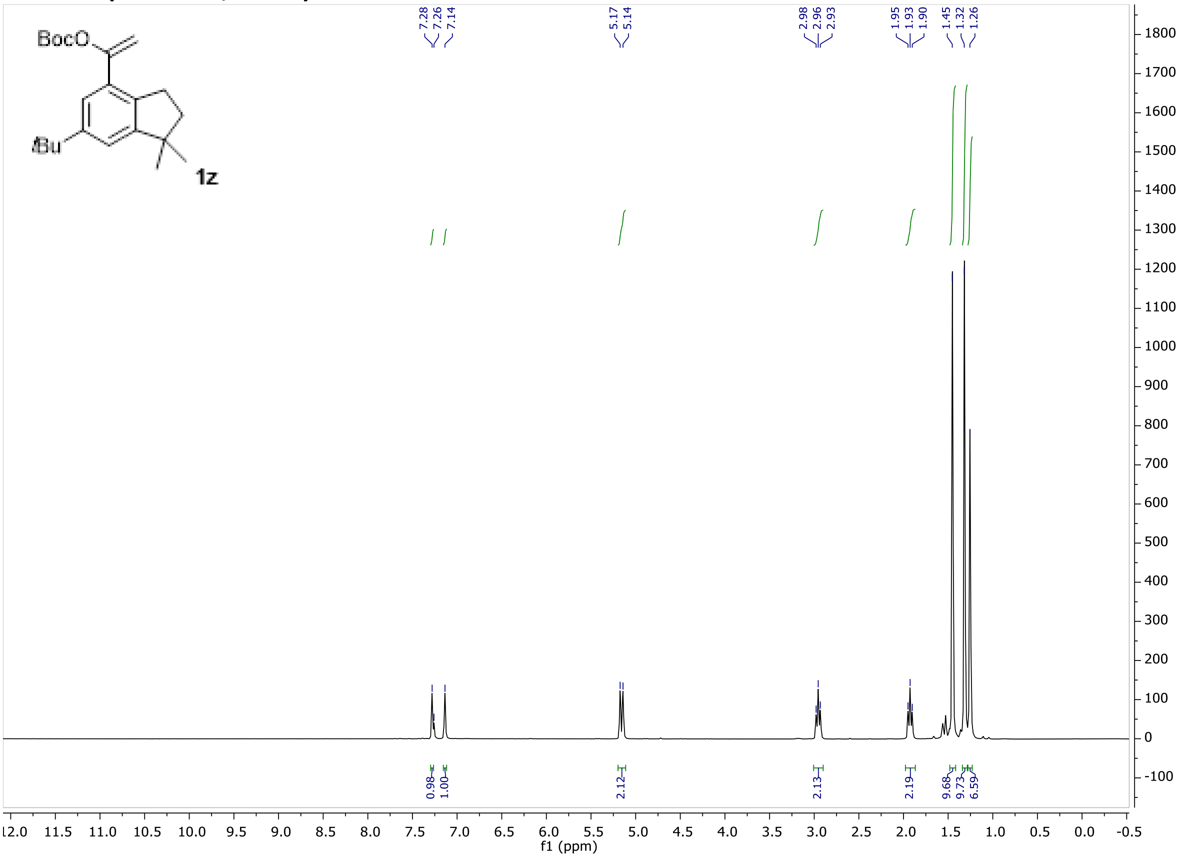

^{13}C NMR (75 MHz, $CDCl_3$)

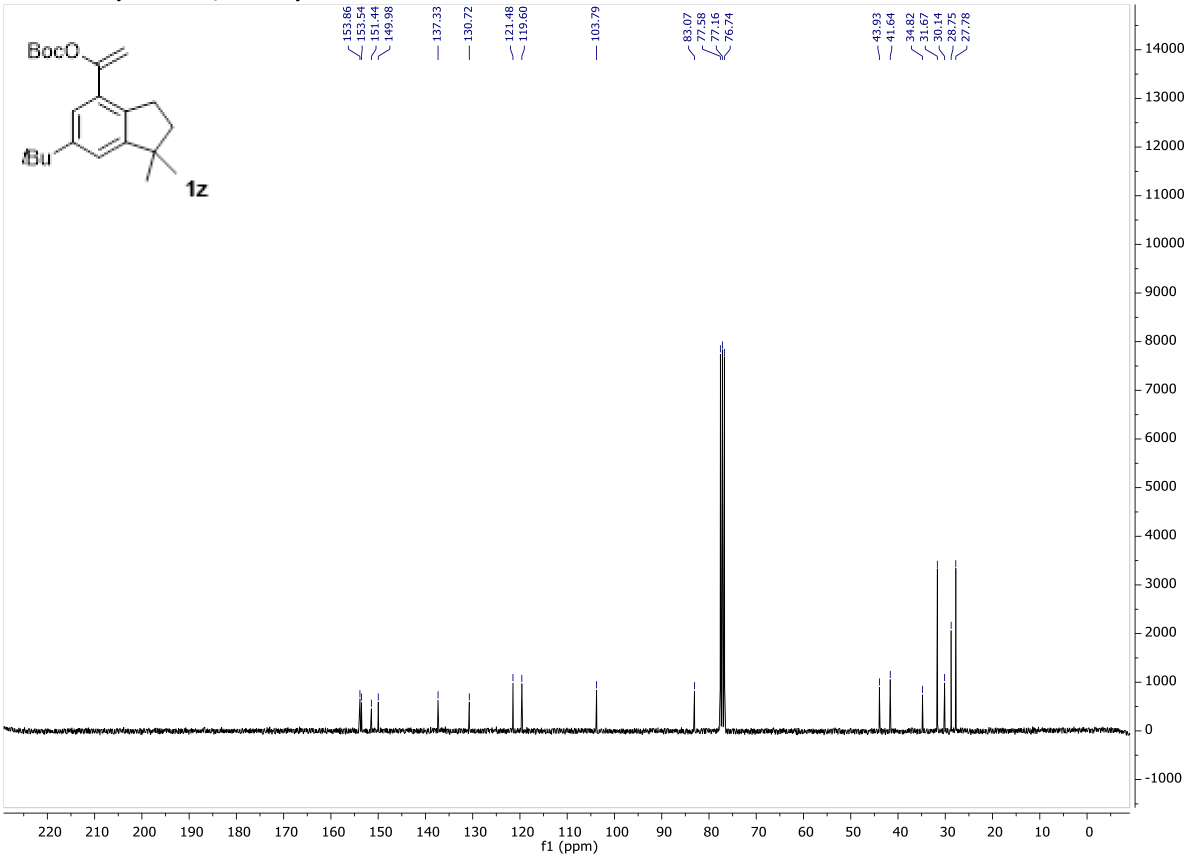

(E)-tert-butyl (4-(2,6,6-trimethylcyclohex-2-en-1-yl)buta-1,3-dien-2-yl) carbonate 1aa

^{1}H NMR (300 MHz, $CDCl_3$)

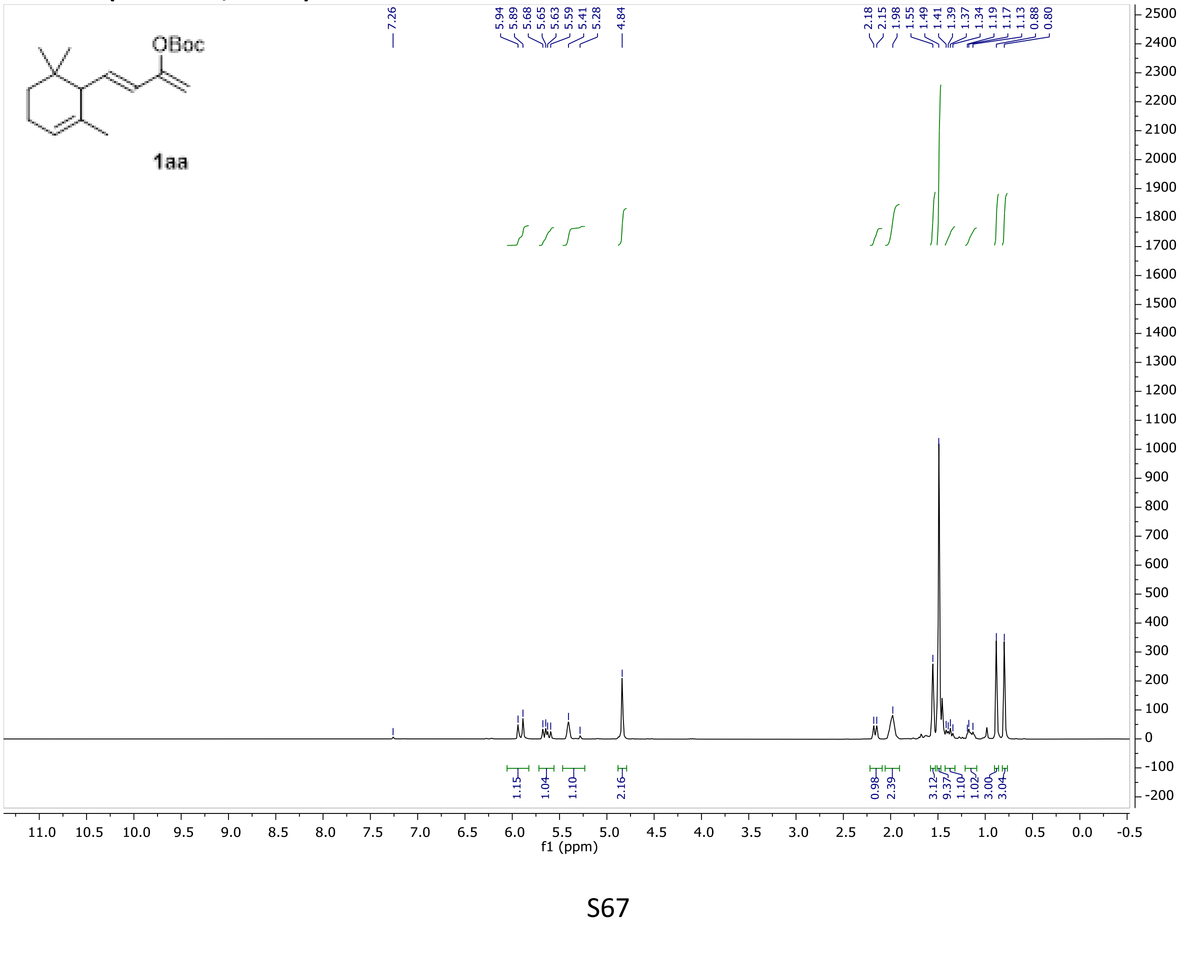

^{13}C NMR (75 MHz, CDCl$_3$)

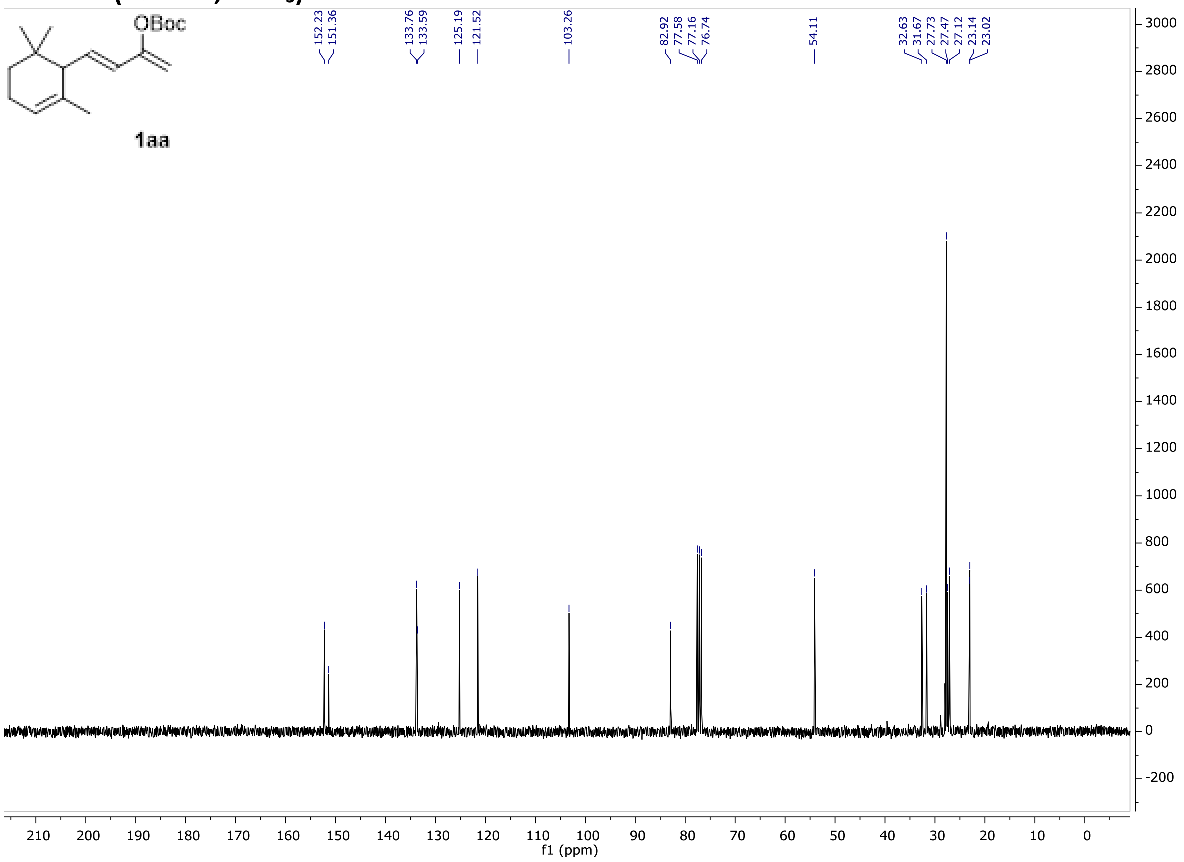

tert-butyl (1-(3-methoxy-10,13-dimethyl-2,3,4,7,8,9,10,11,12,13,14,15,16,17-tetradecahydro-1H-cyclopenta[a]phenanthren-17-yl)vinyl) carbonate 1ab

^{1}H NMR (500 MHz, CDCl$_3$)

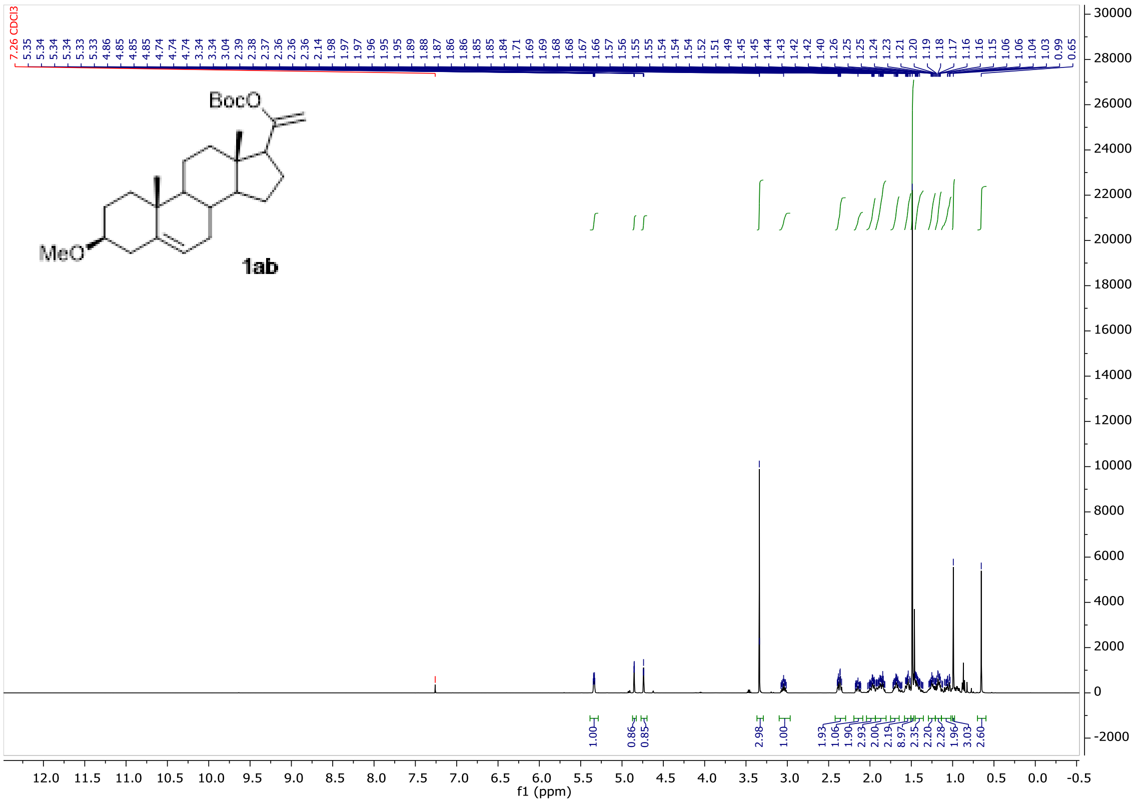

^{13}C NMR (126 MHz, $CDCl_3$)

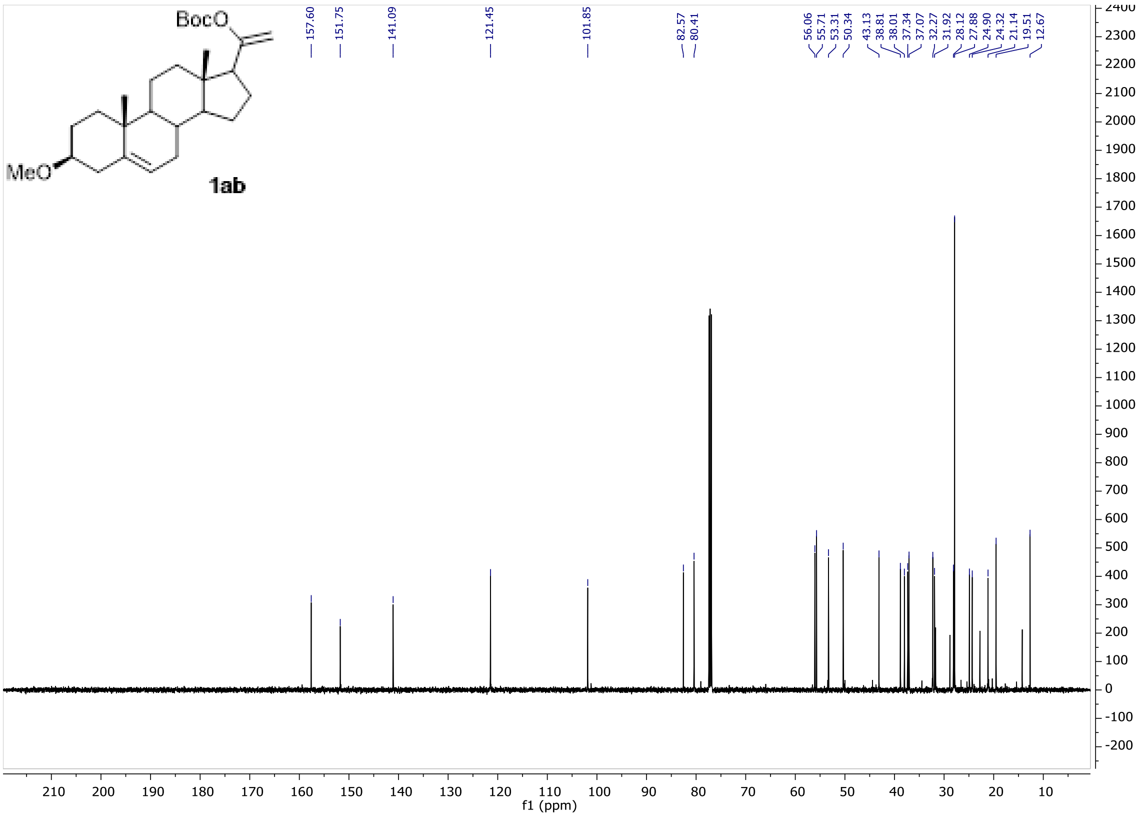

tert-butyl (-17-methoxy-10,13-dimethyl-2,7,8,9,10,11,12,13,14,15,16,17-dodecahydro-1H-cyclopenta[a]phenanthren-3-yl) carbonate 1ac

^{1}H NMR (300 MHz, $CDCl_3$)

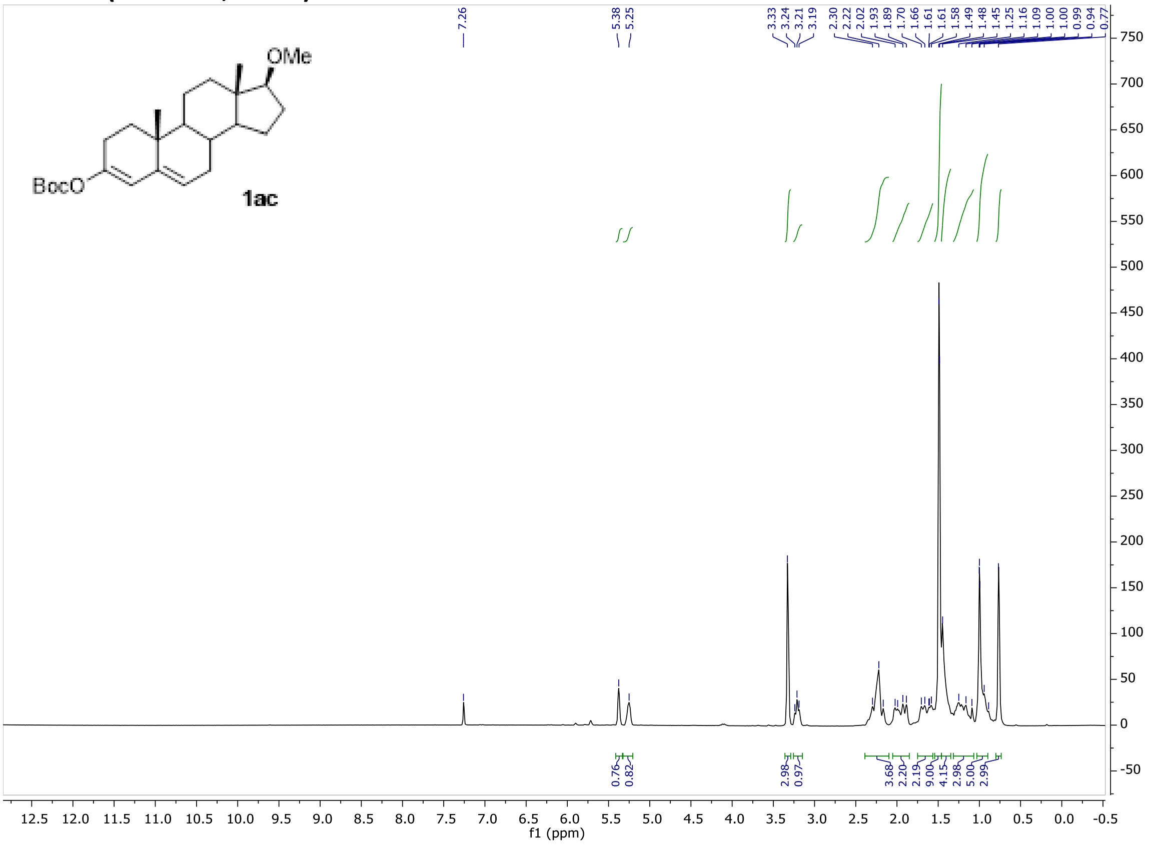

^{13}C NMR (75 MHz, CDCl$_3$)

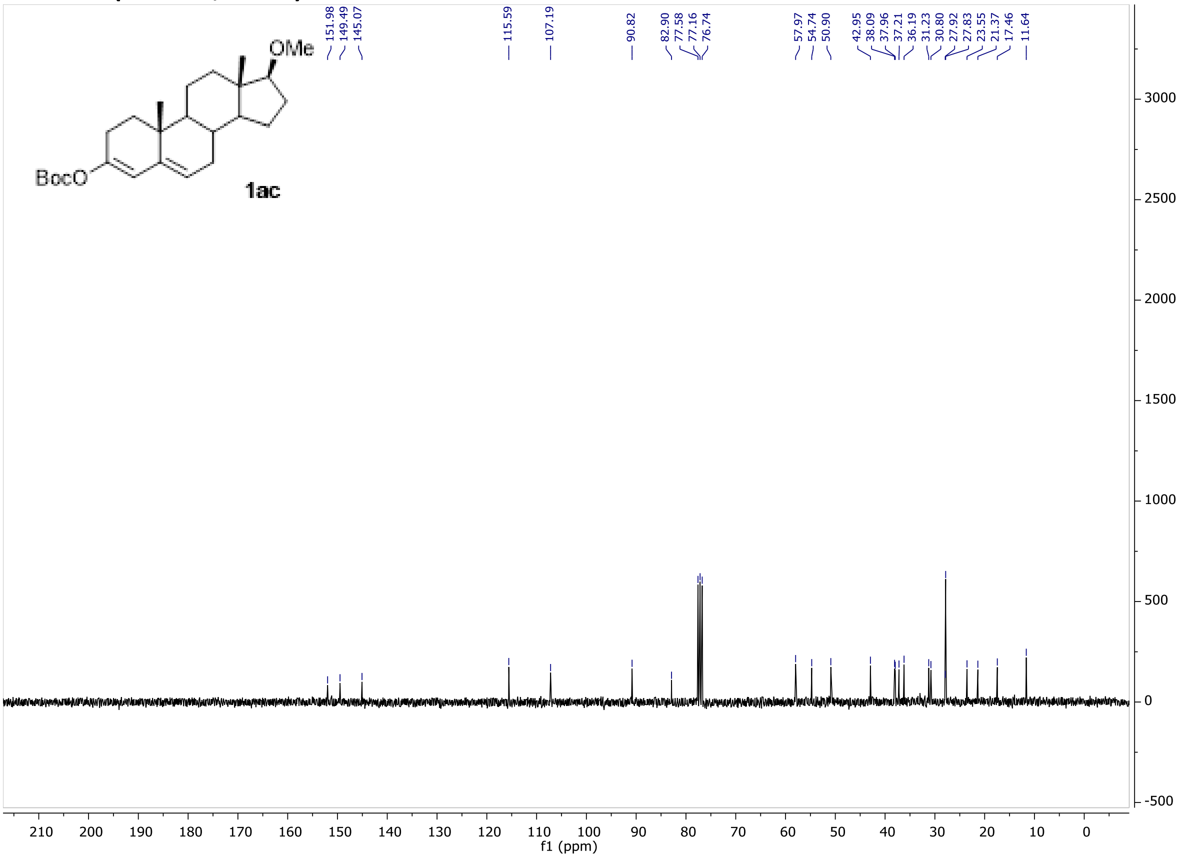

1-(4-fluorophenyl)-2-(trifluoromethoxy)ethanone 3

^{1}H NMR (300 MHz, CDCl$_3$)

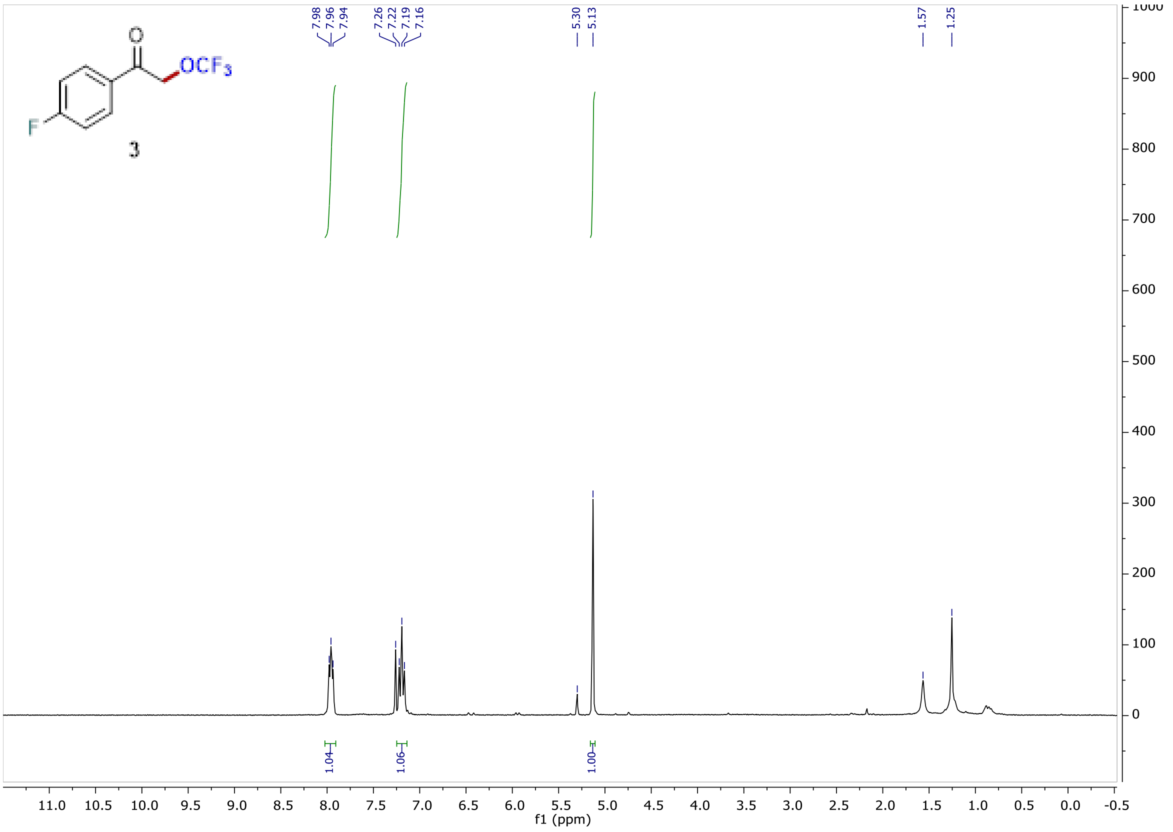

^{13}C NMR (75 MHz, $CDCl_3$)

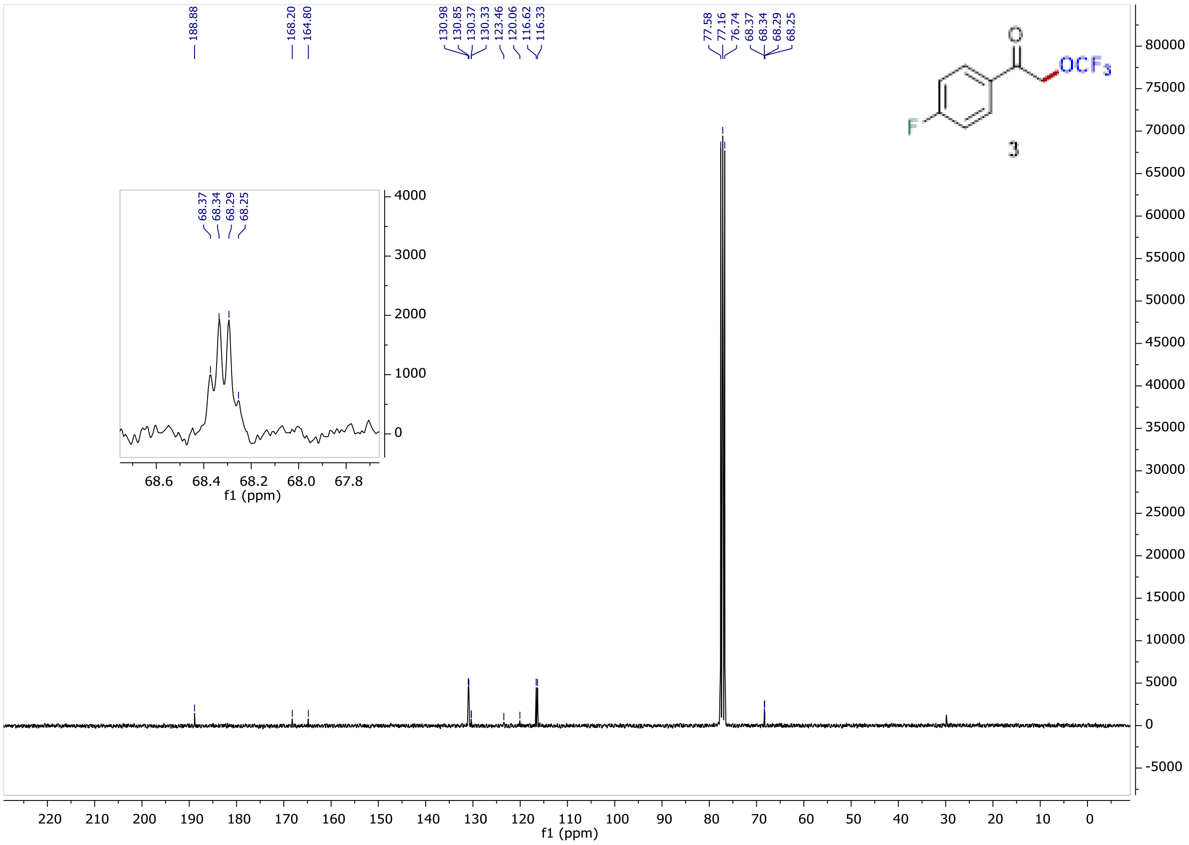

^{19}F NMR (188 MHz, $CDCl_3$)

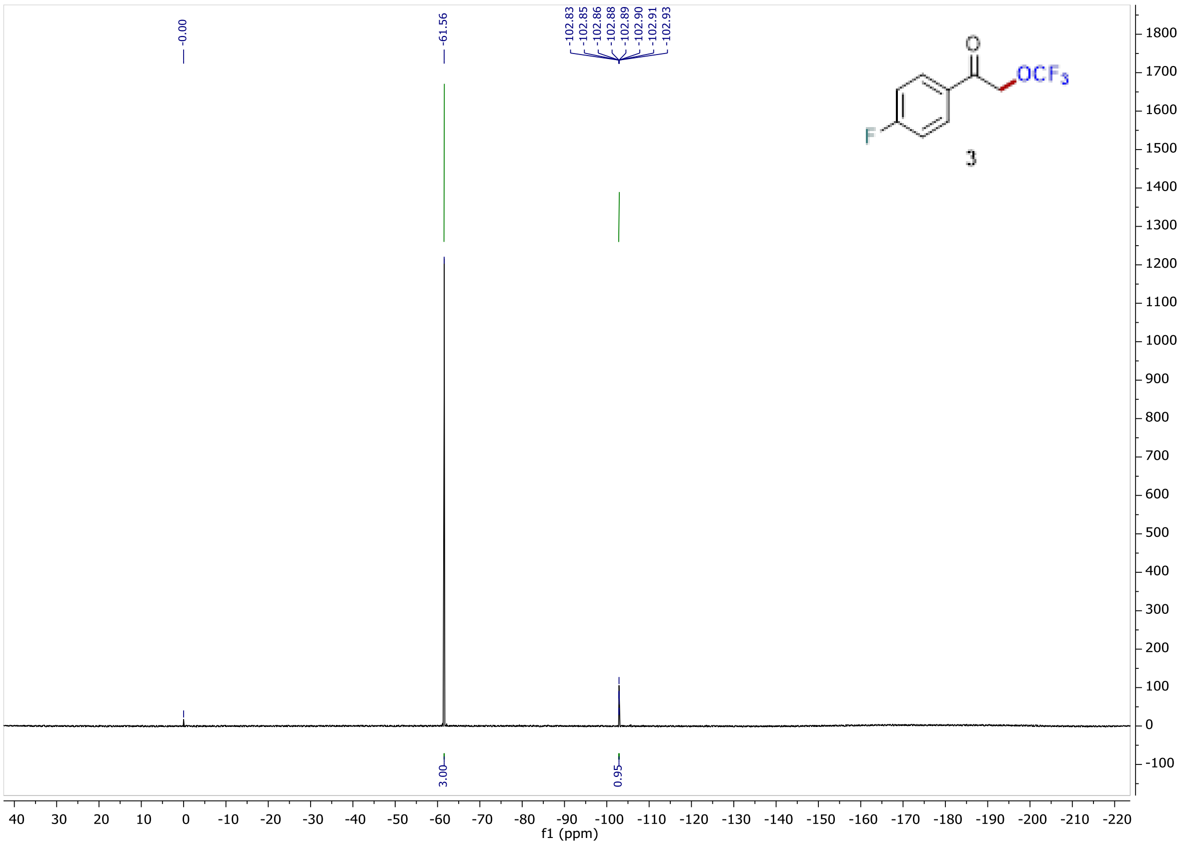

1-(*o*-tolyl)-2-(trifluoromethoxy)ethanone 7

^{1}H NMR (300 MHz, $CDCl_3$)

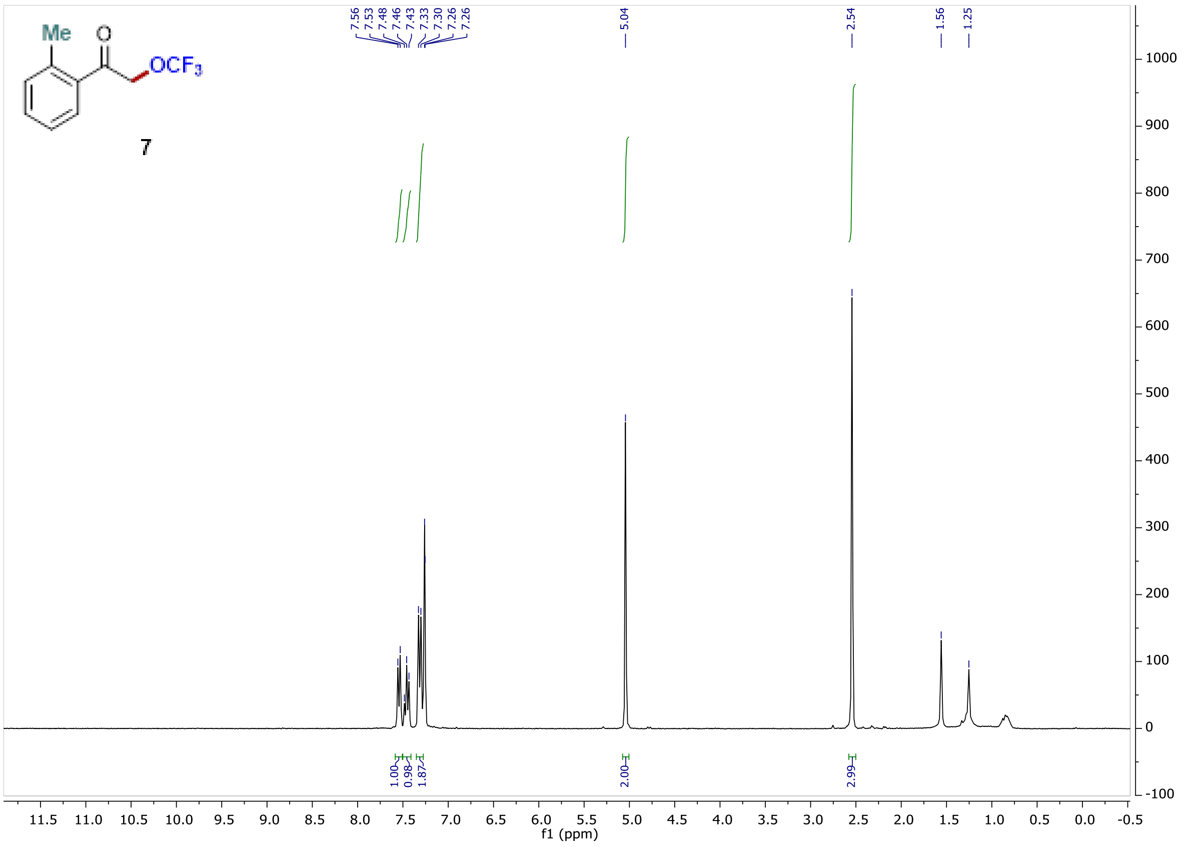

^{13}C NMR (75 MHz, $CDCl_3$)

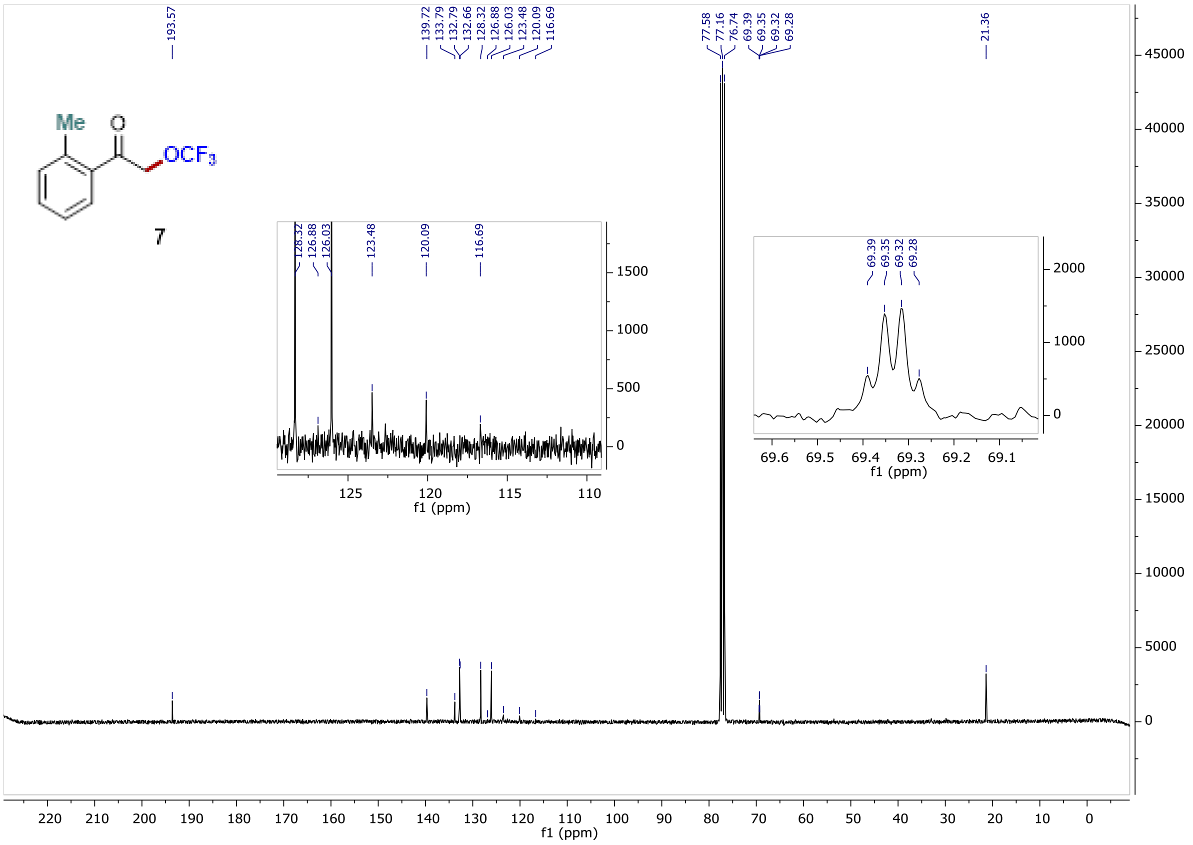

^{19}F NMR (188 MHz, $CDCl_3$)

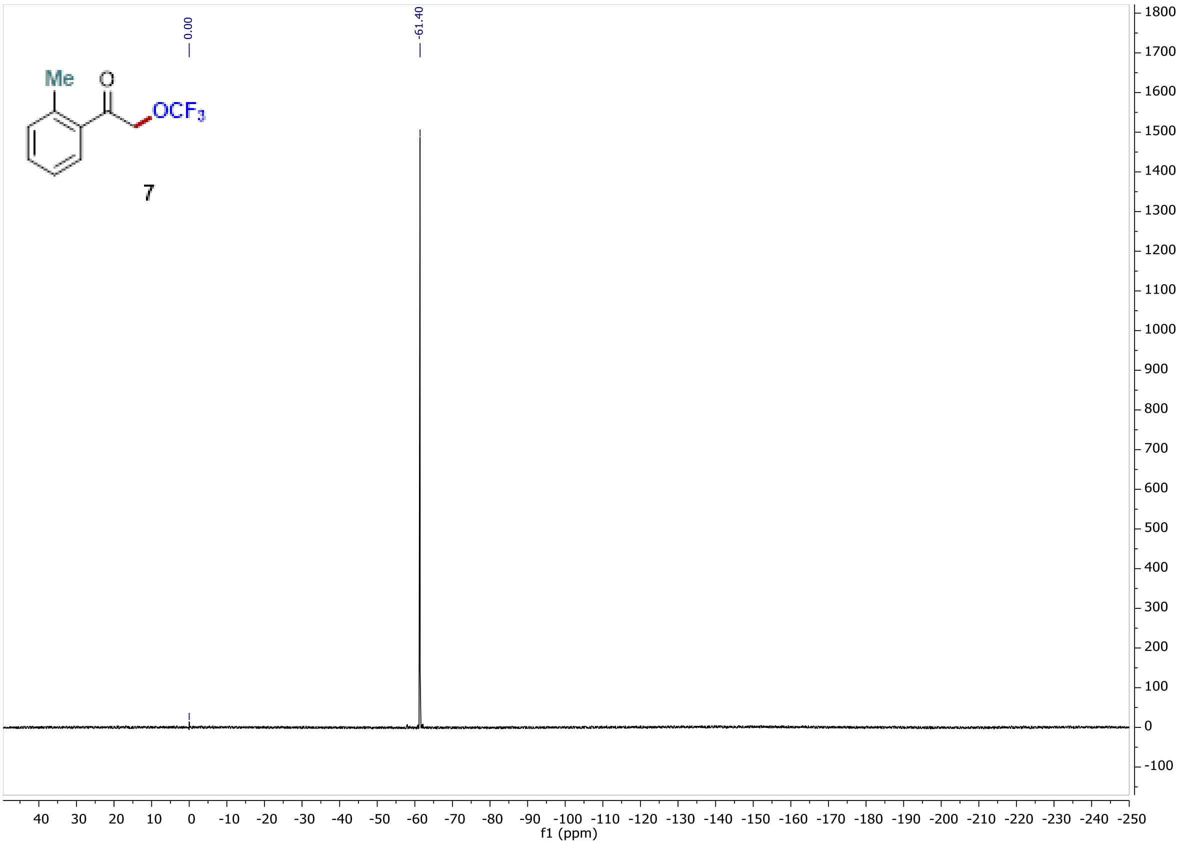

1-(*m*-tolyl)-2-(trifluoromethoxy)ethanone 8

^{1}H NMR (300 MHz, $CDCl_3$)

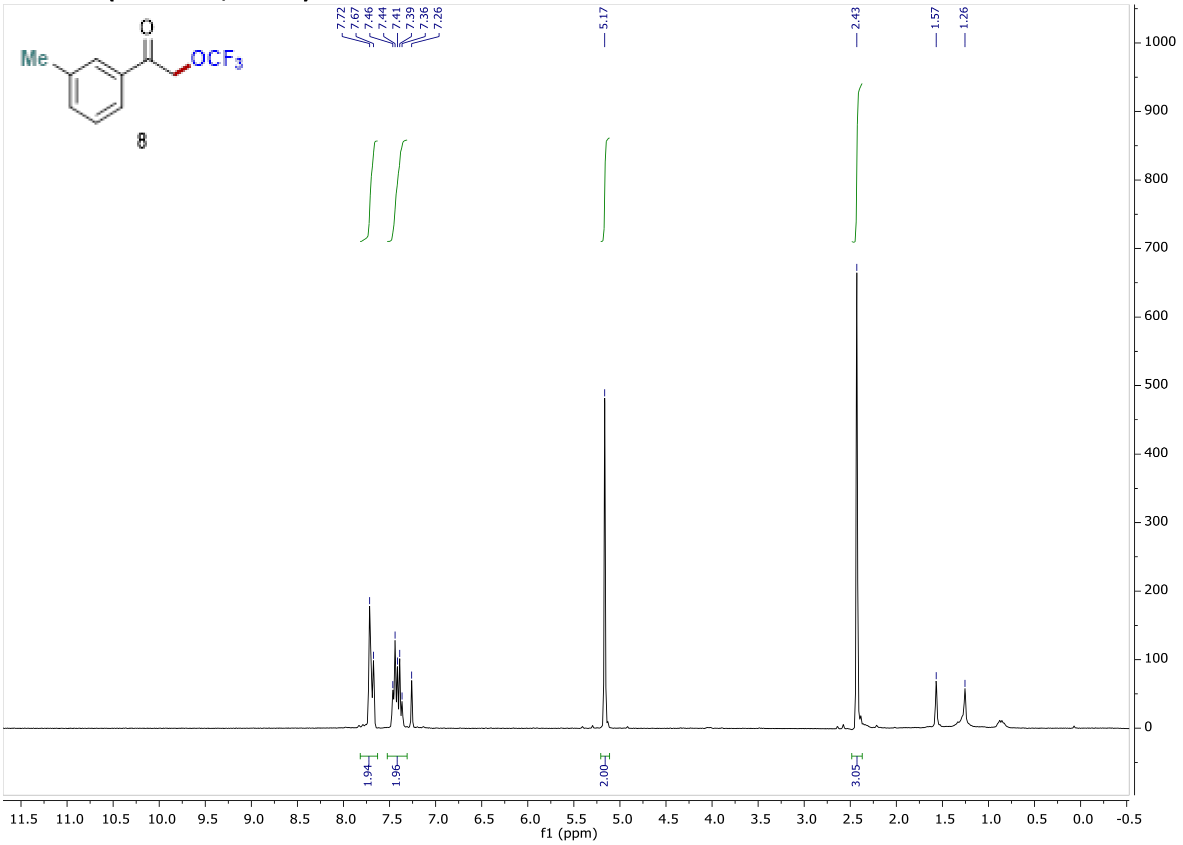

^{13}C NMR (75 MHz, CDCl₃)

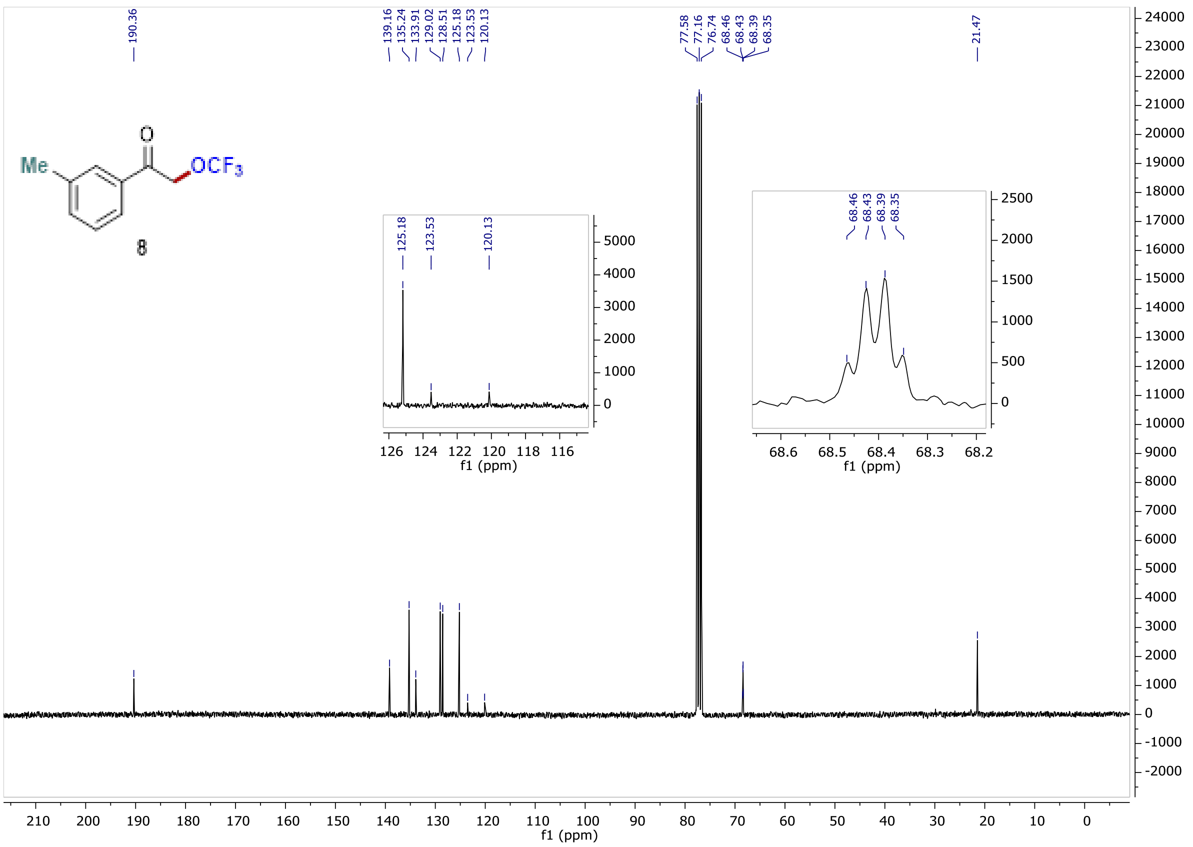

^{19}F NMR (188 MHz, CDCl₃)

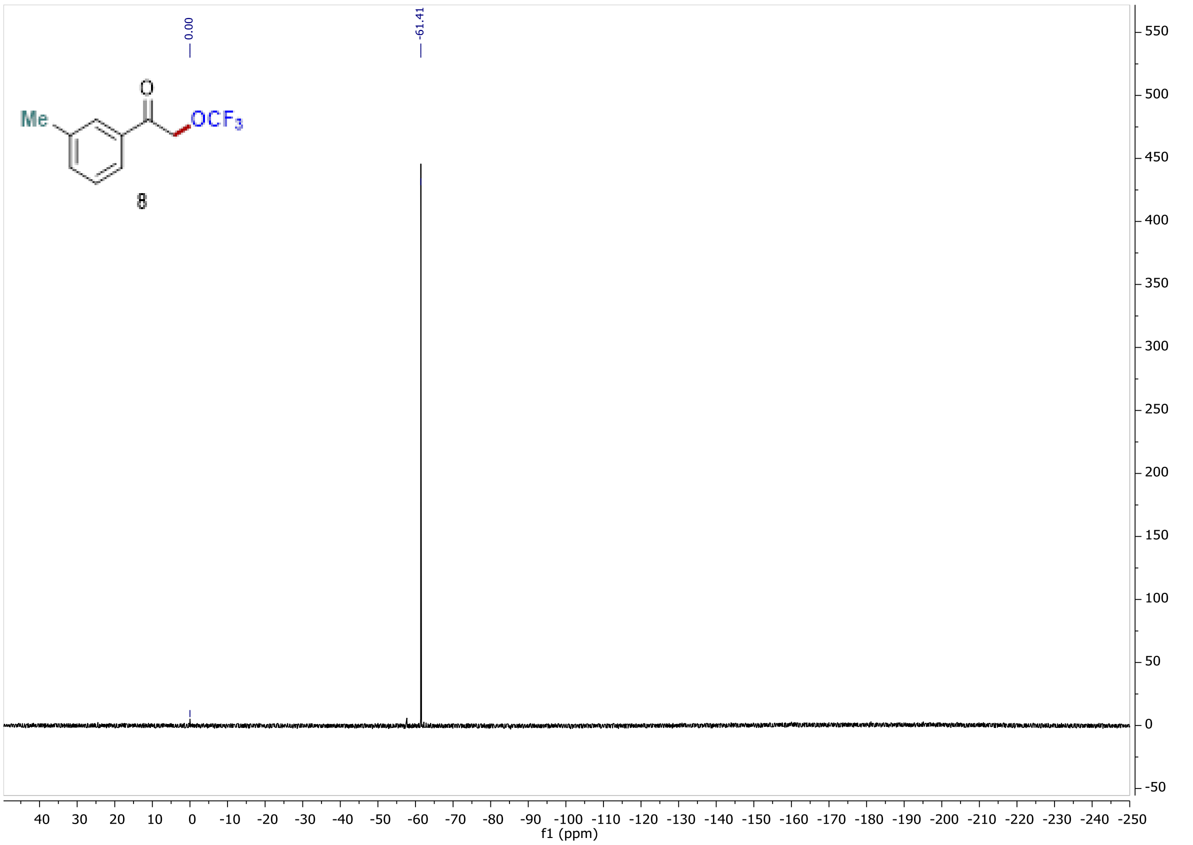

1-(*p*-tolyl)-2-(trifluoromethoxy)ethanone 9

^{1}H NMR (300 MHz, $CDCl_3$)

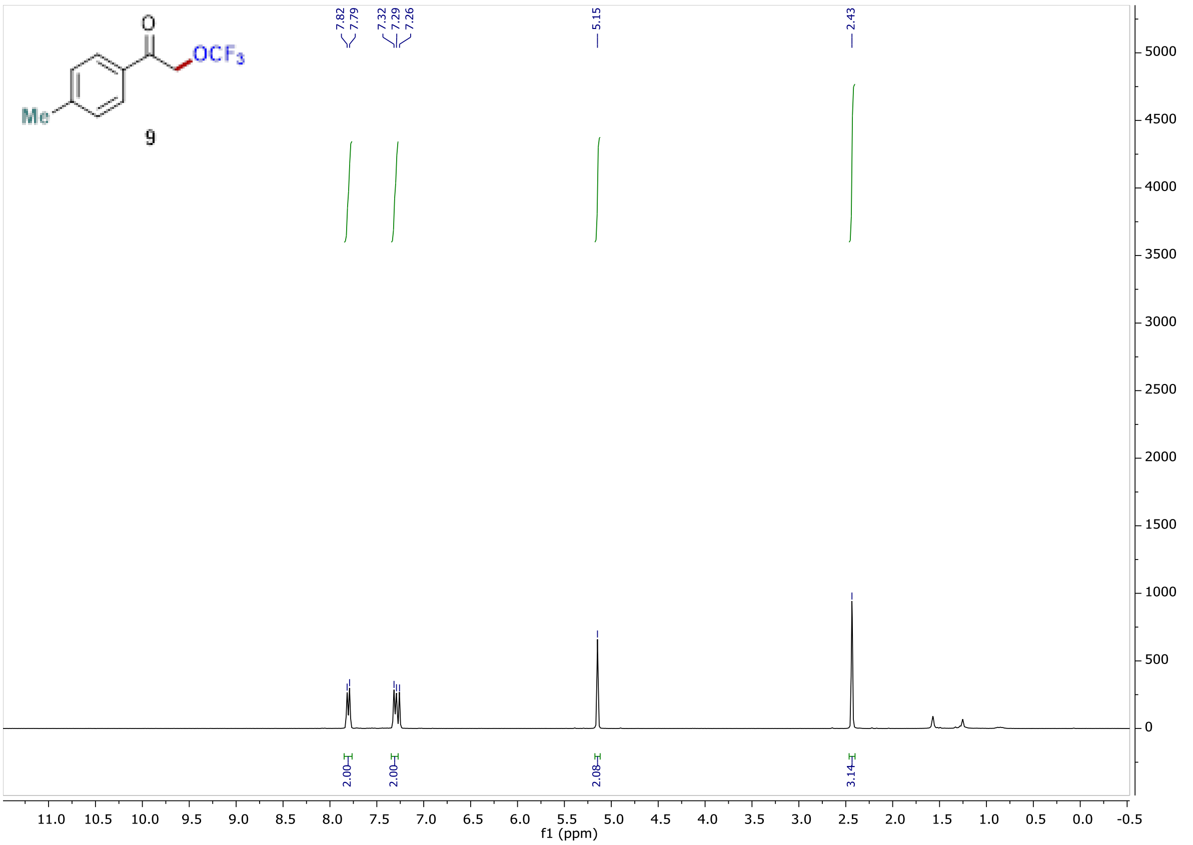

^{13}C NMR (75 MHz, $CDCl_3$)

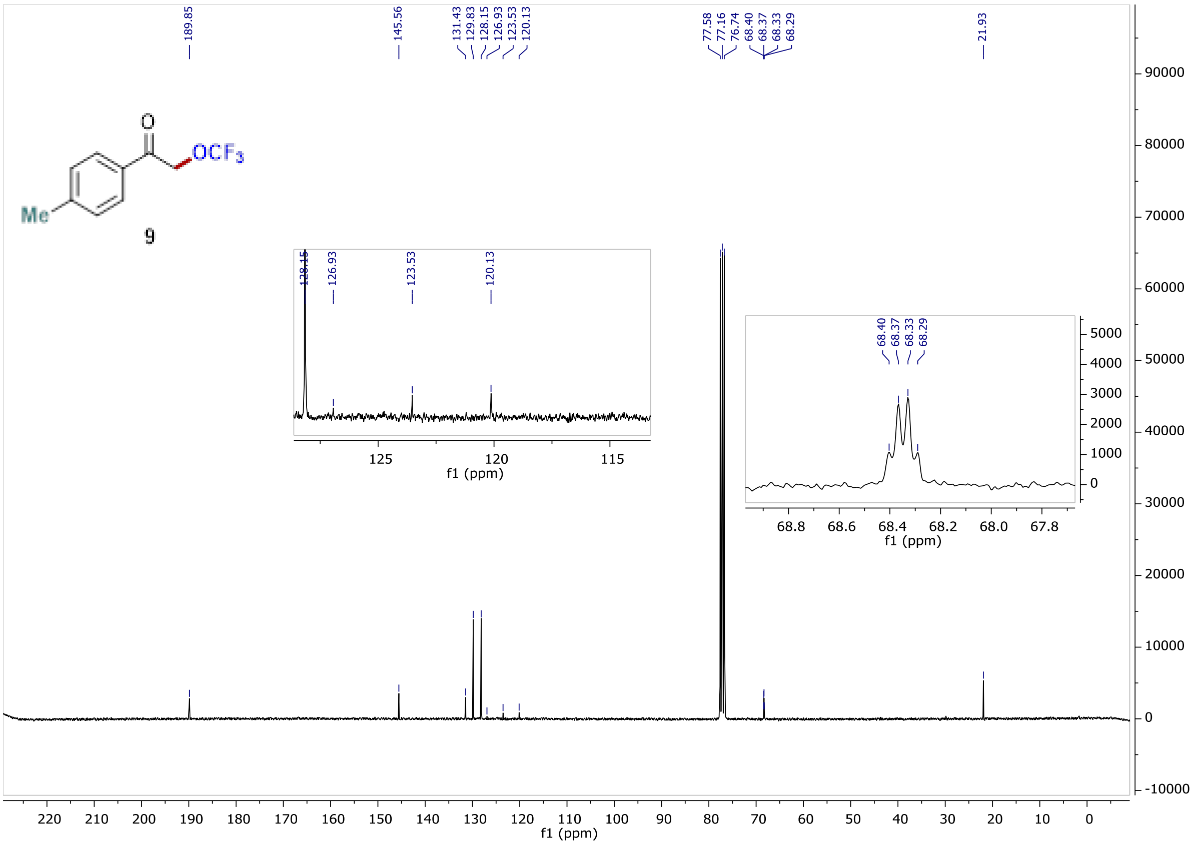

^{19}F NMR (188 MHz, $CDCl_3$)

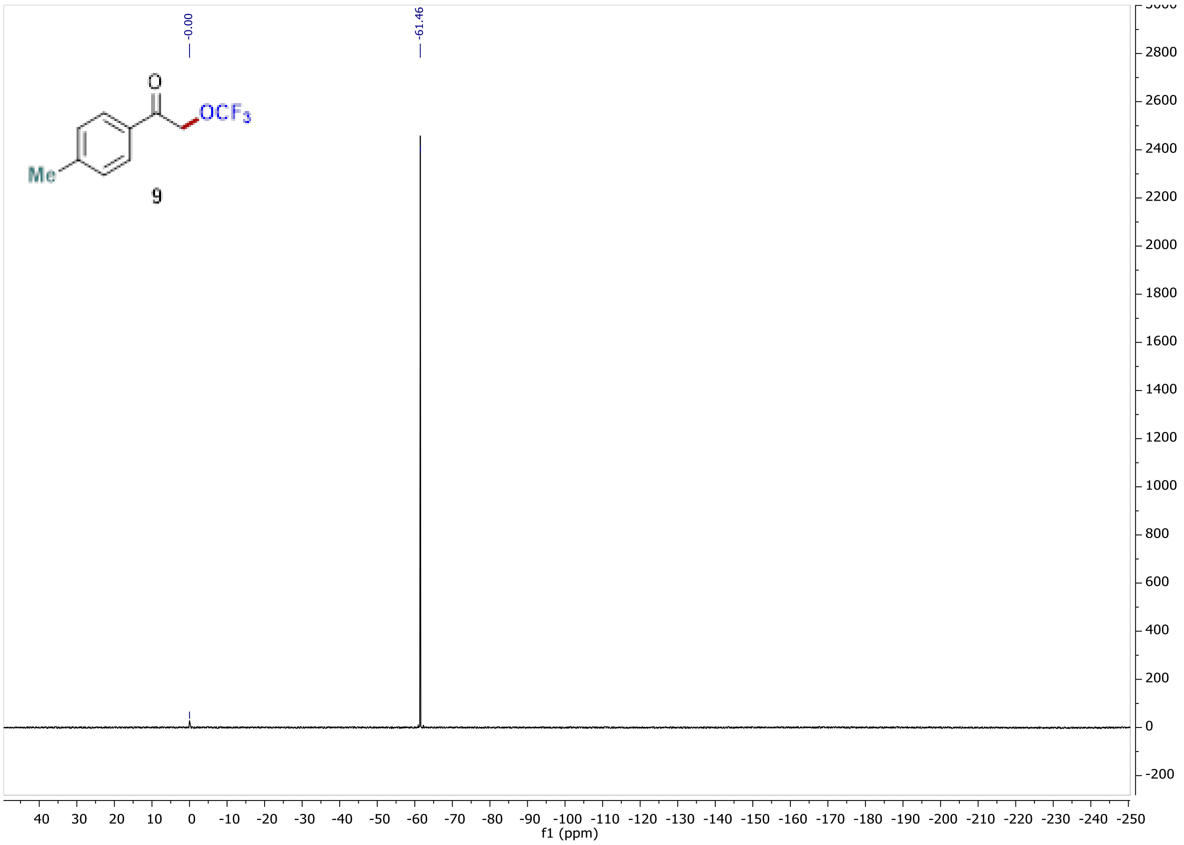

4-(2-(trifluoromethoxy)acetyl)benzonitrile 10

^1H NMR (300 MHz, $CDCl_3$)

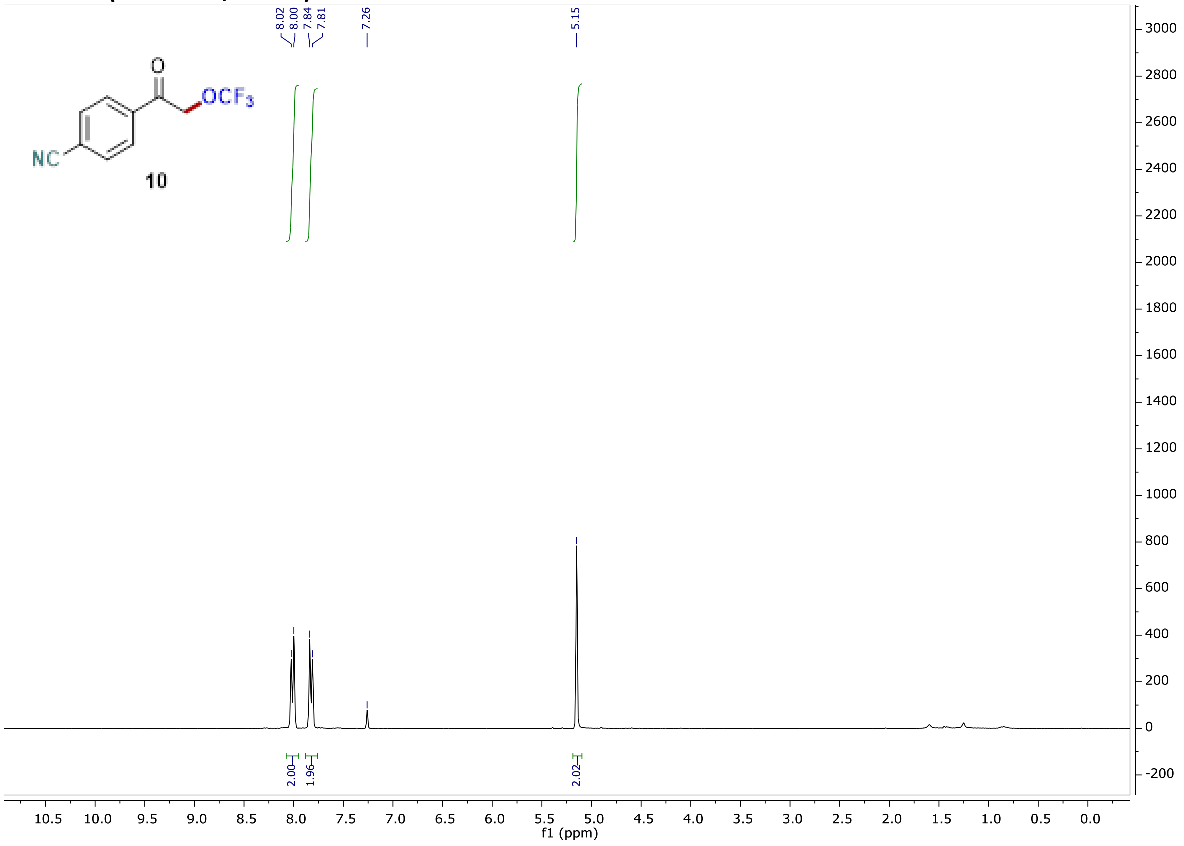

^{13}C NMR (75 MHz, $CDCl_3$)

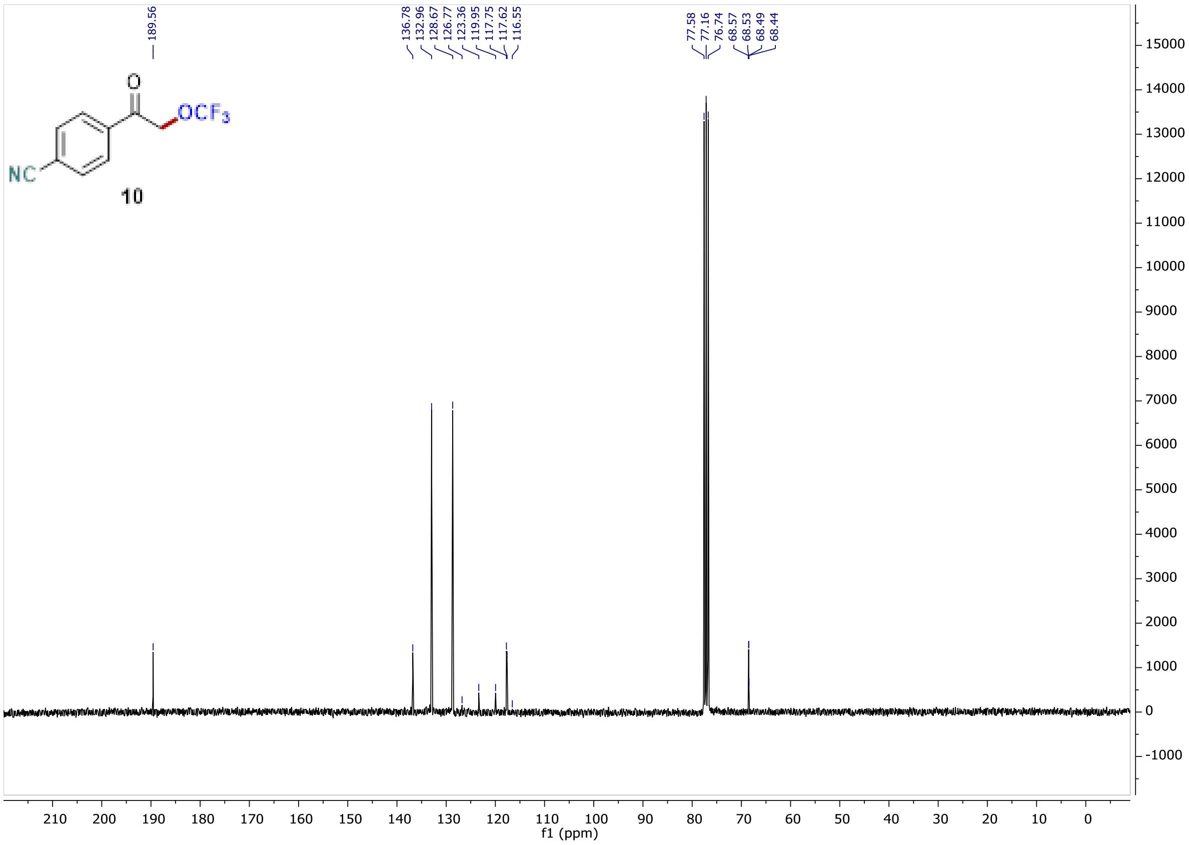

^{19}F NMR (188 MHz, $CDCl_3$)

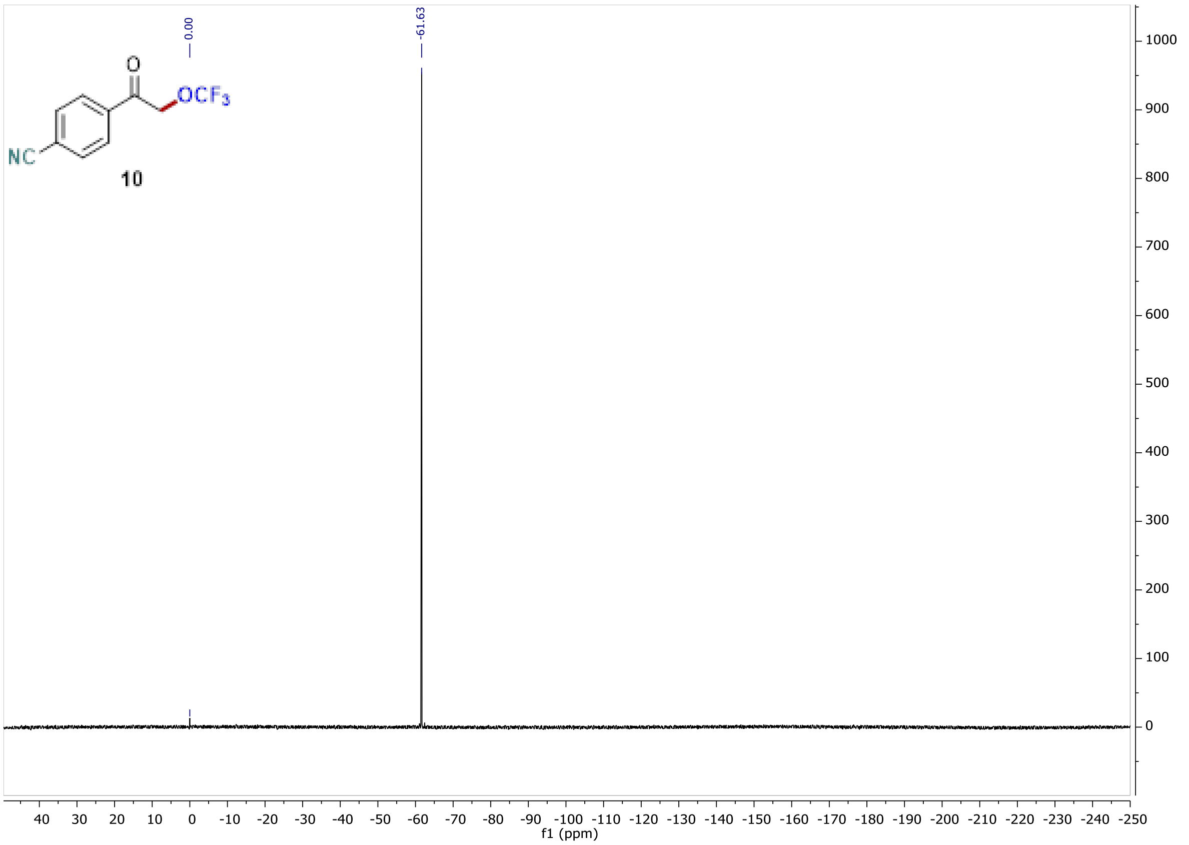

2-(trifluoromethoxy)-1-(3-(trifluoromethyl)phenyl)ethanone 12

^{1}H NMR (300 MHz, $CDCl_3$)

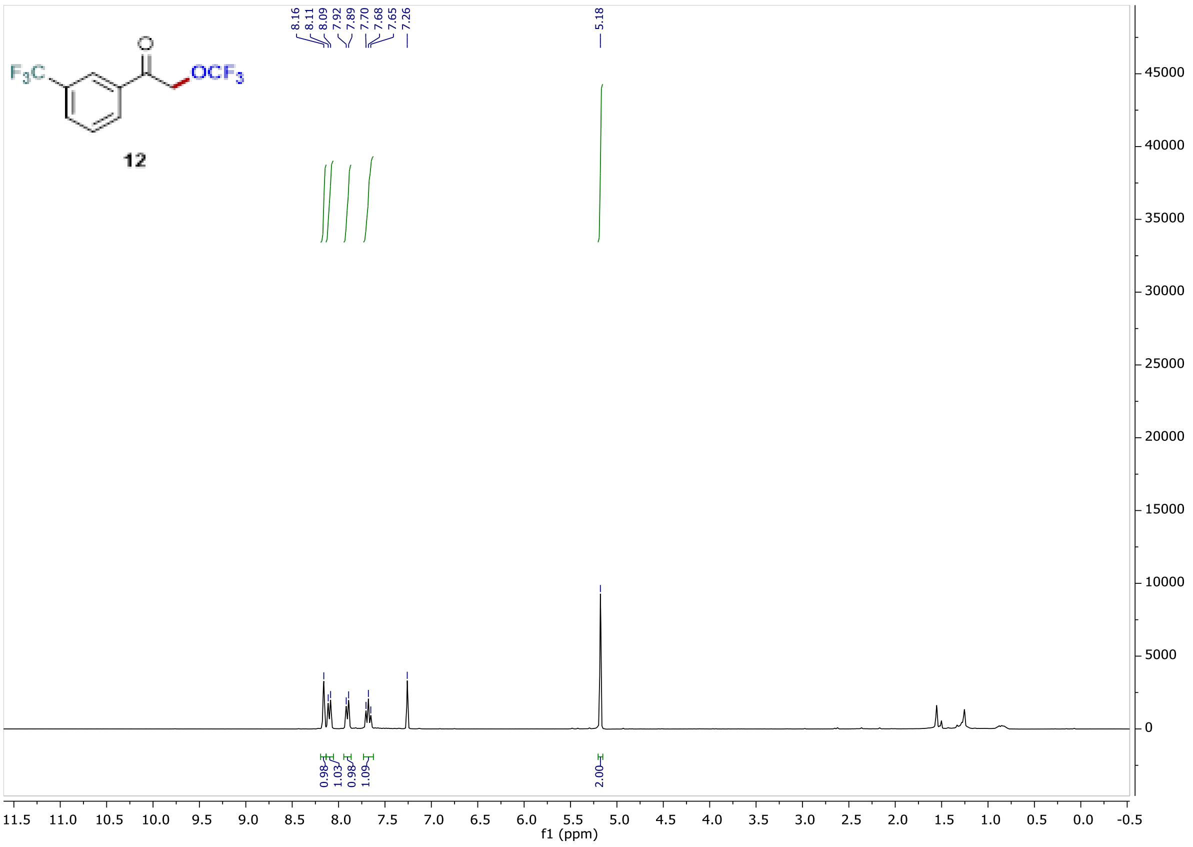

^{13}C NMR (75 MHz, $CDCl_3$)

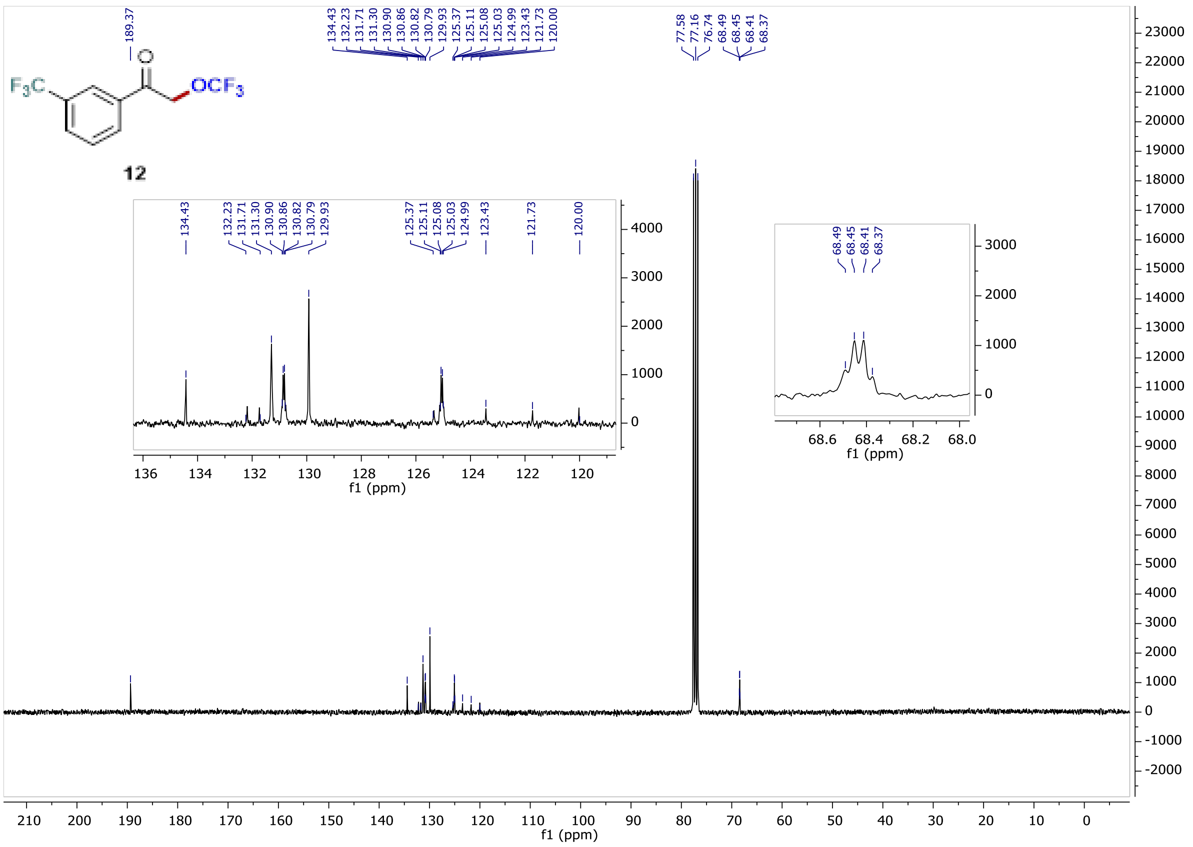

^{19}F NMR (188 MHz, $CDCl_3$)

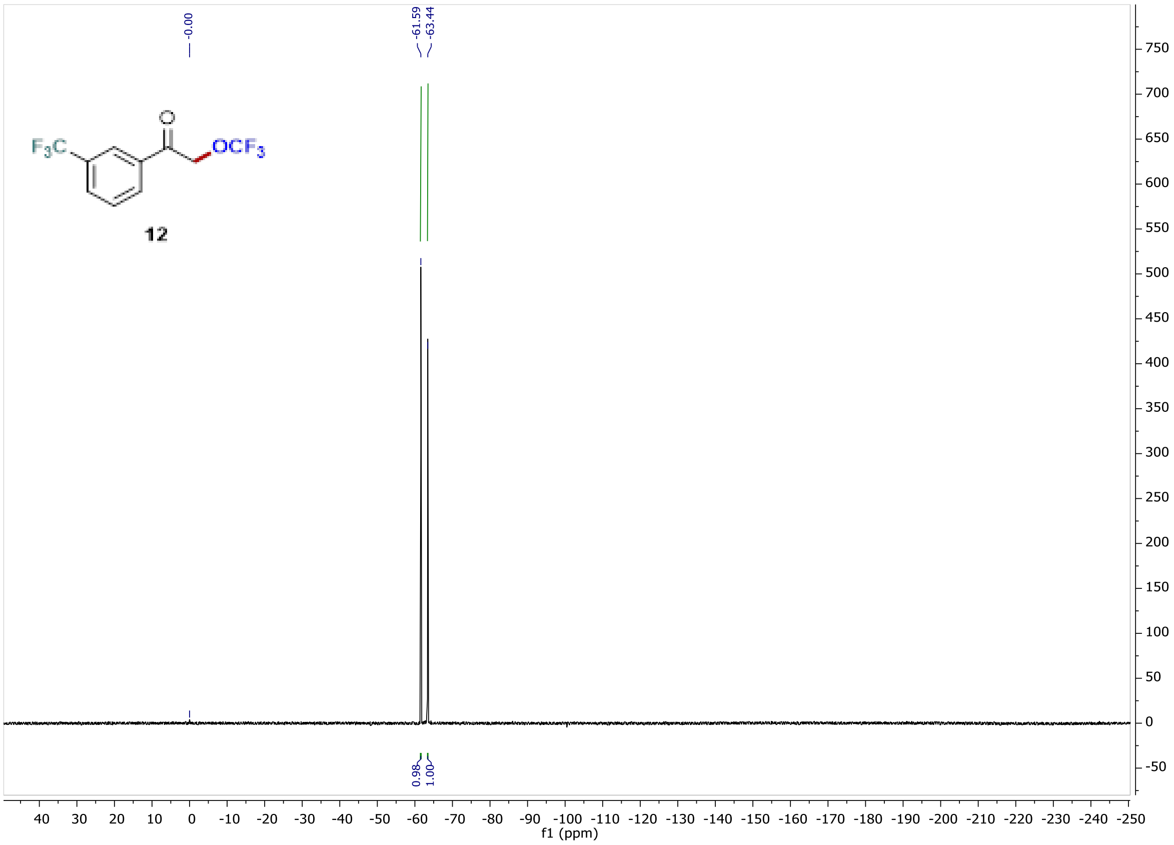

1-(2-bromophenyl)-2-(trifluoromethoxy)ethan-1-one 14

^{1}H NMR (500 MHz, $CDCl_3$)

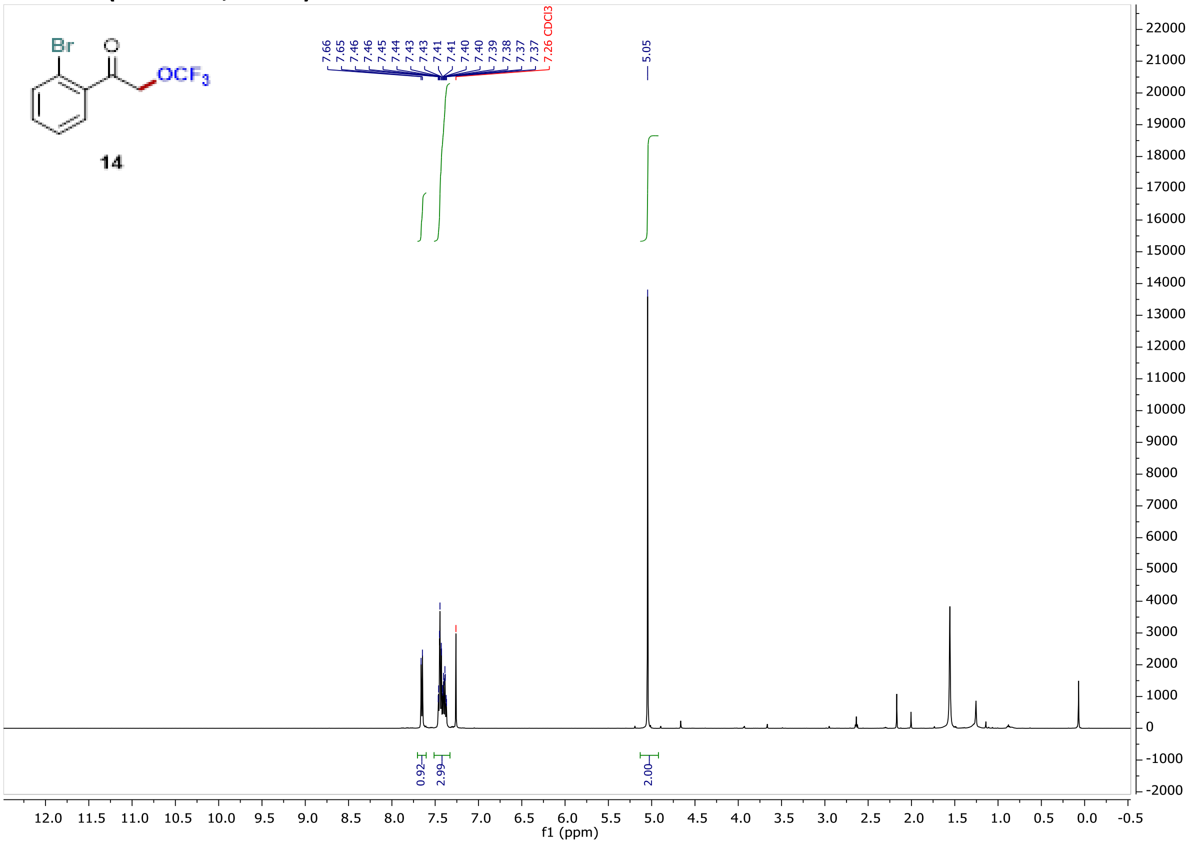

^{13}C NMR (126 MHz, $CDCl_3$)

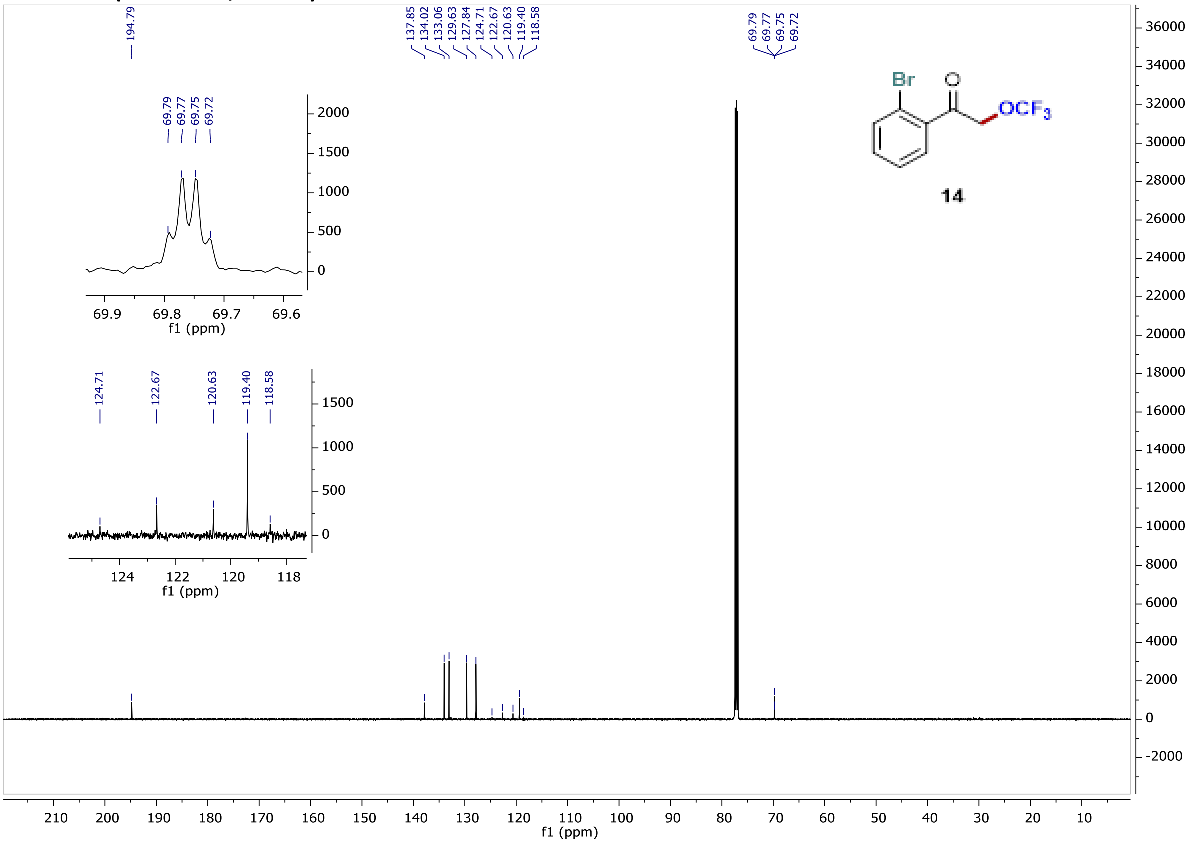

^{19}F NMR (188 MHz, $CDCl_3$)

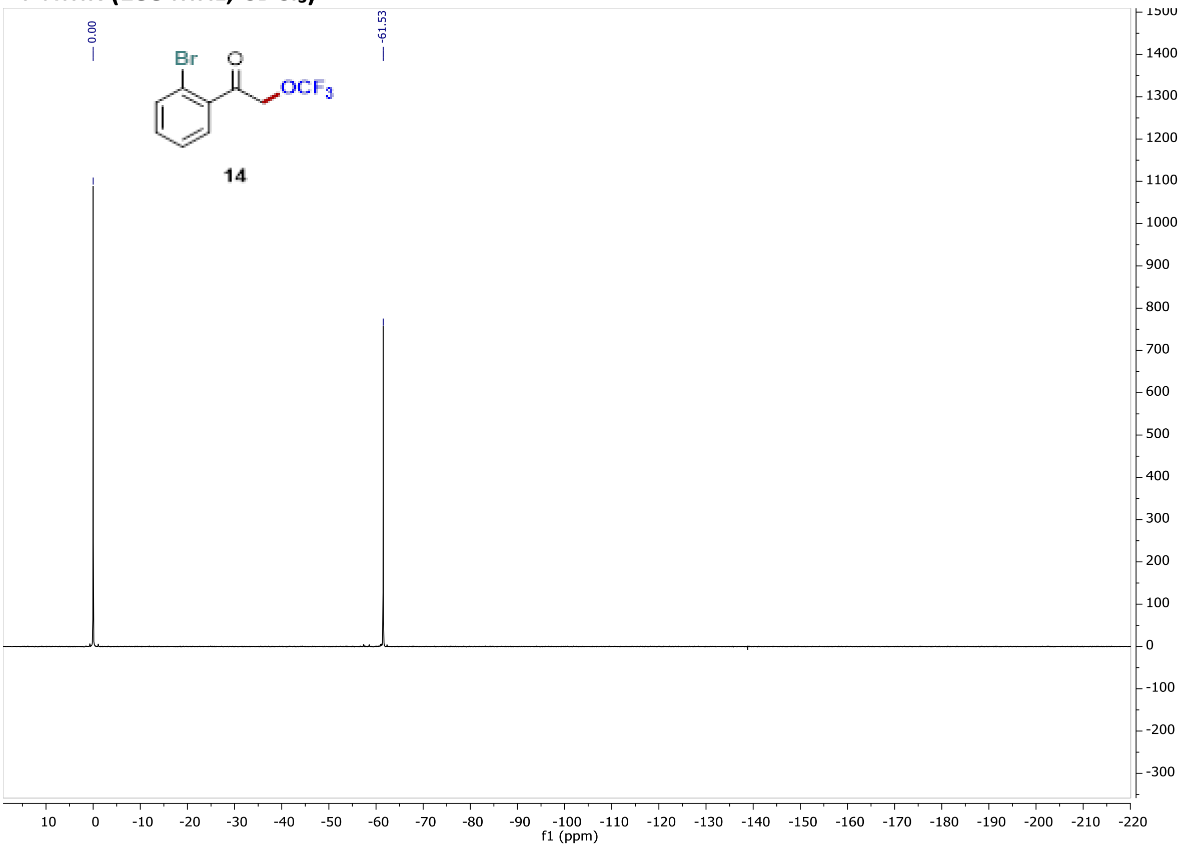

1-(3-bromophenyl)-2-(trifluoromethoxy)ethan-1-one 15

^{1}H NMR (500 MHz, $CDCl_3$)

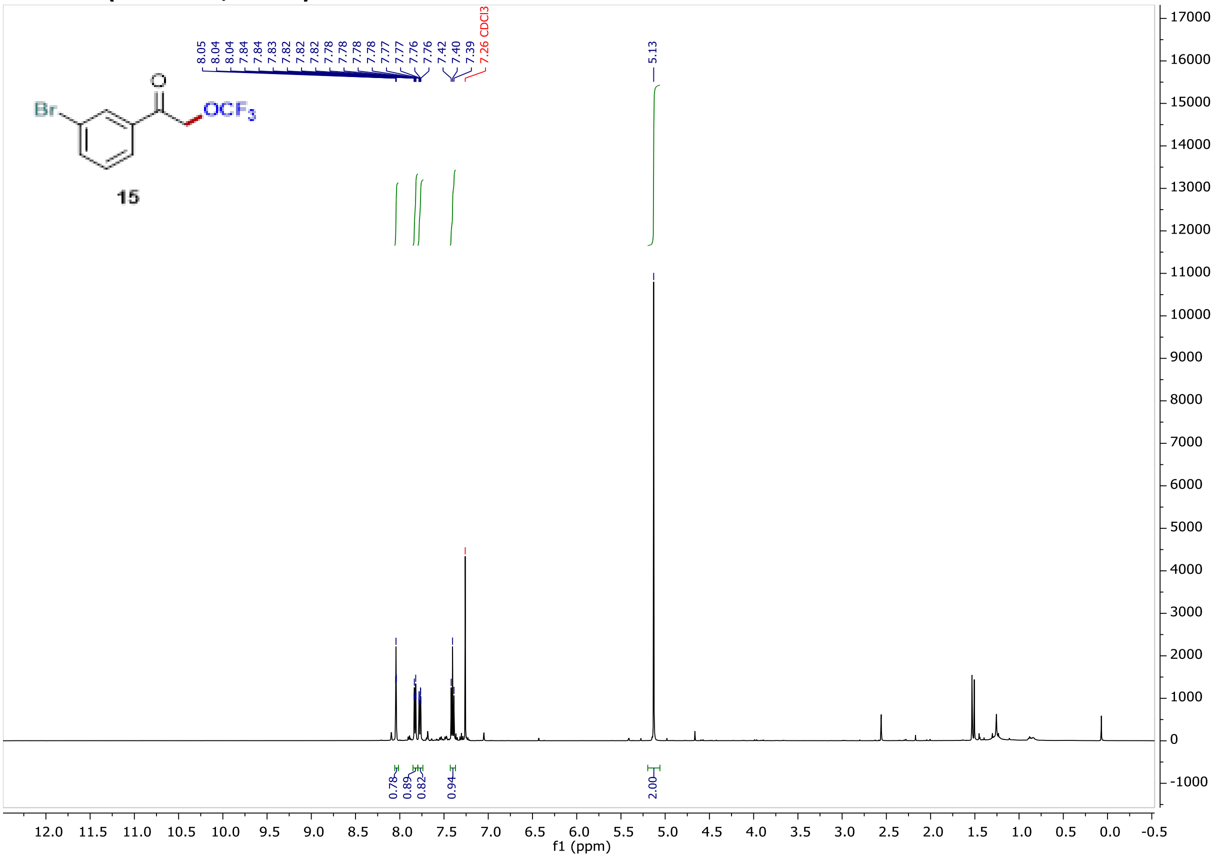

^{13}C NMR (126 MHz, $CDCl_3$)

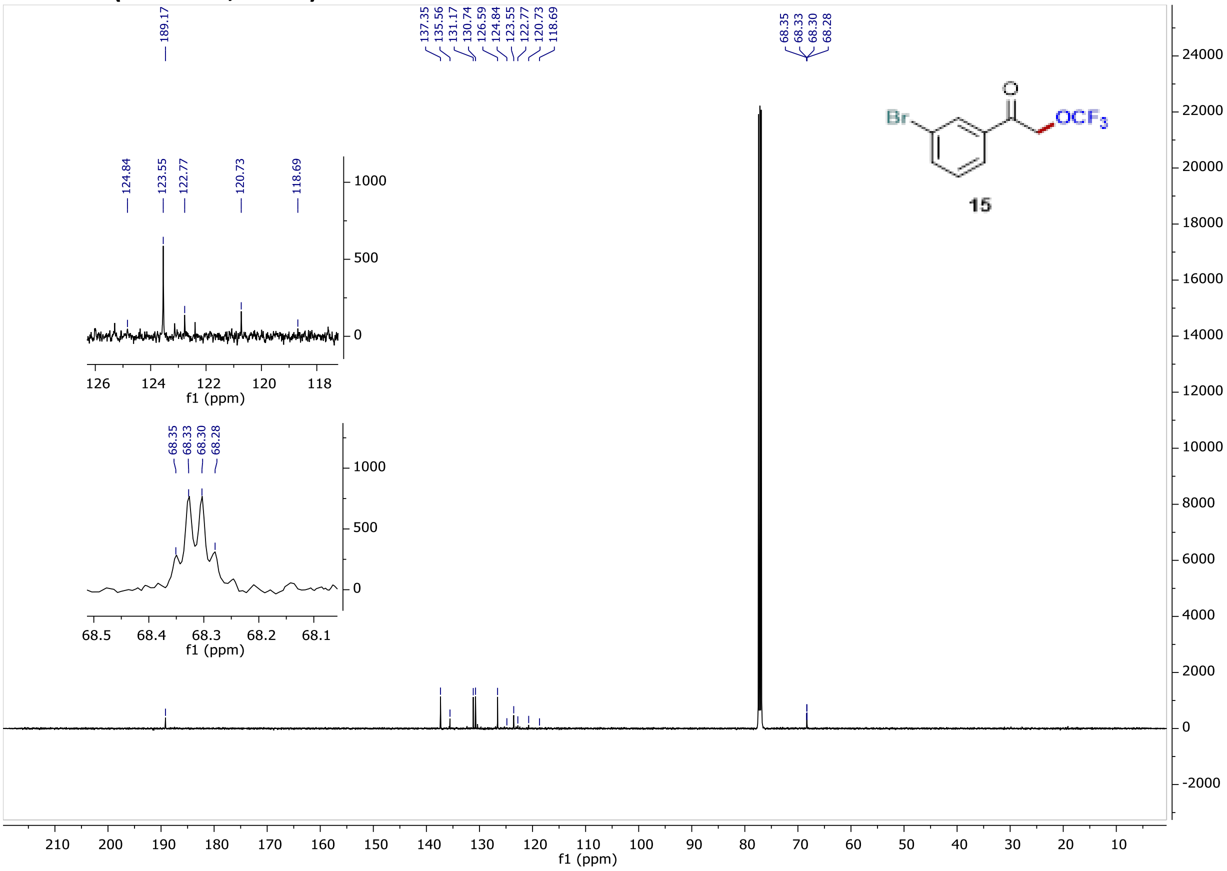

^{19}F NMR (188 MHz, $CDCl_3$)

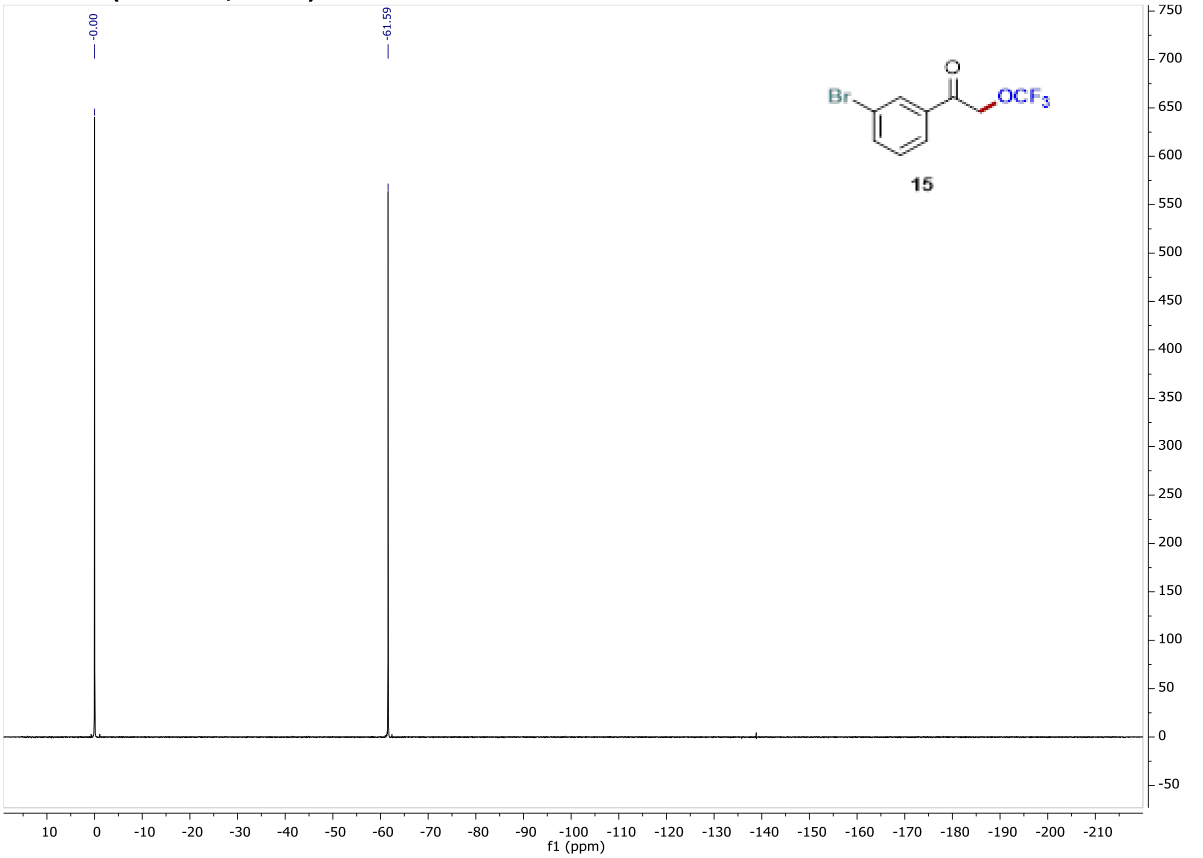

1-(4-bromophenyl)-2-(trifluoromethoxy)ethan-1-one 16

^{1}H NMR (500 MHz, $CDCl_3$)

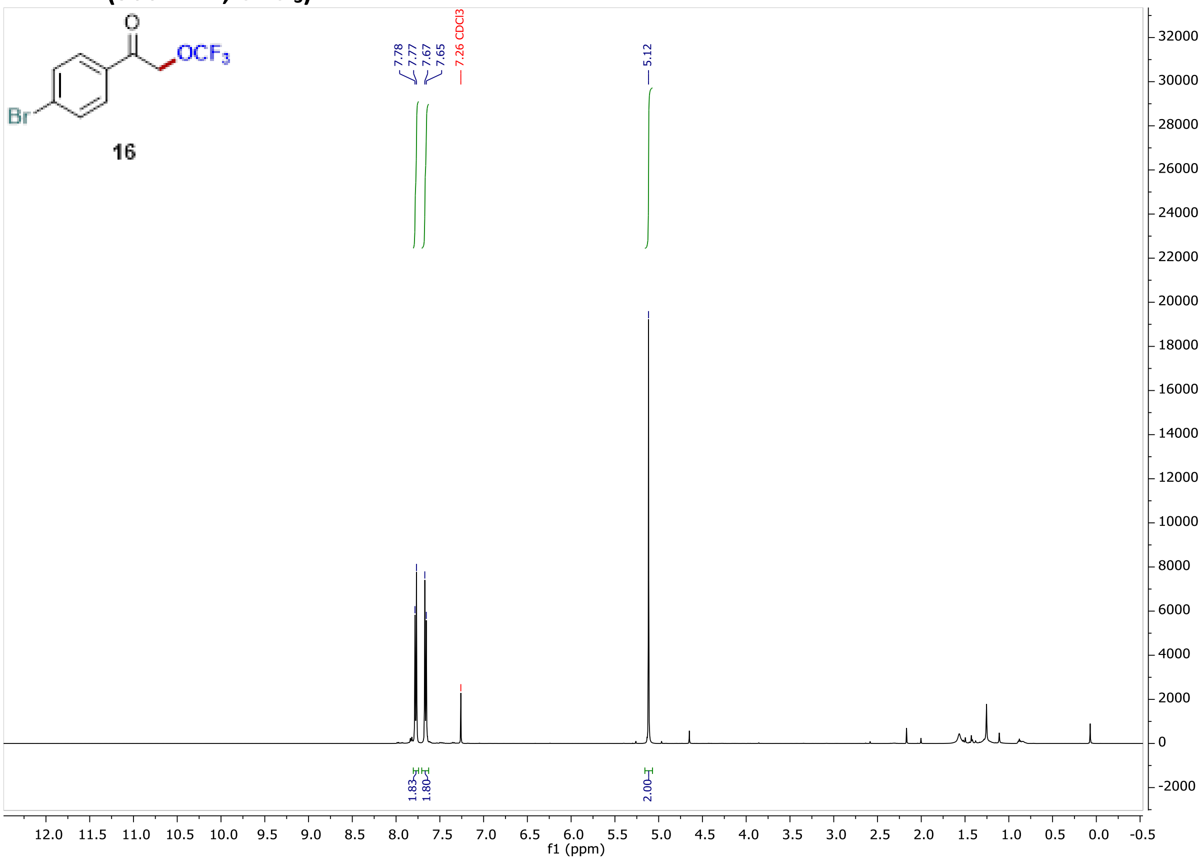

^{13}C NMR (126 MHz, $CDCl_3$)

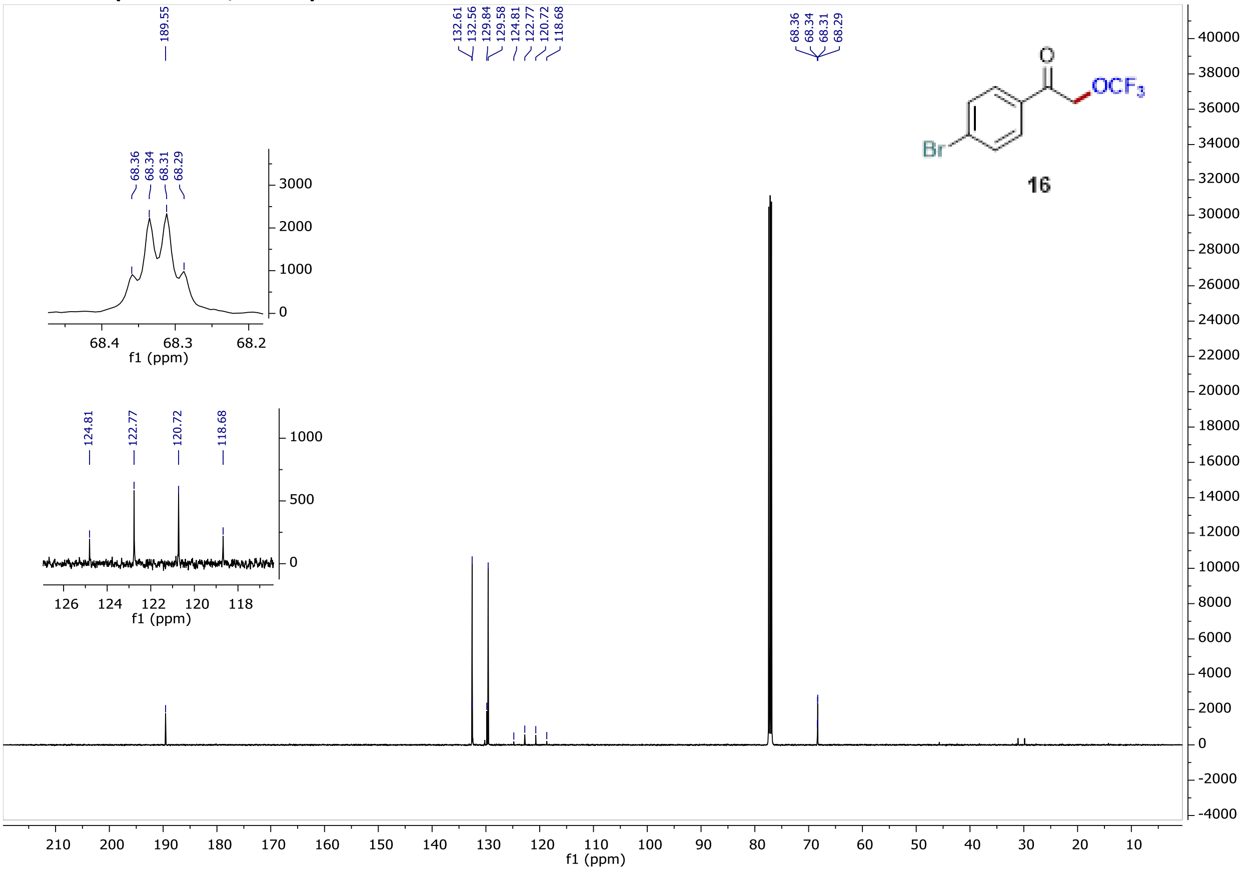

^{19}F NMR (188 MHz, $CDCl_3$)

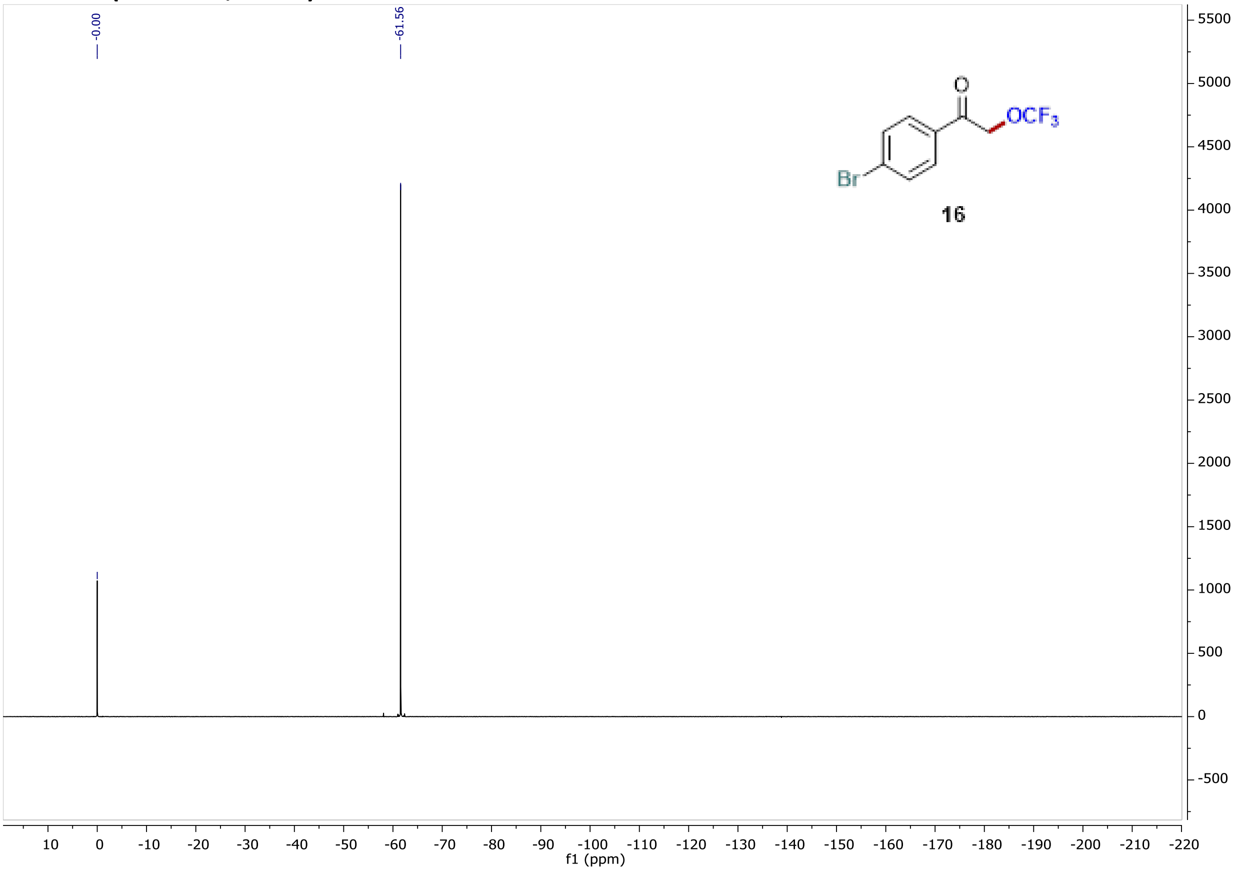

1-phenyl-2-(trifluoromethoxy)propan-1-one 17

^{1}H NMR (500 MHz, $CDCl_3$)

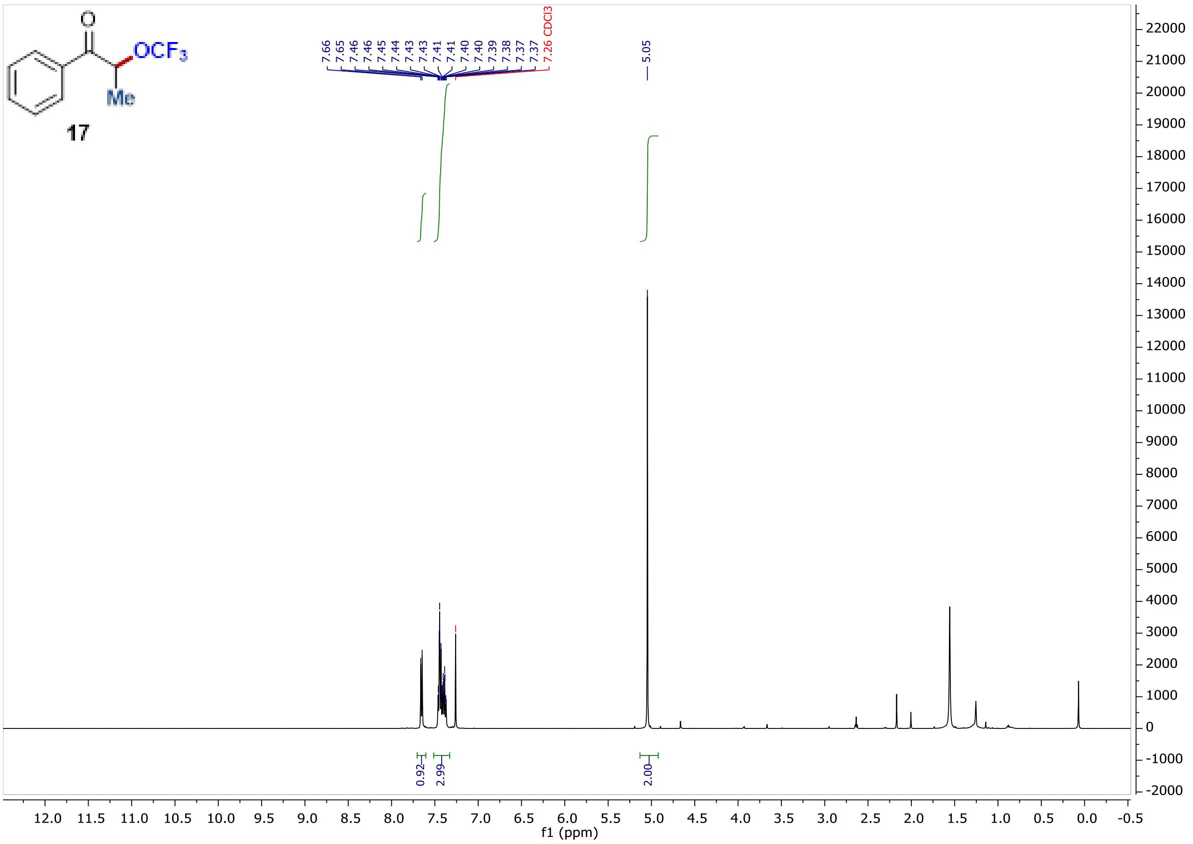

^{13}C NMR (126 MHz, $CDCl_3$)

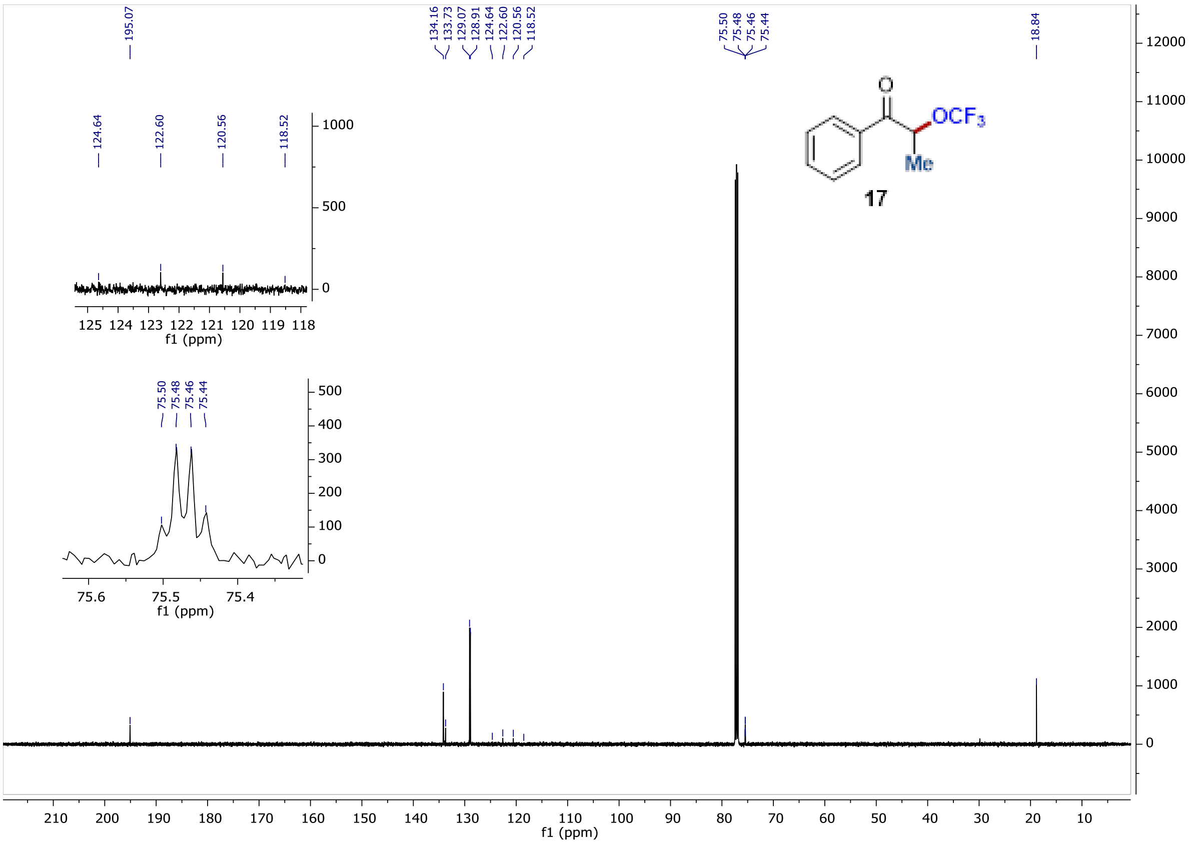

^{19}F NMR (188 MHz, $CDCl_3$)

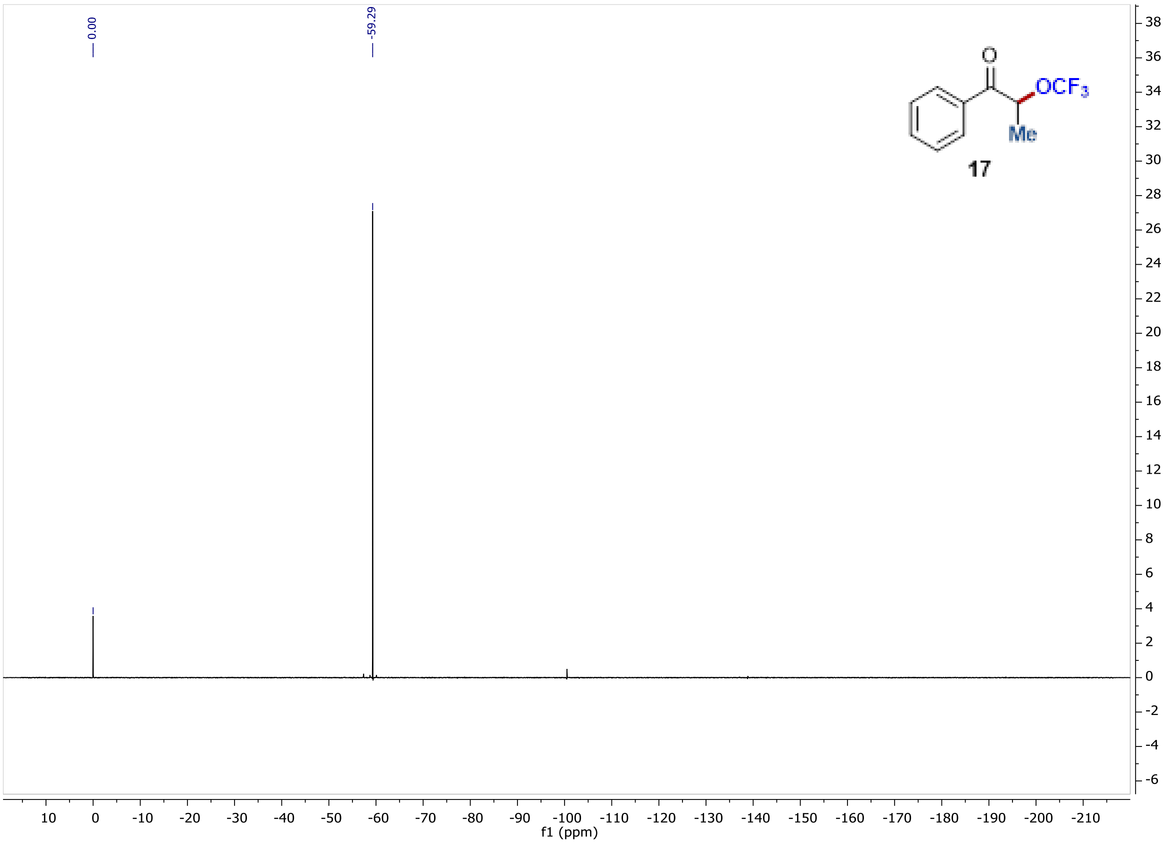

1,3-diphenyl-2-(trifluoromethoxy)propan-1-one 18

^{1}H NMR (500 MHz, $CDCl_3$)

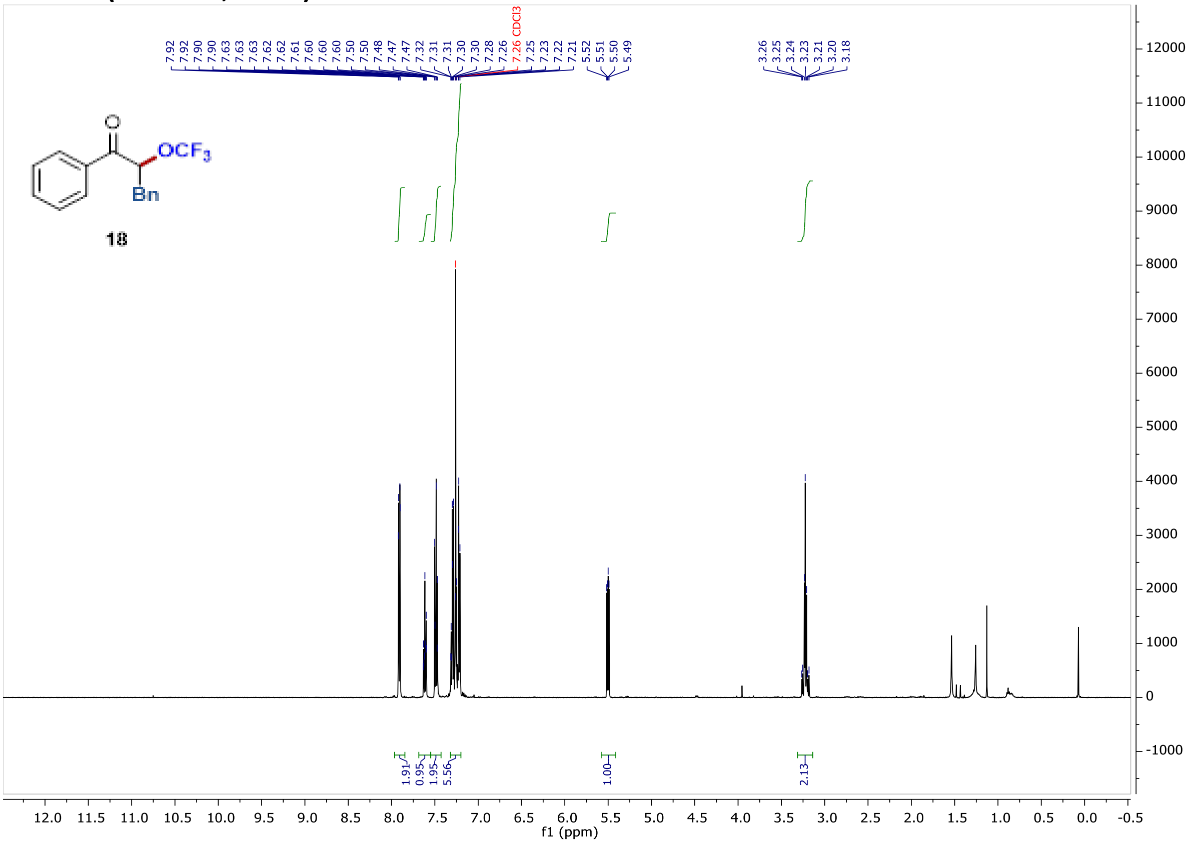

^{13}C NMR (126 MHz, $CDCl_3$)

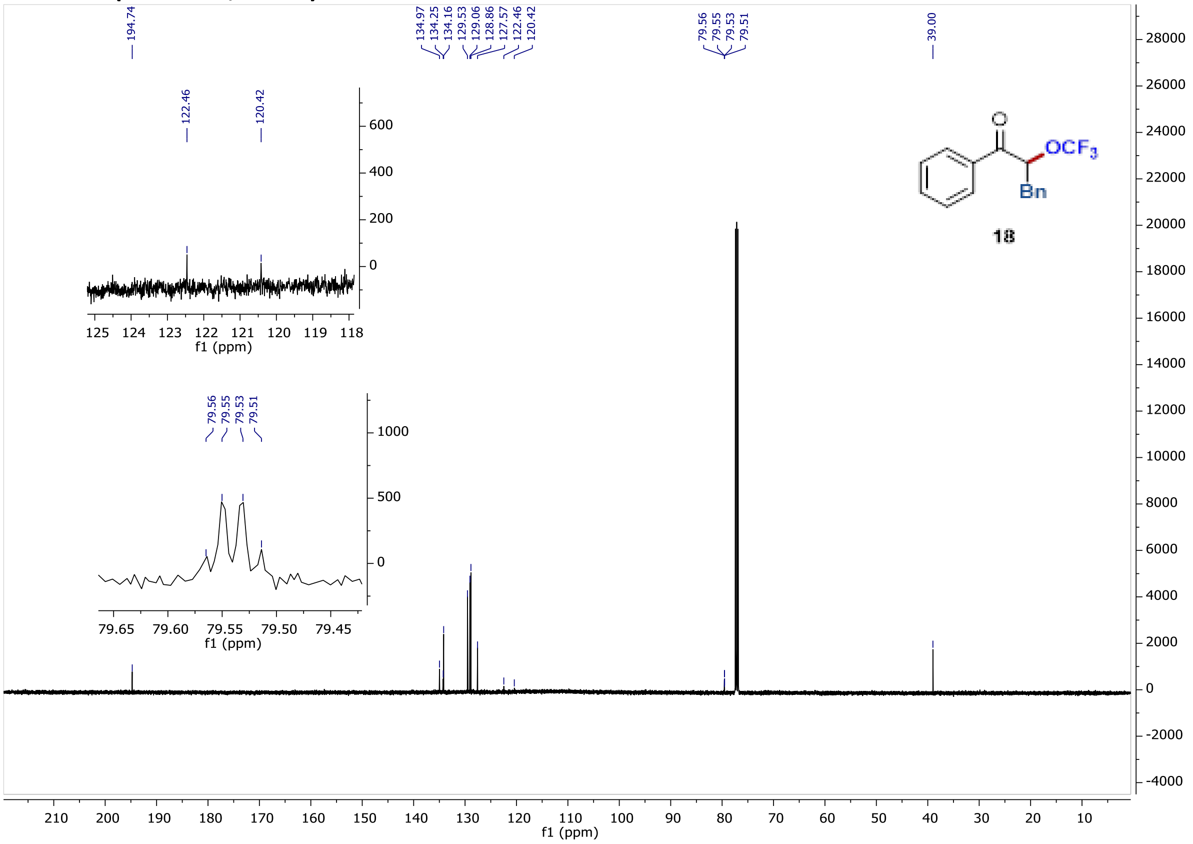

^{19}F NMR (188 MHz, $CDCl_3$)

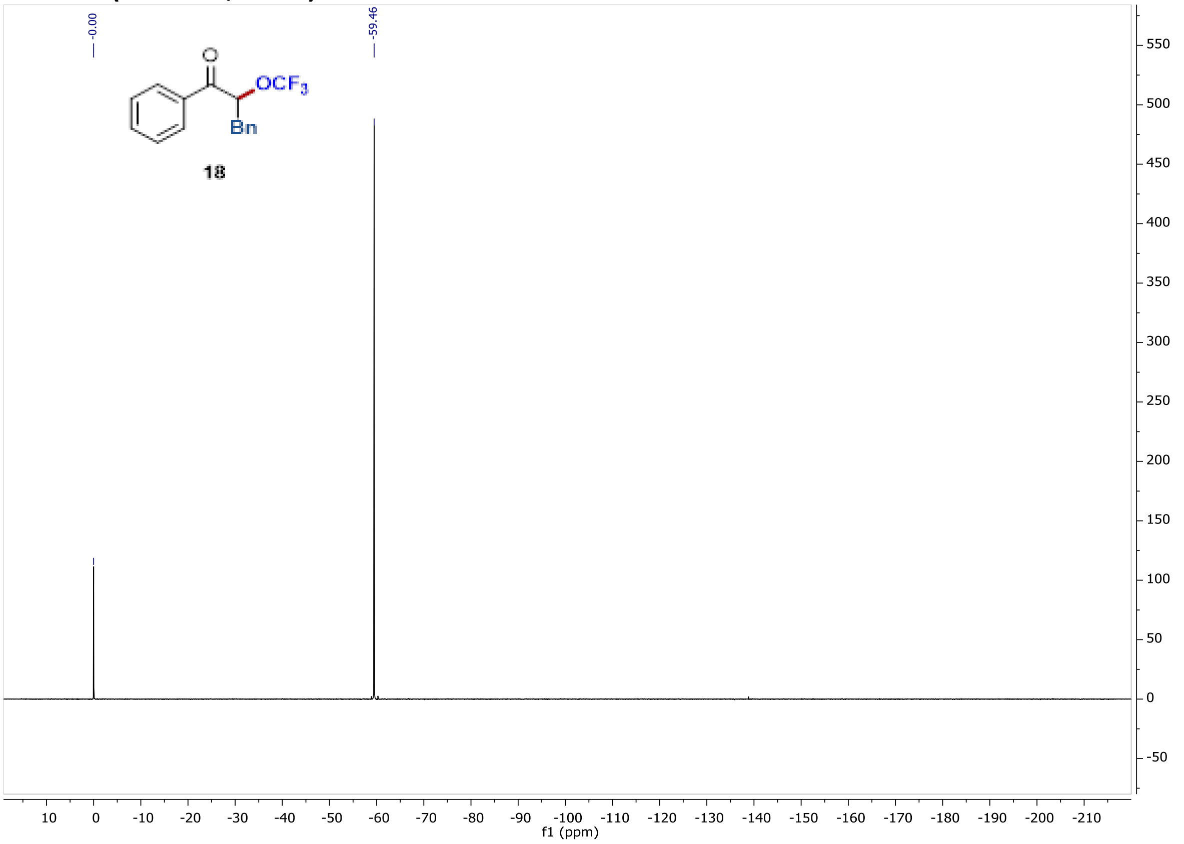

2-(trifluoromethoxy)-2,3-dihydro-1H-inden-1-one 19

¹H NMR (300 MHz, $CDCl_3$)

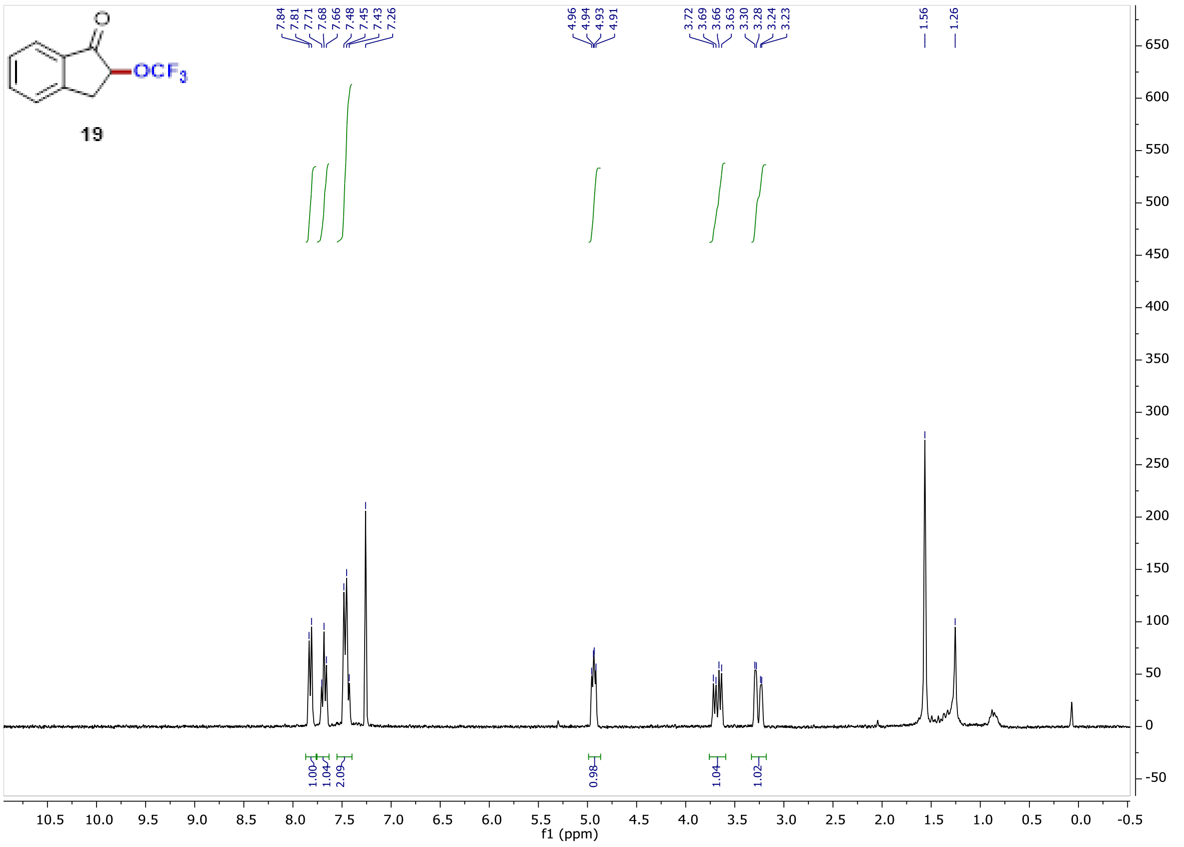

¹³C NMR (75 MHz, $CDCl_3$)

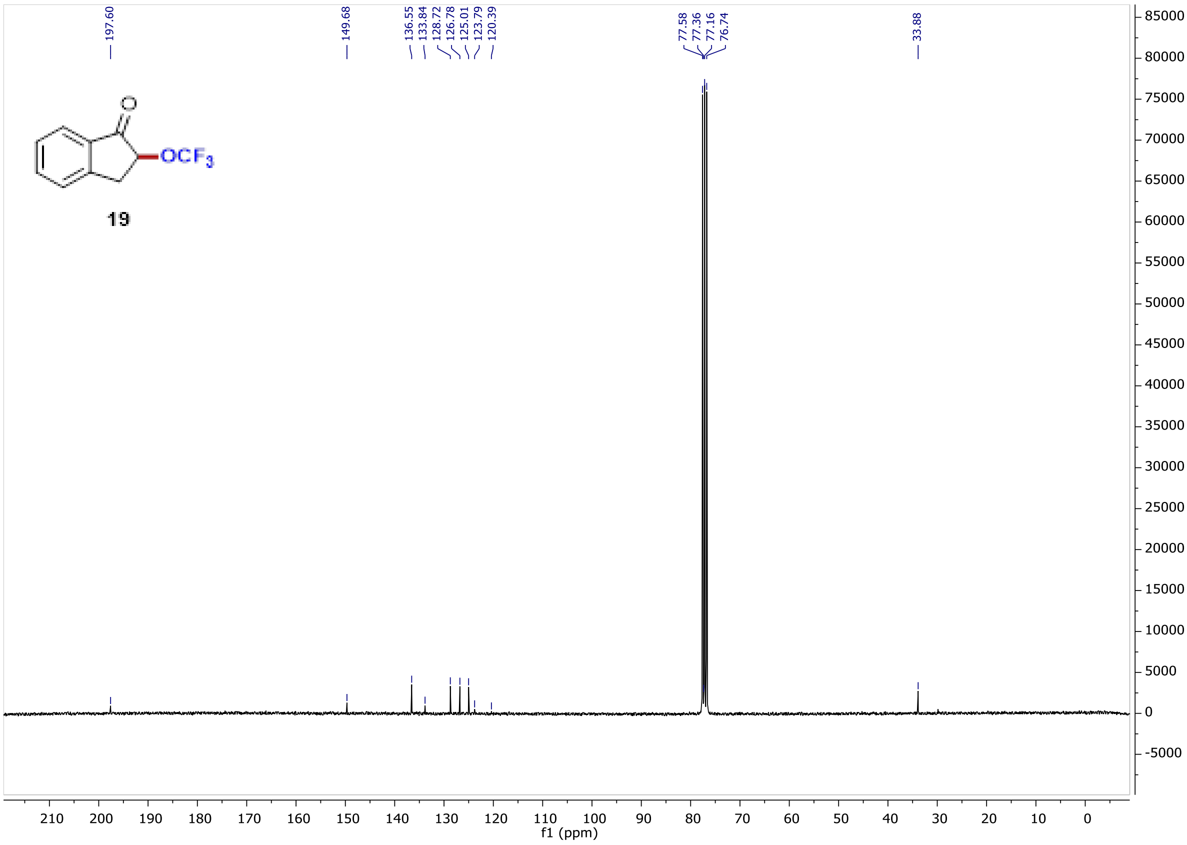

^{19}F NMR (188 MHz, $CDCl_3$)

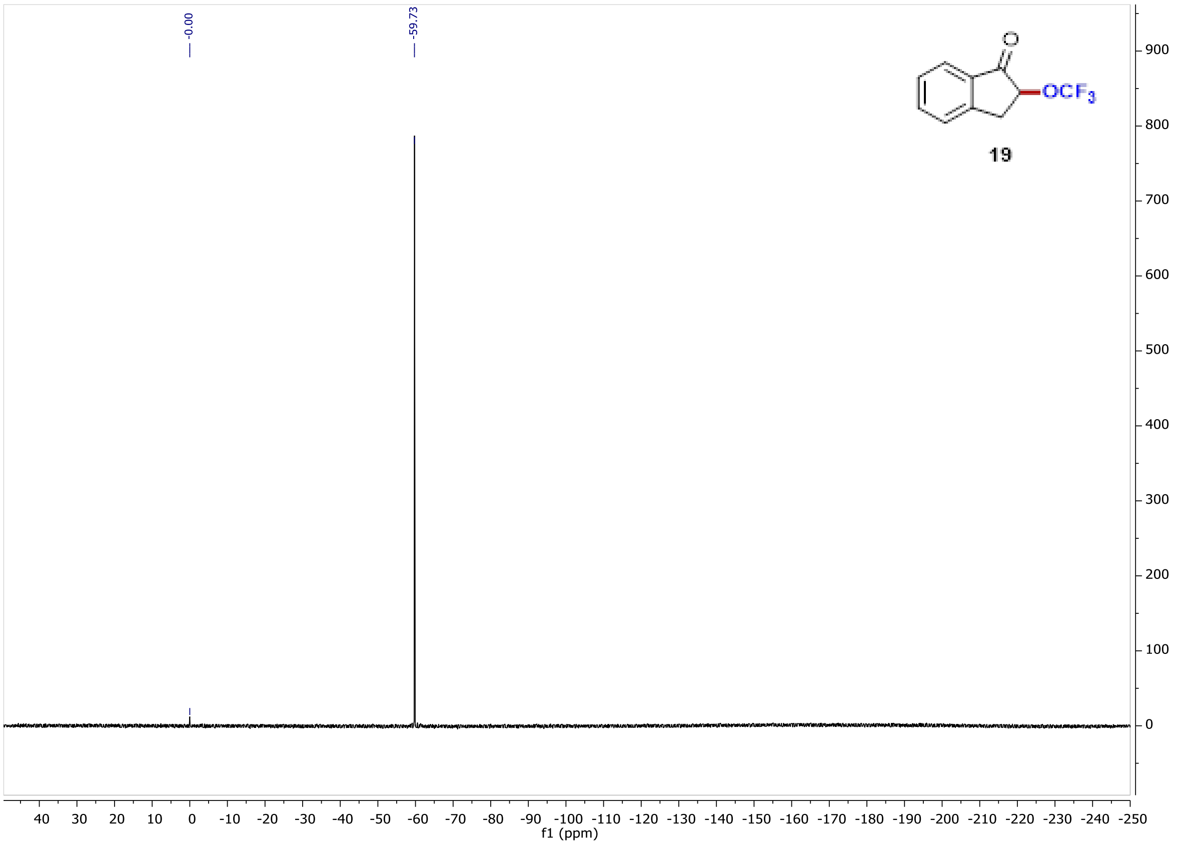

5-fluoro-2-(trifluoromethoxy)-2,3-dihydro-1H-inden-1-one 20

^{1}H NMR (300 MHz, $CDCl_3$)

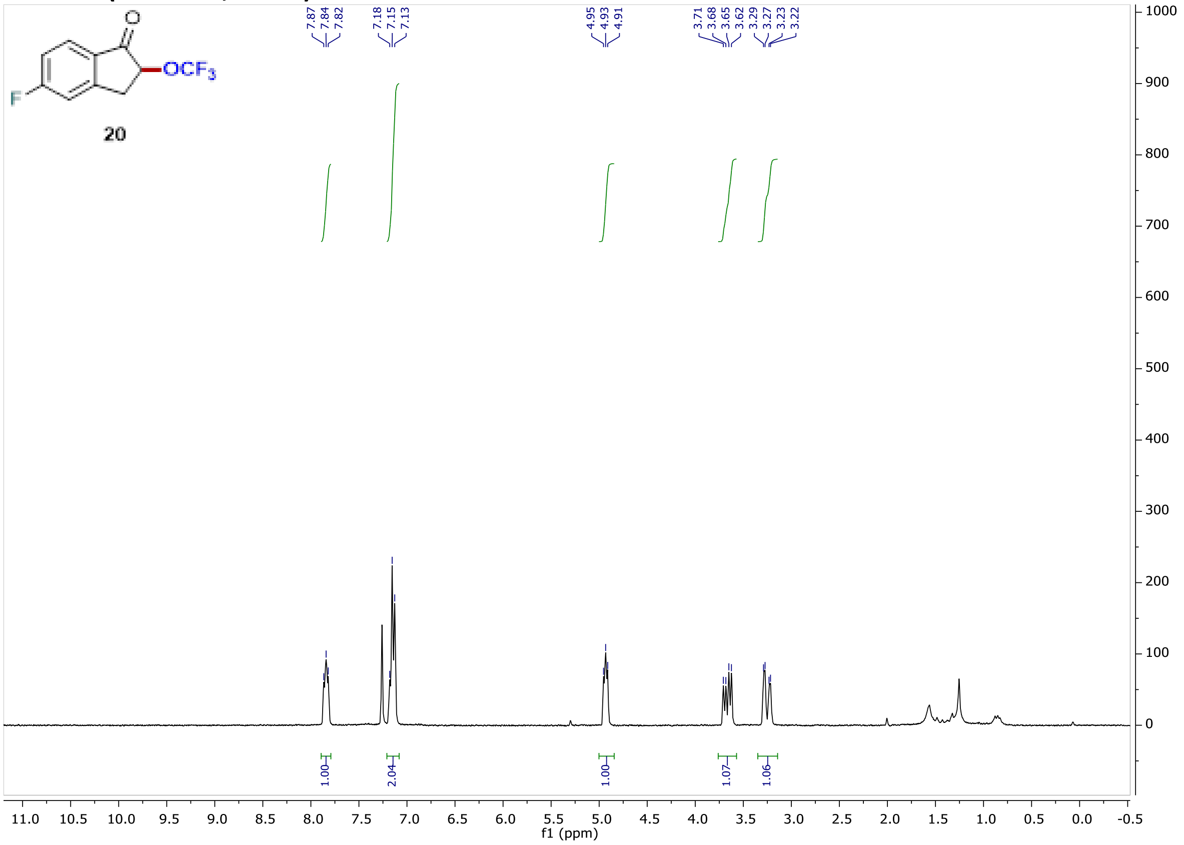

^{13}C NMR (75 MHz, $CDCl_3$)

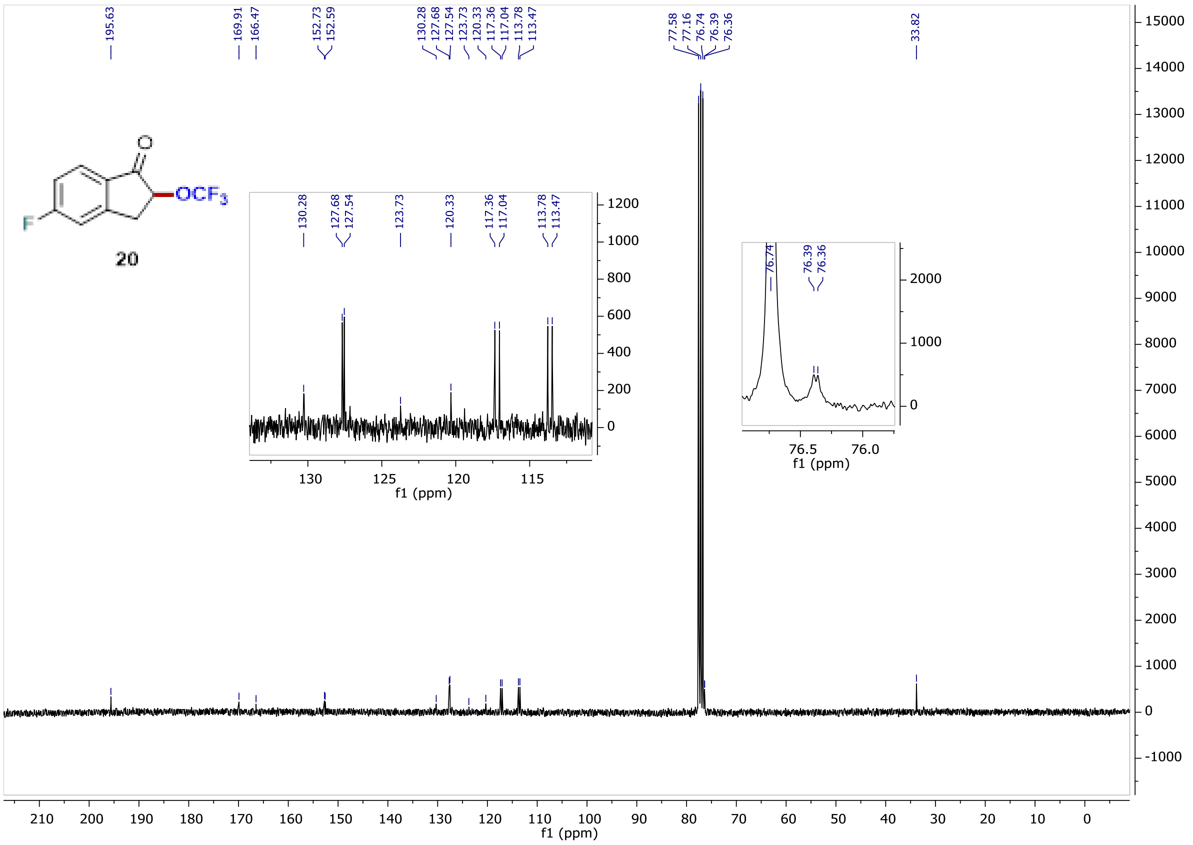

^{19}F NMR (188 MHz, $CDCl_3$)

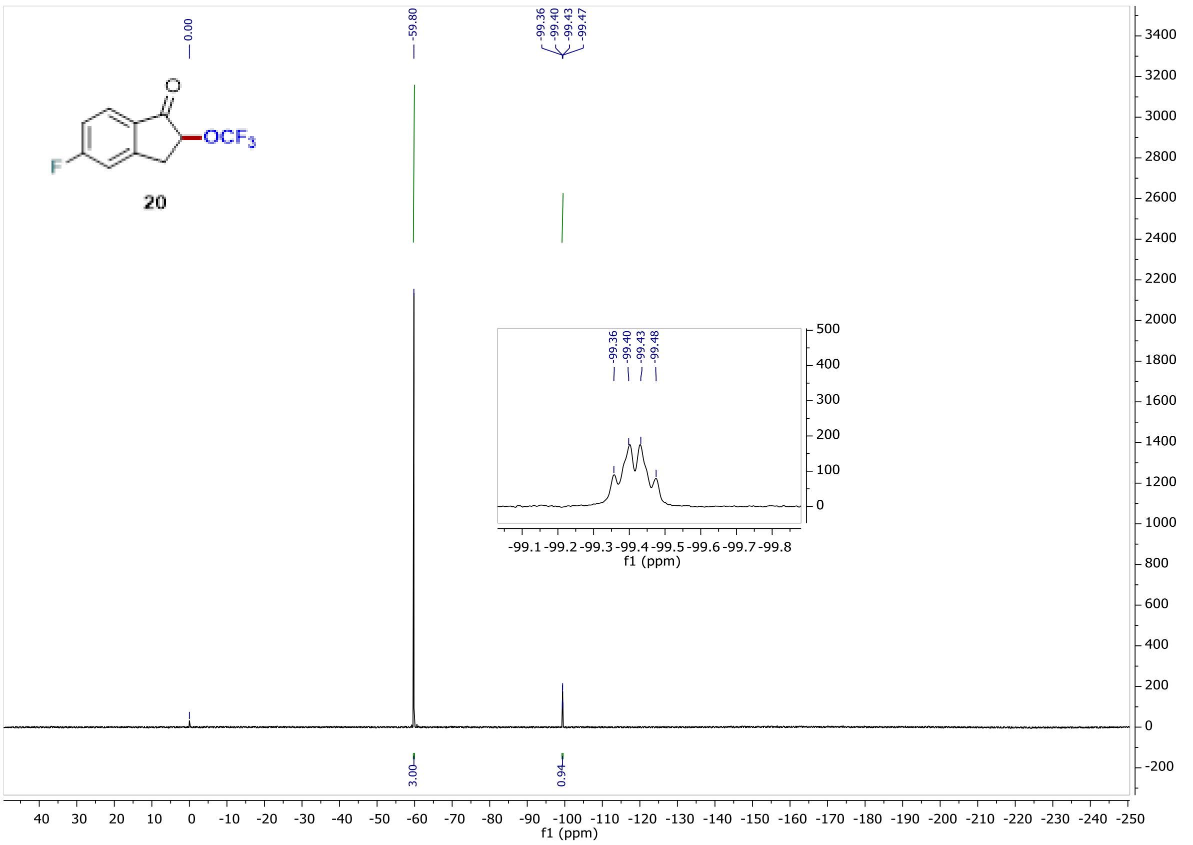

6-(trifluoromethoxy)-6,7,8,9-tetrahydro-5H-benzo[7]annulen-5-one 21

^{1}H NMR (300 MHz, $CDCl_3$)

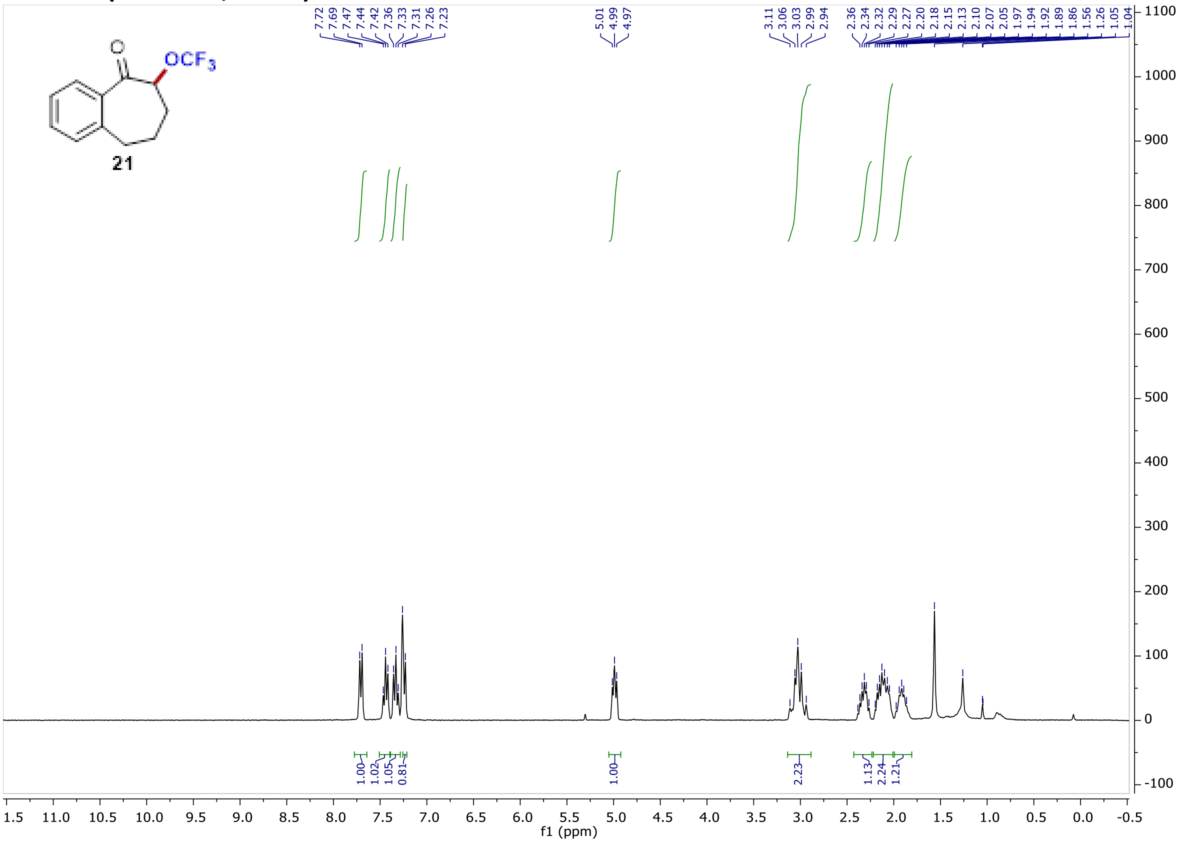

^{13}C NMR (75 MHz, $CDCl_3$)

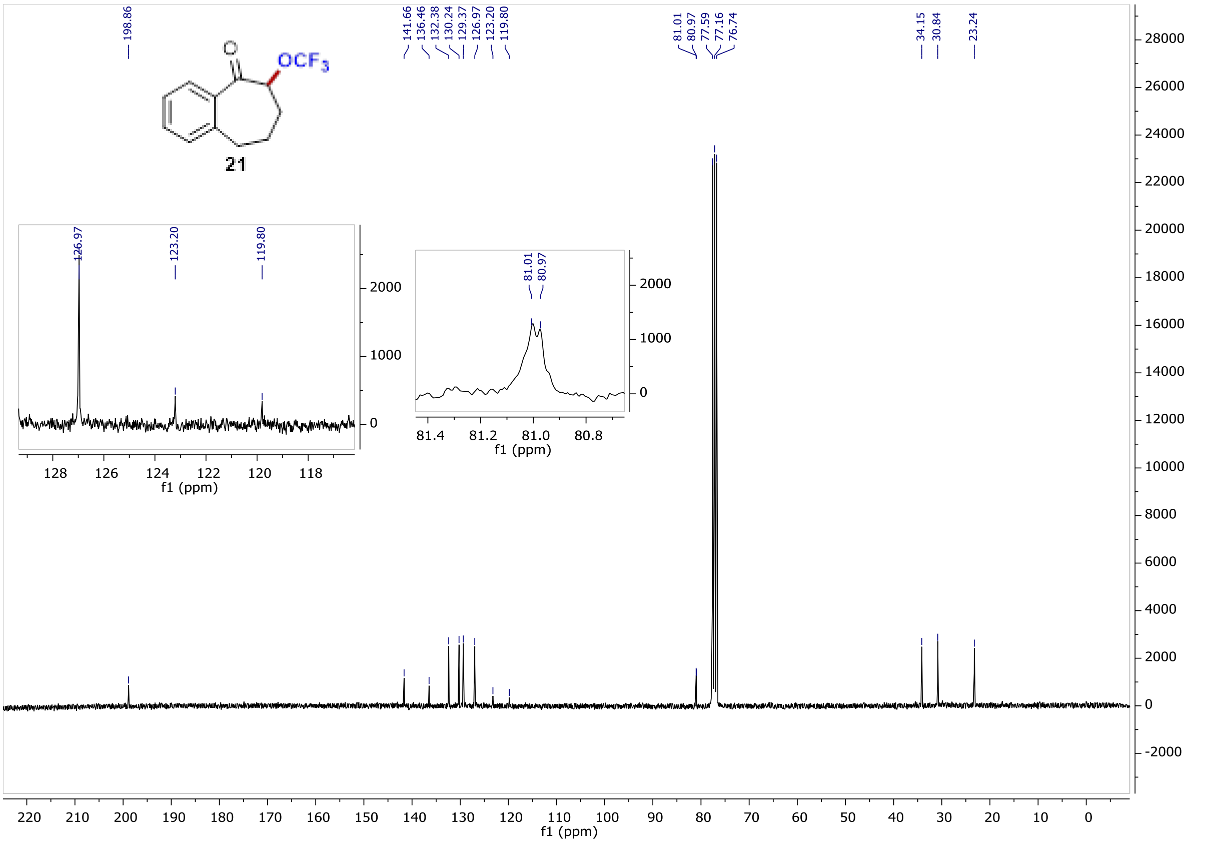

^{19}F NMR (188 MHz, $CDCl_3$)

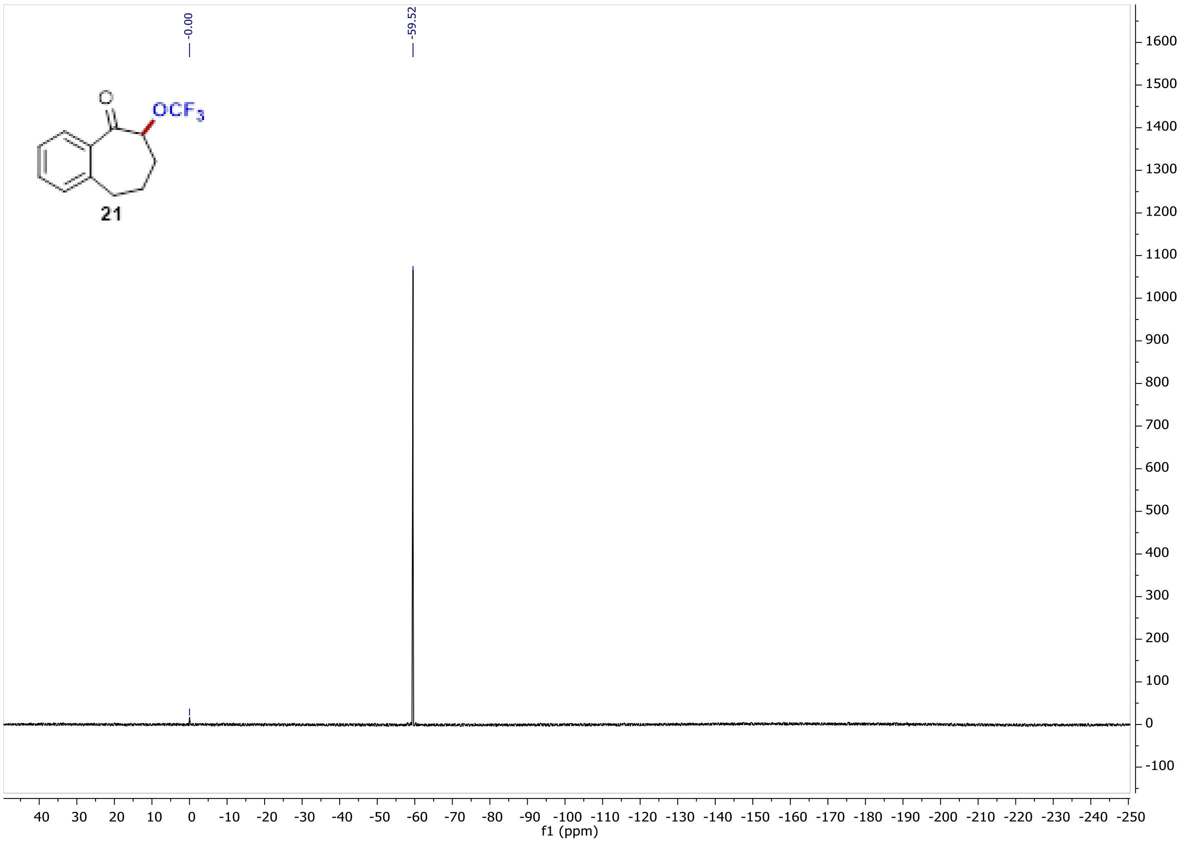

(E)-4-phenyl-1-(trifluoromethoxy)but-3-en-2-one 25

^{1}H NMR (300 MHz, $CDCl_3$)

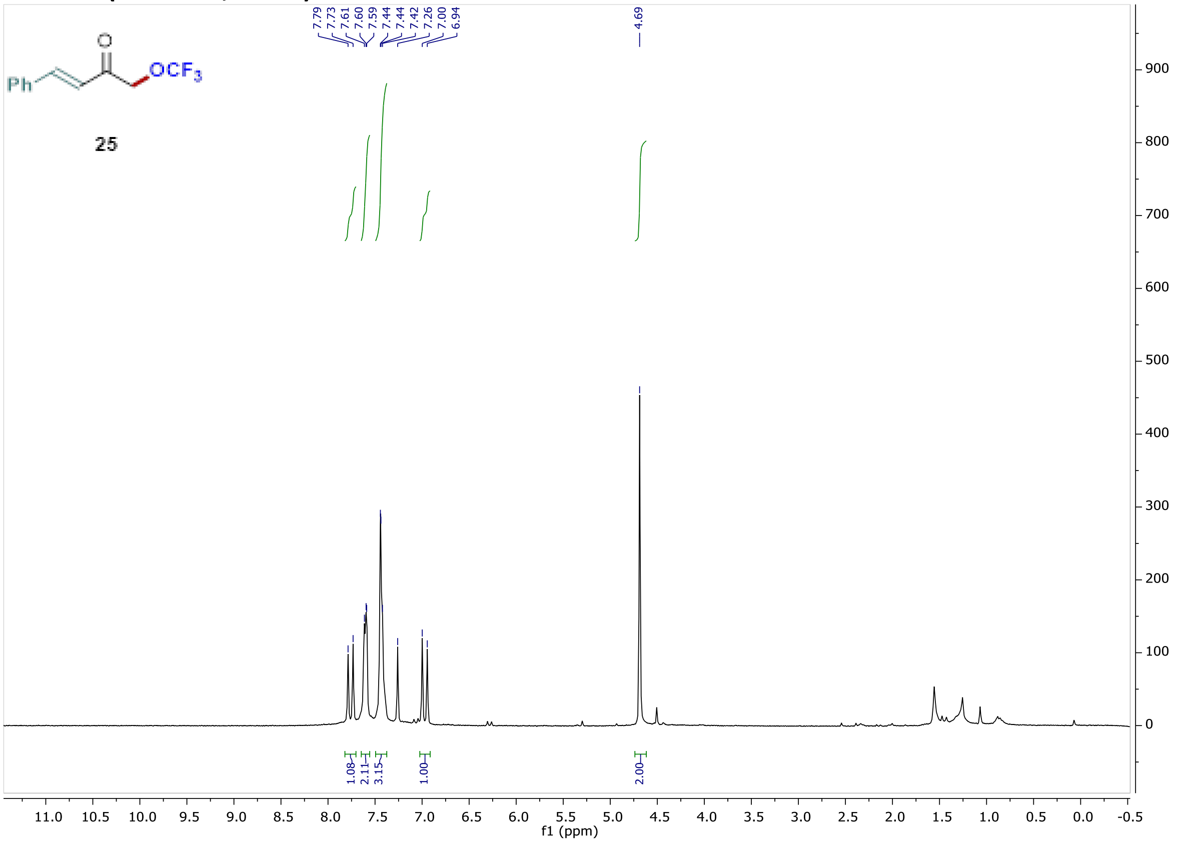

^{13}C NMR (75 MHz, $CDCl_3$)

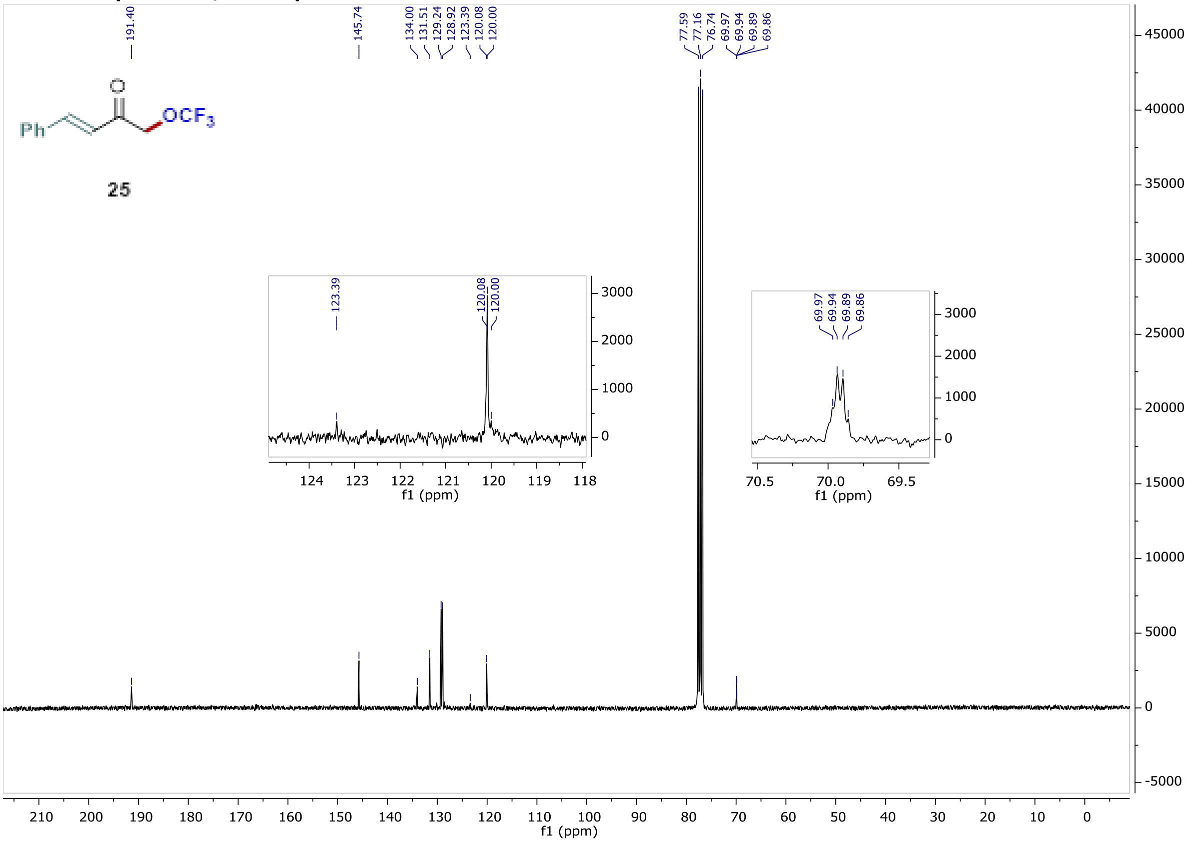

^{19}F NMR (188 MHz, $CDCl_3$)

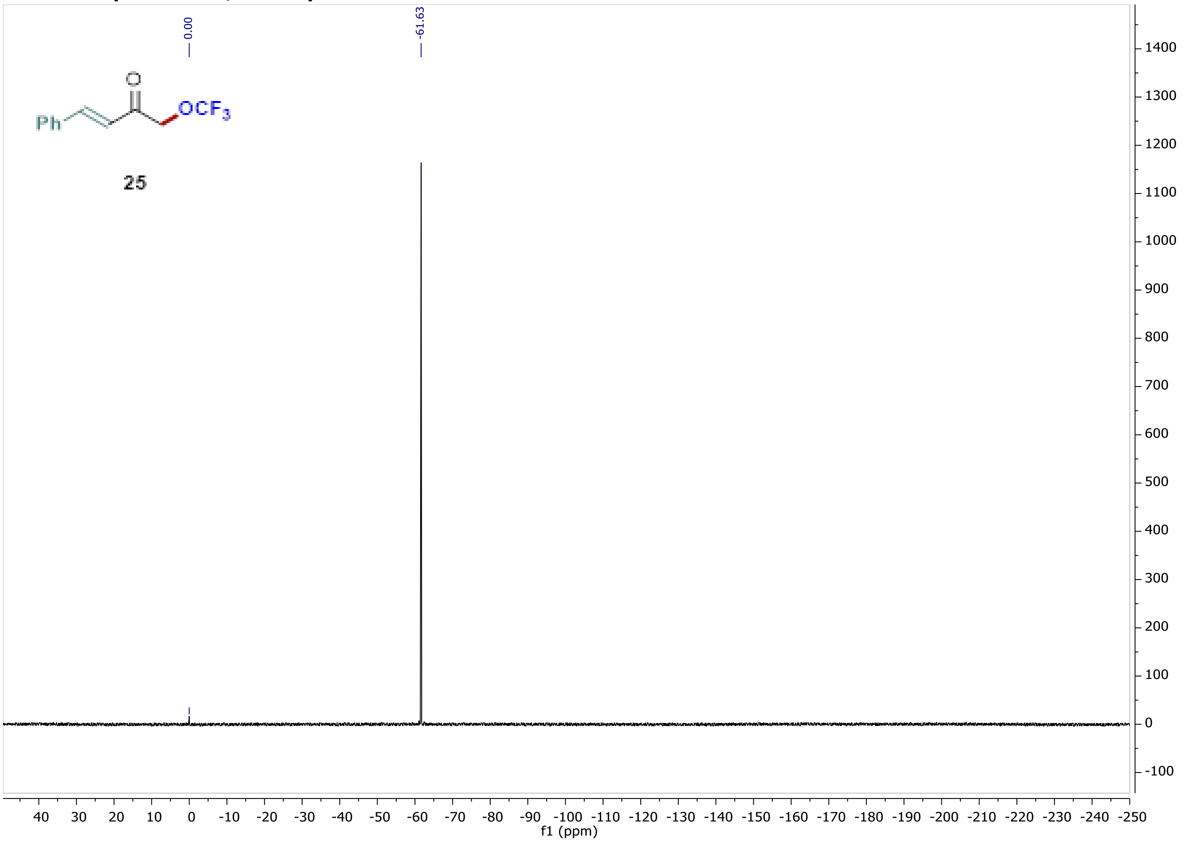

(E)-1-(trifluoromethoxy)non-3-en-2-one 26

^{19}F NMR (188 MHz, $CDCl_3$)

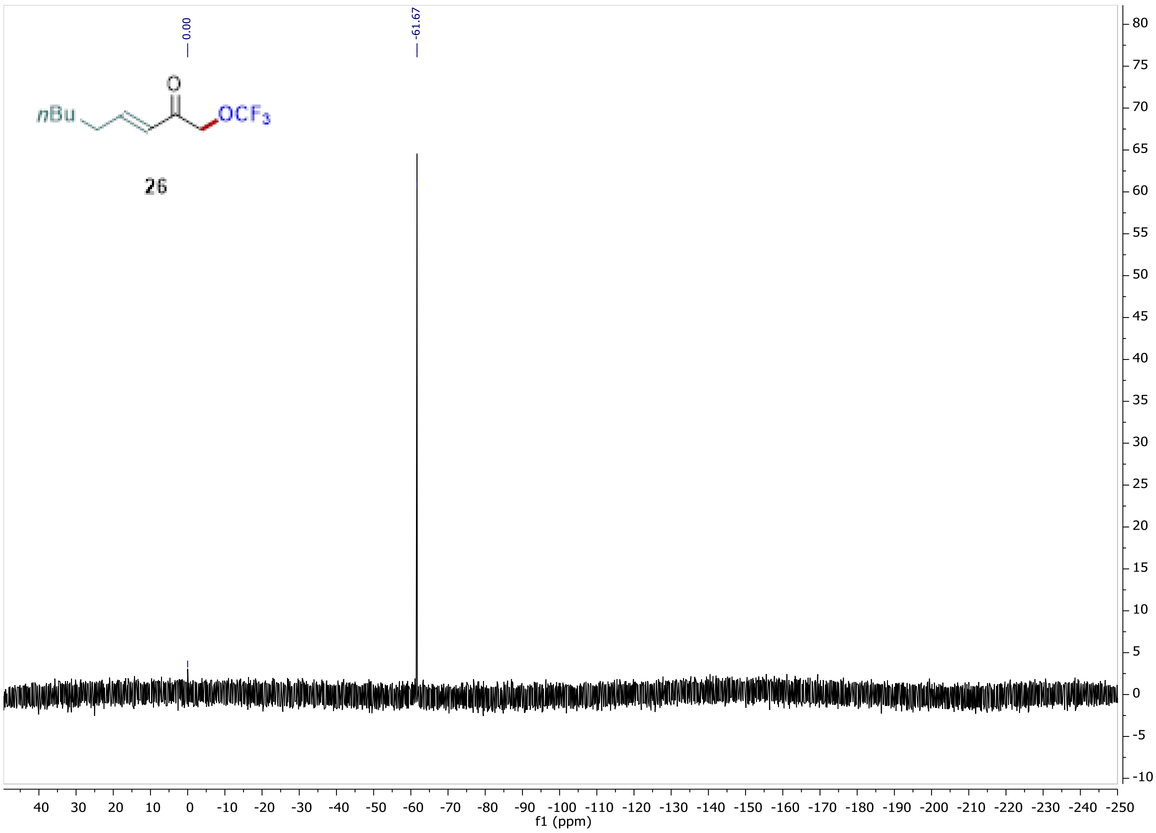

1-(3,5,5,6,8,8-hexamethyl-5,6,7,8-tetrahydronaphthalen-2-yl)-2-(trifluoromethoxy)ethan-1-one 27

^{1}H NMR (500 MHz, $CDCl_3$)

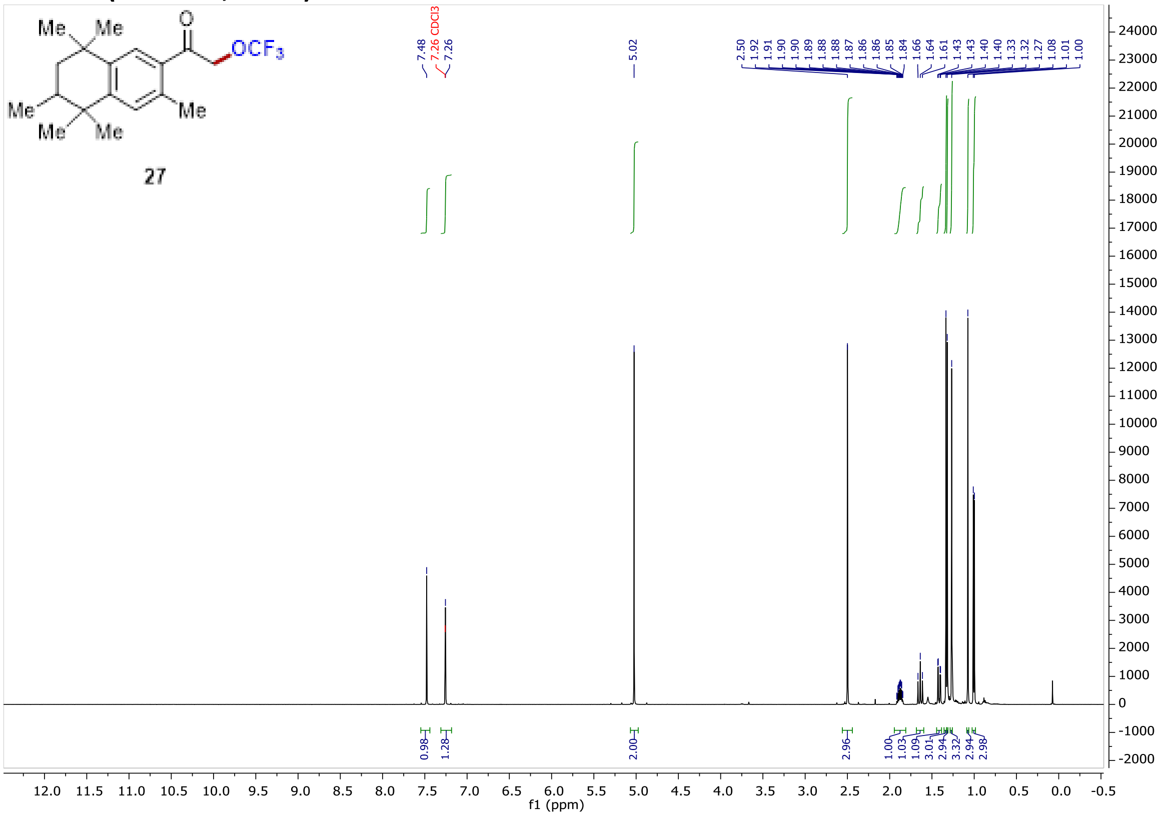

^{13}C NMR (126 MHz, $CDCl_3$)

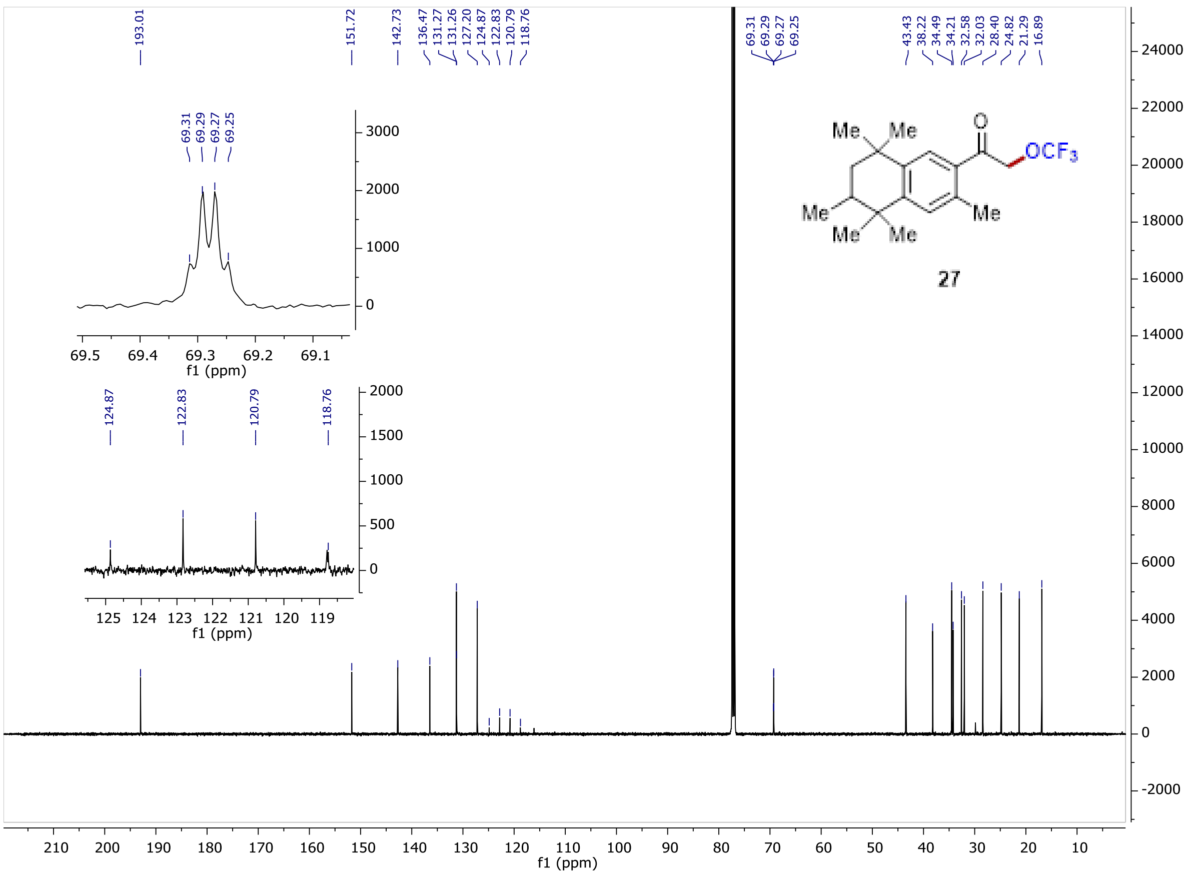

^{19}F NMR (188 MHz, $CDCl_3$)

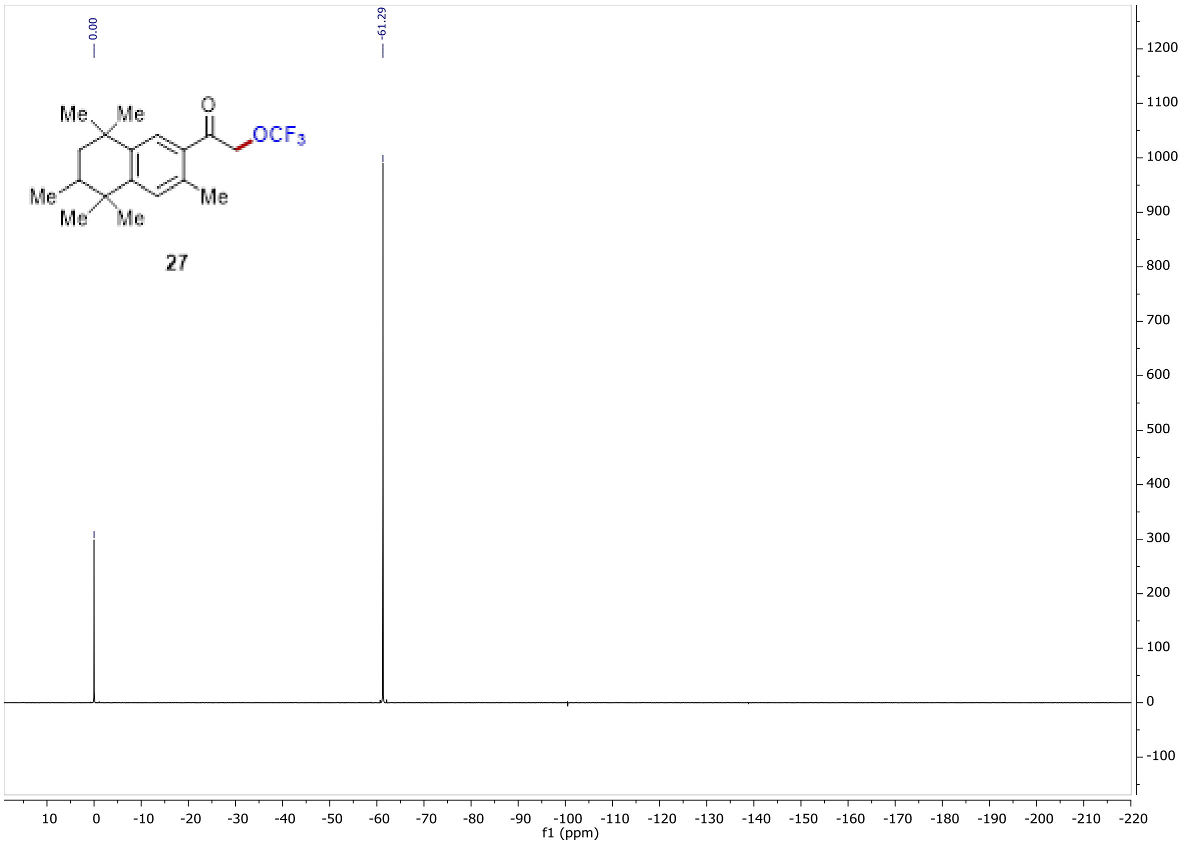

1-(4-(tert-butyl)-2,6-dimethyl-3,5-dinitrophenyl)-2-(trifluoromethoxy)ethanone 28

^{1}H NMR (300 MHz, $CDCl_3$)

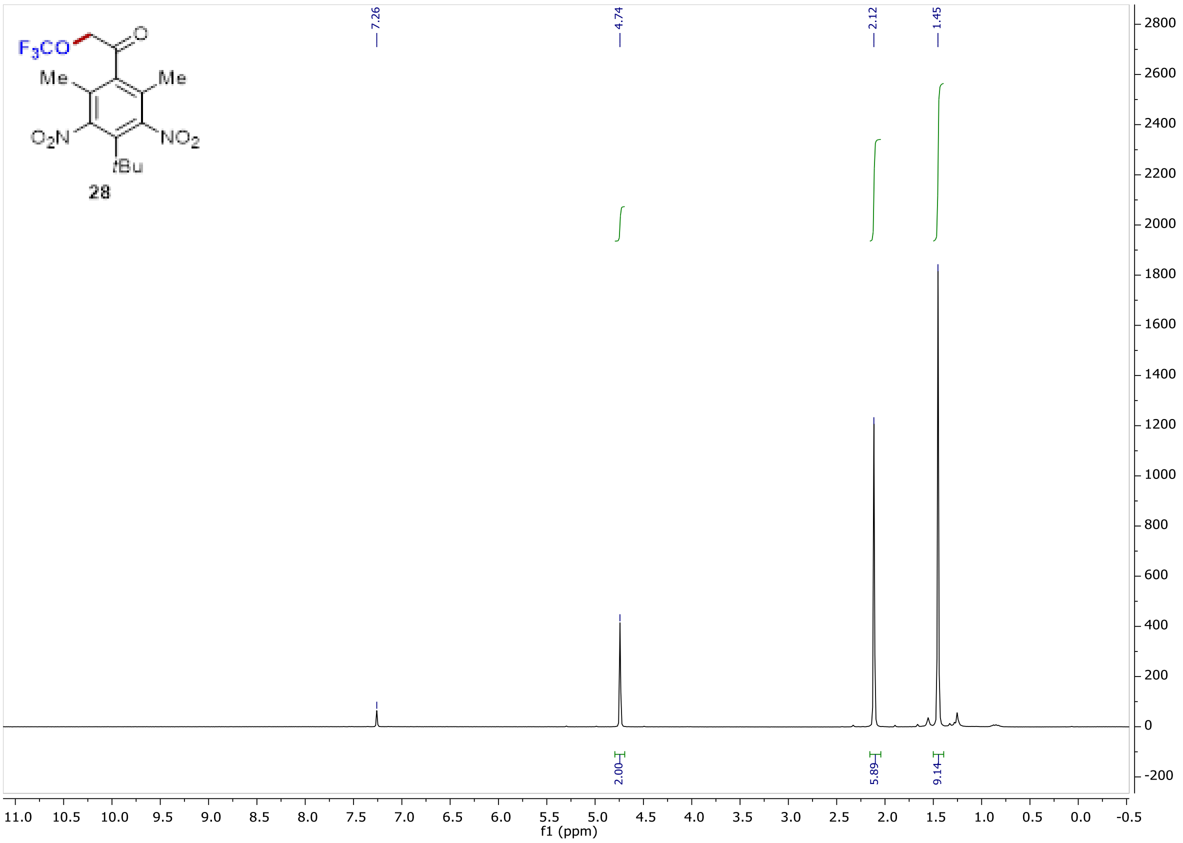

^{13}C NMR (75 MHz, $CDCl_3$)

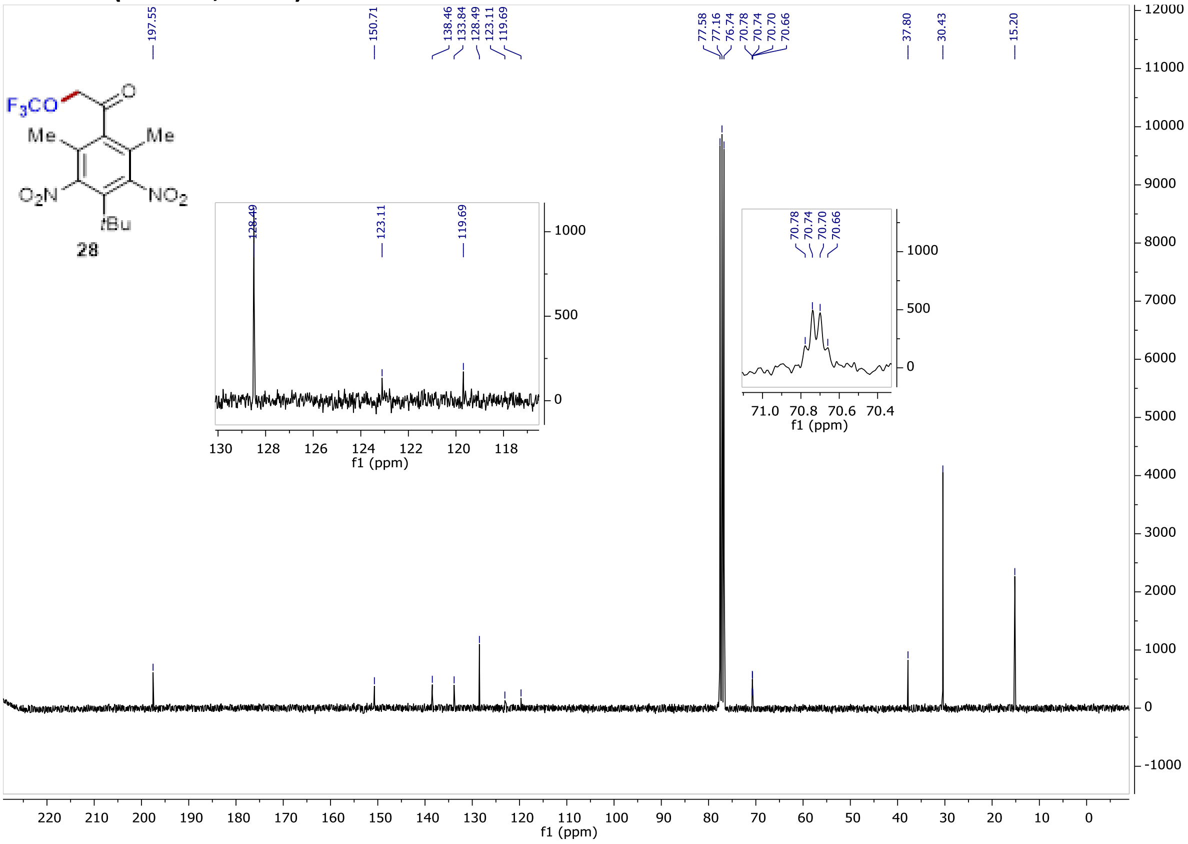

^{19}F NMR (188 MHz, $CDCl_3$)

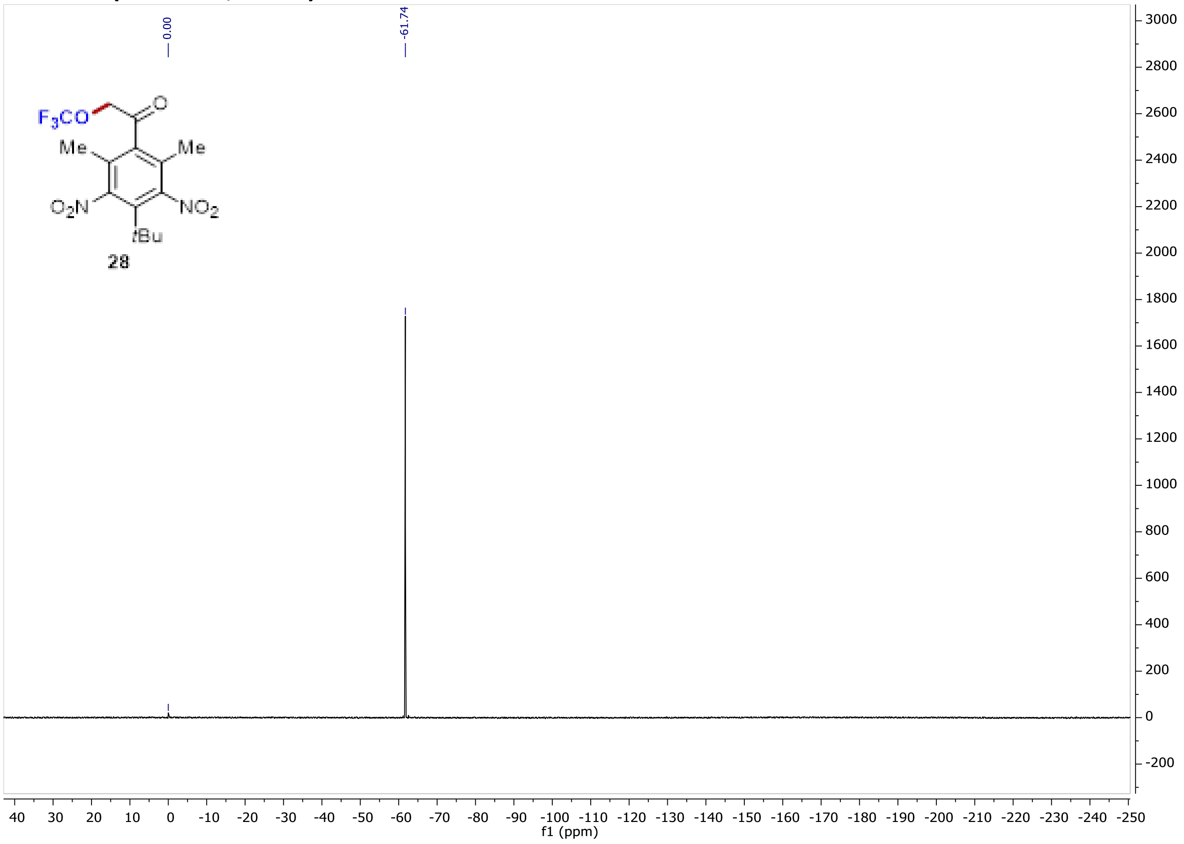

1-(6-(tert-butyl)-1,1-dimethyl-2,3-dihydro-1H-inden-4-yl)-2-(trifluoromethoxy)ethanone 29

^{1}H NMR (300 MHz, $CDCl_3$)

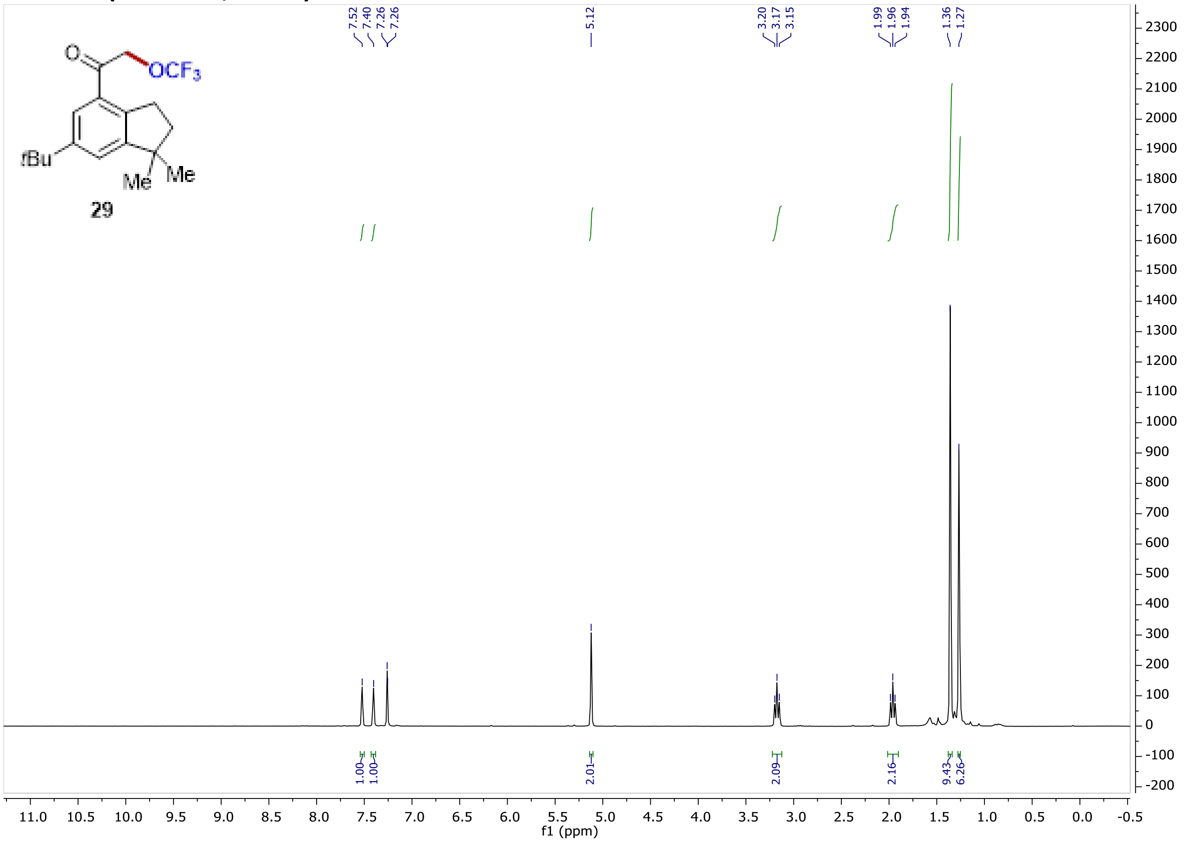

^{13}C NMR (75 MHz, $CDCl_3$)

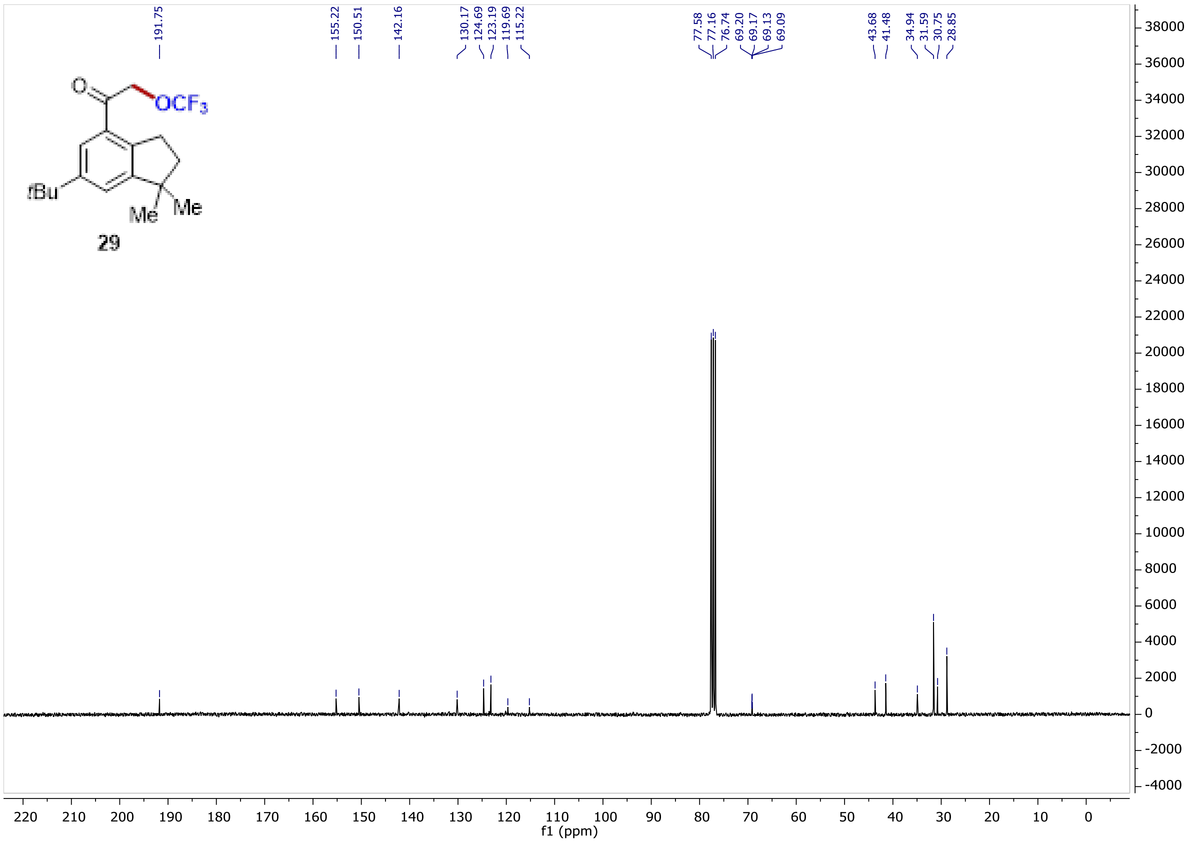

^{19}F NMR (188 MHz, $CDCl_3$)

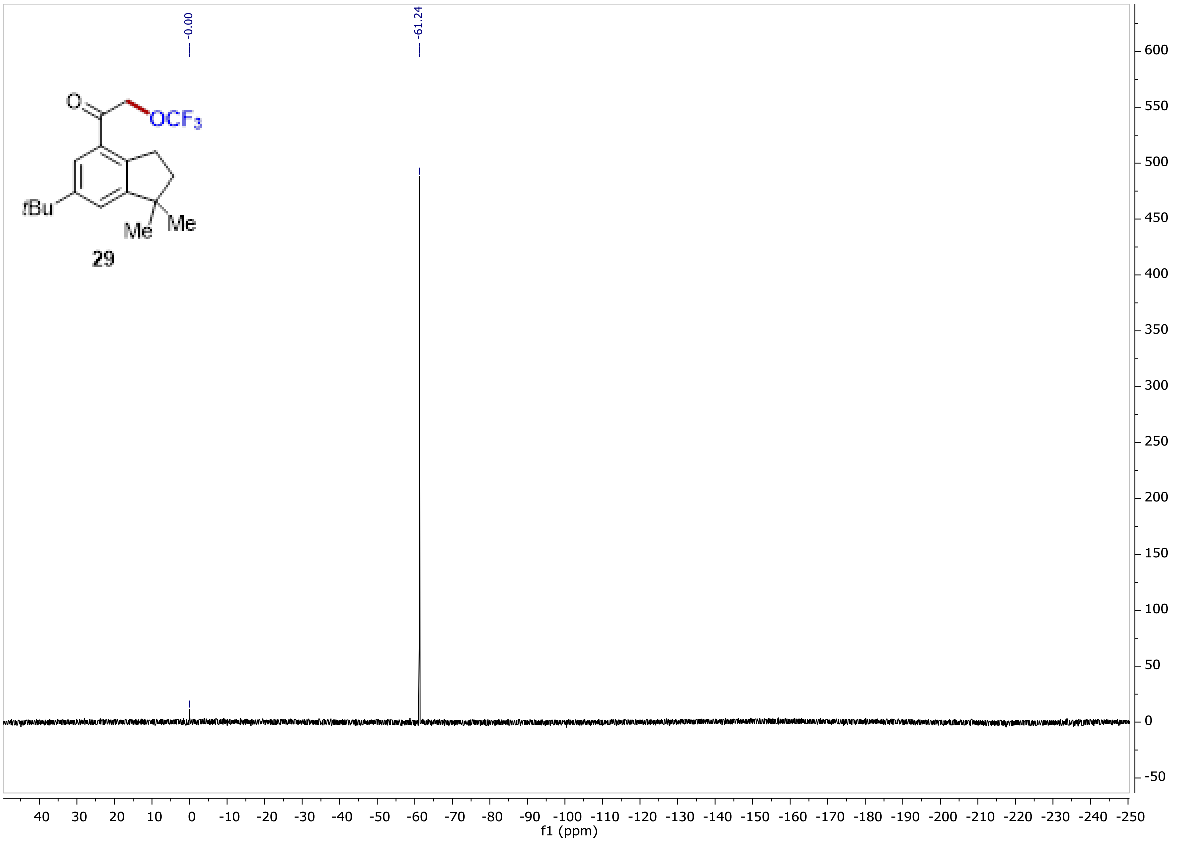

(E)-1-(trifluoromethoxy)-4-(2,6,6-trimethylcyclohex-2-en-1-yl)but-3-en-2-one 30

^{1}H NMR (200 MHz, $CDCl_3$)

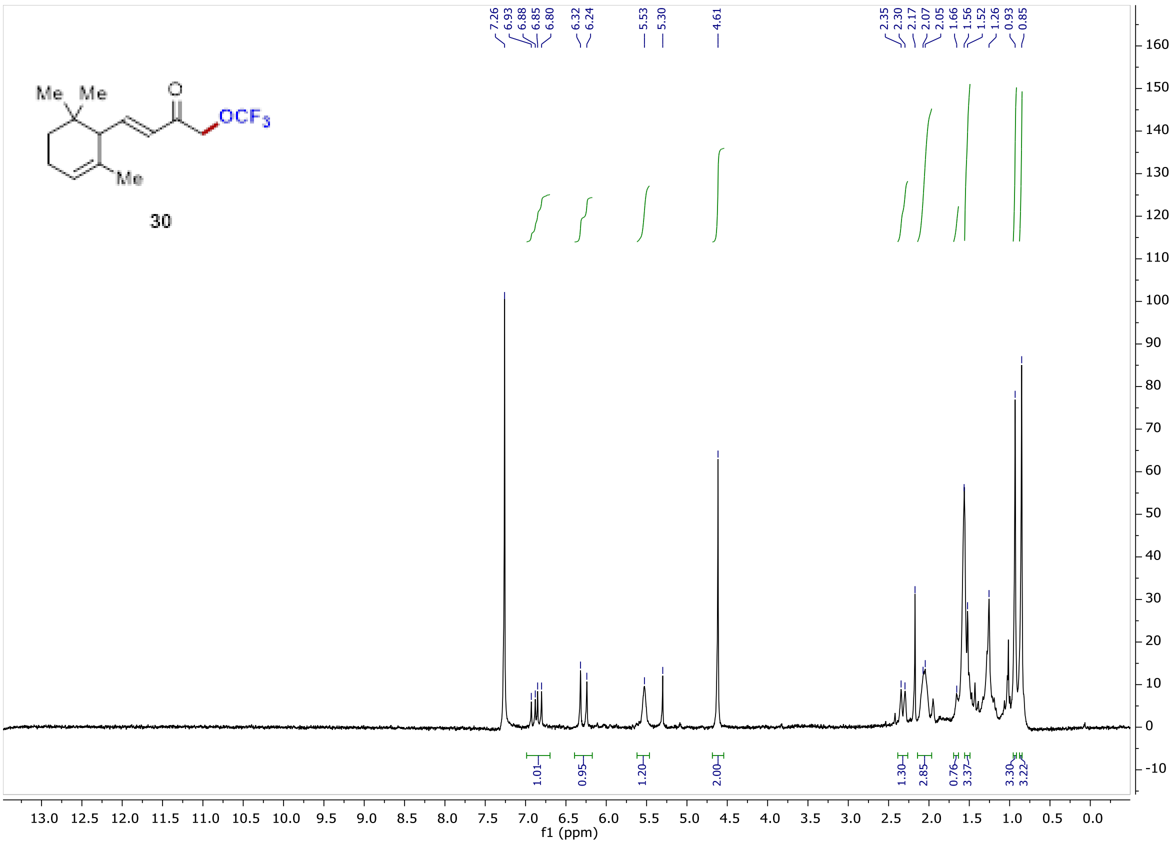

^{13}C NMR (151 MHz, $CDCl_3$)

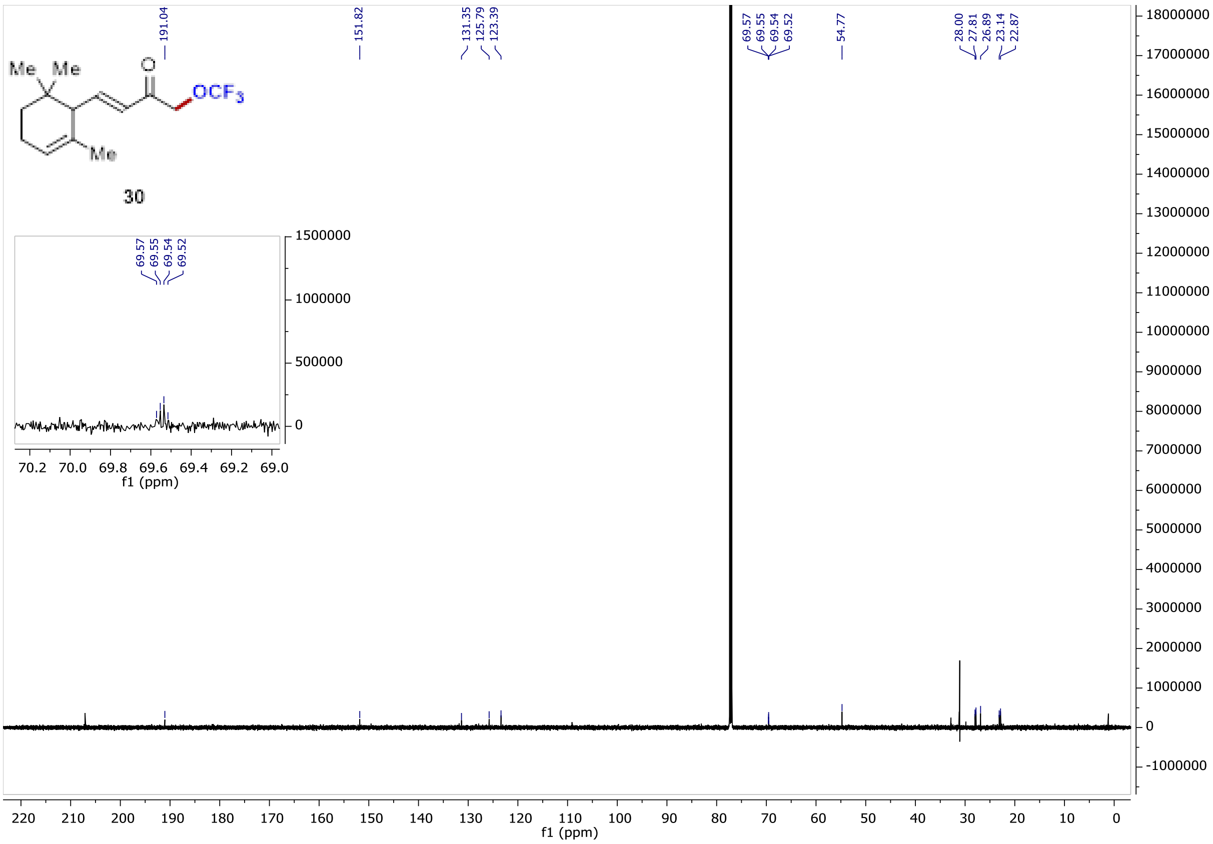

^{19}F NMR (188 MHz, $CDCl_3$)

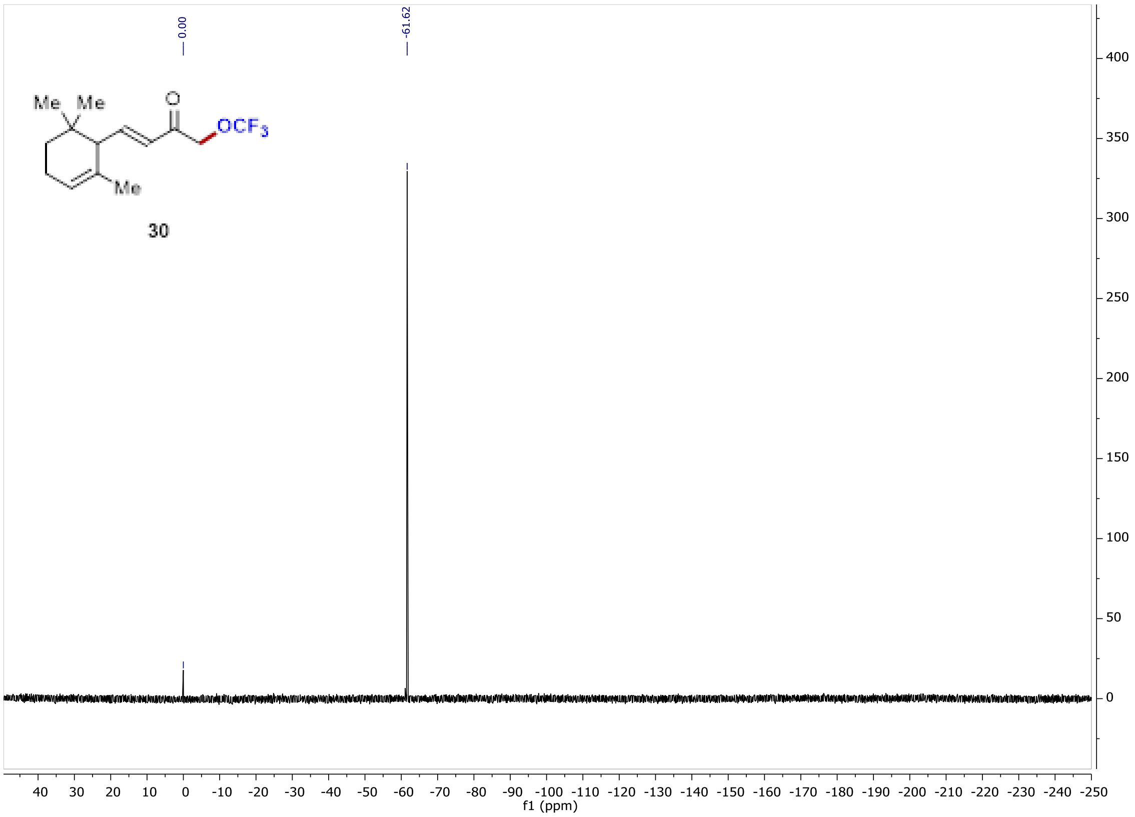

17-methoxy-10,13-dimethyl-6-(trifluoromethoxy)-6,7,8,9,10,11,12,13,14,15,16,17-dodecahydro-1H-cyclopenta[a]phenanthren-3(2H)-one 32

^{1}H NMR (600 MHz, $CDCl_3$)

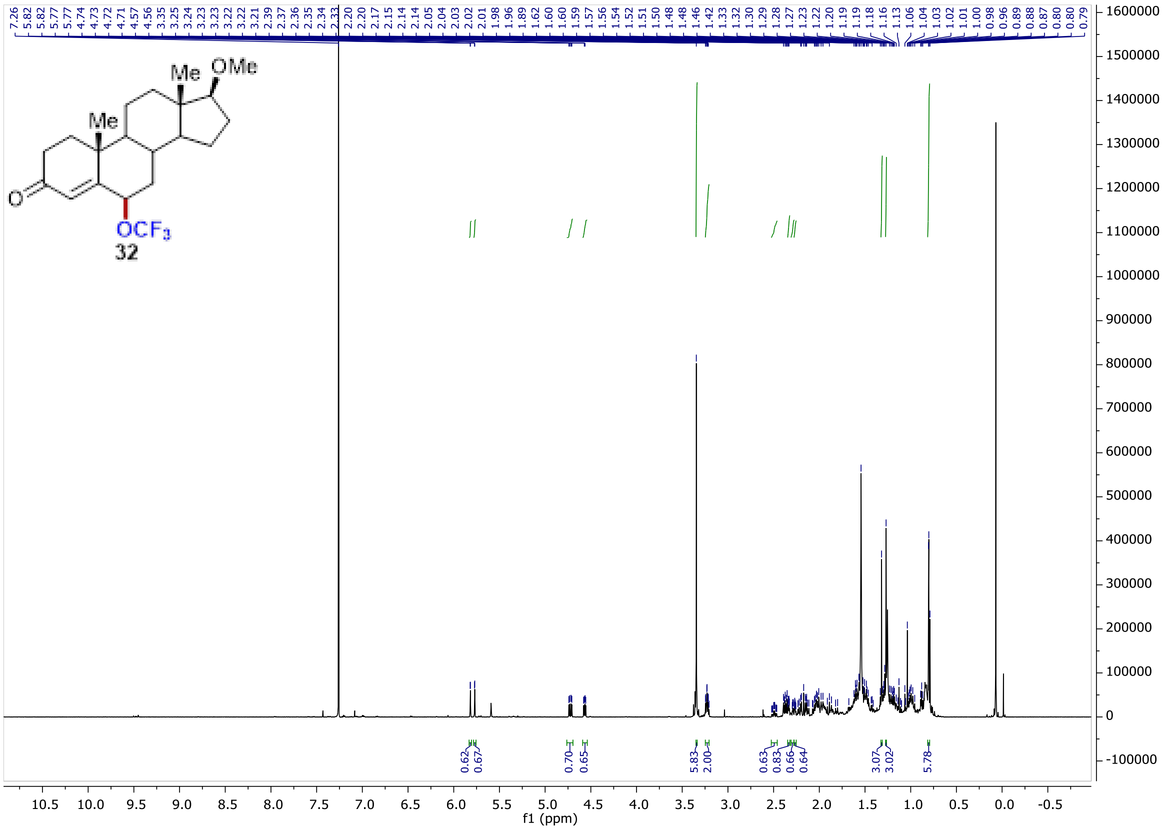

^{13}C NMR (151 MHz, $CDCl_3$)

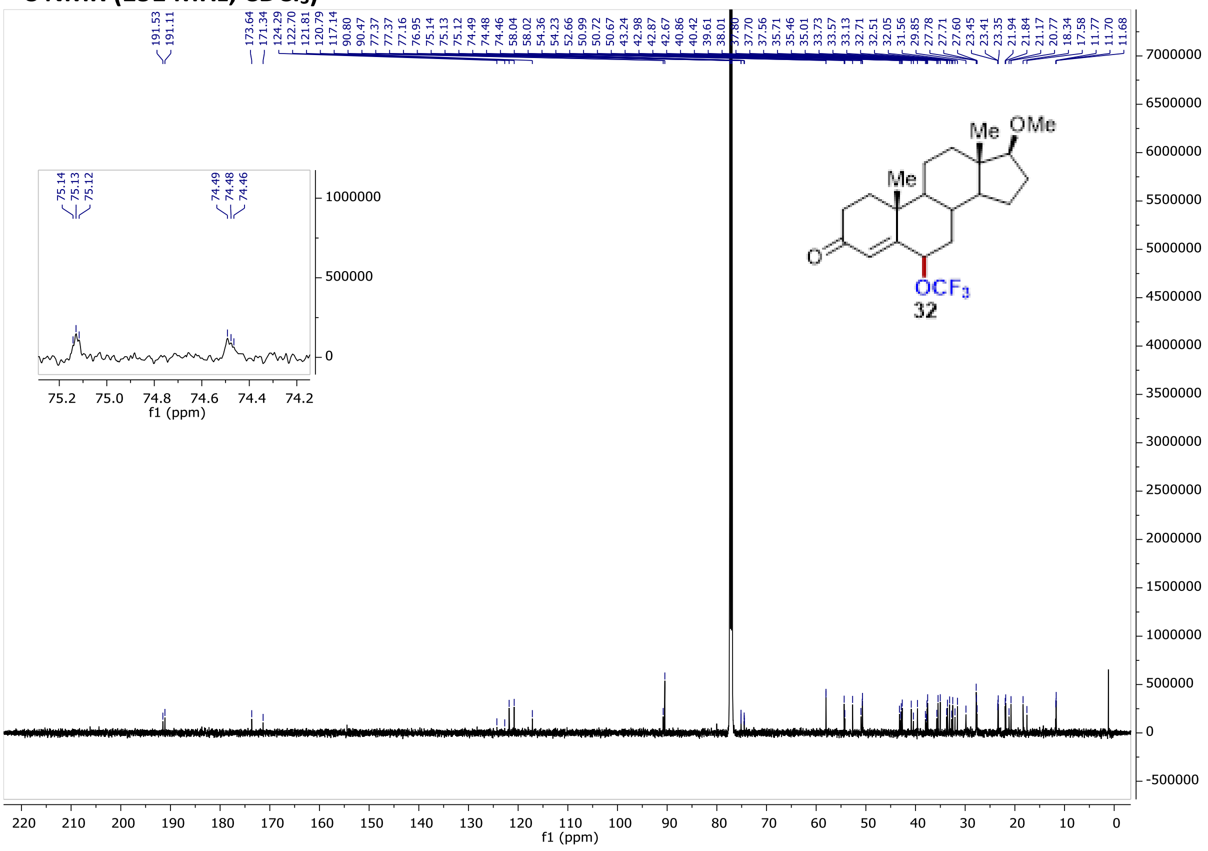

^{19}F NMR (188 MHz, $CDCl_3$)

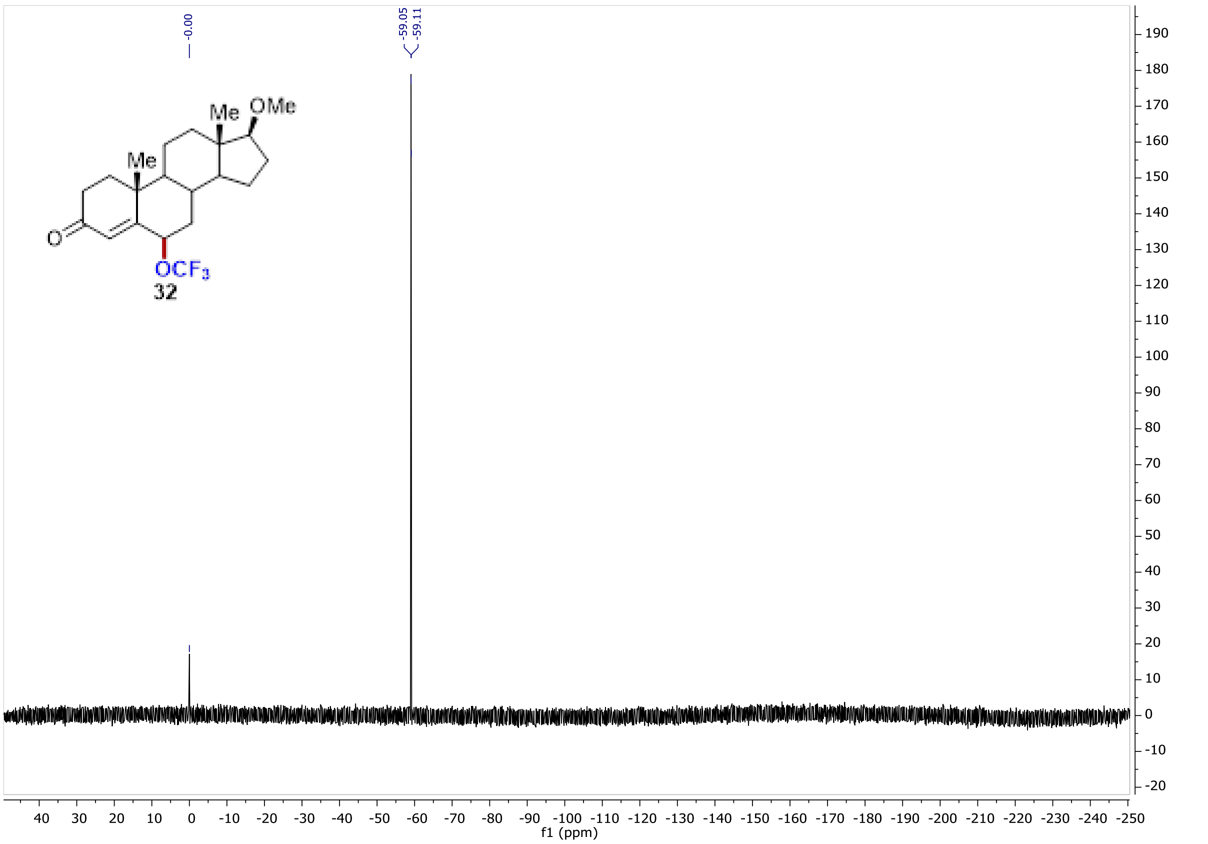

COSY (600 MHz, $CDCl_3$)

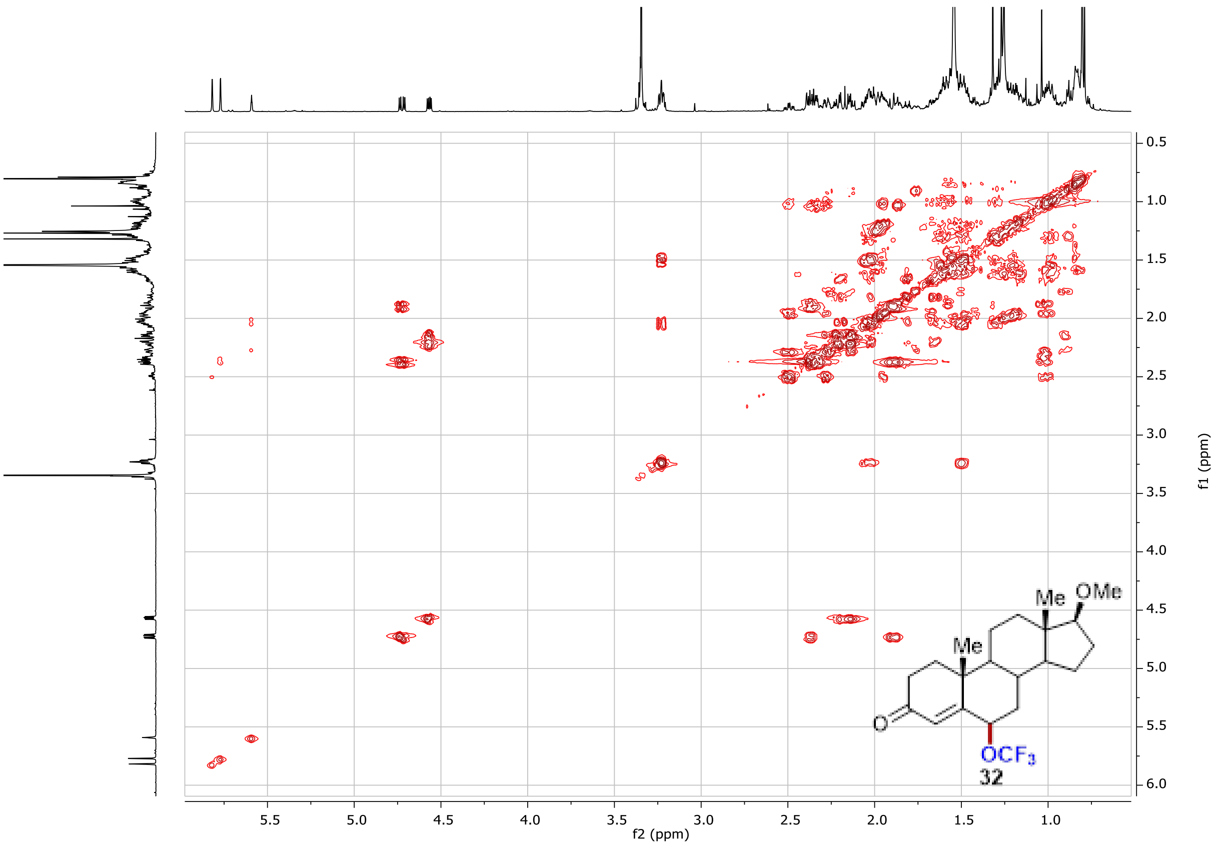

HSQC (600 MHz, $CDCl_3$)

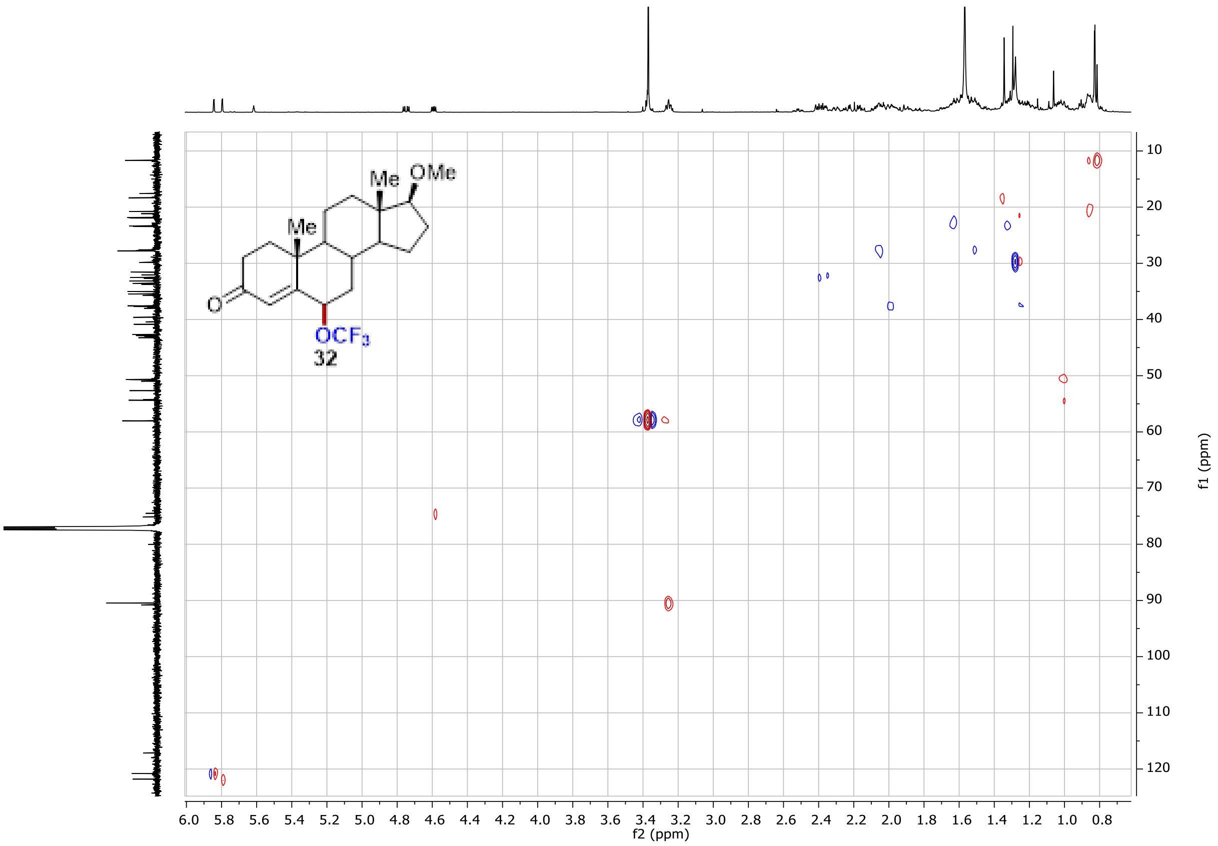

1-(4-bromophenyl)-2-(trifluoromethoxy)ethan-1-ol 33

^{1}H NMR (500 MHz, $CDCl_3$)

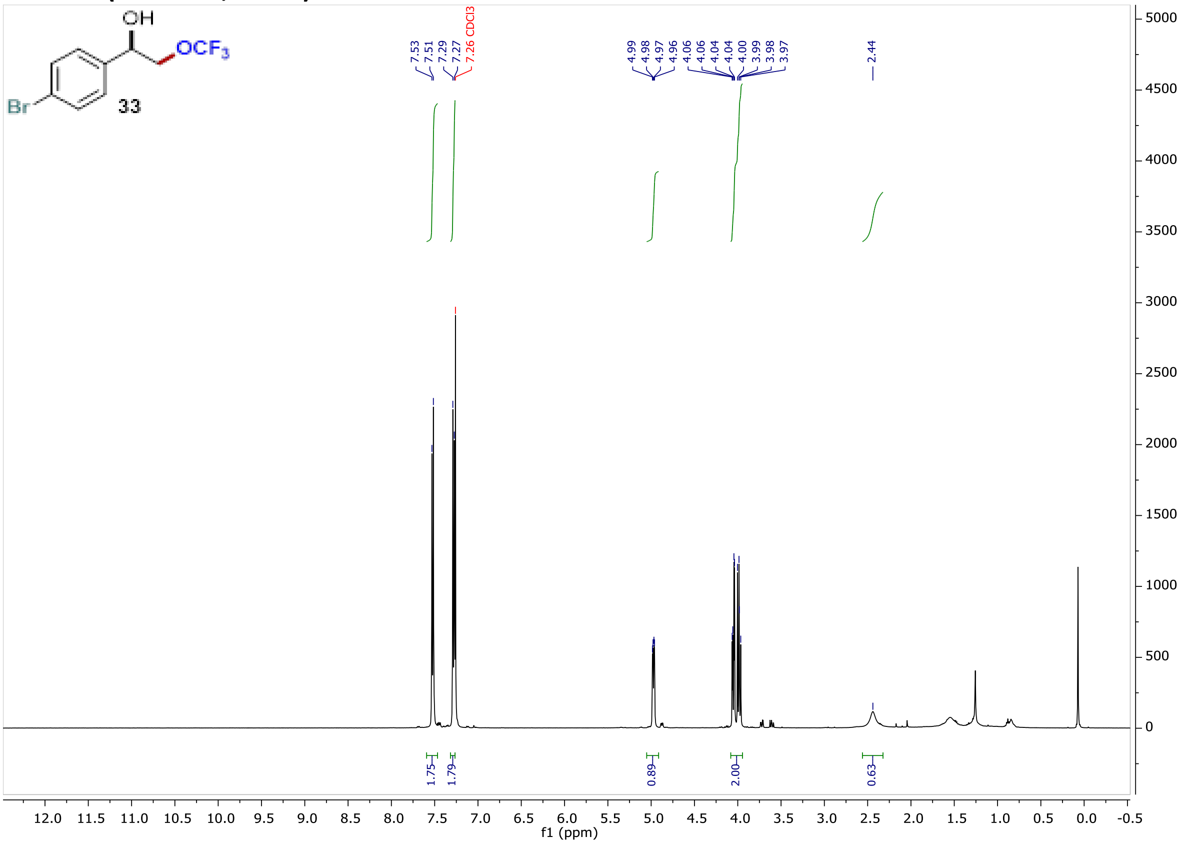

^{13}C NMR (126 MHz, $CDCl_3$)

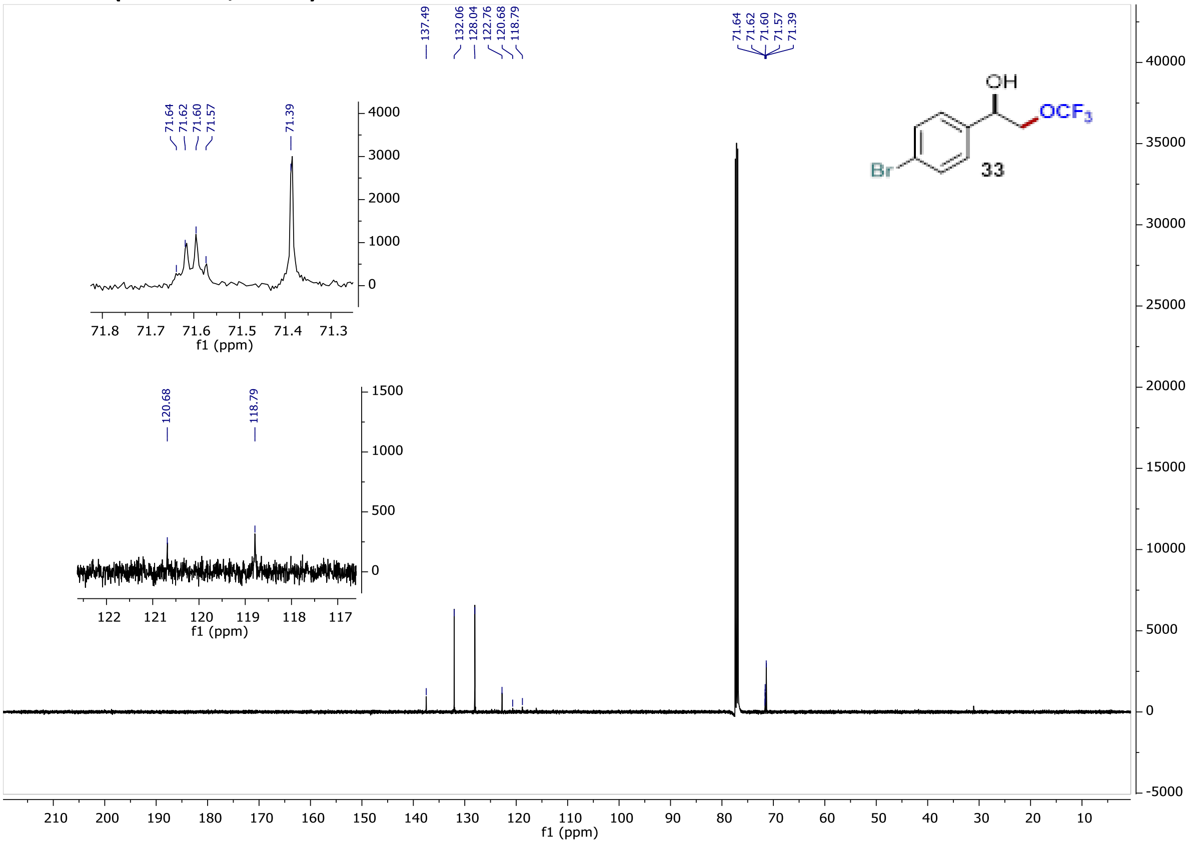

^{19}F NMR (188 MHz, $CDCl_3$)

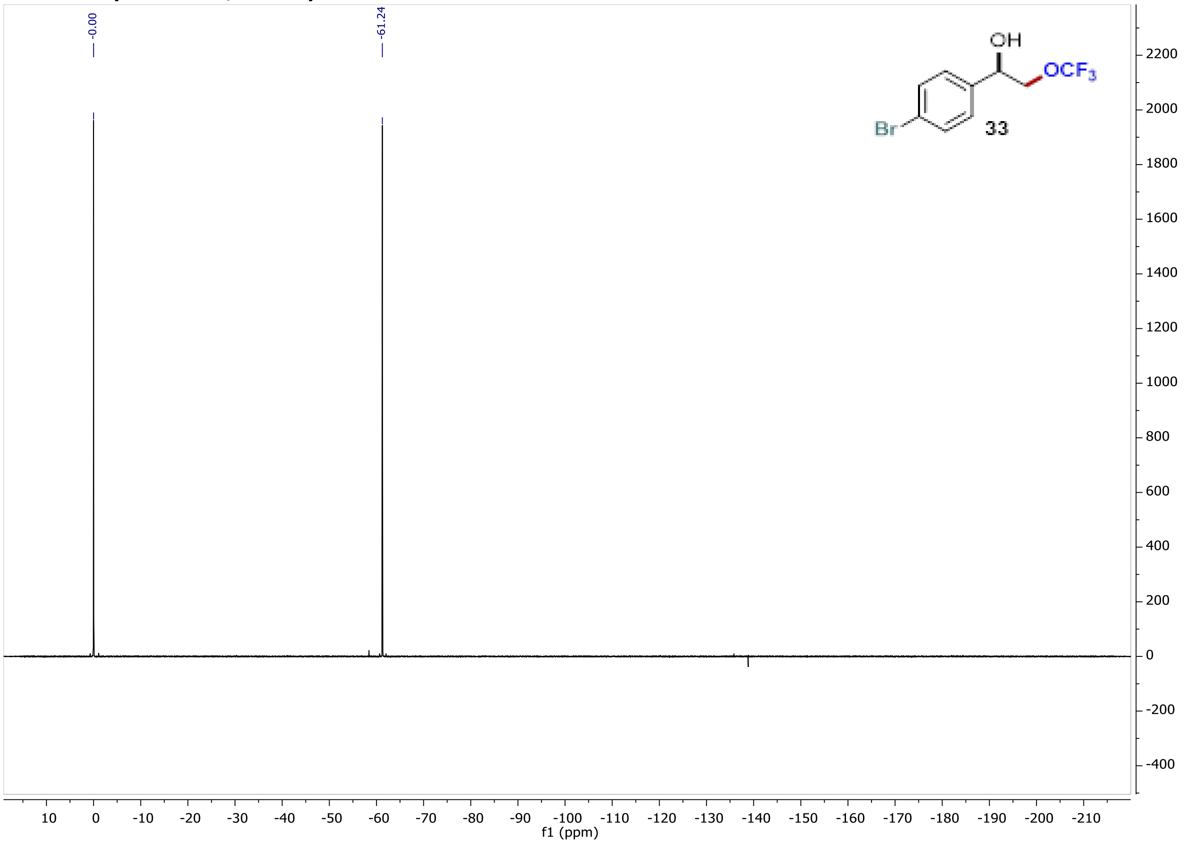

1-(4-morpholinophenyl)-2-(trifluoromethoxy)ethan-1-ol 34

^{1}H NMR (500 MHz, $CDCl_3$)

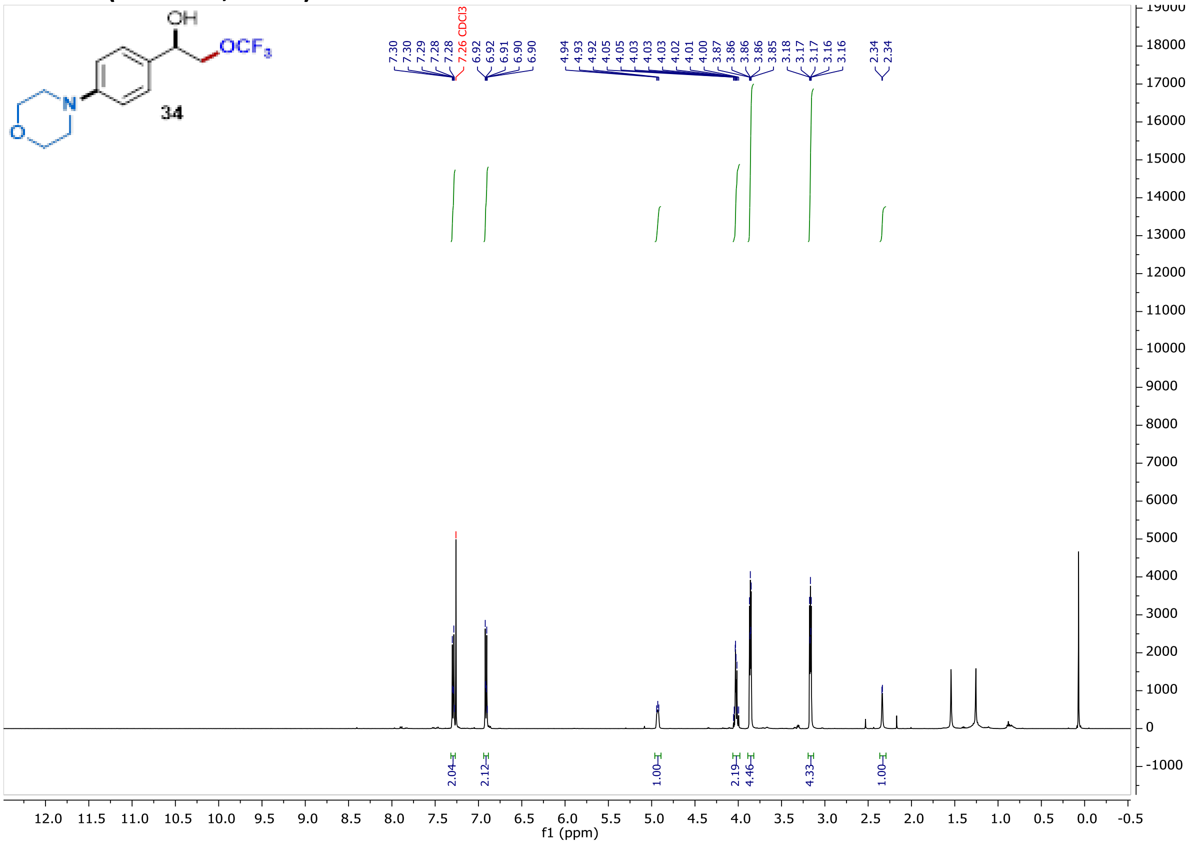

^{13}C NMR (126 MHz, $CDCl_3$)

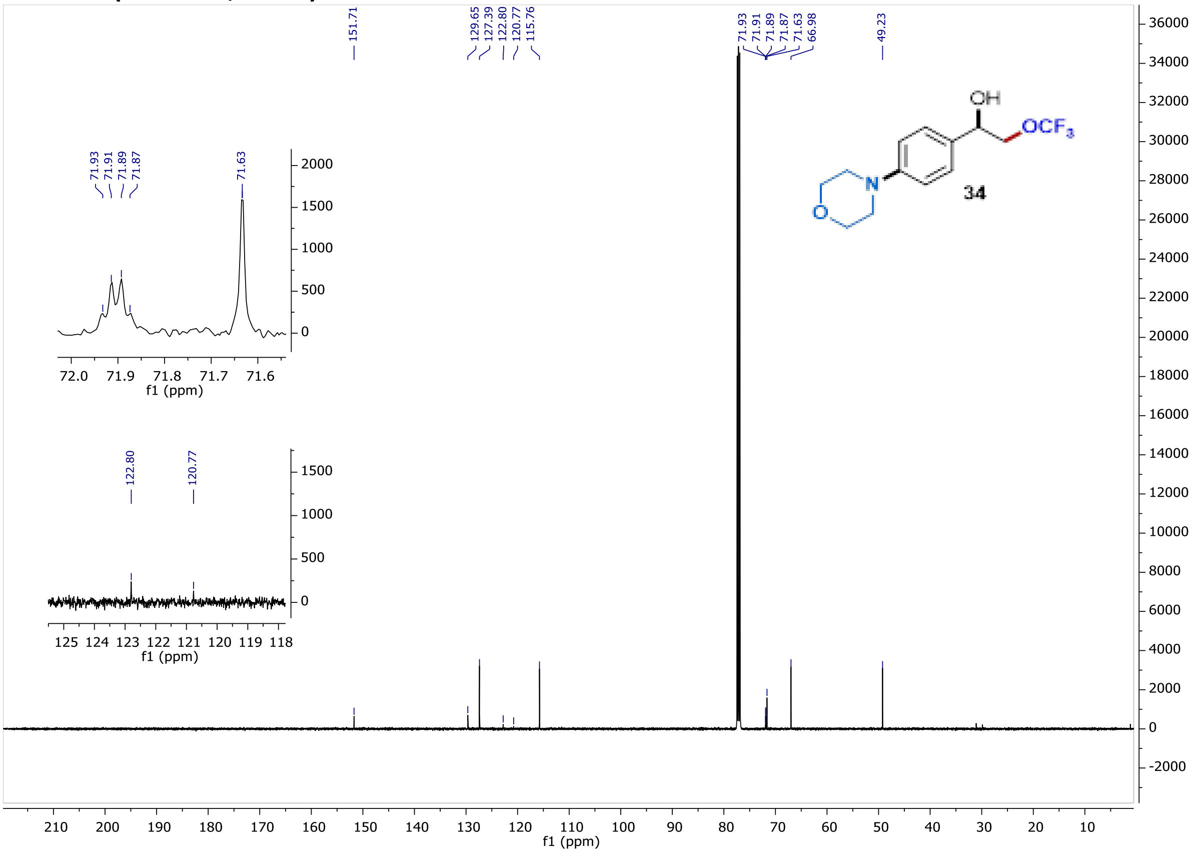

^{19}F NMR (188 MHz, $CDCl_3$)

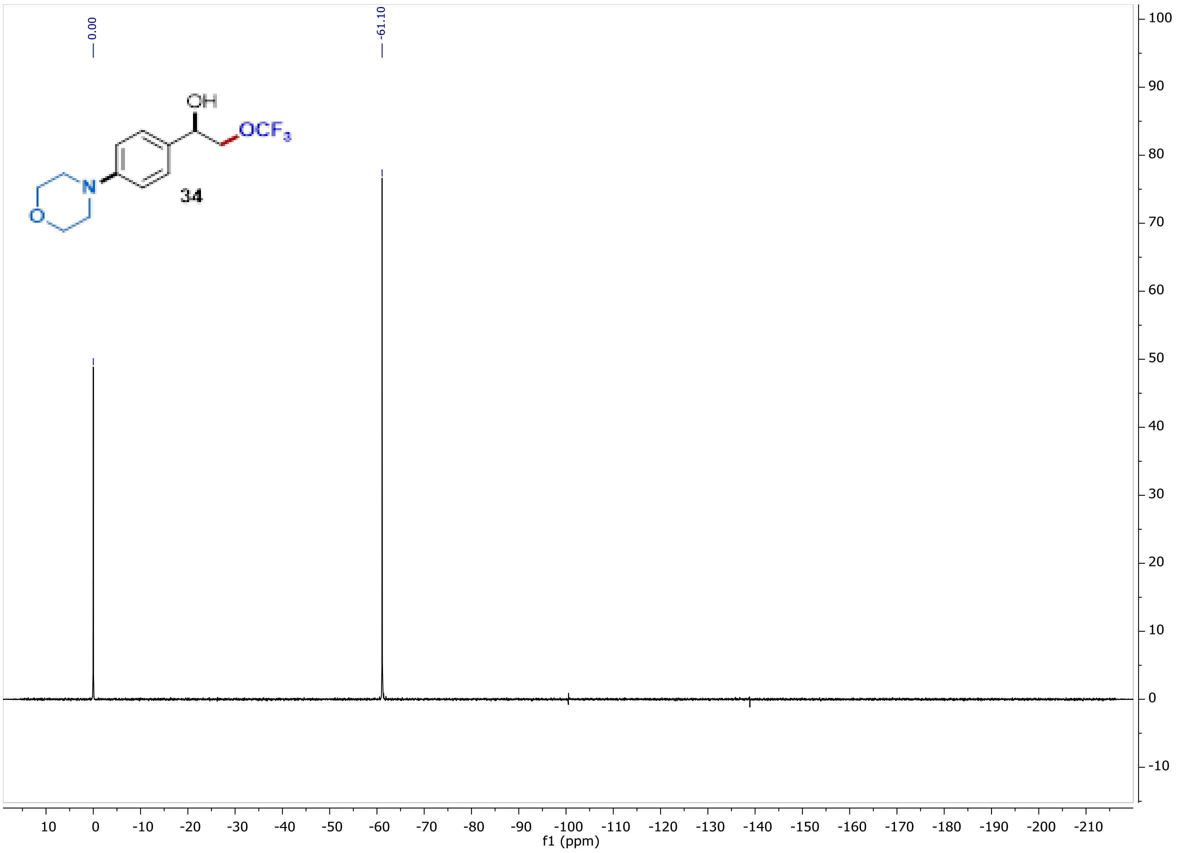

1-(4-bromophenyl)-2-(trifluoromethoxy)ethan-1-aminium acetate 35

^{1}H NMR (500 MHz, $CDCl_3$)

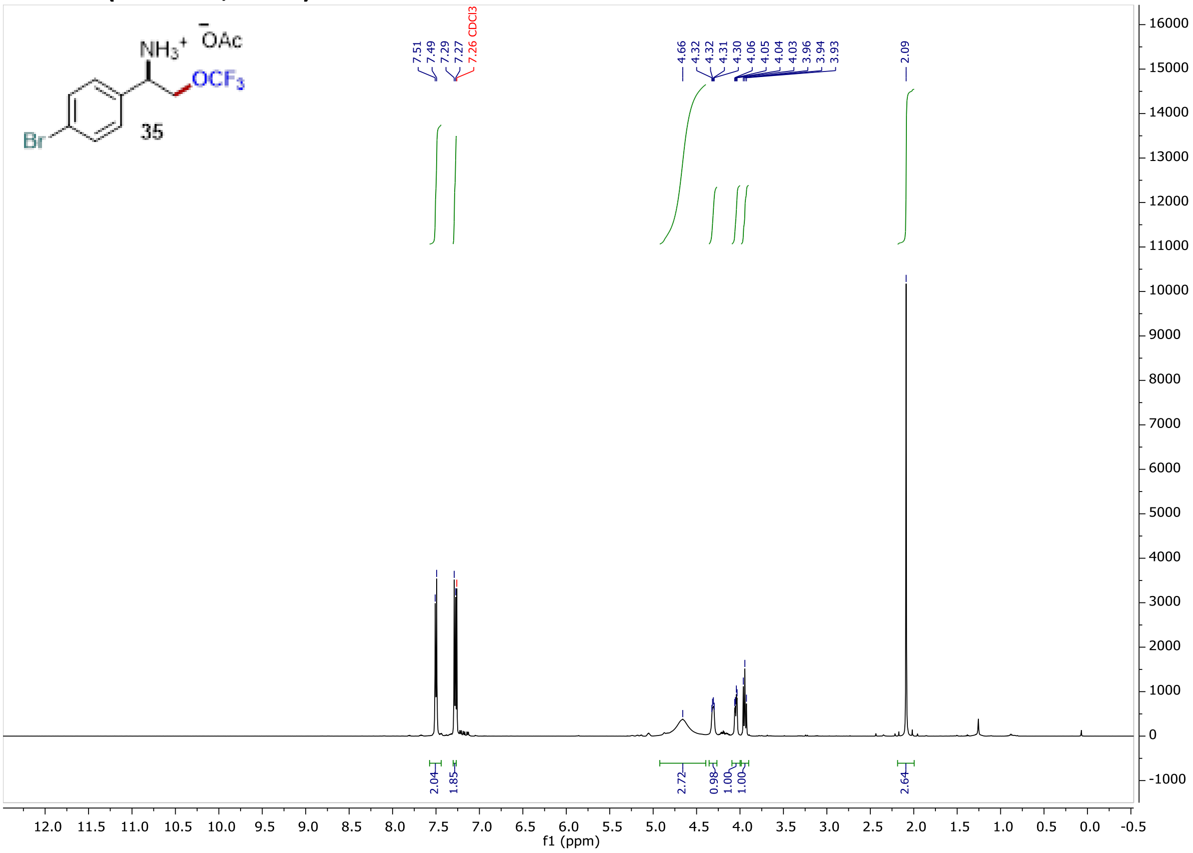

^{13}C NMR (126 MHz, $CDCl_3$)

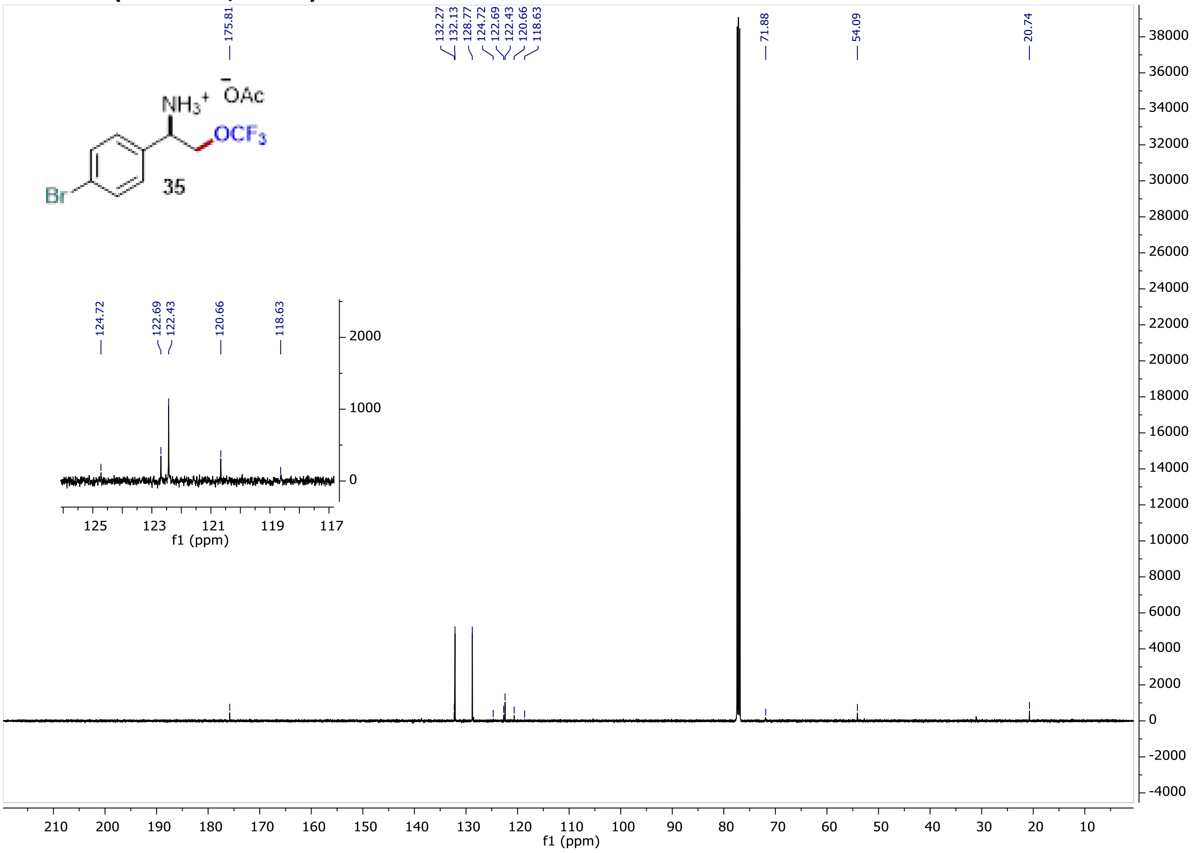

^{19}F NMR (188 MHz, $CDCl_3$)

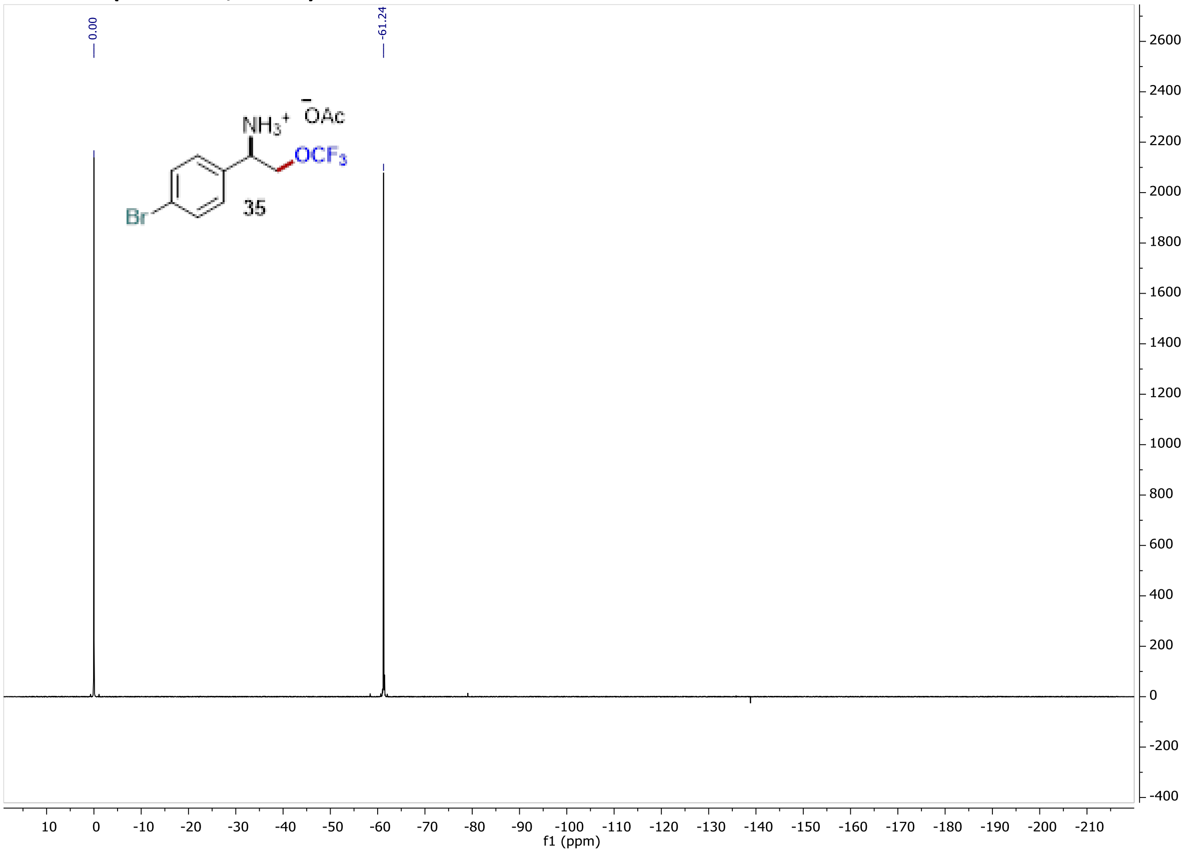

4-(3-(dimethylamino)-2-(trifluoromethoxy)acryloyl)benzonitrile 36

^{1}H NMR (300 MHz, $CDCl_3$)

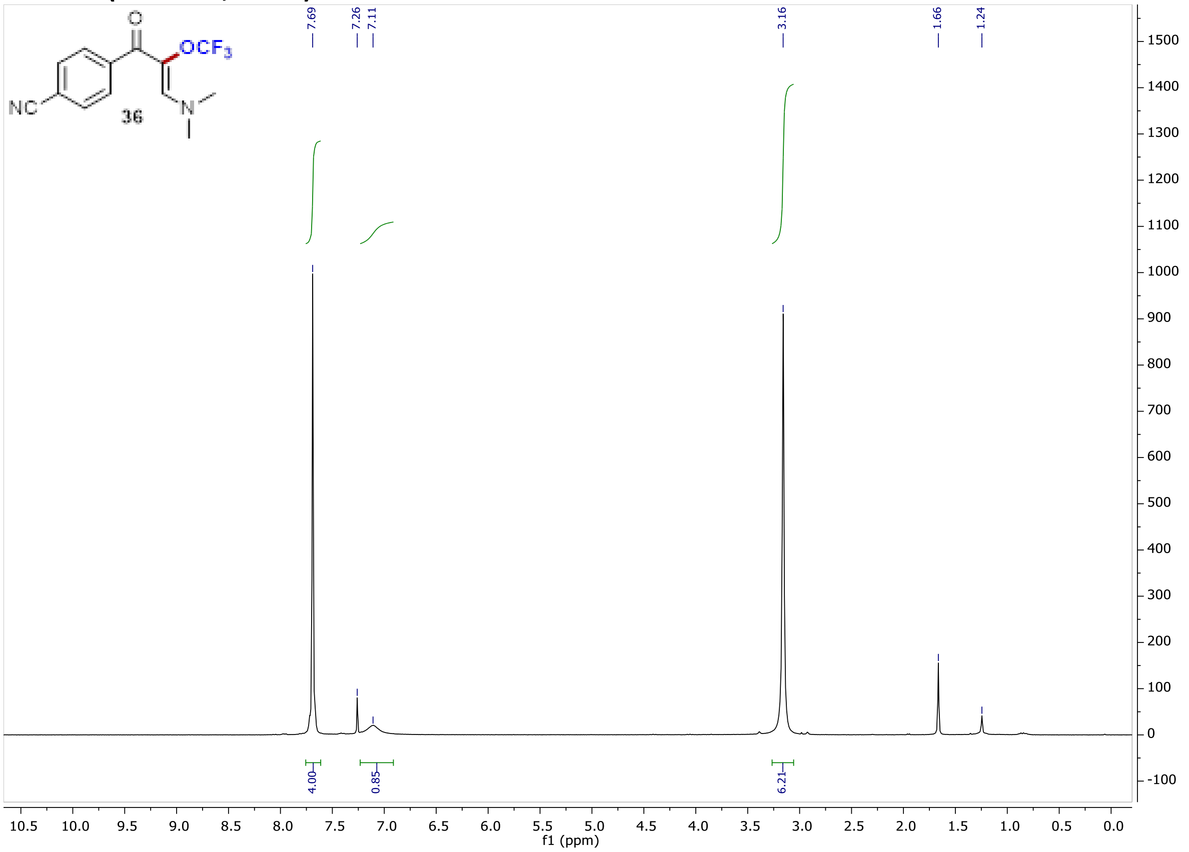

^{13}C NMR (75 MHz, $CDCl_3$)

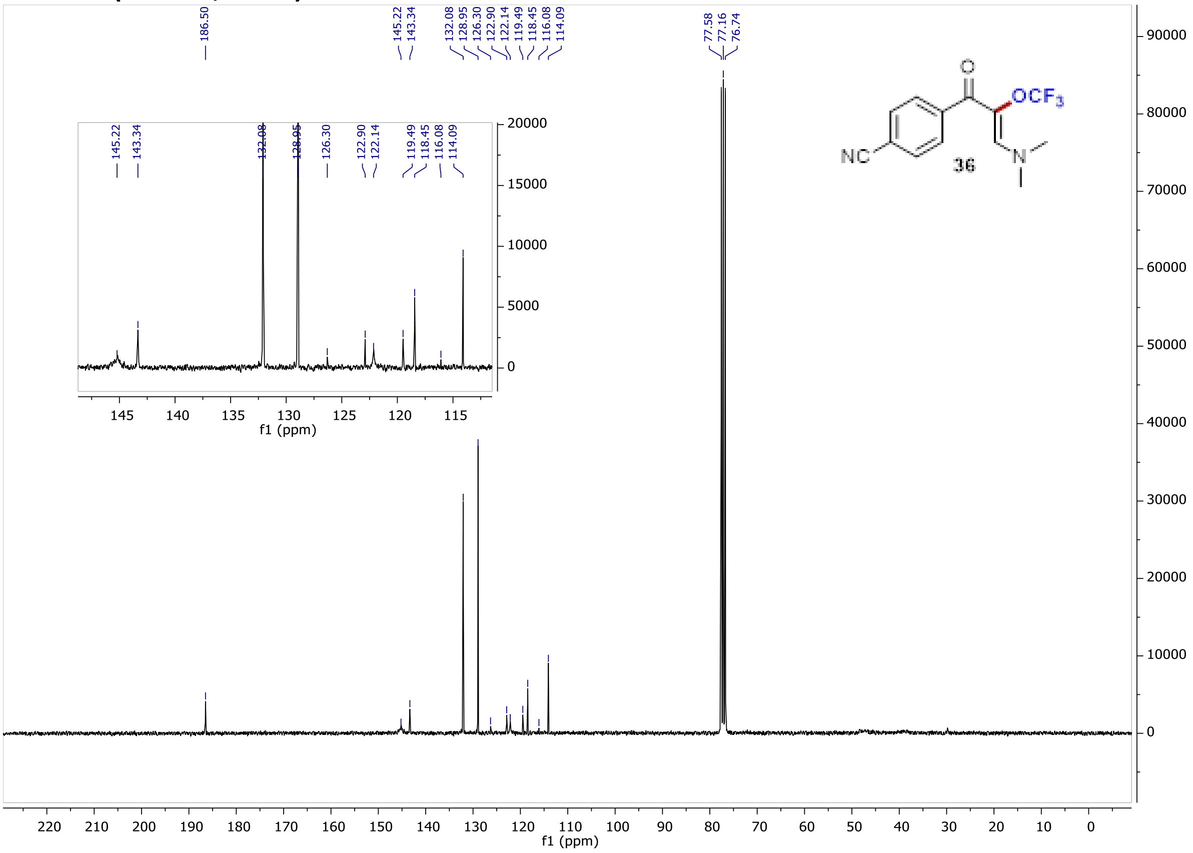

^{19}F NMR (188 MHz, $CDCl_3$)

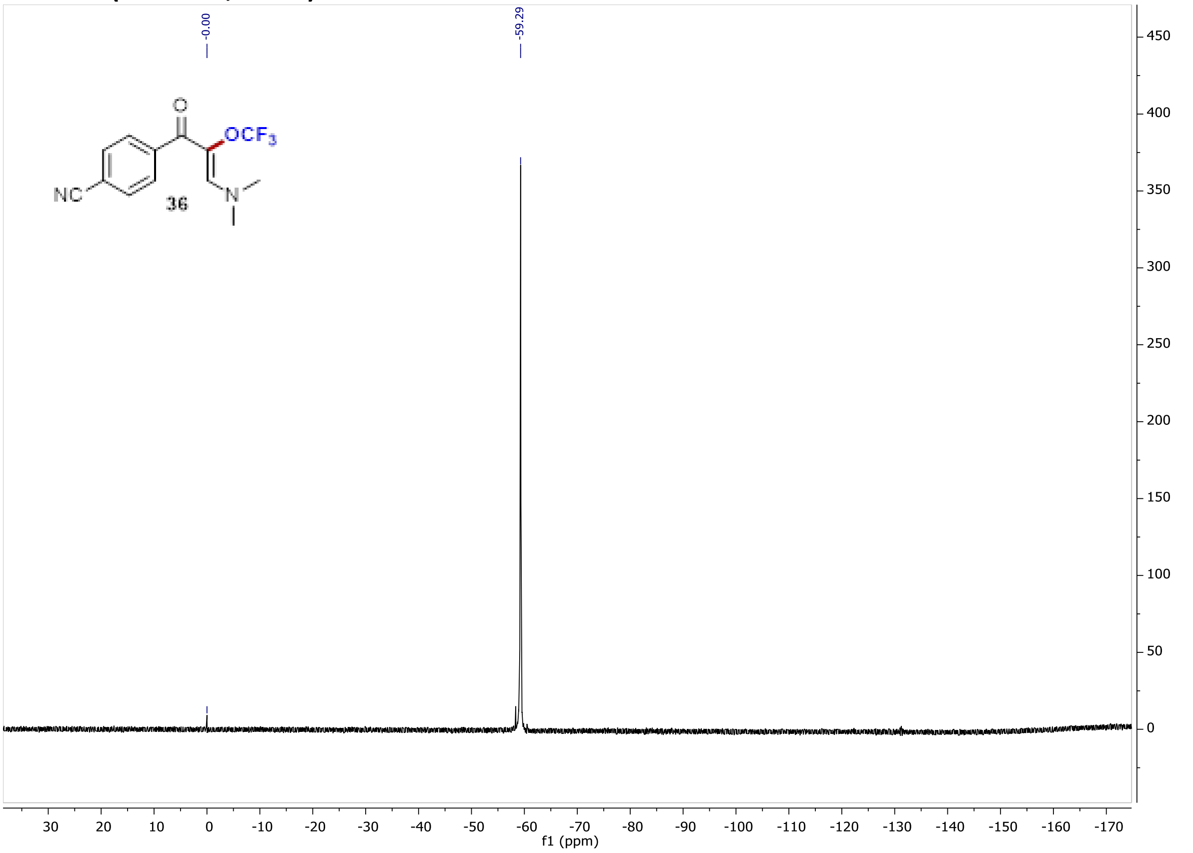

Supplement: Supplementary file 1 — ol1c02494_si_001.pdf [file ol1c02494_si_001.pdf]
